# Effector Polymorphisms of the Sunflower Downy Mildew Pathogen *Plasmopara halstedii* and Their Use to Identify Pathotypes from Field Isolates

**DOI:** 10.1371/journal.pone.0148513

**Published:** 2016-02-04

**Authors:** Quentin Gascuel, Amandine Bordat, Erika Sallet, Nicolas Pouilly, Sébastien Carrere, Fabrice Roux, Patrick Vincourt, Laurence Godiard

**Affiliations:** 1 Institut National de la Recherche Agronomique, INRA, Laboratoire des Interactions Plantes-Microorganismes (LIPM), Unité Mixte de Recherches UMR441, F-31326 Castanet-Tolosan, France; 2 Centre National de la Recherche Scientifique, CNRS, Laboratoire des Interactions Plantes-Microorganismes (LIPM), Unité Mixte de Recherches UMR2594, F-31326 Castanet-Tolosan, France; Agriculture and Agri-Food Canada, CANADA

## Abstract

The obligate biotroph oomycete *Plasmopara halstedii* causes downy mildew on sunflower crop, *Helianthus annuus*. The breakdown of several *Pl* resistance genes used in sunflower hybrids over the last 25 years came along with the appearance of new *Pl*. *halstedii* isolates showing modified virulence profiles. In oomycetes, two classes of effector proteins, key players of pathogen virulence, are translocated into the host: RXLR and CRN effectors. We identified 54 putative CRN or RXLR effector genes from transcriptomic data and analyzed their genetic diversity in seven *Pl*. *halstedii* pathotypes representative of the species variability. *Pl*. *halstedii* effector genes were on average more polymorphic at both the nucleic and protein levels than random non-effector genes, suggesting a potential adaptive dynamics of pathogen virulence over the last 25 years. Twenty-two KASP (Competitive Allele Specific PCR) markers designed on polymorphic effector genes were genotyped on 35 isolates belonging to 14 *Pl*. *halstedii* pathotypes. Polymorphism analysis based on eight KASP markers aims at proposing a determination key suitable to classify the eight multi-isolate pathotypes into six groups. This is the first report of a molecular marker set able to discriminate *Pl*. *halstedii* pathotypes based on the polymorphism of pathogenicity effectors. Compared to phenotypic tests handling living spores used until now to discriminate *Pl*. *halstedii* pathotypes, this set of molecular markers constitutes a first step in faster pathotype diagnosis of *Pl*. *halstedii* isolates. Hence, emerging sunflower downy mildew isolates could be more rapidly characterized and thus, assessment of plant resistance breakdown under field conditions should be improved.

## Introduction

The sunflower (*Helianthus annuus)* is the fourth most widely grown oil crop in the world after palm, soybean and rapeseed. In contrast to other oil crops, sunflower is environmental friendly, since it grows under low inputs (water, soil fertilizers and fungicides). Downy mildew caused by the oomycete *Plasmopara halstedii* is one of the major diseases affecting sunflower crop production (for review [1]). *Pl*. *halstedii* is a biotrophic and mainly homothallic oomycete belonging to the Peronosporales, and presents a cycle of one sexual generation during winter and up to two asexual generations during the growing season. *Pl*. *halstedii* has been described as a highly selfing species, and a high rate of homozygosity is observed in natural populations [1, 2]. First described in the United States [3], this disease was then reported worldwide mostly in areas where sunflowers were largely cultivated. *Pl*. *halstedii* infection may severely impact sunflower seed yield through either damping off of seedlings and reduction of plant population size, or ongoing disease symptoms which in turn induce dwarfing and sterility of heads. Since 1992, *Pl*. *halstedii* has been submitted to quarantine regulation in the European Union (directive 92/103/CEE).

*Pl*. *halstedii* isolates are classified into pathotypes (also called “races”), that are defined by an international nomenclature system based on differential virulence profiles on a set of sunflower inbred lines with different resistance patterns [4, 5] (S1 Fig). Pathotype determination of *Pl*. *halstedii* isolates is therefore based only on phenotypic tests performed in controlled growth chambers, on seedlings of defined sunflower differential lines, inoculated with the isolate of interest, and recorded as resistant (no leaf sporulation) or susceptible (leaf sporulation) two weeks after inoculation [1, 6].

Historically, downy mildew has been one of the major threats for the development of sunflower cultivation around the world. The discovery of the *Pl1* dominant gene allows protecting the crop for many years against the first pathotype ever mentioned, pathotype 100 [7]. However, the breakdown of several *Pl* resistance genes used in sunflower hybrids over the last 25 years came along with the appearance of new *Pl*. *halstedii* isolates showing modified virulence profiles [2, 8]. The number of recorded *Pl*. *halstedii* pathotypes increased from 1 in 1987 to 14 in 2011 [2]. For each of those 14 pathotypes, an isolate was chosen as representative of the pathotype and thereafter called “reference isolate”. These 14 reference isolates were classified into three distinct clusters according to CAPS (cleaved amplified polymorphic sequence) or SSCP (single-strand conformation polymorphism) markers designed on *Pl*. *halstedii* expressed sequence tags (ESTs) [9]. These clusters could have resulted from three independent *Pl*. *halstedii* introductions in France, and inter-cluster recombination could have facilitated the rapid emergence of pathotypes with increased virulence [2]. Sets of molecular diagnostic markers easy to handle are still lacking to characterize individually *Pl*. *halstedii* pathotype isolates. Identification of such markers should be a precise alternative (i) to discriminate *Pl*. *halstedii* into pathotypes more rapidly than with phenotypic tests handling living spores, (ii) to help monitoring sunflower field contaminations, and (iii) to follow the emergence of new field isolates.

For their developmental cycle, plant pathogenic oomycetes rely on pathogenicity factors called effectors that modify the metabolism of the host to their benefit and enable pathogenicity [10, 11]. Oomycete effectors are secreted by pathogens in plant cell apoplast via haustorial structures, and a signal peptide is usually identified in their N-terminal part [12]. In contrast to apoplastic effectors that stay in the apoplastic space, cytoplasmic effectors translocate into host cell cytoplasm and target different subcellular compartments to increase pathogen virulence [13]. Two major classes of cytoplasmic effectors have been identified in oomycete genomes and possess in their N-terminal region, conserved translocation domains RXLR-dEER and LXLFLAK for RXLR and Crinkler (CRN) effectors, respectively [14, 15]. RXLR and CRN effectors present a characteristic modular structure composed of an N-terminal conserved region followed by a C-terminal variable region carrying the putative biochemical effector activity [12]. The necrotic activity of several CRN has been shown to be dependent on their addressing to the nucleus of the host plant but the role of most CRN proteins is still unknown [14, 17–19]. While CRN effectors have been described in all oomycete species, RXLR effectors seem to be restricted to the Peronosporales group [14]. Several RXLRs have been reported as being recognized by plant resistance (R) genes or to act as suppressors of plant innate immunity [20–23]. Direct plant targets of RXLR proteins have been characterized in a few cases [10, 24–26].

Studies on the organization and dynamics of the effector repertoire at the genome level have been reported for some pathogens whose genomes have been completely sequenced. A comparison of 19 genomes of *Pseudomonas syringae*, a bacterial pathogen of many crop species, indicated both the presence of conserved (core) effectors and of a variable effector repertoire underlying differences in virulence across host plants, highlighting the dynamic role of effectors on virulence evolution [27]. Cytoplasmic effector genes of the oomycete *Phytophthora infestans* have been localized in regions characterized by low gene density and high density of transposable and repeated elements that favor mutations and recombination [16, 28]. In these dynamic repeat-rich regions, accelerated effector gene evolution has been shown to be associated with pathogen virulence adaptation in *P*. *infestans* lineage [29]. The emergence of an aggressive and invasive isolate of the oomycete *P*. *infestans*, breaking down potato blight resistance in Great Britain, has been associated with deletion and acquisition in its effector repertoire, especially RXLRs [28]. In addition, molecular variation in a particular downy mildew effector gene known to be essential for interaction with the plant *Arabidopsis thaliana* can provide insights on the evolution of pathogen virulence [30]. Polymorphism in effector sequences among pathogen isolates could therefore impact pathogen virulence thus creating a melting pot for new variants involved in host adaptation [30].

Molecular mechanisms underlying pathogen virulence are starting to emerge for model oomycete species [31], but are still unknown for *Pl*. *halstedii*. Despite the economic importance of sunflower downy mildew, genomic resources for *Pl*. *halstedii* were only recently obtained and its effector repertoire has just begun to be analyzed [1, 32].

In this study, we report the identification of 54 *Pl*. *halstedii* putative effector genes selected from transcriptomic data. Then, we describe the genetic diversity of these effectors in seven *Pl*. *halstedii* pathotypes representative of the species variability, this analysis suggesting a potential adaptive dynamics in pathogen virulence. Finally, we use Single Nucleotide Polymorphisms (SNPs) in effector genes to design 22 KASP (Competitive Allele Specific PCR) markers and genotype them in a set of 35 isolates belonging to 14 *Pl*. *halstedii* pathotypes. Using a combination of eight KASP markers, the eight multi-isolate *Pl*. *halstedii* pathotypes were classified into six groups, providing for the first time a set of molecular diagnosis markers for pathotype determination based on effectors.

## Results

### Identification of *Pl*. *halstedii* putative RXLR and CRN effectors

**Fig 1:**
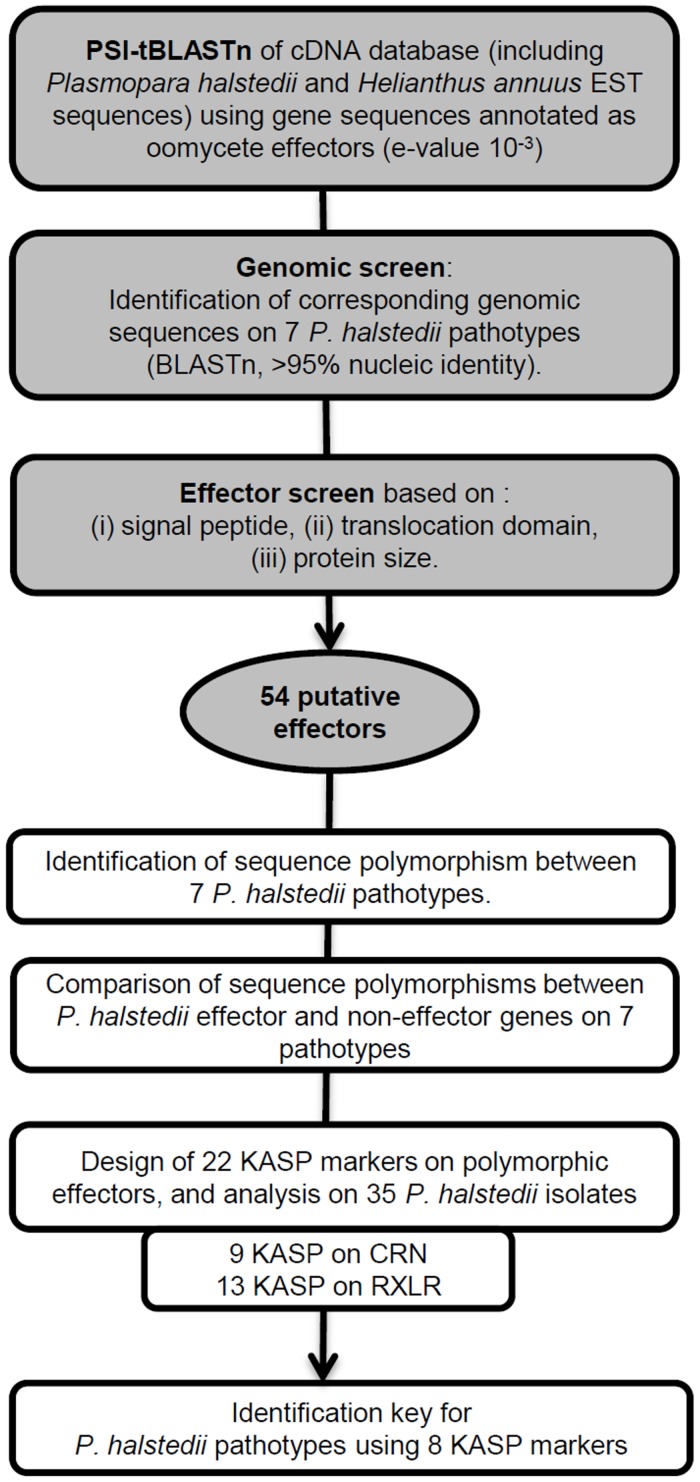
Workflow of the study. In grey, the procedure used to select candidate effector genes. Transcriptomic data from sunflower leaves infected by *Pl*. *halstedii* or isolated zoospores were analyzed by PSI-tBLASTn using the annotated sequences that were available in NCBI in March 2010 as models for RXLR and CRN effectors. In white, the procedure used to build the *Pl*. *halstedii* pathotype determination key. Identification of effector polymorphism was done by comparisons between genomic sequences obtained in 7 representative pathogen pathotypes (100, 300, 304, 334, 700, 703 and 710). Among the 22 KBioscience Competitive Allele Specific PCR (KASP) markers, eight were used in a determination key to discriminate *Pl*. *halstedii* pathotypes.

We identified 54 putative effector genes (Fig 1), hereafter named ‘effectors’, with 27 RXLR and 27 CRN listed on the Web page (S1 Table, Plasmopara halstedii effector polymorphism). Genomic sequences were found for 53 out of the 54 effectors in the seven representative pathotypes (100, 300, 304, 334, 700, 703 and 710), and PhRXLR41 was found in only three pathotypes (100, 300, 334). Of the 27 RXLR-type effectors, 11 have the RXLR consensus motif and three present an alternative RXLQ motif, previously shown as a functional translocation motif in secreted proteins of some oomycetes [33–35]. An EER motif was present after the RXLR motif in 11 effectors, whereas 13 effectors presented the EER motif alone. Of the 27 CRN-type effectors, seven have the LXLFLAK motif and six have an alternative LXLSLAK motif, each of them followed by the HVLVVVP consensus ending the conserved region of CRN effectors [16]. Five CRN present a LXLYLAK domain followed, by the HVLVVVP motif for three of them. Other CRN-type effectors have variant motifs (S1 Table, Plasmopara halstedii effector polymorphism). Presence of conserved effector motifs found in effectors from other oomycete species, and/or high PSI-tBLASTn values against the oomycete effector database (43 genes show e-values smaller than 10^−8^, S1 Table, Plasmopara halstedii effector polymorphism) suggest that these genes are reliable effector candidates.

### *Pl*. *halstedii* effector genes are more diverse at the non-synonymous level than random non-effector genes

**Fig 2:**
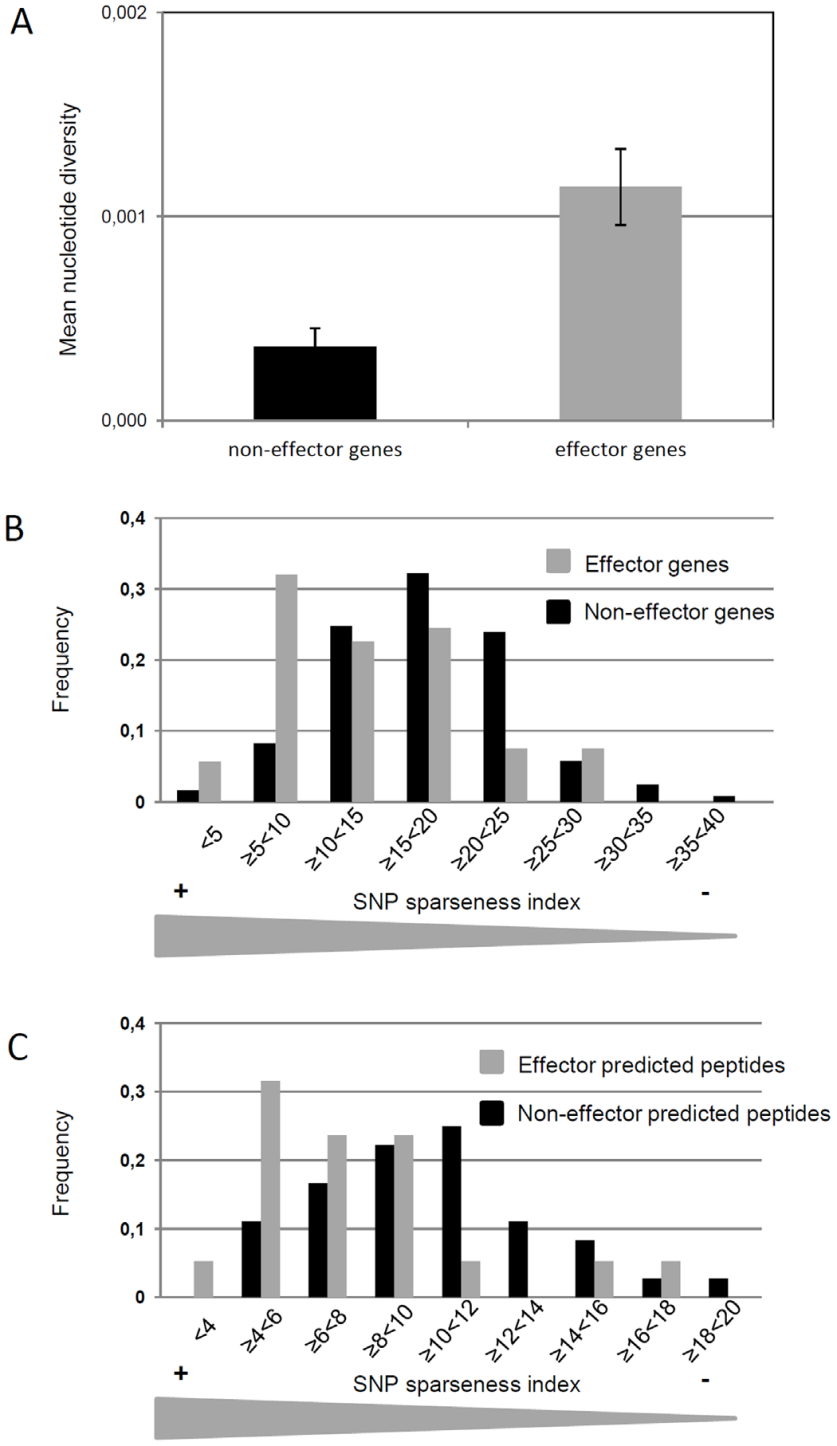
Polymorphism analysis of *Pl*. *halstedii* effector and non-effector genes. (A) Mean nucleotide diversity (π) calculated on non-effector genes (black bar) and effector genes (grey bar). Π values were calculated on DnaSP v5 software. Error bars represent two SEM. (B, C) Comparisons of polymorphism distributions (represented by SNP sparseness, i.e. minimum average distance between two polymorphisms) in *Pl*. *halstedii* effector (grey) and non-effector genes (black). (B) Frequency of genes in each class of index. (C) Frequency of predicted peptides in each class of SNP sparseness, among genes with nucleotide polymorphism.

While 18 effector genes presented no genomic polymorphism in the set of seven pathotypes (and in the set of 3 pathotypes for PhRXLR41), 36 effector genes (14 RXLR and 22 CRN) exhibited at least one SNP (S1 Table, Plasmopara halstedii effector polymorphism). To test whether the patterns of polymorphism observed overall on the 54 effector genes differ from the mean pattern of polymorphism in non–effector genes, we randomly selected 125 non-effector genes (see Materials and methods). For effector genes the percentage of polymorphic genes is twice as high as for non-effector genes (66.6% and 33.3% respectively). We also found that mean nucleotide diversity (π) was significantly more than three folds higher for effector genes than for non-effector genes (Mann-Whitney test, *P*-value = 5.82 E-08, Fig 2A). This result was supported with the SNP sparseness index (see Materials & Methods) which was found statistically higher in the class of effector genes than in the class of non-effector genes. Indeed, the comparison of the distributions for the index for NA (Kolmogorov-Smirnoff test, *P*-value = 2.34 E-05, Fig 2B) and AA (Kolmogorov-Smirnoff test, *P*-value = 3.55 E-04; Fig 2C) values, indicating that effector genes appeared to be on average more diverse at the non-synonymous level than non-effector genes.

### Localisation of polymorphism in *Pl*. *halstedii* effectors

Because of the previously described modular structure of effectors [12], we analyzed the polymorphisms in the N-terminal conserved and C-terminal variable regions classically found in effectors, with C-terminal regions starting after the effector motif 2 (S1 Table, EER motif for RXLRs and HVLVXXP motif for CRNs). For nine full size polymorphic RXLR proteins, 21 non-synonymous polymorphisms were localized in the variable region after the EER motif and only two in the conserved regions (S1 Table, Plasmopara halstedii effector polymorphism).

### Using effector polymorphisms to design KASP markers in order to characterize *Pl*. *halstedii* isolates

**Fig 3:**
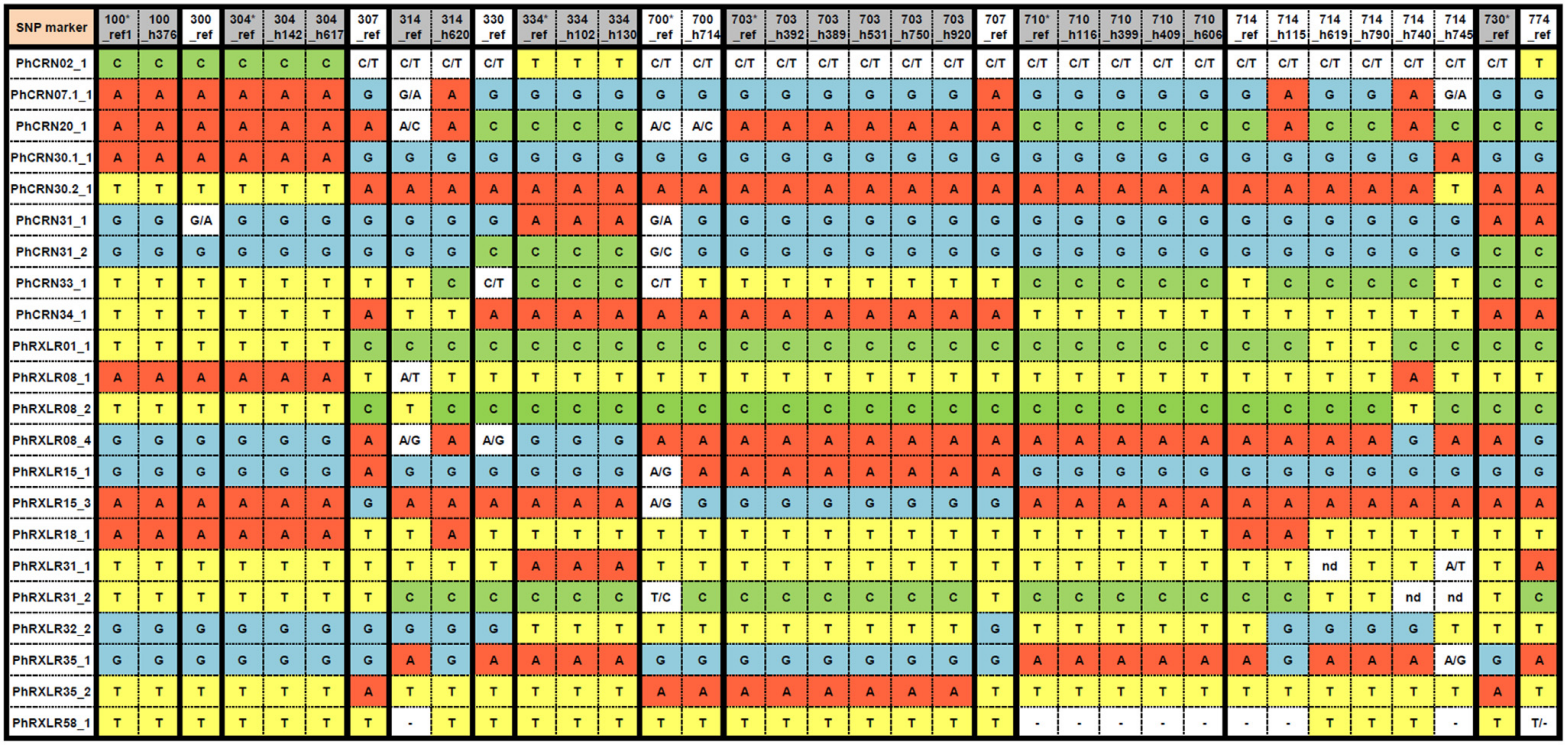
Genotypes of 14 reference isolates (_ref) and 21 geographical isolates of *Pl*. *halstedii* for 22 KASP markers based on effector gene SNPs. The DNA base involved in polymorphism is indicated by a specific colour. Heterozygous DNA bases are separated with a slash. (–) sign corresponds to the absence of the indel (versus T in PhRXLR58_1). *Genomic sequences of effectors available for these pathotypes (S1_File). Not determined results: nd.

Using housekeeping genes to design markers aiming to characterize *Pl*. *halstedii* was made difficult due to their low level of polymorphisms. Due to their high polymorphism levels and their putative role in pathogenicity, we reasoned that *Pl*. *halstedii e*ffector genes would be better candidates to design markers and to develop a high throughput genotypic set for discriminating pathotypes. We chose KASP (KBioscience Competitive Allele-Specific PCR) technology because it is suitable for every SNP and is user-friendly when genomic facilities are available. Twenty-two KASP markers were selected as non-redundant and revealed polymorphisms in 8 CRN and 8 RXLR effector genes (S1 Table, Plasmopara halstedii effector polymorphism, Fig 3).

To take into account possible variation among isolates within a given pathotype and to identify robust pathotype markers, the 22 KASP markers were genotyped on 21 additional *Pl*. *halstedii* isolates collected from different locations in France between 1993 and 2007, and belonging to 8 *Pl*. *halstedii* pathotypes (Table 1). For the remaining six pathotypes, only one isolate (*i*.*e*. the reference isolate) was available due to either absence (330), or paucity (300, 307,707) or recent emergences in France (730, 774) [2].

**Table 1:** *Plasmopara halstedii* geographical isolates used in this study.

*Pl*.*halstedii* isolate	Pathotype	Collection site in France	Year of collection
h376	100	Drôme	1993
h142	304	Gers	2006
h617	304	Puy-de-Dôme	2007
h620	314	Puy-de-Dôme	2007
h102	334	Charente	2004
h130	334	Charente-Maritime	2004
h714	700	Gers	2007
h392	703	Lot-et-Garonne	1993
h389	703	Gers	1993
h531	703	Gers	2007
h750	703	Tarn-et-Garonne	2007
h920	703	Gers	2007
h116	710	Meuse	2004
h399	710	Allier	1993
h409	710	Maine-et-Loire	1993
h606	710	Puy-de-Dôme	2007
h115	714	Charente	2004
h619	714	Puy-de-Dôme	2007
h790	714	Puy-de-Dôme	2007
h740	714	nd	2007
h745	714	nd	2007

Pathotype, collection site in France (“département”) and year of collection are indicated. nd: not determined.

Two types of KASP markers were revealed by the genotyping of *Pl*. *halstedii* multi-isolate pathotypes (Fig 3). For a given pathotype, either the same allele was found in each isolate, hereafter named intra-pathotype monomorphic markers; or at least two alleles were found between the different isolates, hereafter named intra-pathotype polymorphic markers. Only 3 markers (*i*.*e*. PhCRN02_1, PhCRN34_1 and PhRXLR35_2) were monomorphic in every multi-isolate pathotype. The 19 others showed intra-pathotype polymorphism in at least one of the eight multi-isolate pathotypes. For instance, the PhRXLR01_1 marker was monomorphic within each multi-isolate pathotype with the exception of pathotype 714.

Finally, five (100, 304, 334, 703, 710) out of the eight multi-isolate pathotypes, had only intra-pathotype monomorphic KASP markers, while the remaining three pathotypes (314, 700 and 714) have at least one intra-pathotype polymorphic marker. For example, while the six isolates of pathotype 703 showed no intra-pathotype polymorphism, the six isolates of pathotype 714 presented five different allelic profiles (two isolates having the same genotypic profile).

The majority of the effector markers were homozygous in most of the 35 *Pl*. *halstedii* isolates. Heterozygous profiles were observed for the two reference isolates 314 and 700 (five to eight markers) and to a lesser extent for the 330 and 714_h745 isolates (three to four markers). KASP marker PhCRN02_1 revealed amplifications of two different alleles for most isolates (25/35) (Fig 3), suggesting either PCR amplification of paralogs or positive selection of the heterogyzous status at one single locus. One CRN (PhCRN31) and four RxLR (PhRXLR08, PhRXLR15, PhRXLR31, PhRXLR35) effectors carry two non-redundant KASP markers per gene showing different allelic combinations among pathotypes, and thereby suggesting the occurrence of intragenic recombination in these five effector genes (Fig 3).

### Construction of an identification key for *Pl*. *halstedii* pathotypes

**Fig 4:**
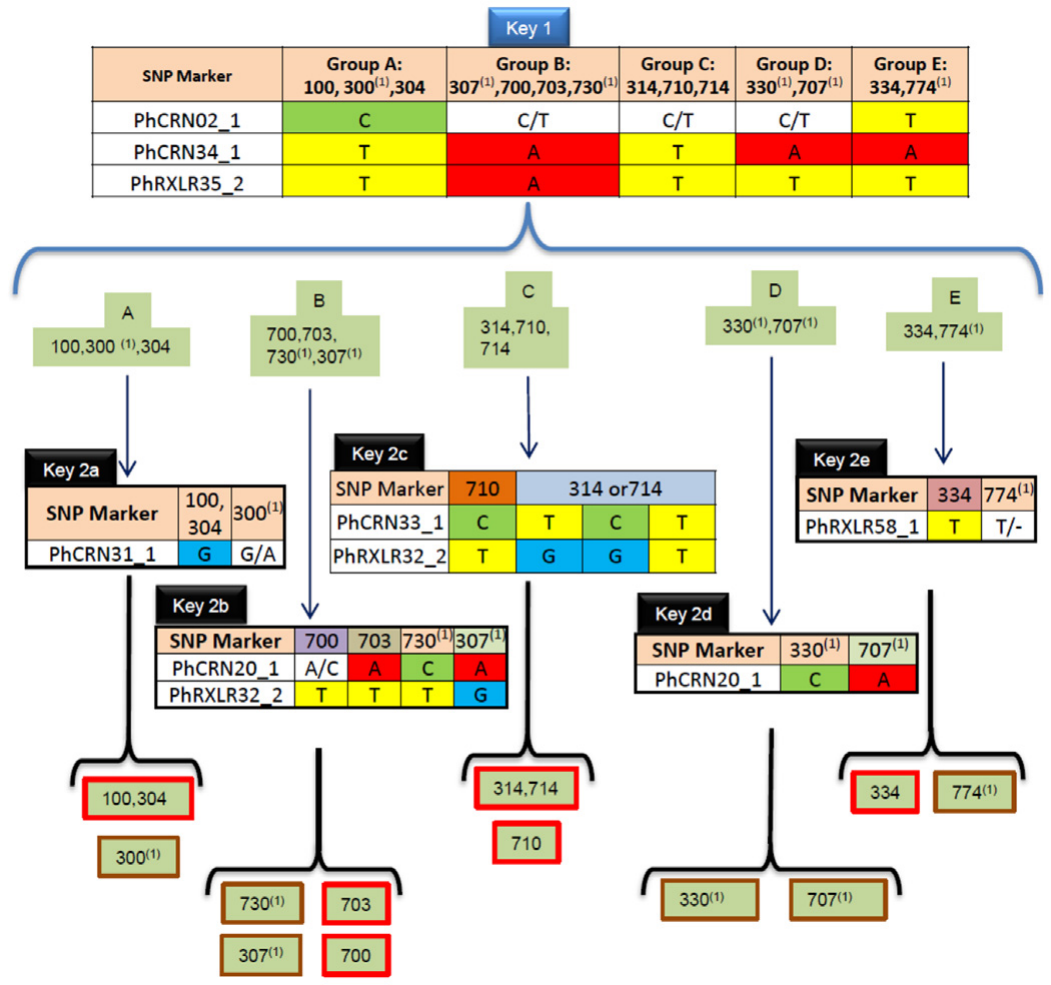
Identification key for *Pl*. *halstedii* pathotypes using KASP markers designed on effector gene SNPs. First level (Key 1) separated the 14 *Pl*. *halstedii* pathotypes in 5 groups with 3 markers. Second level (Key 2) used five other markers to distinguish 12 subgroups of pathotypes, and especially 6 subgroups of multi-isolate pathotypes (Red boxes). 100 and 304, 314 and 714 pathotypes could not be distinguished. ^1^*Pl*. *halstedii* pathotypes with only one isolate available.

Based on KASP genotyping data, a two-level key was constructed in order to improve further pathotype identification of field isolates (Fig 4). At the first level (Key 1), three markers with no intra-pathotype polymorphism, PhCRN02_1, PhCRN34_1 and PhRXLR35_2, were used. Key 1 separated the 14 *Pl*. *halstedii* pathotypes into five groups (group A = 100, 300, 304/ group B = 307, 700, 703, 730/ group C = 314, 710, 714/ group D = 330, 707/ group E = 334, 774).

At the second level (Key 2), we used intra-pathotype monomorphic markers within each group of Key 1, without considering their genotypes in other groups. PhCRN31_1 was used to distinguish pathotypes in group A (i.e. pathotypes 100 and 304 from pathotype 300), PhCRN20_1 and PhRxLR32_2 to distinguish the four pathotypes of group B, PhCRN33_1 and PhRxLR32_2 to distinguish pathotype 710 from the other pathotypes of group C. Finally, PhRxLR58_1 and PhCRN20_1 were used to distinguish pathotypes within groups D and E, respectively.

Pathotypes 100 and 304 from group A and pathotypes 314 and 714 from group C cannot be separated by the 22 KASP markers tested.

Based on eight KASP markers designed on five CRN and three RXLR effectors, the proposed identification key classified the eight multi-isolate pathotypes into six groups (Fig 4). Including the mono-isolate pathotypes, the key distinguished 10 *Pl*. *halstedii* pathotypes and two groups of two pathotypes. Considering the current occurrence of some pathotypes in crop fields [2], especially the scarcity of some mono-isolate pathotypes (300, 330, 774) or the absence of detection during the last three decades of the first observed pathotype 100 that we could exclude from the identification key, the proposed marker set is adapted to differentiate 11 out of the 13 currently detectable *Pl*. *halstedii* pathotypes.

### Are effector genotyping data linked to virulence profiles of *Pl*. *halstedii* pathotypes?

Considering this key, we hypothesized that effector polymorphisms could be linked to virulence profiles of *Pl*. *halstedii* pathotypes. To test this hypothesis, we used the Mantel procedure to estimate the coefficient of correlation between the matrix of pairwise genetic distances between pathotypes calculated from KASP genotyping data (Fig 3), and the matrix of pairwise phenotypic distances between the same pathotypes, calculated from their specific responses when interacting with the nine sunflower differential lines (D1 to D9, S1 Fig). Phenotypic distances were significantly correlated with genetic distances (Spearman correlation *rho* = 0.365, p-value = 0.0066; S1 Fig). This result suggests that our set of effector genotyping data is representative of *Pl*. *halstedii* virulence profiles.

**Fig 5:**
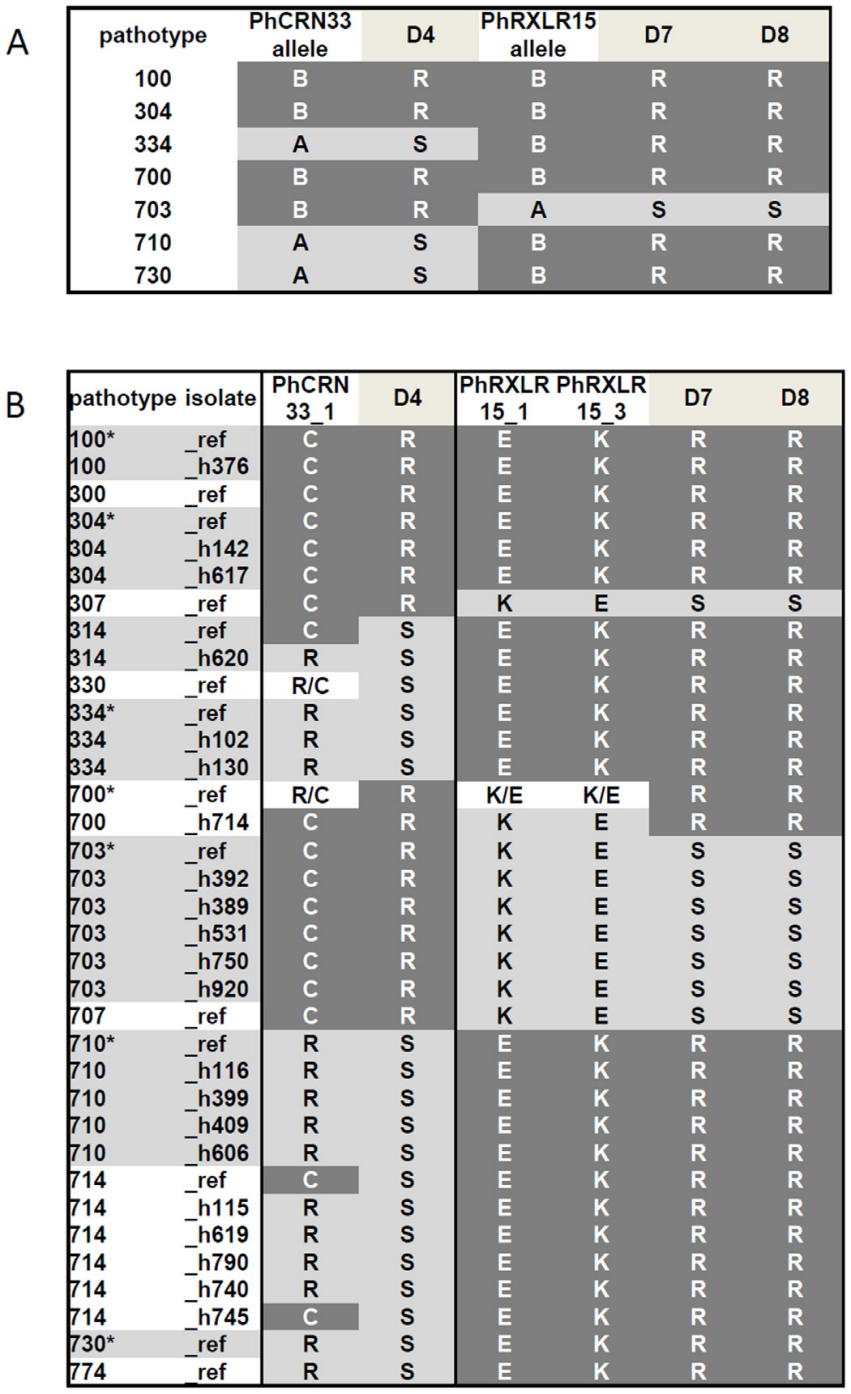
Putative associations between resistance (R) or susceptibility (S) profiles of sunflower lines (D4, D7 and D8) and genetic profiles of effector markers (PhCRN33 and PhRXLR15) in *Pl*. *halstedii* pathotypes from sequenced data (A) and from KASP marker results (B). (A) PhCRN33 and PhRXLR15 type B sequenced alleles from 7 pathotypes (100, 304, 334, 700, 703, 710 and 730) were respectively associated with resistance profiles of D4, D7, and D8 sunflower lines. (B) Respective associations of PhCRN33_1 and PhRXLR15_1-PhRXLR15_3 observed polymorphisms in 35 *Pl*. *halstedii* isolates with resistance (R) profiles of D4, D7, and D8 sunflower lines. The amino acids of the corresponding translated sequences are indicated for each SNP position.

More precisely, we found a strict association between the haplotype sequences of the seven pathotypes, for two effector markers PhCRN33 and PhRXLR15, and the resistance status of differential sunflower lines (Fig 5A). The susceptibility of differential line D4 (PMI3) to the pathotypes 334, 710 and 730 was associated with the presence of an arginine (R) in the A allele of PhCRN33 instead of a cysteine (C) in the B allele (AA 142) (https://www.heliagene.org/P.halstedii/effector_polymorphisms/Plhal040004_to_PLHAL.all.AA.gif). The protein sequence of PhRXLR15 in pathotype 703 showed four amino acid changes (positions 76, 91, 99 and 105) compared to the sequence of the 6 other pathotypes (https://www.heliagene.org/P.halstedii/effector_polymorphisms/Plhal011563_to_PLHAL.all.AA.gif). The rare (A) and frequent (B) alleles of PhRXLR15 were respectively associated with susceptibility and resistance of D7 (HAR4) and D8 (QHP1) lines (Fig 5A). Since two KASP markers on PhRXLR15 effector (i.e. PhRXLR15_1 and PhRXLR15_3 corresponding to AA 76 and 105, respectively) and one KASP marker on PhCRN33 (i.e. PhCRN33_1 corresponding to AA 142) have been genotyped (Fig 3), we compared the genotyping data provided on 35 *Pl*. *halstedii* isolates to the resistance status of D4, D7 and D8 sunflower lines (Fig 5B). For the PhRXLR15 effector, the glutamic acid (E) to lysine (K) reciprocal changes present in allele A, were found in pathotypes 307 and 707, which are also virulent on the D7 and D8 differential lines. For the PhCRN33 effector, the arginine (R) was found in most isolates of the 714 pathotype as well as in pathotypes 730 and 774, all of these pathotypes being virulent on line D4. However, these rules could not be applied to the heterogeneous pathotype 314 (either R or C in amino acid sequences) or to pathotype 330, which showed heterozygosity at PhCRN33_1 marker. While interesting, these results clearly need to be confirmed by thorough functional validation, for example by *Agrobacterium* mediated-overexpression of the two allelic forms of a specific effector in appropriate sunflower lines. Depending of their resistance profile, a differential phenotypic response (i.e. cell death *versus* no symptoms) could be observed in the case of effector recognition. This procedure is commonly used as a screen for oomycete effector identification or as a demonstration of an interaction between an effector and a resistance protein [10–13; 20; 22–23].

## Discussion

The rapid modification of *Pl*. *halstedii* virulence profile illustrated by the occurrence of new pathotypes breaking down *Pl* resistance genes is an important issue for the cultivation of sunflower crop. Use of housekeeping genes to design molecular markers, with the goal of characterizing different pathotypes, was hampered by recent *Pl*. *halstedii* diversification. Here, we focused on effector genes, which have been characterized as key determinants of pathogen virulence in model oomycetes, to set up molecular markers necessary to improve *Pl*. *halstedii* pathotype determination, which in turn will facilitate the epidemiology survey of this field disease.

### How to select *Pl*. *halstedii* reliable effector genes from *in silico* data?

A preliminary work on a smaller *Pl*. *halstedii* transcriptome database, called HP [32] led to the discovery of 5 RXLR and 15 CRN cDNA clusters, from which 7 CRN were polymorphic. Compared to this smaller database which counted at least 425 *Pl*. *halstedii* genes, our study was done on a larger database allowing the identification of 54 putative effector genes. In our current analysis, 13 of the 20 previously described effectors were selected [32]. This ratio might result from the drastic selection of genomic sequences in the seven pathotypes and effector screen we included to our selection procedure. Compared to effector repertoires published for model oomycetes, the present effector list is likely to be not exhaustive, but was sufficient to find polymorphic effectors among sequenced *Pl*. *halstedii* pathotypes.

To minimize exclusion of false negative effectors, we selected effector sequences that fulfilled at least two of the three criteria used in this study (see Materials and methods section), thereby allowing to a certain degree of freedom of choice. For example, none of the 27 *Pl*. *halstedii* selected CRN-type effectors showed canonical signal peptides, but all of them had high homologies with known CRN from other oomycetes and showed conserved motifs [16]. CRN effectors from *Phytophthora capsici* also did not contain canonical signal peptides and yet were shown to be translocated to the host cell, suggesting that they were secreted out of the oomycete [18]. In the case of RXLR-type effectors (i) which are globally less conserved among oomycetes, as suggested by lower alignment P-values, and (ii) whose conserved motifs are smaller than in CRN-type effectors, we considered the signal peptide possession as a good predictor (25 out of 27 selected). Because it has been shown that the ATR5 effector from *H*. *arabidopsidis* is translocated to the plant cell with the unique EER motif [36], we also kept *Pl*. *halstedii* RXLR-type effectors with solely the EER motif.

### Invariable effectors could be essential for pathogen virulence: a path to sustainable resistance?

In this study, a subgroup (18 out of 54) of monomorphic effectors was identified among seven *Pl*. *halstedii* pathotypes. Invariable (core) effectors from the phytopathogenic bacteria *Ps*. *syringae* were identified among 19 strains affecting different plant hosts [37]. Some of them were shown to inhibit a general plant response like antimicrobial vesicle trafficking and modulating plant immunity. Plants may therefore perceive pathogen attack by sensing their own disrupted cellular processes (i.e. vesicle trafficking) rather than by effector recognition *per s*e. These core effectors may be ancestral and subject to purifying selection because of their obligate role in pathogen virulence [37].

By analogy, *Pl*. *halstedii* monomorphic effectors may be involved in basic virulence functions necessary to invade a wide range of sunflower hosts. Functional studies of those monomorphic effectors may help in identifying plant targets and their corresponding disrupted cellular processes. Identification of plant resistant genes targeting monomorphic effector proteins may provide new tools for more sustainable resistance [38–39].

### High rates of non-synonymous polymorphisms in *Pl*. *halstedii* polymorphic effector genes suggest they might have a role in pathogen adaptation to the host

Plant pathogen populations could evade host recognition in different ways, all based on a polymorphic and dynamic effector system. Firstly, some effectors can suppress resistance mechanisms triggered by other effectors, as previously shown in the interaction between *Phytophthora sojae* and soybean [17]. Secondly, effectors from oomycete or fungi could escape host recognition by the appearance of non-synonymous mutations [30,40–41]. Finally, pathogens could modify their effector arsenal in a way to avoid the activation of the plant immune system, either by gain and loss of effector genes or by modifying their expression [28–29,42]. Contrary to monomorphic effectors, polymorphic effectors are supposed to be directly perceived by the plant recognition system and therefore expected to be under positive selection.

*Pl*. *halstedii* polymorphic effector genes presented a higher non synonymous diversity than non-effector genes, suggesting an on-going adaptive dynamics of the effector repertoire with the selection of new alleles to circumvent plant resistance. Studying the molecular evolution of those polymorphic effector genes on a wider set of *Pl*. *halstedii* isolates would certainly help in identifying effectors under positive selection.

Non-synonymous polymorphisms in effectors could either affect protein function directly through amino acid changes or give a non-functional or truncated protein. An in depth analysis of effector coding sequences led us to hypothesize for seven effectors, a process equivalent to Programmed Ribosomal Frameshifting (PRF) or Programmed Transcriptional Realignment (PTR) described in bacteria and viruses [43–44]. Indeed, for 5 CRN and 2 RXLR, PRF or PTR analogous processes should be required to get a full length coding frame matching exactly to the cDNA sequence, obtained independently from transcriptome sequencing (S1 Table). Canonical PRF patterns (A_AA.A_AA.C, underscores separate codons in the initial frame while dots separate codons in the new frame [44]) were indeed found in PhCRN07.3 and PhCRN10.2 *Pl*. *halstedii* effectors (S1 Table, Plasmopara halstedii effector polymorphism). While interesting, these hypotheses need to be confirmed by sequence determination of corresponding proteins, such experiments being very difficult to perform on an obligate biotroph such as *Pl*. *halstedii*. If confirmed, it will be to our knowledge, the first description of potential PRF in oomycetes, raising the following questions: Are eucaryotic oomycetes sharing such processes with procaryotes? If polymorphisms are detected in the presumed PRF site, as for the PhCRN10.2 effector, does it impair the protein encoded and does it impact the expression of corresponding genes in pathotypes?

### Genotyping of multi-isolate pathotypes highlighted a complex and dynamic pool of *Pl*. *halstedii* effectors

Assuming that effectors play a crucial role in the virulence of a given pathotype, we may have expected to find low intra-pathotype polymorphism for a large proportion of variable effectors. However, many putative effector genes were found to exhibit intra-pathotype polymorphisms, suggesting that not all the polymorphic effectors contributed equally to the pathogenicity profile of *Pl*. *halstedii*. Based on the phenotypic pathogenicity profile available so far, we hypothesize that (i) the monomorphic marker in a given pathotype may play a particular role in virulence specificity and (ii) the contribution of intra-pathotype polymorphic markers would constitute a basis for further virulence evolution. We identified at least 3 effectors showing a strong association between marker polymorphisms and resistance/susceptibility of sunflower differential lines, and therefore potential key effectors of *Pl*. *halstedii* virulence.

In addition, we observed a link between the emergence date of *Pl*. *halstedii* pathotypes and their genotypic variability. Indeed, *Pl*. *halstedii* pathotypes 100/710/703 first described in France (between 1966 and 1989 [2]) present no genotypic variation while pathotypes 700/314/714 that appeared more recently (between 1995 and 2002) were found to be variable. As previously shown in gene spare regions of *P*. *infestans* genome containing effector genes [16, 29], the genotypic variation in the recent pathotypes could be due to a higher genome plasticity (due to activation of transposable elements and recombination), which in turn may have produced new effector alleles. Associated with an increase in fitness of the pathotype isolate, these new effector alleles would have been selected rapidly, especially in the presence of a strong selective pressure exerted by sunflower *Pl* resistance genes.

### Towards designing a pathotype diagnosis marker set for *Pl*. *halstedii*

An applied objective was to propose a combination of markers designed on effector genes, to identify through genotyping the pathotype of any *Pl*. *halstedii* isolate sampled in the field. Ten *Pl*. *halstedii* pathotypes out of a total of 14 could be distinguished with 8 markers. In order to reinforce the determination key, the level of intra-pathotype variability should be estimated for the 6 pathotypes presenting only one isolate. Two groups of pathotypes (100 versus 304 and 314 versus 714) could not be distinguished one from the other with the set of KASP markers used. Therefore, differentiation of these grouped pathotypes still requires phenotyping tests. Yet, pathotype 100 became undetectable in France [2]. The high level of intra-pathotype heterogeneity in pathotypes 314 and 714 may have impeded their classification in the identification key.

Still, this original study highlights that molecular markers based on effector genes and organized in multilevel keys could be used to discriminate pathotypes recently emerged in crop populations where housekeeping genes failed. Most importantly, this approach could ultimately lead to the identification of major pathogenicity actors in sunflower downy mildew. *Pl*. *halstedii* pathotype virulence towards a sunflower genotype is probably caused by a complex combination of pathogen effectors, either conserved or polymorphic. Conserved effectors could be involved in common and important virulence functions that are probably linked to host specialization, whereas highly dynamic polymorphic effectors could play a role in breaking down sunflower resistance and be major actors of *Pl*. *halstedii* virulence evolution.

## Materials and Methods

### Pathotypes of *Plasmopara halstedii* isolates

For each of the 14 *Pl*. *halstedii* pathotypes, one reference isolate was collected and maintained by INRA Clermont-Ferrand (UMR1095 INRA-Université Blaise Pascal, France). In addition to these 14 reference isolates, 21 geographical isolates were collected from sunflower fields in different locations in France by INRA Clermont-Ferrand (Table 1), and were considered to be homogeneous in this study. DNA of all *Pl*. *halstedii* isolates was extracted with DNeasy Plant Mini Kits (Quiagen) from isolated spores collected from a susceptible sunflower line called GB [9]. Pathotypes of *Pl*. *halstedii* isolates were determined following [45], using the international nomenclature based on three digits (S1 Fig) [4].

### Identification of expressed *Pl*. *halstedii* putative RXLR and CRN effectors and their corresponding genomic sequences in seven pathotypes

The adopted strategy to find putative effectors is summarized in Fig 1. A PSI-tBLASTn analysis [46] was performed starting from transcriptomic data, in order to work on genes expressed during the interaction and to avoid pseudogenes that could have been selected from genomic data. PSI-tBLASTn search was realized on cDNA databases including *Pl*. *halstedii* and *H*. *annuus* sequences using an annotated oomycete effector gene database generated from Genbank [32]. cDNA consensus sequences present in these databases and named Plhalxxxxxx resulted from clustering of ESTs originating from inoculated sunflower and *Pl*. *halstedii* germinating spores of the four reference isolates for pathotypes 100, 304, 703, and 710 [32,47].

In order (i) to confirm that selected cDNA sequences were from pathogen and not from plant and (ii) to obtain genomic sequences from pathotypes, BLASTn searches were done from cDNA on draft genomic sequences obtained from *Pl*. *halstedii* sporangia and spores DNA of seven *Pl*. *halstedii* reference isolates (100, 304, 334, 700, 703, 710, 730), and selected if a minimum of six out of seven corresponding genomic sequences were found (except for the PhRXLR41 effector gene present only in 3 pathotypes). The 7 pathotypes were chosen to be representative of the species variability at pathogenic (S1 Fig) and phylogenetic levels [9]. Draft genomic sequences were obtained for the 7 pathotypes as described in [47]. Genomic sequences of the 54 *Pl*.*halstedii* effector genes in the 7 pathotypes are provided in S1 Table and in S1 File.

Candidate effector genes were selected when their predicted protein sequences fulfilled at least two of the three following criteria: (i) presence of conserved translocation domains, (ii) detection of a signal peptide, as checked with SignalP 3.0 [48] and (iii) a size between 50 and 300 amino acids for RXLR and more than 50 amino acids for CRN., MultAlin alignments [49] of nucleic acid coding sequences (NA) and corresponding predicted amino acid (AA) sequences of seven *Pl*. *halstedii* pathotypes were performed for each of the 54 effectors (S1 Table, Plasmopara halstedii effector polymorphism).

### Comparison of levels of polymorphism in *Pl*. *halstedii* putative effectors and non-effector genes

All polymorphism studies were based on gene coding sequences (CDS) and their corresponding predicted peptides. To build a subset of *Pl*. *halstedii* non-effector genes, we screened the EST database [32] for the following four criteria: (i) the cDNA clusters contained reads from the libraries of germinated spores from the pathotypes 100, 304, 703, 710, (ii) they had an INTERPRO annotation, (iii) their hit definition contained the word “Phytophthora” and (iv) cluster length was between 300 and 2300 bp. A total of 2933 EST clusters were obtained, from which 150 were randomly chosen. Out of theses EST clusters, only 125 alignments were obtained when blasted against the seven representative genomic sequences. We checked that no EST in this subset was annotated as effector. The same procedure was used to identify NA and AA polymorphisms between the genomic sequences of a minimum of six out of the seven pathotypes. The nucleotide diversity π [50] was calculated for each sequence in the two different samples (effectors and non effectors) using DNAsp v5.0 software (Fig 2A). Moreover, in order to depict by another way the occurrence of the polymorphism events within the sequence, we built a “SNP sparseness index” (Fig 2B and 2C) as follows: we divided the maximum length of the alignment by the number of SNPs or amino acid changes plus one. The square-root transformation applied to these raw data was found able to stabilize the variance within the two samples (effector and non-effector genes), thus allowing statistical means comparisons between effector and non-effector sets. We compared the distributions using a Kolmogorov-Smirnoff test in R (ks.test) and the means using a t-test.

### Genotyping with the Competitive Allele-Specific PCR (KASP)

Primers for KASP markers (S2 Table) were designed with BatchPrimer3 V1.0 to choose allele-specific primers and allele-flanking primers (http://probes.pw.usda.gov/batchprimer3/).

Amplification reactions were done with 3μl of KASP V4.0 2X Master mix (LGC Genomics, Herts, UK), 1μl ultrapure water, 2μl DNA (1ng/μl for DNA of isolates and 0.5ng/μl for DNA of reference isolates), and 0.07μl of primers mix (12μM each of allele-specific primer, carrying standard FAM or VIC compatible tails, and 32μM of allele-flanking primer).

Amplifications were carried out on GeneAmp^®^ PCR System 9700 (Applied BioSystems). PCR amplification programs began with pre-denaturation at 94°C for 15 min, followed by 11 cycles of denaturation at 94°C for 20s, annealing using touchdown at 65°C to 57°C for program A and 62°C to 54°C for program B, losing 0.8°C per cycle in both programs, elongation at 72°C for 45s. 25 cycles of denaturation at 94°C for 20s and annealing at 50°C for 30s were added.

Endpoint fluorescence was read using a 7900HT Fast Real-Time PCR System (Applied BioSystems), monitored by SDS 2.3 software with allelic discrimination under ROX conditions. Genotyping results were validated by at least two amplification runs for each marker.

## Supporting Information

S1 Fig (A) International nomenclature of *Pl*. *halstedii* pathotypes based on the virulence profile of a given isolate on 9 differential sunflower lines (D1-D9) selected according to their resistance patterns [3]. Resistance (R) and susceptibility (S) are defined by the absence or presence of disease symptoms and sporulation on leaves 2–3 weeks after inoculation of sunflower seedling roots, grown in soil [5]. A triplet coding system was set up on nine sunflower lines [3]. The phenotyping results on each triplet of sunflower differential lines give the pathotype digit values. If the first differential line of a set of three is susceptible, a value of ‘1’ is assigned to the pathotype. If the second line is S, a value of ‘2’, and, for the third line, a value of ‘4’. When the line is resistant, a value of ‘0’ is assigned to the pathotype. The virulence code is additive within each set. For example, virulence code 710 is explained by ‘7’ (S for D1–D3, 1 + 2 + 4 = 7), ‘1’ (S for D4) and ‘0’ (R for D7–D9). (B) Correlation between genetic distance and phenotypic distance based on nine differential *H*. *annuus* lines. Phenotypic distances between the differential *H*. *annuus* lines were computed on their virulence profile (presence/absence of symptoms), using the simple matching coefficient [51]. Genetic distances between the differential *H*. *annuus* lines were also computed using the simple matching coefficient, based on 21 KASP markers developped on effector genes (PhCRN02_1 was excluded due to high heterozygosity); for the remaining KASP markers, heterozygous genotypes (<5%) at a given KASP marker were replaced by the most common allele. Mantel analysis was performed with the mantel function of ecodist R package, using 21 KASP markers and 13 pathotypes (pathotype 714 was excluded due to its heterogeneity). The relationship between the phenotypic and genetic distance matrices was estimated by the Spearman correlation coefficient according to the Mantel test (10 000 permutations).
(TIF)

S1 File Genomic sequences of the 54 *Pl*.*halstedii* effector genes in 7 pathotypes. The pathotype name is indicated in the effector sequence name after the underscore sign (for example: PhCRN01_100).
(PDF)

S1 Table Description of the 54 putative effectors identified in *Pl*. *halstedii* and their polymorphisms. The table lists EST names of effectors and their corresponding PSI-tBLASTn best values against an oomycete effector gene database generated from Genebank, the effector names and their conserved motifs, and eventual signal peptide predictions. For each effector candidate, a multifasta file combined with an alignment of the effector genomic sequences in seven pathotypes (PLHAL”pathotype_name”xxxx) and the EST used as query (Plhalyyyyyy), are provided in column “NA multifasta file”. Corresponding translated sequences and alignments are also provided (AA multifasta file and AA multifasta alignment). Hypothetical Programmed Ribosomal Frameshifting (PRF) processes are indicated (see Discussion). The nucleic sequences showing identified SNPs are listed (see also S1 File) and indicated as KASP markers when used.
(TIF)

S2 Table Effector polymorphisms, PCR programs, and primers used for KASP markers. The position of the polymorphism relative to the start of the CDS is indicated in parentheses. Sequences of specific primers are respectively added at 5’ end by FAM tag (GAAGGTGACCAAGTTCATGCT) or VIC tag (GAAGGTCGGAGTCAACGGATT).
(TIF)
