# Supplementary figures and images for: Effector Polymorphisms of the Sunflower Downy Mildew Pathogen Plasmopara halstedii and Their Use to Identify Pathotypes from Field Isolates
Source: PLoS One. 2016 Feb 4;11(2):e0148513. doi: 10.1371/journal.pone.0148513 (PMC4742249; doi:10.1371/journal.pone.0148513)

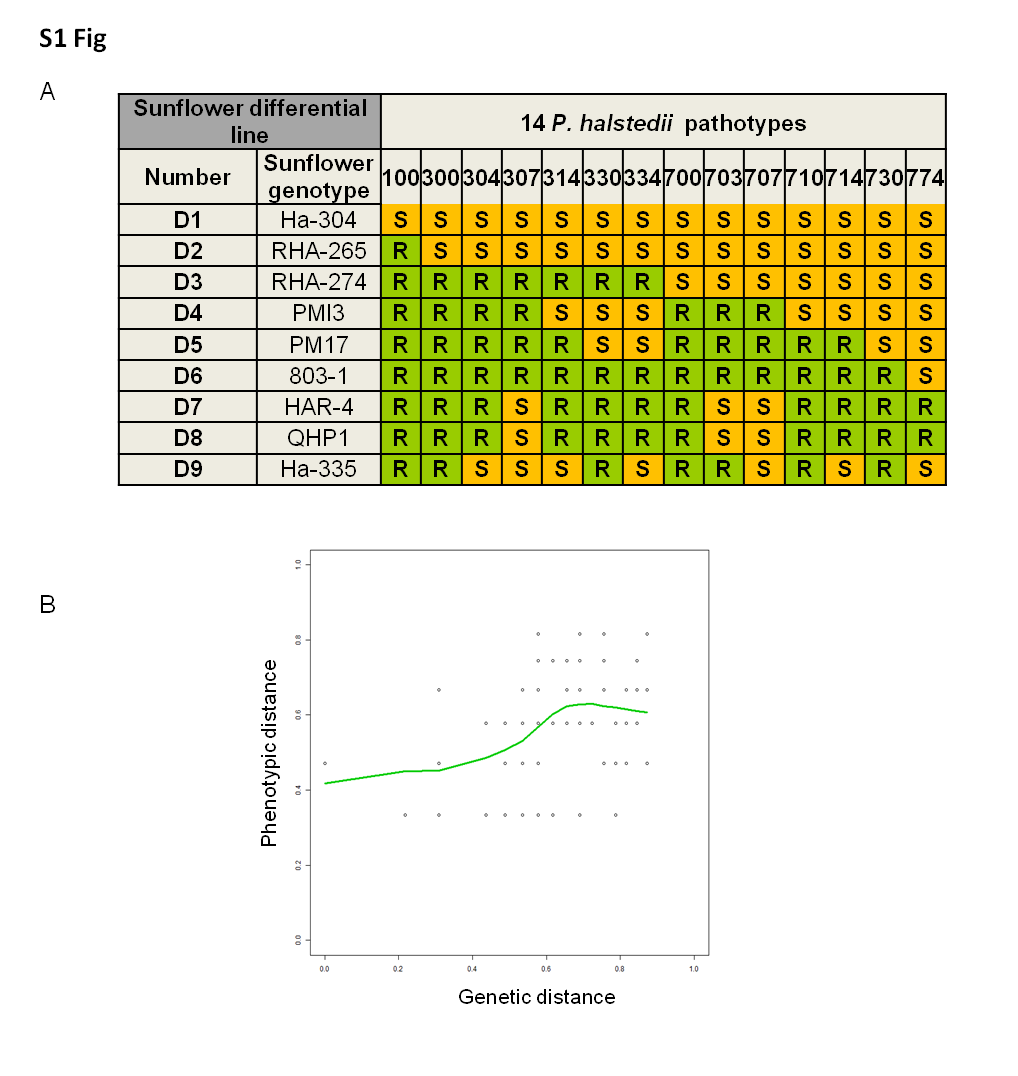

Supplement: S1 Fig — (A) International nomenclature of Pl. halstedii pathotypes based on the virulence profile of a given isolate on 9 differential sunflower lines (D1-D9) selected according to their resistance patterns [3]. Resistance (R) and susceptibility (S) are defined by the absence or presence of disease symptoms and sporulation on leaves 2–3 weeks after inoculation of sunflower seedling roots, grown in soil [5]. A triplet coding system was set up on nine sunflower lines [3]. The phenotyping results on each triplet of sunflower differential lines give the pathotype digit values. If the first differential line of a set of three is susceptible, a value of ‘1’ is assigned to the pathotype. If the second line is S, a value of ‘2’, and, for the third line, a value of ‘4’. When the line is resistant, a value of ‘0’ is assigned to the pathotype. The virulence code is additive within each set. For example, virulence code 710 is explained by ‘7’ (S for D1–D3, 1 + 2 + 4 = 7), ‘1’ (S for D4) and ‘0’ (R for D7–D9). (B) Correlation between genetic distance and phenotypic distance based on nine differential H. annuus lines. Phenotypic distances between the differential H. annuus lines were computed on their virulence profile (presence/absence of symptoms), using the simple matching coefficient [51]. Genetic distances between the differential H. annuus lines were also computed using the simple matching coefficient, based on 21 KASP markers developped on effector genes (PhCRN02_1 was excluded due to high heterozygosity); for the remaining KASP markers, heterozygous genotypes (<5%) at a given KASP marker were replaced by the most common allele. Mantel analysis was performed with the mantel function of ecodist R package, using 21 KASP markers and 13 pathotypes (pathotype 714 was excluded due to its heterogeneity). The relationship between the phenotypic and genetic distance matrices was estimated by the Spearman correlation coefficient according to the Mantel test (10 000 permutations). (TIF) [file pone.0148513.s001.tif]

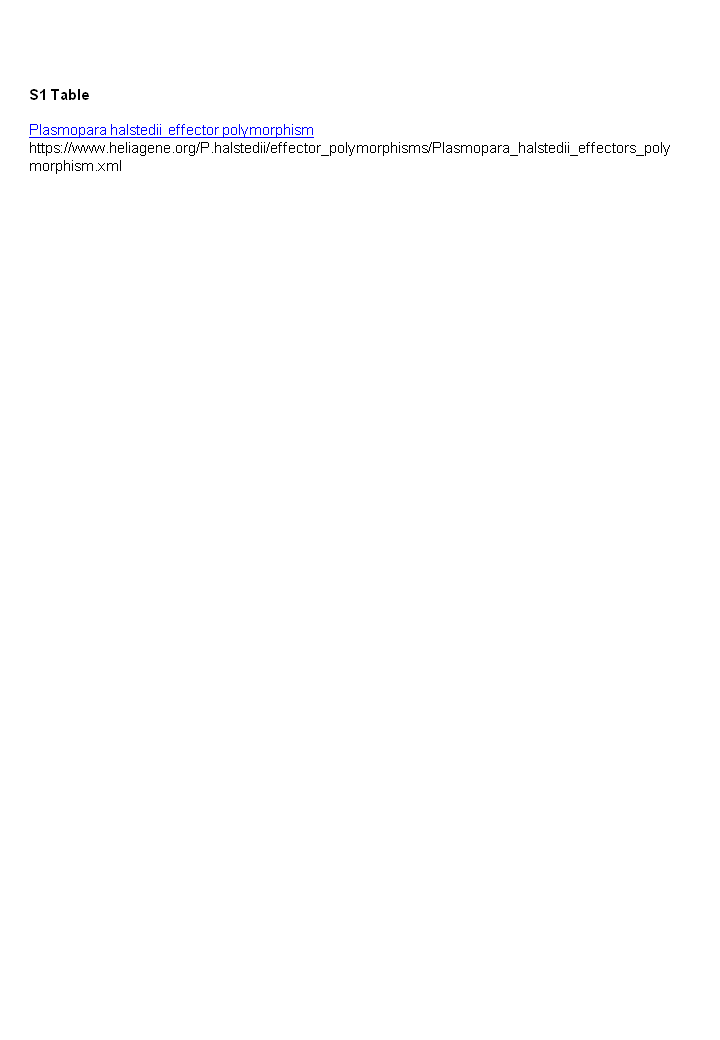

Supplement: S1 Table — The table lists EST names of effectors and their corresponding PSI-tBLASTn best values against an oomycete effector gene database generated from Genebank, the effector names and their conserved motifs, and eventual signal peptide predictions. For each effector candidate, a multifasta file combined with an alignment of the effector genomic sequences in seven pathotypes (PLHAL”pathotype_name”xxxx) and the EST used as query (Plhalyyyyyy), are provided in column “NA multifasta file”. Corresponding translated sequences and alignments are also provided (AA multifasta file and AA multifasta alignment). Hypothetical Programmed Ribosomal Frameshifting (PRF) processes are indicated (see Discussion). The nucleic sequences showing identified SNPs are listed (see also S1 File) and indicated as KASP markers when used. (TIF) [file pone.0148513.s003.tif]

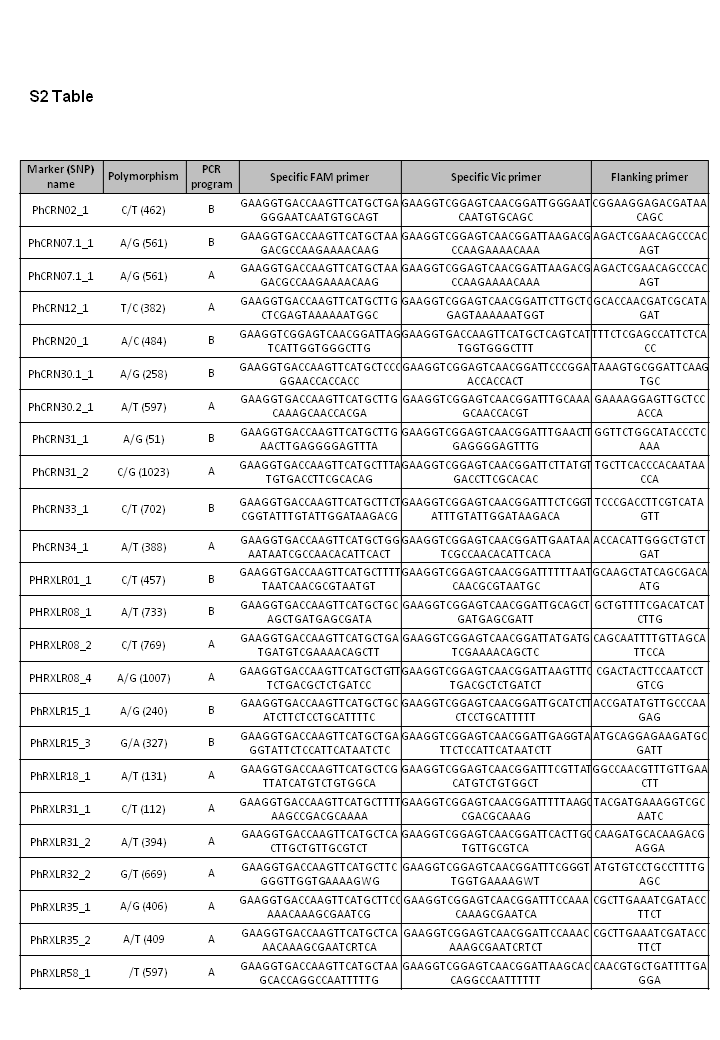

Supplement: S2 Table — The position of the polymorphism relative to the start of the CDS is indicated in parentheses. Sequences of specific primers are respectively added at 5’ end by FAM tag (GAAGGTGACCAAGTTCATGCT) or VIC tag (GAAGGTCGGAGTCAACGGATT). (TIF) [file pone.0148513.s004.tif]
