# Supplementary material for: Effector Polymorphisms of the Sunflower Downy Mildew Pathogen Plasmopara halstedii and Their Use to Identify Pathotypes from Field Isolates
Source: PLoS One. 2016 Feb 4;11(2):e0148513. doi: 10.1371/journal.pone.0148513 (PMC4742249; doi:10.1371/journal.pone.0148513)
Supplement: S1 File — The pathotype name is indicated in the effector sequence name after the underscore sign (for example: PhCRN01_100). (PDF) [file pone.0148513.s002.pdf]

>PhCRN01_100 PLHAL100A12922
GGGGTCAGCAGAACGGAAGTGTGTGACATGATACGGGACGGACGAGGCATTCGGAAGTTCAACGAACCACTTGGGGATTCCGAGTTG
GAATATGAAGGTGGAGTTCCTGCAACATTCAAGACGTTGCGAAAAGCCACTGTGGCCGCATTTGCAAGTCTTCTCGATCGCATACCA
GTGATATTCATCCGCGCTCCCCCTTTGTCCGGAAAGACGGCCTTGTGCGATCTACTCTACGACCACATTGTTCGCTCCAAGCCTGAT
GCACTTGTTTCCCGCATTCTGGCCAATCAAATGCCTGTAGATGGCAAGTTTACCGAGTTTTTCAAATTTTTGTACGGCTATGAGTTT
GAAGCATTCTGCGCACACAAAGGCGATCGGGTGGTACTGATTGATGGAGCTCAATTTACGTATAACGACGAGCAGCTGTGGAGGGGA
TACGTGAAAGGCGCATTGGAATCGCAAATACGAGGCCTTCGCTTCGTTCTCTTTTCATCGTATGGCAGCTTTAATATTTACCGCAAA
TTTGAACGCGCTGGAACTCCGATCCTTGTGCCTCCTCAGAATACTTTTGGATTGAATGCCACACCATACAAGCCTGGCCTGCAGCTT
TCACGTGAGGAGCTGAAAGAAATGGCTCAAAACAGCATTGGCATATCAGCCTCCGATCTCATTTGGATCTTGTGCTCGGGTCATATC
GGAATTGCTCGAGCTATCCTCATCTTTTTGCATCGTAAGTTTGGTTCTGGAAAGCCAAACGATGAAGATGTTGAGCTGGAACTTCGC
TCCTTGGGGCTGCTGCAAGAAATTCGCTCCAGTTATCGGGGTATCCCAAGTGCTGATGCTTTCGAGCGAGTGAAAGATGCTAATAAC
CTGCCGGATGAAGCAGTGCTGAAGATGATTGGAATCTTGAATGGTGTTGCTTCTGGCAAGGTTATGCTTGTATCAGATGGACATCGA
ACTCCTAGAAGCCAGTTTGCTGCGGAATTGTTGACAAGGTACGGGTTCTTGTACGAGGATCAGGCGGGGCAGCTTCAGTTCGCTTCA
AGCATGCACCTCAAGATCTGGCTGTATTCAAATCGAACAGATCCAATTTCGTACATGGTATCAGACGTCGCACACGAAGACTTTGTT
GTCGCGTGCGTACAGCGAATGAGTGCTTTGCGACTGCAGAACTTCGCGAACGAAAACACCAACGGCATTGCTCGAGAACGCCAAATA
CAGATGGAGCTATACGGCGCCACAGTATCATGCTTGCCTCGAGATGTGTTGGTTACCCCTGAATGGCGAACGACTGATGGCAAAGGA
TACATTGATTTAATGATTCGTGGCTCGAGCATTCTTTGGTTTTGGGAGTTGTTAGTGAATGGTGACGATGCTGTCGGCCACTCAAAT
CGATTTGAAACTGGGGGGAAGTACCATGGAAGCTTAACTGGAAGTTCTAAGTATGTGCTCATCGATTTTCGTCAGAGTAAGGGCGTT
CGGCACCAGAAGCATGGGTTTCTGTATGTGTCGTTTGTGGATTCTTACACCAAAGCGCTGGTTTTCAGCACTGGTAAGTCCGTTGTA
AGCGTCAAACTGCTAAGCTAATTAACTGTTCATGTTTAATACATAAGGTTTT
>PhCRN01_334 PLHAL3342991
GAACGGAAGTGTGTGACATGATACGGGACGGACGAGGCATTCGGAAGTTCAACGAACCACTTGGGGATTCCGAGTTGGAATATGAAG
GTGGAGTTCCTGCAACATTCAAGACGTTGCGAAAAGCCACTGTGGCCGCATTTGCAAGTCTTCTCGATCGCATACCAGTGATATTCA
TCCGCGCTCCCCCTTTGTCCGGAAAGACGGCCTTGTGCGATCTACTCTACGACCACATTGTTCGCTCCAAGCCTGATGCACTTGTTT
CCCGCATTCTGGCCAATCAAATGCCTGTAGATGGCAAGTTTACCGAGTTTTTCAAATTTTTGTACGGCTATGAGTTTGAAGCATTCT
GCGCACACAAAGGCGATCGGGTGGTACTGATTGATGAAGCTCAAATTACGTATAACGACGAGCAGCTGTGGAGGGGATACGTGAAAG
GCGCATTGGAATCGCAAATACGAGGCCTTCGCTTCGTTCTCTTTTCATCGTATGGCAGCTTTAATATTTACCGCAAATTTGAACGCG
CTGGAACTCCGATCCTTGTGCCTCCTCAGAATACTTTTGGATTGAATGCCACACCATACAAGCCTGGCCTGCAGCTTTCACGTGAGG
AGCTGAAAGAAATGGCTCAAAACAGCATTGGCATATCAGCCTCCGATCTCATTTGGATCTTGTGCTCGGGTCATATCGGAATTGCTC
GAGCTATCCTCATCTTTTTGCATCGTAAGTTTGGTTCTGGAAAGCCAAACGATGAAGATGTTGAGCTGGAACTTCGCTCCTTGGGGC
TGCTGCAAGAAATTCGCTCCAGTTATCGGGGTATCCCAAGTGCTGATGCTTTCGAGCGAGTGAAAGATGCTAATAACCTGCCGGATG
AAGCAGTGCTGAAGATGATTGGAATCTTGAATGGTGTTGCTTCTGGCAAGGTTATGCTTGTATCAGATGGACATCGAACTCCTAGAA
GCCAGTTTGCTGCGGAATTGTTGACAAGGTACGGGTTCTTGTACGAGGATCAGGCGGGGCAGCTTCAGTTCGCTTCAAGCATGCACC
TCAAGATCTGGCTGTATTCAAATCGAACAGATCCAATTTCGTACATGGTATCAGACGTCGCACACGAAGACTTTGTTGTCGCGTGCG
TACAGCGAATGAGTGCTTTGCGACTGCAGAACTTCGCGAACGAAAACACCAACGGCATTGCTCGAGAACGCCAAATACAGATGGAGC
TATACGGCGCCACAGTATCATGCTTGCCTCGAGATGTGTTGGTTACCCCTGAATGGCGAACGACTGATGGCAAAGGATACATTGATT
TAATGATTCGTGGCTCGAGCATTCTTTGGTTTTGGGAGTTGTTAGTGAATGGTGACGATGCTGTCGGCCACTCAAATCGATTTGAAA
CTGGGGGGAAGTACCATGGAAGCTTAACTGGAAGTTCTAAGTATGTGCTCATCGATTTTCGTCAGAGTAAGGGCGTTCGGCACCAGA
AGCATGGGTTTCTGTATGTGTCGTTTGTGGATTCTTACACCAAAGCGCTGGTTTTCAGCACTGGTAAGTCCGTTGTAAGCGTCAAAC
TGCTAAGCTAATTAACTGTTCACGTTTAATACATAAGGTTTT
>PhCRN01_703 PLHAL7032745
GAACGGAAGTGTGTGACATGATACGGGACGGACGAGGCATTCGGAAGTTCAACGAACCACTTGGGGATTCCGAGTTGGAATATGAAG
GTGGAGTTCCTGCAACATTCAAGACGTTGCGAAAAGCCACTGTGGCCGCATTTGCAAGTCTTCTCGATCGCATACCAGTGATATTCA
TCCGCGCTCCCCCTTTGTCCGGAAAGACGGCCTTGTGCGATCTACTCTACGACCACATTGTTCGCTCCAAGCCTGATGCACTTGTTT
CCCGCATTCTGGCCAATCAAATGCCTGTAGATGGCAAGTTTACCGAGTTTTTCAAATTTTTGTACGGCTATGAGTTTGAAGCATTCT
GCGCACACAAAGGCGATCGGGTGGTACTGATTGATGAAGCTCAAATTACGTATAACGACGAGCAGCTGTGGAGGGGATACGTGAAAG
GCGCATTGGAATCGCAAATACGAGGCCTTCGCTTCGTTCTCTTTTCATCGTATGGCAGCTTTAATATTTACCGCAAATTTGAACGCG
CTGGAACTCCGATCCTTGTGCCTCCTCAGAATACTTTTGGATTGAATGCCACACCATACAAGCCTGGCCTGCAGCTTTCACGTGAGG
AGCTGAAAGAAATGGCTCAAAACAGCATTGGCATATCAGCCTCCGATCTCATTTGGATCTTGTGCTCGGGTCATATCGGAATTGCTC
GAGCTATCCTCATCTTTTTGCATCGTAAGTTTGGTTCTGGAAAGCCAAACGATGAAGATGTTGAGCTGGAACTTCGCTCCTTGGGGC
TGCTGCAAGAAATTCGCTCCAGTTATCGGGGTATCCCAAGTGCTGATGCTTTCGAGCGAGTGAAAGATGCTAATAACCTGCCGGATG
AAGCAGTGCTGAAGATGATTGGAATCTTGAATGGTGTTGCTTCTGGCAAGGTTATGCTTGTATCAGATGGACATCGAACTCCTAGAA
GCCAGTTTGCTGCGGAATTGTTGACAAGGTACGGGTTCTTGTACGAGGATCAGGCGGGGCAGCTTCAGTTCGCTTCAAGCATGCACC
TCAAGATCTGGCTGTATTCAAATCGAACAGATCCAATTTCGTACATGGTATCAGACGTCGCACACGAAGACTTTGTTGTCGCGTGCG
TACAGCGAATGAGTGCTTTGCGACTGCAGAACTTCGCGAACGAAAACACCAACGGCATTGCTCGAGAACGCCAAATACAGATGGAGC
TATACGGCGCCACAGTATCATGCTTGCCTCGAGATGTGTTGGTTACCCCTGAATGGCGAACGACTGATGGCAAAGGATACATTGATT
TAATGATTCGTGGCTCGAGCATTCTTTGGTTTTGGGAGTTGTTAGTGAATGGTGACGATGCTGTCGGCCACTCAAATCGATTTGAAA
CTGGGGGGAAGTACCATGGAAGCTTAACTGGAAGTTCTAAGTATGTGCTCATCGATTTTCGTCAGAGTAAGGGCGTTCGGCACCAGA
AGCATGGGTTTCTGTATGTGTCGTTTGTGGATTCTTACACCAAAGCGCTGGTTTTCAGCACTGGT
>PhCRN01_710
GGGGTCAGCAGAACGGAAGTGTGTGACATGATACGGGACGGACGAGGCATTCGGAAGTTCAACGAACCACTTGGGGATTCCGAGTTG
GAATATGAAGGTGGAGTTCCTGCAACATTCAAGACGTTGCGAAAAGCCACTGTGGCCGCATTTGCAAGTCTTCTCGATCGCATACCA
GTGATATTCATCCGCGCTCCCCCTTTGTCCGGAAAGACGGCCTTGTGCGATCTACTCTACGACCACATTGTTCGCTCCAAGCCTGAT
GCACTTGTTTCCCGCATTCTGGCCAATCAAATGCCTGTAGATGGCAAGTTTACCGAGTTTTTCAAATTTTTGTACGGCTATGAGTTT
GAAGCATTCTGCGCACACAAAGGCGATCGGGTGGTACTGATTGATGGAGCTCAATTTACGTATAACGACGAGCAGCTGTGGAGGGGA
TACGTGAAAGGCGCATTGGAATCGCAAATACGAGGCCTTCGCTTCGTTCTCTTTTCATCGTATGGCAGCTTTAATATTTACCGCAAA
TTTGAACGCGCTGGAACTCCGATCCTTGTGCCTCCTCAGAATACTTTTGGATTGAATGCCACACCATACAAGCCTGGCCTGCAGCTT

TCACGTGAGGAGCTGAAAGAAATGGCTCAAAACAGCATTGGCATATCAGCCTCCGATCTCATTTGGATCTTGTGCTCGGGTCATATC
GGAATTGCTCGAGCTATCCTCATCTTTTTGCATCGTAAGTTTGGTTCTGGAAAGCCAAACGATGAAGATGTTGAGCTGGAACTTCGC
TCCTTGGGGCTGCTGCAAGAAATTCGCTCCAGTTATCGGGGTATCCCAAGTGCTGATGCTTTCGAGCGAGTGAAAGATGCTAATAAC
CTGCCGGATGAAGCAGTGCTGAAGATGATTGGAATCTTGAATGGTGTTGCTTCTGGCAAGGTTATGCTTGTATCAGATGGACATCGA
ACTCCTAGAAGCCAGTTTGCTGCGGAATTGTTGACAAGGTACGGGTTCTTGTACGAGGATCAGGCGGGGCAGCTTCAGTTCGCTTCA
AGCATGCACCTCAAGATCTGGCTGTATTCAAATCGAACAGATCCAATTTCGTACATGGTATCAGACGTCGCACACGAAGACTTTGTT
GTCGCGTGCGTACAGCGAATGAGTGCTTTGCGACTGCAGAACTTCGCGAACGAAAACACCAACGGCATTGCTCGAGAACGCCAAATA
CAGATGGAGCTATACGGCGCCACAGTATCATGCTTGCCTCGAGATGTGTTGGTTACCCCTGAATGGCGAACGACTGATGGCAAAGGA
TACATTGATTTAATGATTCGTGGCTCGAGCATTCTTTGGTTTTGGGAGTTGTTAGTGAATGGTGACGATGCTGTCGGCCACTCAAAT
CGATTTGAAACTGGGGGGAAGTACCATGGAAGCTTAACTGGAAGTTCTAAGTATGTGCTCATCGATTTTCGTCAGAGTAAGGGCGTT
CGGCACCAGAAGCATGGGTTTCTGTATGTGTCGTTTGTGGATTCTTACACCAAAGCGCTGGTTTTCAGCACTGGTAAGTCCGTTGTA
AGCGTCAAACTGCTAAGCTAATTAACTGTTCACGTTTAATACATAAGGTTTT
>PhCRN01_304 PLHAL3043337
GGGATACGTGAAAGGCGCATTGGAATCGCAAATACGAGGCCTTCGCTTCGTTCTCTTTTCATCGTATGGCAGCTTTAATATTTACCG
CAAATTTGAACGCGCTGGAACTCCGATCCTTGTGCCTCCTCAGAATACTTTTGGATTGAATGCCACACCATACAAGCCTGGCCTGCA
GCTTTCACGTGAGGAGCTGAAAGAAATGGCTCAAAACAGCATTGGCATATCAGCCTCCGATCTCATTTGGATCTTGTGCTCGGGTCA
TATCGGAATTGCTCGAGCTATCCTCATCTTTTTGCATCGTAAGTTTGGTTCTGGAAAGCCAAACGATGAAGATGTTGAGCTGGAACT
TCGCTCCTTGGGGCTGCTGCAAGAAATTCGCTCCAGTTATCGGGGTATCCCAAGTGCTGATGCTTTCGAGCGAGTGAAAGATGCTAA
TAACCTGCCGGATGAAGCAGTGCTGAAGATGATTGGAATCTTGAATGGTGTTGCTTCTGGCAAGGTTATGCTTGTATCAGATGGACA
TCGAACTCCTAGAAGCCAGTTTGCTGCGGAATTGTTGACAAGGTACGGGTTCTTGTACGAGGATCAGGCGGGGCAGCTTCAGTTCGC
TTCAAGCATGCACCTCAAGATCTGGCTGTATTCAAATCGAACAGATCCAATTTCGTACATGGTATCAGACGTCGCACACGAAGACTT
TGTTGTCGCGTGCGTACAGCGAATGAGTGCTTTGCGACTGCAGAACTTCGCGAACGAAAACACCAACGGCATTGCTCGAGAACGCCA
AATACAGATGGAGCTATACGGCGCCACAGTATCATGCTTGCCTCGAGATGTGTTGGTTACCCCTGAATGGCGAACGACTGATGGCAA
AGGATACATTGATTTAATGATTCGTGGCTCGAGCATTCTTTGGTTTTGGGAGTTGTTAGTGAATGGTGACGATGCTGTCGGCCACTC
AAATCGATTTGAAACTGGGGGGAAGTACCATGGAAGCTTAACTGGAAGTTCTAAGTATGTGCTCATCGATTTTCGTCAGAGTAAGGG
CGTTCGGCACCAGAAGCATGGGTTTCTGTATGTGTCGTTTGTGGATTCTTACACCAAAGCGCTGGTTTTCAGCACTGGTAAGTCCGT
TGTAAGCGTCAAACTGCTAAGCTAATTAACTGTTCA
>PhCRN01_700 PLHAL7004275
AGCTCAAATTACGTATAACGACGAGCAGCTGTGGAGGGGATACGTGAAAGGCGCATTGGAATCGCAAATACGAGGCCTTCGCTTCGT
TCTCTTTTCATCGTATGGCAGCTTTAATATTTACCGCAAATTTGAACGCGCTGGAACTCCGATCCTTGTGCCTCCTCAGAATACTTT
TGGATTGAATGCCACACCATACAAGCCTGGCCTGCAGCTTTCACGTGAGGAGCTGAAAGAAATGGCTCAAAACAGCATTGGCATATC
AGCCTCCGATCTCATTTGGATCTTGTGCTCGGGTCATATCGGAATTGCTCGAGCTATCCTCATCTTTTTGCATCGTAAGTTTGGTTC
TGGAAAGCCAAACGATGAAGATGTTGAGCTGGAACTTCGCTCCTTGGGGCTGCTGCAAGAAATTCGCTCCAGTTATCGGGGTATCCC
AAGTGCTGATGCTTTCGAGCGAGTGAAAGATGCTAATAACCTGCCGGATGAAGCAGTGCTGAAGATGATTGGAATCTTGAATGGTGT
TGCTTCTGGCAAGGTTATGCTTGTATCAGATGGACATCGAACTCCTAGAAGCCAGTTTGCTGCGGAATTGTTGACAAGGTACGGGTT
CTTGTACGAGGATCAGGCGGGGCAGCTTCAGTTCGCTTCAAGCATGCACCTCAAGATCTGGCTGTATTCAAATCGAACAGATCCAAT
TTCGTACATGGTATCAGACGTCGCACACGAAGACTTTGTTGTCGCGTGCGTACAGCGAATGAGTGCTTTGCGACTGCAGAACTTCGC
GAACGAAAACACCAACGGCATTGCTCGAGAACGCCAAATACAGATGGAGCTATACGGCGCCACAGTATCATGCTTGCCTCGAGATGT
GTTGGTTACCCCTGAATGGCGAACGACTGATGGCAAAGGATACATTGATTTAATGATTCGTGGCTCGAGCATTCTTTGGTTTTGGGA
GTTGTTAGTGAATGGTGACGATGCTGTCGGCCACTCAAATCGATTTGAAACTGGGGGGAAGTACCATGGAAGCTTAACTGGAAGTTC
TAAGTATGTGCTCATCGATTTTCGTCAGAGTAAGGGCGTTCGG
>PhCRN01_730 PLHAL7303476
TTACGTATAACGACGAGCAGCTGTGGAGGGGATACGTGAAAGGCGCATTGGAATCGCAAATACGAGGCCTTCGCTTCGTTCTCTTTT
CATCGTATGGCAGCTTTAATATTTACCGCAAATTTGAACGCGCTGGAACTCCGATCCTTGTGCCTCCTCAGAATACTTTTGGATTGA
ATGCCACACCATACAAGCCTGGCCTGCAGCTTTCACGTGAGGAGCTGAAAGAAATGGCTCAAAACAGCATTGGCATATCAGCCTCCG
ATCTCATTTGGATCTTGTGCTCGGGTCATATCGGAATTGCTCGAGCTATCCTCATCTTTTTGCATCGTAAGTTTGGTTCTGGAAAGC
CAAACGATGAAGATGTTGAGCTGGAACTTCGCTCCTTGGGGCTGCTGCAAGAAATTCGCTCCAGTTATCGGGGTATCCCAAGTGCTG
ATGCTTTCGAGCGAGTGAAAGATGCTAATAACCTGCCGGATGAAGCAGTGCTGAAGATGATTGGAATCTTGAATGGTGTTGCTTCTG
GCAAGGTTATGCTTGTATCAGATGGACATCGAACTCCTAGAAGCCAGTTTGCTGCGGAATTGTTGACAAGGTACGGGTTCTTGTACG
AGGATCAGGCGGGGCAGCTTCAGTTCGCTTCAAGCATGCACCTCAAGATCTGGCTGTATTCAAATCGAACAGATCCAATTTCGTACA
TGGTATCAGACGTCGCACACGAAGACTTTGTTGTCGCGTGCGTACAGCGAATGAGTGCTTTGCGACTGCAGAACTTCGCGAACGAAA
ACACCAACGGCATTGCTCGAGAACGCCAAATACAGATGGAGCTATACGGCGCCACAGTATCATGCTTGCCTCGAGATGTGTTGGTTA
CCCCTGAATGGCGAACGACTGATGGCAAAGGATACATTGATTTAATGATTCGTGGCTCGAGCATTCTTTGGTTTTGGGAGTTGTTAG
TGAATGGTGACGATGCTGTCGGCCACTCAAATCGATTTGAAACTGGGGGGAAGTACCATGGAAGCTTAACTGGAAGTTCTAAGTATG
TGCTCATCGATTTTCGTCAGAGTAAGGGCGTTCGGCACCAGAAGCATGGGTTTCTGTA

>PhCRN02_100 PLHAL100A12728
GATGTGTTGGATATGAATGGAATGCCACCGCCTCAAACGCGGCAAATTCACGTGCTGGTGGTGGTTCCGTATCGGACCGAGGATGCC
AACCTTTCCCAGATTTTTCCGACCTTTCGACCATACTGTGAGGGAATCAATGTGCAGCACCCCTCATCTTTCGCGGGATGTGCTGGTT
GAAAAACTCTACGAAGCTATTATCCGAACCAATTTTGTGCTGTTATCGTCTCCTTCCGGTTCTGGAAAGACGTCGCTGTTGACATTG
TTTGCGCGTAAACATCCTGAAATTTCCTGCGCACCTATCGCTTTTGATGGTAGCACAGAAGATGCCACAACTCTTCTTTCCACTTAT
GGCGTCAATGTTTATAAGAAGGCGTGCGATATACCCAGTGGCAAGCTGTGTGTCTTGTTGTTGGACGACTGTCAACGGCGGTATAAT
GATCTCGTCTTTTGGACTCGTCTGATTAAAGGCTCAGCATCATGGTTACCAGATCACGTCCGATTTATCATCTCGGCGACTCATTTG
CTGGAGACGGATGCCCCTTCCAGCCCTGTTGCTTTCAGCAGCATCCAGTATAAGTTGACGAGGGACGATTTCTTGATTAATGATGAG
GAGGCGTATCAGTGCTTTAATTTGGAAAACGGCCTACCACCAGGATTGCGTTTTCCAACGCTGGTAGAGGTGATGATACGCGAGTGC
AATGGGCACATCGGTTCATTACGCATATCAATTGATGCAATTTATGAGCGTTTTCGGAGGGCGGGTGCTCGAACTGAGGAGGAGTTG
CTGGCGTTCTATCTGTCGGACTTTTGTCGAACAAATGGCAAGATGCTTCGGAAGTGAACACACAACACCTGCTAGTCCAAACCAGCA

GAAGTTTCTTAGCACGTGTTTGTTGTGCGATCGTCAGCCACTGGTACAGCAATTGAGTCGTGATGAACACGAATGTTTTACAAGGTT
GAAGAAAGCGGGTATTGTAACTGAAGTTGGCGGCT
>PhCRN02_334 PLHAL3343893
GATGTGTTGGATATGAATGGAATGCCACCGCCTCAAACGCGGCAAATTCACGTGCTGGTGGTGGTTCCGTATCGGACCGAGGATGCC
AACCTTTCCCAGATTTTTCCGACCTTTCGACCATACTGTGAGGGAATCAATGTGCAGTACCCTCATCTTTCGCGGGATGTGCTGGTT
GAAAAACTCTACGAAGCTATTATCCGAACCAATTTTGTGCTGTTATCGTCTCCTTCCGGTTCTGGAAAGACGTCGCTGTTGACATTG
TTTGCGCGTAAACATCCTGAAATTTCCTGCGCACCTATCGCTTTTGATGGTAGCACAGAAGATGCCACAACTCTTCTTTCCACTTAT
GGCGTCAATGTTTATAAGAAGGCGTGCGATATACCCAGTGGCAAGCTGTGTGTCTTGTTGTTGGACGACTGTCAACGGCGGTATAAT
GATCTCGTCTTTTGGACTCGTCTGATTAAAGGCTCAGCATCATGGTTACCAGATCACGTCCGATTTATCATCTCGGCGACTCATTTG
CTGGAGACGGATGCCCCTTCCAGCCCTGTTGCTTTCAGCAGCATCCAGTATAAGTTGACGAGGGACGATTTCTTGATTAATGATGAG
GAGGCGTATCAGTGCTTCAATTTGGAAAACGGCCTACCACCAGGATTGCGTTTTCCAACGCTGGTAGAGGTGATGATACGCGAGTGC
AATGGGCACATCGGTTCATTACGCATATCAATTGATGCAATTTATGAGCGTTTTCGGAGGGCGGGTGCTCGAACTGAGGAGGAGTTG
CTGGCGTTCTATCTGTCGGACTTTTGTCGAACAAATGGCAAGATGCTTCGGAAGTGAACACACAACACCTGCTAGTCCAAACCAGCA
GAAGTTTCTTAGCACGTGTTTGTTGTGCGATC
>PhCRN02_703 PLHAL7034223
CCTTTCGACCATACTGTGAGGGAATCAATGTGCAGCACCCTCATCTTTCGCGGGATGTGCTGGTTGAAAAACTCTACGAAGCTATTA
TCCGAACCAATTTTGTGCTGTTATCGTCTCCTTCCGGTTCTGGAAAGACGTCGCTGTTGACATTGTTTGCGCGTAAACATCCTGAAA
TTTCCTGCGCACCTATCGCTTTTGATGGTAGCACAGAAGATGCCACAACTCTTCTTTCCACTTATGGCGTCAATGTTTATAAGAAGG
CGTGCGATATACCCAGTGGCAAGCTGTGTGTCTTGTTGTTGGACGACTGTCAACGGCGGTATAATGATCTCGTCTTTTGGACTCGTC
TGATTAAAGGCTCAGCATCATGGTTACCAGATCACGTCCGATTTATCATCTCGGCGACTCATTTGCTGGAGACGGATGCCCCTTCCA
GCCCTGTTGCTTTCAGCAGCATCCAGTATAAGTTGACGAGGGACGATTTCTTGATTAATGATGAGGAGGCGTATCAGTGCTTTAATT
TGGAAAACGGCCTACCACCAGGATTGCGTTTTCCAACGCTGGTAGAGGTGATGATACGCGAGTGCAATGGGCACATCGGTTCATTAC
GCATATCAATTGATGCAATTTATGAGCGTTTTCGGAGGGCGGGTGCTCGAACTGAGGAGGAGTTGCTGGCGTTCTATCTGTCGG
>PhCRN02_710 PLHAL7104310
GATGTGTTGGATATGAATGGAATGCCACCGCCTCAAACGCGGCAAATTCACGTGCTGGTGGTGGTTCCGTATCGGACCGAGGATGCC
AACCTTTCCCAGATTTTTCCGACCTTTCGACCATACTGTGAGGGAATCAATGTGCAGCACCCTCATCTTTCGCGGGATGTGCTGGTT
GAAAAACTCTACGAAGCTATTATCCGAACCAATTTTGTGCTGTTATCGTCTCCTTCCGGTTCTGGAAAGACGTCGCTGTTGACATTG
TTTGCGCGTAAACATCCTGAAATTTCCTGCGCACCTATCGCTTTTGATGGTAGCACAGAAGATGCCACAACTCTTCTTTCCACTTAT
GGCGTCAATGTTTATAAGAAGGCGTGCGATATACCCAGTGGCAAGCTGTGTGTCTTGTTGTTGGACGACTGTCAACGGCGGTATAAT
GATCTCGTCTTTTGGACTCGTCTGATTAAAGGCTCAGCATCATGGTTACCAGATCACGTCCGATTTATCATCTCGGCGACTCATTTG
CTGGAGACGGATGCCCCTTCCAGCCCTGTTGCTTTCAGCAGCATCCAGTATAAGTTGACGAGGGACGATTTCTTGATTAATGATGAG
GAGGCGTATCAGTGCTTTAATTTGGAAAACGGCCTACCACCAGGATTGCGTTTTCCAACGCTGGTAGAGGTGATGATACGCGAGTGC
AATGGGCACATCGGTTCATTACGCATATCAATTGATGCAATTTATGAGCGTTTTCGGAGGGCGGGTGCTCGAACTGAGGAGGAGTTG
CTGGCGTTCTATCTGTCGG
>PhCRN02_304 PLHAL3042846
GATGTGTTGGATATGAATGGAATGCCACCGCCTCAAACGCGGCAAATTCACGTGCTGGTGGTGGTTCCGTATCGGACCGAGGATGCC
AACCTTTCCCAGATTTTTCCGACCTTTCGACCATACTGTGAGGGAATCAATGTGCAGCACCCTCATCTTTCGCGGGATGTGCTGGTT
GAAAAACTCTACGAAGCTATTATCCGAACCAATTTTGTGCTGTTATCGTCTCCTTCCGGTTCTGGAAAGACGTCGCTGTTGACATTG
TTTGCGCGTAAACATCCTGAAATTTCCTGCGCACCTATCGCTTTTGATGGTAGCACAGAAGATGCCACAACTCTTCTTTCCACTTAT
GGCGTCAATGTTTATAAGAAGGCGTGCGATATACCCAGTGGCAAGCTGTGTGTCTTGTTGTTGGACGACTGTCAACGGCGGTATAAT
GATCTCGTCTTTTGGACTCGTCTGATTAAAGGCTCAGCATCATGGTTACCAGATCACGTCCGATTTATCATCTCGGCGACTCATTTG
CTGGAGACGGATGCCCCTTCCAGCCCTGTTGCTTTCAGCAGCATCCAGTATAAGTTGACGAGGGACGATTTCTTGATTAATGATGAG
GAGGCGTATCAGTGCTTTAATTTGGAAAACGGCCTACCACCAGGATTGCGTTTTCCAACGCTGGTAGAGGTGATGATACGCGAGTGC
AATGGGCACATCGGTTCATTACGCATATCAATTGATGCAATTTATGAGCGTTTTCGGAGGGCGGGTGCTCGAACTGAGGAGGAGTTG
CTGGCGTTCTATCTGTCGGACTTTTGTCGAACAAATGGCAAGATGCTTCGGAAGTGAACACACAACACCTGCTAGTCCAAACCAGCA
GAAGTTTCTTAGCACGTGTTTGTTGTGCGATCGTCAGCCACTGGTACAGCAATTGAGTCGTGATGAACACGAATGTTTTACAAGGTT
GAAGAAAGCGGGTATTGTAACTGAAGTTGGCGGCT
>PhCRN02_700 PLHAL7004676
TTGGATATGAATGGAATGCCACCGCCTCAAACGCGGCAAATTCACGTGCTGGTGGTGGTTCCGTATCGGACCGAGGATGCCAACCTT
TCCCAGATTTTTCCGACCTTTCGACCATACTGTGAGGGAATCAATGTGCAGCACCCTCATCTTTCGCGGGATGTGCTGGTTGAAAAA
CTCTACGAAGCTATTATCCGAACCAATTTTGTGCTGTTATCGTCTCCTTCCGGTTCTGGAAAGACGTCGCTGTTGACATTGTTTGCG
CGTAAACATCCTGAAATTTCCTGCGCACCTATCGCTTTTGATGGTAGCACAGAAGATGCCACAACTCTTCTTTCCACTTATGGCGTC
AATGTTTATAAGAAGGCGTGCGATATACCCAGTGGCAAGCTGTGTGTCTTGTTGTTGGACGACTGTCAACGGCGGTATAATGATCTC
GTCTTTTGGACTCGTCTGATTAAAGGCTCAGCATCATGGTTACCAGATCACGTCCGATTTATCATCTCGGCGACTCATTTGCTGGAG
ACGGATGCCCCTTCCAGCCCTGTTGCTTTCAGCAGCATCCAGTATAAGTTGACGAGGGACGATTTCTTGATTAATGATGAGGAGGCG
TATCAGTGCTTTAATTTGGAAAACGGCCTACCACCAGGATTGCGTTTTCCAACGCTGGTAGAGGTGATGATACGCGAGTGCAATGGG
CACATCGGTTCATTACGCATATCAATTGATGCAATTTATGAGCGTTTTCGGAGGGCGGGTGCTCGAACTGAGGAGGAGTTGCTGGCG
TTCTATCTGTCGGACTTTTGTCGAACAAATGGCAAGATGCTTCGGAAGTGAACACACAACACCTGCTAGTCCAAACCAGCAGAAGTT
TCTTAGCACGTGTTTGTTGTGCGATC
>PhCRN02_730 PLHAL7303907
GATGTGTTGGATATGAATGGAATGCCACCGCCTCAAACGCGGCAAATTCACGTGCTGGTGGTGGTTCCGTATCGGACCGAGGATGCC
AACCTTTCCCAGATTTTTCCGACCTTTCGACCATACTGTGAGGGAATCAATGTGCAGCACCCTCATCTTTCGCGGGATGTGCTGGTT
GAAAAACTCTACGAAGCTATTATCCGAACCAATTTTGTGCTGTTATCGTCTCCTTCCGGTTCTGGAAAGACGTCGCTGTTGACATTG
TTTGCGCGTAAACATCCTGAAATTTCCTGCGCACCTATCGCTTTTGATGGTAGCACAGAAGATGCCACAACTCTTCTTTCCACTTAT
GGCGTCAATGTTTATAAGAAGGCGTGCGATATACCCAGTGGCAAGCTGTGTGTCTTGTTGTTGGACGACTGTCAACGGCGGTATAAT
GATCTCGTCTTTTGGACTCGTCTGATTAAAGGCTCAGCATCATGGTTACCAGATCACGTCCGATTTATCATCTCGGCGACTCATTTG
CTGGAGACGGATGCCCCTTCCAGCCCTGTTGCTTTCAGCAGCATCCAGTATAAGTTGACGAGGGACGATTTCTTGATTAATGATGAG

GAGGCGTATCAGTGCTTTAATTTGGAAAACGGCCTACCACCAGGATTGCGTTTTCCAACGCTGGTAGAGGTGATGATACGCGAGTGC
AATGGGCACATCGGTTCATTACGCATATCAATTGATGCAATTTATGAGCGTTTTCGGAGGGCGGGTGCTCGAACTGAGGAGGAGTTG
CTGGCGTTCTATCTGTCGGACTTTTGTCGAACAAATGGCAAGATGCTTCGGAAGTGAACACACAACACCTGCTAGTCCAAACCAGCA
GAAGTTTCTTAGCACGTGTTTGTTGTGCGATC

>PhCRN03_100 PLHAL100A13606
AGCGTCAAGTCTTTATTATGGCTATTATTGAAGCTGTATGCCTTATGCTTGGTGATGCCACGATTCTTGTTGAAGAGGATGTGAAAG
GAAAGAACGTACATGTGCACGGTCGCTTCGAGTTTGTGCTGAAGCGTGGCAAGAAGCGTGTATCCATCGTCGAAGCAAAACGCGATG
ATATCCCGCAAGGTATTGCCCAGAATGTGGCCGGCCTCGAAGCTTTGTCTGACGTGGAGGGATTAGAGCGGACACTTGGTATTGTTA
CCAACTATCTGGAGTGGGTATTCATCAGCGATGACGATGAGAAAATTCGACGGATGAATACAACGCTCAAAGTCTATGGAGCGGTGC
CTTCAACTAAAGAACTTAGGGAAATTGTCGGCATGATTTGTGGTTTGCTCGCGAATAGCACTTAATAGTCTCGCAATTACAATATTT
TTTACGAATGCCATTCCGTGTTGGAAAGTTGCTTTCATCAGTTTTATTAGACCCGGCCCACTGCAAGTGCTTGCAGTTAACATCGAA
GCACAGCACAAAATAATTTTTATTCCTGATAAATAAAAA
>PhCRN03_334 PLHAL3343720
AGCGTCAAGTCTTTATTATGGCTATTATTGAAGCTGTATGCCTTATGCTTGGTGATGCCACGATTCTTGTTGAAGAGGATGTGAAAG
GAAAGAACGTACATGTGCACGGTCGCTTCGAGTTTGTGCTGAAGCGTGGCAAGAAGCGTGTATCCATCGTCGAAGCAAAACGCGATG
ATATCCCGCAAGGTATTGCCCAGAATGTGGCCGGCCTCGAAGCTTTGTCTGACGTGGAGGGATTAGAGCGGACACTTGGTATTGTTA
CCAACTATCTGGAGTGGGTATTCATCAGCGATGACGATGAGAAAATTCGACGGATGAATACAACGCTCAAAGTCTATGGAGCGGTGC
CTTCAACTAAAGAACTTAGGGAAATTGTCGGCATGATTTGTGGTTTGCTCGCGAATAGCACTTAATAGTCTCGCAATTACAATATTT
TTTACGAATGCCATTCCGTGTTGGAAAGTTGCTTTCATCAGTTTTATTAGACCCGGCCCACTGCAAGTGCTTGCAGTTAACATCGAA
GCACAGCACAAAATAATTTTTATTCCTGATAAATAAAAA
>PhCRN03_703 PLHAL7033388
AGCGTCAAGTCTTTATTATGGCTATTATTGAAGCTGTATGCCTTATGCTTGGTGATGCCACGATTCTTGTTGAAGAGGATGTGAAAG
GAAAGAACGTACATGTGCACGGTCGCTTCGAGTTTGTGCTGAAGCGTGGCAAGAAGCGTGTATCCATCGTCGAAGCAAAACGCGATG
ATATCCCGCAAGGTATTGCCCAGAATGTGGCCGGCCTCGAAGCTTTGTCTGACGTGGAGGGATTAGAGCGGACACTTGGTATTGTTA
CCAACTATCTGGAGTGGGTATTCATCAGCGATGACGATGAGAAAATTCGACGGATGAATACAACGCTCAAAGTCTATGGAGCGGTGC
CTTCAACTAAAGAACTTAGGGAAATTGTCGGCATGATTTGTGGTTTGCTCGCGAATAGCACTTAATAGTCTCGCAATTACAATATTT
TTTACGAATGCCATTCCGTGTTGGAAAGTTGCTTTCATCAGTTTTATTAGACCCGGCCCACTGCAAGTGCTTGCAGTTAACATCGAA
GCACAGCACAAAATAATTTTTATTCCTGATAAATAAAAA
>PhCRN03_710 PLHAL7103757
AGCGTCAAGTCTTTATTATGGCTATTATTGAAGCTGTATGCCTTATGCTTGGTGATGCCACGATTCTTGTTGAAGAGGATGTGAAAG
GAAAGAACGTACATGTGCACGGTCGCTTCGAGTTTGTGCTGAAGCGTGGCAAGAAGCGTGTATCCATCGTCGAAGCAAAACGCGATG
ATATCCCGCAAGGTATTGCCCAGAATGTGGCCGGCCTCGAAGCTTTGTCTGACGTGGAGGGATTAGAGCGGACACTTGGTATTGTTA
CCAACTATCTGGAGTGGGTATTCATCAGCGATGACGATGAGAAAATTCGACGGATGAATACAACGCTCAAAGTCTATGGAGCGGTGC
CTTCAACTAAAGAACTTAGGGAAATTGTCGGCATGATTTGTGGTTTGCTCGCGAATAGCACTTAATAGTCTCGCAATTACAATATTT
TTTACGAATGCCATTCCGTGTTGGAAAGTTGCTTTCATCAGTTTTATTAGACCCGGCCCACTGCAAGTGCTTGCAGTTAACATCGAA
GCACAGCACAAAATAATTTTTATTCCTGATAAATAAAAA
>PhCRN03_304 PLHAL3043566
AGCGTCAAGTCTTTATTATGGCTATTATTGAAGCTGTATGCCTTATGCTTGGTGATGCCACGATTCTTGTTGAAGAGGATGTGAAAG
GAAAGAACGTACATGTGCACGGTCGCTTCGAGTTTGTGCTGAAGCGTGGCAAGAAGCGTGTATCCATCGTCGAAGCAAAACGCGATG
ATATCCCGCAAGGTATTGCCCAGAATGTGGCCGGCCTCGAAGCTTTGTCTGACGTGGAGGGATTAGAGCGGACACTTGGTATTGTTA
CCAACTATCTGGAGTGGGTATTCATCAGCGATGACGATGAGAAAATTCGACGGATGAATACAACGCTCAAAGTCTATGGAGCGGTGC
CTTCAACTAAAGAACTTAGGGAAATTGTCGGCATGATTTGTGGTTTGCTCGCGAATAGCACTTAATAGTCTCGCAATTACAATATTT
TTTACGAATGCCATTCCGTGTTGGAAAGTTGCTTTCATCAGTTTTATTAGACCCGGCCCACTGCAAGTGCTTGCAGTTAACATCGAA
GCACAGCACAAAATAATTTTTATTCCTGATAAATAAAAA
>PhCRN03_700 PLHAL7004445
AGCGTCAAGTCTTTATTATGGCTATTATTGAAGCTGTATGCCTTATGCTTGGTGATGCCACGATTCTTGTTGAAGAGGATGTGAAAG
GAAAGAACGTACATGTGCACGGTCGCTTCGAGTTTGTGCTGAAGCGTGGCAAGAAGCGTGTATCCATCGTCGAAGCAAAACGCGATG
ATATCCCGCAAGGTATTGCCCAGAATGTGGCCGGCCTCGAAGCTTTGTCTGACGTGGAGGGATTAGAGCGGACACTTGGTATTGTTA
CCAACTATCTGGAGTGGGTATTCATCAGCGATGACGATGAGAAAATTCGACGGATGAATACAACGCTCAAAGTCTATGGAGCGGTGC
CTTCAACTAAAGAACTTAGGGAAATTGTCGGCATGATTTGTGGTTTGCTCGCGAATAGCACTTAATAGTCTCGCAATTACAATATTT
TTTACGAATGCCATTCCGTGTTGGAAAGTTGCTTTCATCAGTTTTATTAGACCCGGCCCACTGCAAGTGCTTGCAGTTAACATCGAA
GCACAGCACAAAATAATTTTTATTCCTGATAAATAAAAA
>PhCRN03_730 PLHAL7303709
AGCGTCAAGTCTTTATTATGGCTATTATTGAAGCTGTATGCCTTATGCTTGGTGATGCCACGATTCTTGTTGAAGAGGATGTGAAAG
GAAAGAACGTACATGTGCACGGTCGCTTCGAGTTTGTGCTGAAGCGTGGCAAGAAGCGTGTATCCATCGTCGAAGCAAAACGCGATG
ATATCCCGCAAGGTATTGCCCAGAATGTGGCCGGCCTCGAAGCTTTGTCTGACGTGGAGGGATTAGAGCGGACACTTGGTATTGTTA
CCAACTATCTGGAGTGGGTATTCATCAGCGATGACGATGAGAAAATTCGACGGATGAATACAACGCTCAAAGTCTATGGAGCGGTGC
CTTCAACTAAAGAACTTAGGGAAATTGTCGGCATGATTTGTGGTTTGCTCGCGAATAGCACTTAATAGTCTCGCAATTACAATATTT
TTTACGAATGCCATTCCGTGTTGGAAAGTTGCTTTCATCAGTTTTATTAGACCCGGCCCACTGCAAGTGCTTGCAGTTAACATCGAA
GCACAGCACAAAATAATTTTTATTCCTGATAAATAAAAA

>PhCRN05_100 PLHAL100A10250
TCCGCCGGCTCGAGGAAAGATGGTGAAGCTCTTCTGTGCGATTGTTGGTGTGGCGGGAAGCGCGTTCTCGGTGGAAATTGACGAAGG
CAAAACGGTGGATGAATTGAAGAAGGCGATCAAGGAGGAGAACTCGGACGACCCAATCCTGAAAAATGTCGCTCCGAAGAACCTGCA
GCTCTTTCTGGCGAAGAAAGACGGCGCGTGGCTGAAGTCGAAGGATCCTGCCGTGATTGCTATGCGGAGTGGGGGTGTTCCCGAGCA
AGTCAAGGCACTGCTGGACGTGGAAATGGATCCAGCAGACGAGATTGGTGACTTGTTTGGTGATGCTCCGACGAAGAAGACCATTCA

TGTGTTGGTGGTGGTTCTAACTCAGCAACAAGCTGCTTCAATCAAGAAGCAACTGCGATACAAGGGAATGAGCACCGAAGCTTCGTG
TCGCAAGTTCTTGGATGCGTTGGCGCAGAATCTCGCCACGCTGTACGACTTTGAGTGCAGTTATGGTGATGTCGCGACAATTGGCGA
CGTCTTCAGTGCTGTCAAAAATGATGAATGGGGTTTTCGTCTGAAGAGAGGGAGGCAGTTGACCAGTGAACCATTGCCAAGCTTCTT
CACCGAGGACGAGTGGAAAGACCTCAAGGATTTGAATTGGCGTACAAATCGCCGTATTCACGATGGCAAGGTTCCCACAACCAGCAA
AGGGAAATCGTACGTCATTCTCCCGCACGCCATTTTCAGTGATGATCGAGTCGACAGATACAAAACTATTGCGACTAGAGCAAGCGT
GGTGTTCGAAGCGACCGATTTCGAAGTGAAAGATGAAGATGAGTTCTCGGGTAGTTCTCGTTCAAGTAGTGGTCGTTCGGATATTGC
ATGATTTCATCGTAGTTCTAGCTGGTACATAAACCTGAATCCAAGCTCAACTACAATTTTTGTTTT
>PhCRN05_334 PLHAL3340253
TCCGCCGGCTCGAGGAAAGATGGTGAAGCTCTTCTGTGCGATTGTTGGTGTGGCGGGAAGCGCGTTCTCGGTGGAAATTGACGAAGG
CAAAACGGTGGATGAATTGAAGAAGGCGATCAAGGAGGAGAACTCGGACGACCCAATCCTGAAAAATGTCGCTCCGAAGAACCTGCA
GCTCTTTCTGGCGAAGAAAGACGGCGCGTGGCTGAAGTCGAAGGATCCTGCCGTGATTGCTATGCGGAGTGGGGGTGTTCCCGAGCA
AGTCAAGGCACTGCTGGACGTGGAAATGGATCCAGCAGACGAGATTGGTGACTTGTTTGGTGATGCTCCGACGAAGAAGACCATTCA
TGTGTTGGTGGTGGTTCTAACTCAGCAACAAGCTGCTTCAATCAAGAAGCAACTGCGATACAAGGGAATGAGCACCGAAGCTTCGTG
TCGCAAGTTCTTGGATGCGTTGGCGCAGAATCTCGCCACGCTGTACGACTTTGAGTGCAGTTATGGTGATGTCGCGACAATTGGCGA
CGTCTTCAGTGCTGTCAAAAATGATGAATGGGGTTTTCGTCTGAAGAGAGGGAGGCAGTTGACCAGTGAACCATTGCCAAGCTTCTT
CACCGAGGACGAGTGGAAAGACCTCAAGGATTTGAATTGGCGTACAAATCGCCGTATTCACGATGGCAAGGTTCCCACAACCAGCAA
AGGGAAATCGTACGTCATTCTCCCGCACGCCATTTTCAGTGATGATCGAGTCGACAGATACAAAACTATTGCGACTAGAGCAAGCGT
GGTGTTCGAAGCGACCGAGTTCGAAGTGAAAGATGAAGATGAGTTCTCGGGTAGTTCTCGTTCAAGTAGTGGTCGTTCGGATATTGC
ATGATTTCATCGTAGTTCTAGCTGGTACATAAACCTGAATCCAAGCTCAACTACAATTTTTGTTTT
>PhCRN05_703 PLHAL7030287
TCCGCCGGCTCGAGGAAAGATGGTGAAGCTCTTCTGTGCGATTGTTGGTGTGGCGGGAAGCGCGTTCTCGGTGGAAATTGACGAAGG
CAAAACGGTGGATGAATTGAAGAAGGCGATCAAGGAGGAGAACTCGGACGACCCAATCCTGAAAAATGTCGCTCCGAAGAACCTGCA
GCTCTTTCTGGCGAAGAAAGACGGCGCGTGGCTGAAGTCGAAGGATCCTGCCGTGATTGCTATGCGGAGTGGGGGTGTTCCCGAGCA
AGTCAAGGCACTGCTGGACGTGGAAATGGATCCAGCAGACGAGATTGGTGACTTGTTTGGTGATGCTCCGACGAAGAAGACCATTCA
TGTGTTGGTGGTGGTTCTAACTCAGCAACAAGCTGCTTCAATCAAGAAGCAACTGCGATACAAGGGAATGAGCACCGAAGCTTCGTG
TCGCAAGTTCTTGGATGCGTTGGCGCAGAATCTCGCCACGCTGTACGACTTTGAGTGCAGTTATGGTGATGTCGCGACAATTGGCGA
CGTCTTCAGTGCTGTCAAAAATGATGAATGGGGTTTTCGTCTGAAGAGAGGGAGGCAGTTGACCAGTGAACCATTGCCAAGCTTCTT
CACCGAGGACGAGTGGAAAGACCTCAAGGATTTGAATTGGCGTACAAATCGCCGTATTCACGATGGCAAGGTTCCCACAACCAGCAA
AGGGAAATCGTACGTCATTCTCCCGCACGCCATTTTCAGTGATGATCGAGTCGACAGATACAAAACTATTGCGACTAGAGCAAGCGT
GGTGTTCGAAGCGACCGAGTTCGAAGTGAAAGATGAAGATGAGTTCTCGGGTAGTTCTCGTTCAAGTAGTGGTCGTTCGGATATTGC
ATGATTTCATCGTAGTTCTAGCTGGTACATAAACCTGAATCCAAGCTCAACTACAATTTTTGTTTT
>PhCRN05_710 PLHAL7100306
TCCGCCGGCTCGAGGAAAGATGGTGAAGCTCTTCTGTGCGATTGTTGGTGTGGCGGGAAGCGCGTTCTCGGTGGAAATTGACGAAGG
CAAAACGGTGGATGAATTGAAGAAGGCGATCAAGGAGGAGAACTCGGACGACCCAATCCTGAAAAATGTCGCTCCGAAGAACCTGCA
GCTCTTTCTGGCGAAGAAAGACGGCGCGTGGCTGAAGTCGAAGGATCCTGCCGTGATTGCTATGCGGAGTGGGGGTGTTCCCGAGCA
AGTCAAGGCACTGCTGGACGTGGAAATGGATCCAGCAGACGAGATTGGTGACTTGTTTGGTGATGCTCCGACGAAGAAGACCATTCA
TGTGTTGGTGGTGGTTCTAACTCAGCAACAAGCTGCTTCAATCAAGAAGCAACTGCGATACAAGGGAATGAGCACCGAAGCTTCGTG
TCGCAAGTTCTTGGATGCGTTGGCGCAGAATCTCGCCACGCTGTACGACTTTGAGTGCAGTTATGGTGATGTCGCGACAATTGGCGA
CGTCTTCAGTGCTGTCAAAAATGATGAATGGGGTTTTCGTCTGAAGAGAGGGAGGCAGTTGACCAGTGAACCATTGCCAAGCTTCTT
CACCGAGGACGAGTGGAAAGACCTCAAGGATTTGAATTGGCGTACAAATCGCCGTATTCACGATGGCAAGGTTCCCACAACCAGCAA
AGGGAAATCGTACGTCATTCTCCCGCACGCCATTTTCAGTGATGATCGAGTCGACAGATACAAAACTATTGCGACTAGAGCAAGCGT
GGTGTTCGAAGCGACCGAGTTCGAAGTGAAAGATGAAGATGAGTTCTCGGGTAGTTCTCGTTCAAGTAGTGGTCGTTCGGATATTGC
ATGATTTCATCGTAGTTCTAGCTGGTACATAAACCTGAATCCAAGCTCAACTACAATTTTTGTTTT
>PhCRN05_304 PLHAL3040260
TCCGCCGGCTCGAGGAAAGATGGTGAAGCTCTTCTGTGCGATTGTTGGTGTGGCGGGAAGCGCGTTCTCGGTGGAAATTGACGAAGG
CAAAACGGTGGATGAATTGAAGAAGGCGATCAAGGAGGAGAACTCGGACGACCCAATCCTGAAAAATGTCGCTCCGAAGAACCTGCA
GCTCTTTCTGGCGAAGAAAGACGGCGCGTGGCTGAAGTCGAAGGATCCTGCCGTGATTGCTATGCGGAGTGGGGGTGTTCCCGAGCA
AGTCAAGGCACTGCTGGACGTGGAAATGGATCCAGCAGACGAGATTGGTGACTTGTTTGGTGATGCTCCGACGAAGAAGACCATTCA
TGTGTTGGTGGTGGTTCTAACTCAGCAACAAGCTGCTTCAATCAAGAAGCAACTGCGATACAAGGGAATGAGCACCGAAGCTTCGTG
TCGCAAGTTCTTGGATGCGTTGGCGCAGAATCTCGCCACGCTGTACGACTTTGAGTGCAGTTATGGTGATGTCGCGACAATTGGCGA
CGTCTTCAGTGCTGTCAAAAATGATGAATGGGGTTTTCGTCTGAAGAGAGGGAGGCAGTTGACCAGTGAACCATTGCCAAGCTTCTT
CACCGAGGACGAGTGGAAAGACCTCAAGGATTTGAATTGGCGTACAAATCGCCGTATTCACGATGGCAAGGTTCCCACAACCAGCAA
AGGGAAATCGTACGTCATTCTCCCGCACGCCATTTTCAGTGATGATCGAGTCGACAGATACAAAACTATTGCGACTAGAGCAAGCGT
GGTGTTCGAAGCGACCGATTTCGAAGTGAAAGATGAAGATGAGTTCTCGGGTAGTTCTCGTTCAAGTAGTGGTCGTTCGGATATTGC
ATGATTTCATCGTAGTTCTAGCTGGTACATAAACCTGAATCCAAGCTCAACTACAATTTTTGTTTT
>PhCRN05_700 PLHAL7000193
TCCGCCGGCTCGAGGAAAGATGGTGAAGCTCTTCTGTGCGATTGTTGGTGTGGCGGGAAGCGCGTTCTCGGTGGAAATTGACGAAGG
CAAAACGGTGGATGAATTGAAGAAGGCGATCAAGGAGGAGAACTCGGACGACCCAATCCTGAAAAATGTCGCTCCGAAGAACCTGCA
GCTCTTTCTGGCGAAGAAAGACGGCGCGTGGCTGAAGTCGAAGGATCCTGCCGTGATTGCTATGCGGAGTGGGGGTGTTCCCGAGCA
AGTCAAGGCACTGCTGGACGTGGAAATGGATCCAGCAGACGAGATTGGTGACTTGTTTGGTGATGCTCCGACGAAGAAGACCATTCA
TGTGTTGGTGGTGGTTCTAACTCAGCAACAAGCTGCTTCAATCAAGAAGCAACTGCGATACAAGGGAATGAGCACCGAAGCTTCGTG
TCGCAAGTTCTTGGATGCGTTGGCGCAGAATCTCGCCACGCTGTACGACTTTGAGTGCAGTTATGGTGATGTCGCGACAATTGGCGA
CGTCTTCAGTGCTGTCAAAAATGATGAATGGGGTTTTCGTCTGAAGAGAGGGAGGCAGTTGACCAGTGAACCATTGCCAAGCTTCTT
CACCGAGGACGAGTGGAAAGACCTCAAGGATTTGAATTGGCGTACAAATCGCCGTATTCACGATGGCAAGGTTCCCACAACCAGCAA
AGGGAAATCGTACGTCATTCTCCCGCACGCCATTTTCAGTGATGATCGAGTCGACAGATACAAAACTATTGCGACTAGAGCAAGCGT
GGTGTTCGAAGCGACCGAGTTCGAAGTGAAAGATGAAGATGAGTTCTCGGGTAGTTCTCGTTCAAGTAGTGGTCGTTCGGATATTGC
ATGATTTCATCGTAGTTCTAGCTGGTACATAAACCTGAATCCAAGCTCAACTACAATTTTTGTTTT

>PhCRN05_730 PLHAL7300262
TCCGCCGGCTCGAGGAAAGATGGTGAAGCTCTTCTGTGCGATTGTTGGTGTGGCGGGAAGCGCGTTCTCGGTGGAAATTGACGAAGG
CAAAACGGTGGATGAATTGAAGAAGGCGATCAAGGAGGAGAACTCGGACGACCCAATCCTGAAAAATGTCGCTCCGAAGAACCTGCA
GCTCTTTCTGGCGAAGAAAGACGGCGCGTGGCTGAAGTCGAAGGATCCTGCCGTGATTGCTATGCGGAGTGGGGGTGTTCCCGAGCA
AGTCAAGGCACTGCTGGACGTGGAAATGGATCCAGCAGACGAGATTGGTGACTTGTTTGGTGATGCTCCGACGAAGAAGACCATTCA
TGTGTTGGTGGTGGTTCTAACTCAGCAACAAGCTGCTTCAATCAAGAAGCAACTGCGATACAAGGGAATGAGCACCGAAGCTTCGTG
TCGCAAGTTCTTGGATGCGTTGGCGCAGAATCTCGCCACGCTGTACGACTTTGAGTGCAGTTATGGTGATGTCGCGACAATTGGCGA
CGTCTTCAGTGCTGTCAAAAATGATGAATGGGGTTTTCGTCTGAAGAGAGGGAGGCAGTTGACCAGTGAACCATTGCCAAGCTTCTT
CACCGAGGACGAGTGGAAAGACCTCAAGGATTTGAATTGGCGTACAAATCGCCGTATTCACGATGGCAAGGTTCCCACAACCAGCAA
AGGGAAATCGTACGTCATTCTCCCGCACGCCATTTTCAGTGATGATCGAGTCGACAGATACAAAACTATTGCGACTAGAGCAAGCGT
GGTGTTCGAAGCGACCGAGTTCGAAGTGAAAGATGAAGATGAGTTCTCGGGTAGTTCTCGTTCAAGTAGTGGTCGTTCGGATATTGC
ATGATTTCATCGTAGTTCTAGCTGGTACATAAACCTGAATCCAAGCTCAACTACAATTTTTGTTTT

>PhCRN06.1_100 PLHAL100A12116
AATCTACCGCCTCGAGGAACGATGGTGACGCTCTTCTGTGCGGTCGTTGGTAAAGAGGGAAGCACGTTCTCCATAGACATTGACACG
AACAAGTCGGTGGACCACTTGAATGATGCGATCATGGCAAAAAAGCCGAACGCCTTTAAAGGTTTTGATGCCGACGAGCTTGAGCTC
TCTCTGGCGAAGAAGGGCGCAGGATGGCTACCAAGTGCGGAACTTAGTGCGATACGAAAAGGAGAGGATATACCGGGTTTCGAAAAG
ATATCACTGGTTGATACAGAGGATGAGCCATATTCGACCTATTCGCTTCGGGTTGTGTTGGAGACGAATCAAATGCCACCGCCTCAA
ACACGACAAATTCACGTGCTGGTGTTGGTTCCAGATCAGCTGTCGGTTTCTGCTGTTGCGACCGTTTCGATCAAGAAGAGAAAGCTA
ACTGA
>PhCRN06.1_334 PLHAL3342222
AATCTACCGCCTCGAGGAACGATGGTGACGCTCTTCTGTGCGGTCGTTGGTAAAGAGGGAAGCACGTTCTCCATAGACATTGACACG
AACAAGTCGGTGGACCACTTGAATGATGCGATCATGGCAAAAAAGCCGAACGCCTTTAAAGGTTTTGATGCCGACGAGCTTGAGCTC
TCTCTGGCGAAGAAGGGCGCAGGATGGCTACCAAGTGCGGAACTTAGTGCGATACGAAAAGGAGAGGATATACCGGGTTTCGAAAAG
ATATCACTGGTTGATACAGAGGATGAGCCATATTCGACCTATTCGCTTCGGGTTGTGTTGGAGACGAATCAAATGCCACCGCCTCAA
ACACGACAAATTCACGTGCTGGTGTTGGTTCCAGATCAGCTGTCGGTTTCTGCTGTTGCGACCGTTTCGATCAAGAAGAGAAAGCTA
ACTGA
>PhCRN06.1_703 PLHAL7031980
AATCTACCGCCTCGAGGAACGATGGTGACGCTCTTCTGTGCGGTCGTTGGTAAAGAGGGAAGCACGTTCTCCATAGACATTGACACG
AACAAGTCGGTGGACCACTTGAATGATGCGATCATGGCAAAAAAGCCGAACGCCTTTAAAGGTTTTGATGCCGACGAGCTTGAGCTC
TCTCTGGCGAAGAAGGGCGCAGGATGGCTACCAAGTGCGGAACTTAGTGCGATACGAAAAGGAGAGGATATACCGGGTTTCGAAAAG
ATATCACTGGTTGATACAGAGGATGAGCCATATTCGACCTATTCGCTTCGGGTTGTGTTGGAGACGAATCAAATGCCACCGCCTCAA
ACACGACAAATTCACGTGCTGGTGTTGGTTCCAGATCAGCTGTCGGTTTCTGCTGTTGCGACCGTTTCGATCAAGAAGAGAAAGCTA
ACTGA
>PhCRN06.1_710 PLHAL7101192
AATCTACCGCCTCGAGGAACGATGGTGACGCTCTTCTGTGCGGTCGTTGGTAAAGAGGGAAGCACGTTCTCCATAGACATTGACACG
AACAAGTCGGTGGACCACTTGAATGATGCGATCATGGCAAAAAAGCCGAACGCCTTTAAAGGTTTTGATGCCGACGAGCTTGAGCTC
TCTCTGGCGAAGAAGGGCGCAGGATGGCTACCAAGTGCGGAACTTAGTGCGATACGAAAAGGAGAGGATATACCGGGTTTCGAAAAG
ATATCACTGGTTGATACAGAGGATGAGCCATATTCGACCTATTCGCTTCGGGTTGTGTTGGAGACGAATCAAATGCCACCGCCTCAA
ACACGACAAATTCACGTGCTGGTGTTGGTTCCAGATCAGCTGTCGGTTTCTGCTGTTGCGACCGTTTCGATCAAGAAGAGAAAGCTA
ACTGA
>PhCRN06.1_304 PLHAL3042116
AATCTACCGCCTCGAGGAACGATGGTGACGCTCTTCTGTGCGGTCGTTGGTAAAGAGGGAAGCACGTTCTCCATAGACATTGACACG
AACAAGTCGGTGGACCACTTGAATGATGCGATCATGGCAAAAAAGCCGAACGCCTTTAAAGGTTTTGATGCCGACGAGCTTGAGCTC
TCTCTGGCGAAGAAGGGCGCAGGATGGCTACCAAGTGCGGAACTTAGTGCGATACGAAAAGGAGAGGATATACCGGGTTTCGAAAAG
ATATCACTGGTTGATACAGAGGATGAGCCATATTCGACCTATTCGCTTCGGGTTGTGTTGGAGACGAATCAAATGCCACCGCCTCAA
ACACGACAAATTCACGTGCTGGTGTTGGTTCCAGATCAGCTGTCGGTTTCTGCTGTTGCGACCGTTTCGATCAAGAAGAGAAAGCTA
ACTGA
>PhCRN06.1_700 PLHAL7002541
AATCTACCGCCTCGAGGAACGATGGTGACGCTCTTCTGTGCGGTCGTTGGTAAAGAGGGAAGCACGTTCTCCATAGACATTGACACG
AACAAGTCGGTGGACCACTTGAATGATGCGATCATGGCAAAAAAGCCGAACGCCTTTAAAGGTTTTGATGCCGACGAGCTTGAGCTC
TCTCTGGCGAAGAAGGGCGCAGGATGGCTACCAAGTGCGGAACTTAGTGCGATACGAAAAGGAGAGGATATACCGGGTTTCGAAAAG
ATATCACTGGTTGATACAGAGGATGAGCCATATTCGACCTATTCGCTTCGGGTTGTGTTGGAGACGAATCAAATGCCACCGCCTCAA
ACACGACAAATTCACGTGCTGGTGTTGGTTCCAGATCAGCTGTCGGTTTCTGCTGTTGCGACCGTTTCGATCAAGAAGAGAAAGCTA
ACTGA
>PhCRN06.1_730 PLHAL7302045
AATCTACCGCCTCGAGGAACGATGGTGACGCTCTTCTGTGCGGTCGTTGGTAAAGAGGGAAGCACGTTCTCCATAGACATTGACACG
AACAAGTCGGTGGACCACTTGAATGATGCGATCATGGCAAAAAAGCCGAACGCCTTTAAAGGTTTTGATGCCGACGAGCTTGAGCTC
TCTCTGGCGAAGAAGGGCGCAGGATGGCTACCAAGTGCGGAACTTAGTGCGATACGAAAAGGAGAGGATATACCGGGTTTCGAAAAG
ATATCACTGGTTGATACAGAGGATGAGCCATATTCGACCTATTCGCTTCGGGTTGTGTTGGAGACGAATCAAATGCCACCGCCTCAA
ACACGACAAATTCACGTGCTGGTGTTGGTTCCAGATCAGCTGTCGGTTTCTGCTGTTGCGACCGTTTCGATCAAGAAGAGAAAGCTA
ACTGA
>PhCRN06.2_100 PLHAL100A11086
TCGTCGTAATGGAGCAACTTTCATTGTGTCCCCTTCGTCACGCCAATGGCATGATAGTTCGTAATCAGCTACTGAAGATCGAAAACA
CTCTGAAAGATGGATCTTTCGTCCACGGTGATCTTCGGGAGCAAAACGTGATGTGGGATACCAGTAAGAACCGAGTCGTATTGATTG
ATTTCGATTGGTCGGGAAGAGATGGTGTCGATACGTATCCACCATTTATGAATTGTGAAATAGCATGGCCAACAGGAGCCGCGTGTG

GCGAGCCGCTTCGAGTTGCCCATGATGCGTATTGGATTGTTTCGATAGCTGCCCGATTGACATAAGGCGAAACTTTAAATTTACCGC
GTAATCTATTACGTTTTATT

>PhCRN06.2_334 PLHAL3341178
TCGTCGTAATGGAGCAACTTTCATTGTGTCCCCTTCGTCACGCCAATGGCATGATAGTTCGTAATCAGCTACTGAAGATCGAAAACA
CTCTGAAAGATGGATCTTTCGTCCACGGTGATCTTCGGGAGCAAAACGTGATGTGGGATACCAGTAAGAACCGAGTCGTATTGATTG
ATTTCGATTGGTCGGGAAGAGATGGTGTCGATACGTATCCACCATTTATGAATTGTGAAATAGCATGGCCAACAGGAGCCGCGTGTG
GCGAGCCGCTTCGAGTTGCCCATGATGCGTATTGGATTGTTTCGATAGCTGCCCGATTGACATAAGGCGAAACTTTAAATTTACCGC
GTAATCTATTACGTTTTATT
>PhCRN06.2_703 PLHAL7030687
TTTGCTCCCGAACTGCTGTTCTGCGAATATCTACCTAATGGTTGGGTATTCGTCGTAATGGAGCAACTTTCATTGTGTCCCCTTCGT
CACGCCAATGGCATGATAGTTCGTAATCAGCTACTGAAGATCGAAAACACTCTGAAAGATGGATCTTTCGTCCACGGTGATCTTCGG
GAGCAAAACGTGATGTGGGATACCAGTAAGAACCGAGTCGTATTGATTGATTTCGATTGGTCGGGAAGAGATGGTGTCGATACGTAT
CCACCATTTATGAATTGTGAAATAGCATGGCCAACAGGAGCCGCGTGTGGCGAGCCGCTTCGAGTTGCCCATGATGCGTATTGGATT
GTTTCGATAGCTGCCCGATTGACATAAGGCGAAACTTTAAATTTACCGCGTAATCTATTACGTTTTATT
>PhCRN06.2_710 PLHAL7100727
ATGGTTGGGTATTCGTCGTAATGGAGCAACTTTCATTGTGTCCCCTTCGTCACGCCAATGGCATGATAGTTCGTAATCAGCTACTGA
AGATCGAAAACACTCTGAAAGATGGATCTTTCGTCCACGGTGATCTTCGGGAGCAAAACGTGATGTGGGATACCAGTAAGAACCGAG
TCGTATTGATTGATTTCGATTGGTCGGGAAGAGATGGTGTCGATACGTATCCACCATTTATGAATTGTGAAATAGCATGGCCAACAG
GAGCCGCGTGTGGCGAGCCGCTTCGAGTTGCCCATGATGCGTATTGGATTGTTTCGATAGCTGCCCGATTGACATAAGGCGAAACTT
TAAATTTACCGCGTAATCTATTACGTTTTATT
>PhCRN06.2_304 PLHAL3041150
GTATTCGTCGTAATGGAGCAACTTTCATTGTGTCCCCTTCGTCACGCCAATGGCATGATAGTTCGTAATCAGCTACTGAAGATCGAA
AACACTCTGAAAGATGGATCTTTCGTCCACGGTGATCTTCGGGAGCAAAACGTGATGTGGGATACCAGTAAGAACCGAGTCGTATTG
ATTGATTTCGATTGGTCGGGAAGAGATGGTGTCGATACGTATCCACCATTTATGAATTGTGAAATAGCATGGCCAACAGGAGCCGCG
TGTGGCGAGCCGCTTCGAGTTGCCCATGATGCGTATTGGATTGTTTCGATAGCTGCCCGATTGACATAAGGCGAAACTTTAAATTTA
CCGCGTAATCTATTACGTTTTATT
>PhCRN06.2_700 PLHAL7005936
TTTGCTCCCGAACTGCTGTTCTGCGAATATCTACCTAATGGTTGGGTATCGTCGTAATGGAGCAACTTTCATTGTGTCCCCTTCGTC
ACGCCAATGGCATGATAGTTCGTAATCAGCTACTGAAGATCGAAAACACTCTGAAAGATGGATCTTTCGTCCACGGTGATCTTCGGG
AGCAAAACGTGATGTGGGATACCAGTAAGAACCGAGTCGTATTGATTGATTTCGATTGGTCGGGAAGAGATGGTGTCGATACGTATC
CACCATTTATGAATTGTGAAATAGCATGGCCAACAGGAGCCGCGTGTGGCGAGCCGCTTCGAGTTGCCCATGATGCGTATTGGATTG
TTTCGATAGCTGCCCGATTGACATAAGGCGAAACTTTAAATTTACCGCGTAATCTATTACGTT
>PhCRN06.2_730 PLHAL7300743
TCGTCGTAATGGAGCAACTTTCATTGTGTCCCCTTCGTCACGCCAATGGCATGATAGTTCGTAATCAGCTACTGAAGATCGAAAACA
CTCTGAAAGATGGATCTTTCGTCCACGGTGATCTTCGGGAGCAAAACGTGATGTGGGATACCAGTAAGAACCGAGTCGTATTGATTG
ATTTCGATTGGTCGGGAAGAGATGGTGTCGATACGTATCCACCATTTATGAATTGTGAAATAGCATGGCCAACAGGAGCCGCGTGTG
GCGAGCCGCTTCGAGTTGCCCATGATGCGTATTGGATTGTTTCGATAGCTGCCCGATTGACATAAGGCGAAACTTTAAATTTACCGC
GTAATCTATTACGTTTTATT

>PhCRN06.3_100 PLHAL100A12992
ACCACCTCAAACGCAGCAAATTCACGTGCTGGTGGTGGTTCCGAAGGGTGAAAACGACCGCTTCGCAGCCATGGCAATCGGCGTGGC
ACCCTCTTTGCCACCAACTACAATACACCGGCACCCCGAACGTCTCAAACGATGGGCTGCGATCAATGCAATGATTCGTCAGAAGAA
TCAAGAGGCGAACCAGAAGACCACGAGTGAAGACGCCAAGAAAACAAACAAAAAGCGGAAAAATCACGATGTCGACAAGACTGTGGG
CTGTTCGAGTCTTTGTTGGGAAGATATCAAACCTATTTACCACTTCGACGATTCTTTCGACCTTCAACCAAGCGATATACCTGATGC
CGACATCGAGCTACTCCTCGCTCGTATTCGTGACTTGCGGGAACTCTATGGACAAATTTCGGATGGGAAAGAAGCCAAGAGATTGTT
TTTTATCGCCCCGATCCTTGAGACAGTCAGTCGTCTGCTCAAGAACGTCCGCATATTAGTCGAAGAAGACGTGGTTGGGAAGAACGT
GCTCCTGAAAGGTCGATTCGAGTTCGTGCTCAAGCGTGGCACCAAGAGAATTTCACTTGTCGAAGCAAAGCGTGAAGATATGCTGCA
AGGAATGGTTCAGAATGTGACGGGGCTCGAAGCTCTTGCTGATGTCGAAGATCTTACTGTGACGTATGGCATTGTGACGAATTTTCT
CGAGTGGAAGTTCTTGATCAGTGGCGACGAGAAGGTCAGAGAGCATGAGACTGTTCTTCCTCAAGCGAATACCATACCGTCGTTTGA
AGGATTGAAAGAAATTGTGGGGAAAATTTACGCGATGCTGCAATAAATATCTTCCATATGAGTTGCAATCCTTTTGCTTATTTTCTA
ATTGAGATCTTCTTACATGCACCCATCTGCTTAATCATGGCATCGAAAGGTATCTCTGATCGTCCGCTCAACGATAGCACTTAAGAG
CATCTAGAGTAATCCGTCAAGCTACCGGTGAAACTAAAGAATGAGAGATCAACAGAGCGCGCGCCGCACCTCCAGGATTGAATCAAG
TAATATTTGTTTTATTACTGATAAATATAAAGTTAGTAATATACAAAGATGGCGTACTCACCT
>PhCRN06.3_334 PLHAL3343205
CCAACTACAATACACCGGCACCCCGAACGTCTCAAACGATGGGCTGCGATCAATGCAATGATTCGTCAGAAGAATCAAGAGGCGAAC
CAGAAGACCACGAGTGAAGACGCCAAGAAAACAAGCAAAAAGCGGAAAAATCACGATGTCGACAAGACTGTGGGCTGTTCGAGTCTT
TGTTGGGAAGATATCAAACCTATTTACCACTTCGACGATTCTTTCGACCTTCAACCAAGCGATATACCTGATGCCGACATCGAGCTA
CTCCTCGCTCGTATTCGTGACTTGCGGGAACTCTATGGACAAATTTCGGATGGGAAAGAAGCCAAGAGATTGTTTTTTATCGCCCCG
ATCCTTGAGACAGTCAGTCGTCTGCTCAAGAACGTCCGCATATTAGTCGAAGAAGACGTGGTTGGGAAGAACGTGCTCCTGAAAGGT
CGATTCGAGTTCGTGCTCAAGCGTGGCACCAAGAGAATTTCACTTGTCGAAGCAAAGCGTGAAGATATGCTGCAAGGAATGGTTCAG
AATGTGACGGGGCTCGAAGCTCTTGCTGATGTCGAAGATCTTACTGTGACGTATGGCATTGTGACGAATTTTCTCGAGTGGAAGTTC
TTGATCAGTGGCGACGAGAAGGTCAGAGAGCATGAGACTGTTCTTCCTCAAGCGAATACCATACCGTCGTTTGAAGGATTGAAAGAA
ATTGTGGGGAAAATTTACGCGATGCTGCAATAAATATCTTCCATATGAGTTGCAATCCTTTTGCTTATTTTCTAATTGAGATCTTCT
TACATGCACCCATCTGCTTAATCATGGCATCGAAAGGTATCTCTGATCGTCCGCTCAACGATAGCACTTAAGAGCATCTAGAGTAAT
CCGTCAAGCTACCGGTGAAACTAAAGAATGAGAGATCAACAGAGCGCGCGCCGCACCTCCAGGATTGAATCAAGTAATATTTGTTTT
ATTACTGATAAATATAAAGTTAGTAATATACAAAGATGGCGTACTCACCT

>PhCRN06.3_703 PLHAL7032740
ACCACCTCAAACGCAGCAAATTCACGTGCTGGTGGTGGTTCCGAAGGGTGAAAACGACCGCTTCGCAGCCATGGCAATCGGCGTGGC
ACCCTCTTTGCCACCAACTACAATACACCGGCACCCCGAACGTCTCAAACGATGGGCTGCGATCAATGCAATGATTCGTCAGAAGAA
TCAAGAGGCGAACCAGAAGACCACGAGTGAAGACGCCAAGAAAACAAGCAAAAAGCGGAAAAATCACGATGTCGACAAGACTGTGGG
CTGTTCGAGTCTTTGTTGGGAAGATATCAAACCTATTTACCACTTCGACGATTCTTTCGACCTTCAACCAAGCGATATACCTGATGC
CGACATCGAGCTACTCCTCGCTCGTATTCGTGACTTGCGGGAACTCTATGGACAAATTTCGGATGGGAAAGAAGCCAAGAGATTGTT
TTTTATCGCCCCGATCCTTGAGACAGTCAGTCGTCTGCTCAAGAACGTCCGCATATTAGTCGAAGAAGACGTGGTTGGGAAGAACGT
GCTCCTGAAAGGTCGATTCGAGTTCGTGCTCAAGCGTGGCACCAAGAGAATTTCACTTGTCGAAGCAAAGCGTGAAGATATGCTGCA
AGGAATGGTTCAGAATGTGACGGGGCTCGAAGCTCTTGCTGATGTCGAAGATCTTACTGTGACGTATGGCATTGTGACGAATTTTCT
CGAGTGGAAGTTCTTGATCAGTGGCGACGAGAAGGTCAGAGAGCATGAGACTGTTCTTCCTCAAGCGAATACCATACCGTCGTTTGA
AGGATTGAAAGAAATTGTGGGGAAAATTTACGCGATGCTGCAATAAATATCTTCCATATGAGTTGCAATCCTTTTGCTTATTTTCTA
ATTGAGATCTTCTTACATGCACCCATCTGCTTAATCATGGCATCGAAAGGTATCTCTGATCGTCCGCTCAACGATAGCACTTAAGAG
CATCTAGAGTAATCCGTCAAGCTACCGGTGAAACTAAAGAATGAGAGATCAACAGAGCGCGCGCCGCACCTCCAGGATTGAATCAAG
TAATATTTGTTTTATTACTGATAAATATAAAGTTAGTAATATACAAAGATGGCGTACTCACCT
>PhCRN06.3_710 PLHAL7102598
ACCACCTCAAACGCAGCAAATTCACGTGCTGGTGGTGGTTCCGAAGGGTGAAAACGACCGCTTCGCAGCCATGGCAATCGGCGTGGC
ACCCTCTTTGCCACCAACTACAATACACCGGCACCCCGAACGTCTCAAACGATGGGCTGCGATCAATGCAATGATTCGTCAGAAGAA
TCAAGAGGCGAACCAGAAGACCACGAGTGAAGACGCCAAGAAAACAAGCAAAAAGCGGAAAAATCACGATGTCGACAAGACTGTGGG
CTGTTCGAGTCTTTGTTGGGAAGATATCAAACCTATTTACCACTTCGACGATTCTTTCGACCTTCAACCAAGCGATATACCTGATGC
CGACATCGAGCTACTCCTCGCTCGTATTCGTGACTTGCGGGAACTCTATGGACAAATTTCGGATGGGAAAGAAGCCAAGAGATTGTT
TTTTATCGCCCCGATCCTTGAGACAGTCAGTCGTCTGCTCAAGAACGTCCGCATATTAGTCGAAGAAGACGTGGTTGGGAAGAACGT
GCTCCTGAAAGGTCGATTCGAGTTCGTGCTCAAGCGTGGCACCAAGAGAATTTCACTTGTCGAAGCAAAGCGTGAAGATATGCTGCA
AGGAATGGTTCAGAATGTGACGGGGCTCGAAGCTCTTGCTGATGTCGAAGATCTTACTGTGACGTATGGCATTGTGACGAATTTTCT
CGAGTGGAAGTTCTTGATCAGTGGCGACGAGAAGGTCAGAGAGCATGAGACTGTTCTTCCTCAAGCGAATACCATACCGTCGTTTGA
AGGATTGAAAGAAATTGTGGGGAAAATTTACGCGATGCTGCAATAAATATCTTCCATATGAGTTGCAATCCTTTTGCTTATTTTCTA
ATTGAGATCTTCTTACATGCACCCATCTGCTTAATCATGGCATCGAAAGGTATCTCTGATCGTCCGCTCAACGATAGCACTTAAGAG
CATCTAGAGTAATCCGTCAAGCTACCGGTGAAACTAAAGAATGAGAGATCAACAGAGCGCGCGCCGCACCTCCAGGATTGAATCAAG
TAATATTTGTTTTATTACTGATAAATATAAAGTTAGTAATATACAAAGATGGCGTACTCACCT
>PhCRN06.3_304 PLHAL3042731
ACCACCTCAAACGCAGCAAATTCACGTGCTGGTGGTGGTTCCGAAGGGTGAAAACGACCGCTCCGCAGCCATGGCAATCGGCGTGGC
ACCCTCTTTGCCACCAACTACAATACACCGGCACCCCGAACGTCTCAAACGATGGGCTGCGATCAATGCAATGATTCGTCAGAAGAA
TCAAGAGGCGAACCAGAAGACCACGAGTGAAGACGCCAAGAAAACAAACAAAAAGCGGAAAAATCACGATGTCGACAAGACTGTGGG
CTGTTCGAGTCTTTGTTGGGAAGATATCAAACCTATTTACCACTTCGACGATTCTTTCGACCTTCAACCAAGCGATATACCTGATGC
CGACATCGAGCTACTCCTCGCTCGTATTCGTGACTTGCGGGAACTCTATGGACAAATTTCGGATGGGAAAGAAGCCAAGAGATTGTT
TTTTATCGCCCCGATCCTTGAGACAGTCAGTCGTCTGCTCAAGAACGTCCGCATATTAGTCGAAGAAGACGTGGTTGGGAAGAACGT
GCTCCTGAAAGGTCGATTCGAGTTCGTGCTCAAGCGTGGCACCAAGAGAATTTCACTTGTCGAAGCAAAGCGTGAAGATATGCTGCA
AGGAATGGTTCAGAATGTGACGGGGCTCGAAGCTCTTGCTGATGTCGAAGATCTTACTGTGACGTATGGCATTGTGACGAATTTTCT
CGAGTGGAAGTTCTTGATCAGTGGCGACGAGAAGGTCAGAGAGCATGAGACTGTTCTTCCTCAAGCGAATACCATACCGTCGTTTGA
AGGATTGAAAGAAATTGTGGGGAAAATTTACGCGATGCTGCAATAAATATCTTCCATATGAGTTGCAATCCTTTTGCTTATTTTCTA
ATTGAGATCTTCTTACATGCACCCATCTGCTTAATCATGGCATCGAAAGGTATCTCTGATCGTCCGCTCAACGATAGCACTTAAGAG
CATCTAGAGTAATCCGTCAAGCTACCGGTGAAACTAAAGAATGAGAGATCAACAGAGCGCGCGCCGCACCTCCAGGATTGAATCAAG
TAATATTTGTTTTATTACTGATAAATATAAAGTTAGTAATATACAAAGATGGCGTACTCACCT
>PhCRN06.3_700 PLHAL7003619
TCGCAGCCATGGCAATCGGCGTGGCACCCTCTTTGCCACCAACTACAATACACCGGCACCCCGAACGTCTCAAACGATGGGCTGCGA
TCAATGCAATGATTCGTCAGAAGAATCAAGAGGCGAACCAGAAGACCACGAGTGAAGACGCCAAGAAAACAAGCAAAAAGCGGAAAA
ATCACGATGTCGACAAGACTGTGGGCTGTTCGAGTCTTTGTTGGGAAGATATCAAACCTATTTACCACTTCGACGATTCTTTCGACC
TTCAACCAAGCGATATACCTGATGCCGACATCGAGCTACTCCTCGCTCGTATTCGTGACTTGCGGGAACTCTATGGACAAATTTCGG
ATGGGAAAGAAGCCAAGAGATTGTTTTTTATCGCCCCGATCCTTGAGACAGTCAGTCGTCTGCTCAAGAACGTCCGCATATTAGTCG
AAGAAGACGTGGTTGGGAAGAACGTGCTCCTGAAAGGTCGATTCGAGTTCGTGCTCAAGCGTGGCACCAAGAGAATTTCACTTGTCG
AAGCAAAGCGTGAAGATATGCTGCAAGGAATGGTTCAGAATGTGACGGGGCTCGAAGCTCTTGCTGATGTCGAAGATCTTACTGTGA
CGTATGGCATTGTGACGAATTTTCTCGAGTGGAAGTTCTTGATCAGTGGCGACGAGAAGGTCAGAGAGCATGAGACTGTTCTTCCTC
AAGCGAATACCATACCGTCGTTTGAAGGATTGAAAGAAATTGTGGGGAAAATTTACGCGATGCTGCAATAAATATCTTCCATATGAG
TTGCAATCCTTTTGCTTATTTTCTAATTGAGATCTTCTTACATGCACCCATCTGCTTAATCATGGCATCGAAAGGTATCTCTGATCG
TCCGCTCAACGATAGCACTTAAGAGCATCTAGAGTAATCCGTCAAGCTACCGGTGAAACTAAAGAATGAGAGATCAACAGAGCGCGC
GCCGCACCTCCAGGATTGAATCAAGTAATATTTGTTTTATTACTGATAAATATAAAGTTAGTAATATACAAAGATGGCGTACTCACC
T
>PhCRN06.3_730 PLHAL7302988
ACCACCTCAAACGCAGCAAATTCACGTGCTGGTGGTGGTTCCGAAGGGTGAAAACGACCGCTTCGCAGCCATGGCAATCGGCGTGGC
ACCCTCTTTGCCACCAACTACAATACACCGGCACCCCGAACGTCTCAAACGATGGGCTGCGATCAATGCAATGATTCGTCAGAAGAA
TCAAGAGGCGAACCAGAAGACCACGAGTGAAGACGCCAAGAAAACAAGCAAAAAGCGGAAAAATCACGATGTCGACAAGACTGTGGG
CTGTTCGAGTCTTTGTTGGGAAGATATCAAACCTATTTACCACTTCGACGATTCTTTCGACCTTCAACCAAGCGATATACCTGATGC
CGACATCGAGCTACTCCTCGCTCGTATTCGTGACTTGCGGGAACTCTATGGACAAATTTCGGATGGGAAAGAAGCCAAGAGATTGTT
TTTTATCGCCCCGATCCTTGAGACAGTCAGTCGTCTGCTCAAGAACGTCCGCATATTAGTCGAAGAAGACGTGGTTGGGAAGAACGT
GCTCCTGAAAGGTCGATTCGAGTTCGTGCTCAAGCGTGGCACCAAGAGAATTTCACTTGTCGAAGCAAAGCGTGAAGATATGCTGCA
AGGAATGGTTCAGAATGTGACGGGGCTCGAAGCTCTTGCTGATGTCGAAGATCTTACTGTGACGTATGGCATTGTGACGAATTTTCT
CGAGTGGAAGTTCTTGATCAGTGGCGACGAGAAGGTCAGAGAGCATGAGACTGTTCTTCCTCAAGCGAATACCATACCGTCGTTTGA
AGGATTGAAAGAAATTGTGGGGAAAATTTACGCGATGCTGCAATAAATATCTTCCATATGAGTTGCAATCCTTTTGCTTATTTTCTA

ATTGAGATCTTCTTACATGCACCCATCTGCTTAATCATGGCATCGAAAGGTATCTCTGATCGTCCGCTCAACGATAGCACTTAAGAG
CATCTAGAGTAATCCGTCAAGCTACCGGTGAAACTAAAGAATGAGAGATCAACAGAGCGCGCGCCGCACCTCCAGGATTGAATCAAG
TAATATTTGTTTTATTACTGATAAATATAAAGTTAGTAATATACAAAGATGGCGTACTCACCT

>PhCRN07.1_100 PLHAL100A12992
ACCACCTCAAACGCAGCAAATTCACGTGCTGGTGGTGGTTCCGAAGGGTGAAAACGACCGCTTCGCAGCCATGGCAATCGGCGTGGC
ACCCTCTTTGCCACCAACTACAATACACCGGCACCCCGAACGTCTCAAACGATGGGCTGCGATCAATGCAATGATTCGTCAGAAGAA
TCAAGAGGCGAACCAGAAGACCACGAGTGAAGACGCCAAGAAAACAAACAAAAAGCGGAAAAATCACGATGTCGACAAGACTGTGGG
CTGTTCGAGTCTTTGTTGGGAAGATATCAAACCTATTTACCACTTCGACGATTCTTTCGACCTTCAACCAAGCGATATACCTGATGC
CGACATCGAGCTACTCCTCGCTCGTATTCGTGACTTGCGGGAACTCTATGGACAAATTTCGGATGGGAAAGAAGCCAAGAGATTGTT
TTTTATCGCCCCGATCCTTGAGACAGTCAGTCGTCTGCTCAAGAACGTCCGCATATTAGTCGAAGAAGACGTGGTTGGGAAGAACGT
GCTCCTGAAAGGTCGATTCGAGTTCGTGCTCAAGCGTGGCACCAAGAGAATTTCACTTGTCGAAGCAAAGCGTGAAGATATGCTGCA
AGGAATGGTTCAGAATGTGACGGGGCTCGAAGCTCTTGCTGATGTCGAAGATCTTACTGTGACGTATGGCATTGTGACGAATTTTCT
CGAGTGGAAGTTCTTGATCAGTGGCGACGAGAAGGTCAGAGAGCATGAGACTGTTCTTCCTCAAGCGAATACCATACCGTCGTTTGA
AGGATTGAAAGAAATTGTGGGGAAAATTTACGCGATGCTGCAATAAATATCTTCCATATGAGTTGCAATCCTTTTGCTTATTTTCTA
ATTGAGATCTTCTTACATGCACCCATCTGCTTAATCATGGCATCGAAAGGTATCTCTGATCGTCCGCTCAACGATAGCACTTAAGAG
CATCTAGAGTAATCCGTCAAGCTACCGGTGAAACTAAAGAATGAGAGATCAACAGAGCGCGCGCCGCACCTCCAGGATTGAATCAAG
TAATATTTGTTTTATTACTGATAAATATAAAGTTAGTAATATACAAAGATGGCGTACTCACCT
>PhCRN07.1_334 PLHAL3343205
CCAACTACAATACACCGGCACCCCGAACGTCTCAAACGATGGGCTGCGATCAATGCAATGATTCGTCAGAAGAATCAAGAGGCGAAC
CAGAAGACCACGAGTGAAGACGCCAAGAAAACAAGCAAAAAGCGGAAAAATCACGATGTCGACAAGACTGTGGGCTGTTCGAGTCTT
TGTTGGGAAGATATCAAACCTATTTACCACTTCGACGATTCTTTCGACCTTCAACCAAGCGATATACCTGATGCCGACATCGAGCTA
CTCCTCGCTCGTATTCGTGACTTGCGGGAACTCTATGGACAAATTTCGGATGGGAAAGAAGCCAAGAGATTGTTTTTTATCGCCCCG
ATCCTTGAGACAGTCAGTCGTCTGCTCAAGAACGTCCGCATATTAGTCGAAGAAGACGTGGTTGGGAAGAACGTGCTCCTGAAAGGT
CGATTCGAGTTCGTGCTCAAGCGTGGCACCAAGAGAATTTCACTTGTCGAAGCAAAGCGTGAAGATATGCTGCAAGGAATGGTTCAG
AATGTGACGGGGCTCGAAGCTCTTGCTGATGTCGAAGATCTTACTGTGACGTATGGCATTGTGACGAATTTTCTCGAGTGGAAGTTC
TTGATCAGTGGCGACGAGAAGGTCAGAGAGCATGAGACTGTTCTTCCTCAAGCGAATACCATACCGTCGTTTGAAGGATTGAAAGAA
ATTGTGGGGAAAATTTACGCGATGCTGCAATAAATATCTTCCATATGAGTTGCAATCCTTTTGCTTATTTTCTAATTGAGATCTTCT
TACATGCACCCATCTGCTTAATCATGGCATCGAAAGGTATCTCTGATCGTCCGCTCAACGATAGCACTTAAGAGCATCTAGAGTAAT
CCGTCAAGCTACCGGTGAAACTAAAGAATGAGAGATCAACAGAGCGCGCGCCGCACCTCCAGGATTGAATCAAGTAATATTTGTTTT
ATTACTGATAAATATAAAGTTAGTAATATACAAAGATGGCGTACTCACCT
>PhCRN07.1_703 PLHAL7032740
ACCACCTCAAACGCAGCAAATTCACGTGCTGGTGGTGGTTCCGAAGGGTGAAAACGACCGCTTCGCAGCCATGGCAATCGGCGTGGC
ACCCTCTTTGCCACCAACTACAATACACCGGCACCCCGAACGTCTCAAACGATGGGCTGCGATCAATGCAATGATTCGTCAGAAGAA
TCAAGAGGCGAACCAGAAGACCACGAGTGAAGACGCCAAGAAAACAAGCAAAAAGCGGAAAAATCACGATGTCGACAAGACTGTGGG
CTGTTCGAGTCTTTGTTGGGAAGATATCAAACCTATTTACCACTTCGACGATTCTTTCGACCTTCAACCAAGCGATATACCTGATGC
CGACATCGAGCTACTCCTCGCTCGTATTCGTGACTTGCGGGAACTCTATGGACAAATTTCGGATGGGAAAGAAGCCAAGAGATTGTT
TTTTATCGCCCCGATCCTTGAGACAGTCAGTCGTCTGCTCAAGAACGTCCGCATATTAGTCGAAGAAGACGTGGTTGGGAAGAACGT
GCTCCTGAAAGGTCGATTCGAGTTCGTGCTCAAGCGTGGCACCAAGAGAATTTCACTTGTCGAAGCAAAGCGTGAAGATATGCTGCA
AGGAATGGTTCAGAATGTGACGGGGCTCGAAGCTCTTGCTGATGTCGAAGATCTTACTGTGACGTATGGCATTGTGACGAATTTTCT
CGAGTGGAAGTTCTTGATCAGTGGCGACGAGAAGGTCAGAGAGCATGAGACTGTTCTTCCTCAAGCGAATACCATACCGTCGTTTGA
AGGATTGAAAGAAATTGTGGGGAAAATTTACGCGATGCTGCAATAAATATCTTCCATATGAGTTGCAATCCTTTTGCTTATTTTCTA
ATTGAGATCTTCTTACATGCACCCATCTGCTTAATCATGGCATCGAAAGGTATCTCTGATCGTCCGCTCAACGATAGCACTTAAGAG
CATCTAGAGTAATCCGTCAAGCTACCGGTGAAACTAAAGAATGAGAGATCAACAGAGCGCGCGCCGCACCTCCAGGATTGAATCAAG
TAATATTTGTTTTATTACTGATAAATATAAAGTTAGTAATATACAAAGATGGCGTACTCACCT
>PhCRN07.1_710 PLHAL7102598
ACCACCTCAAACGCAGCAAATTCACGTGCTGGTGGTGGTTCCGAAGGGTGAAAACGACCGCTTCGCAGCCATGGCAATCGGCGTGGC
ACCCTCTTTGCCACCAACTACAATACACCGGCACCCCGAACGTCTCAAACGATGGGCTGCGATCAATGCAATGATTCGTCAGAAGAA
TCAAGAGGCGAACCAGAAGACCACGAGTGAAGACGCCAAGAAAACAAGCAAAAAGCGGAAAAATCACGATGTCGACAAGACTGTGGG
CTGTTCGAGTCTTTGTTGGGAAGATATCAAACCTATTTACCACTTCGACGATTCTTTCGACCTTCAACCAAGCGATATACCTGATGC
CGACATCGAGCTACTCCTCGCTCGTATTCGTGACTTGCGGGAACTCTATGGACAAATTTCGGATGGGAAAGAAGCCAAGAGATTGTT
TTTTATCGCCCCGATCCTTGAGACAGTCAGTCGTCTGCTCAAGAACGTCCGCATATTAGTCGAAGAAGACGTGGTTGGGAAGAACGT
GCTCCTGAAAGGTCGATTCGAGTTCGTGCTCAAGCGTGGCACCAAGAGAATTTCACTTGTCGAAGCAAAGCGTGAAGATATGCTGCA
AGGAATGGTTCAGAATGTGACGGGGCTCGAAGCTCTTGCTGATGTCGAAGATCTTACTGTGACGTATGGCATTGTGACGAATTTTCT
CGAGTGGAAGTTCTTGATCAGTGGCGACGAGAAGGTCAGAGAGCATGAGACTGTTCTTCCTCAAGCGAATACCATACCGTCGTTTGA
AGGATTGAAAGAAATTGTGGGGAAAATTTACGCGATGCTGCAATAAATATCTTCCATATGAGTTGCAATCCTTTTGCTTATTTTCTA
ATTGAGATCTTCTTACATGCACCCATCTGCTTAATCATGGCATCGAAAGGTATCTCTGATCGTCCGCTCAACGATAGCACTTAAGAG
CATCTAGAGTAATCCGTCAAGCTACCGGTGAAACTAAAGAATGAGAGATCAACAGAGCGCGCGCCGCACCTCCAGGATTGAATCAAG
TAATATTTGTTTTATTACTGATAAATATAAAGTTAGTAATATACAAAGATGGCGTACTCACCT
>PhCRN07.1_304 PLHAL3042731
ACCACCTCAAACGCAGCAAATTCACGTGCTGGTGGTGGTTCCGAAGGGTGAAAACGACCGCTCCGCAGCCATGGCAATCGGCGTGGC
ACCCTCTTTGCCACCAACTACAATACACCGGCACCCCGAACGTCTCAAACGATGGGCTGCGATCAATGCAATGATTCGTCAGAAGAA
TCAAGAGGCGAACCAGAAGACCACGAGTGAAGACGCCAAGAAAACAAACAAAAAGCGGAAAAATCACGATGTCGACAAGACTGTGGG
CTGTTCGAGTCTTTGTTGGGAAGATATCAAACCTATTTACCACTTCGACGATTCTTTCGACCTTCAACCAAGCGATATACCTGATGC
CGACATCGAGCTACTCCTCGCTCGTATTCGTGACTTGCGGGAACTCTATGGACAAATTTCGGATGGGAAAGAAGCCAAGAGATTGTT
TTTTATCGCCCCGATCCTTGAGACAGTCAGTCGTCTGCTCAAGAACGTCCGCATATTAGTCGAAGAAGACGTGGTTGGGAAGAACGT
GCTCCTGAAAGGTCGATTCGAGTTCGTGCTCAAGCGTGGCACCAAGAGAATTTCACTTGTCGAAGCAAAGCGTGAAGATATGCTGCA

AGGAATGGTTCAGAATGTGACGGGGCTCGAAGCTCTTGCTGATGTCGAAGATCTTACTGTGACGTATGGCATTGTGACGAATTTTCT
CGAGTGGAAGTTCTTGATCAGTGGCGACGAGAAGGTCAGAGAGCATGAGACTGTTCTTCCTCAAGCGAATACCATACCGTCGTTTGA
AGGATTGAAAGAAATTGTGGGGAAAATTTACGCGATGCTGCAATAAATATCTTCCATATGAGTTGCAATCCTTTTGCTTATTTTCTA
ATTGAGATCTTCTTACATGCACCCATCTGCTTAATCATGGCATCGAAAGGTATCTCTGATCGTCCGCTCAACGATAGCACTTAAGAG
CATCTAGAGTAATCCGTCAAGCTACCGGTGAAACTAAAGAATGAGAGATCAACAGAGCGCGCGCCGCACCTCCAGGATTGAATCAAG
TAATATTTGTTTTATTACTGATAAATATAAAGTTAGTAATATACAAAGATGGCGTACTCACCT
>PhCRN07.1_700 PLHAL7003619
TCGCAGCCATGGCAATCGGCGTGGCACCCTCTTTGCCACCAACTACAATACACCGGCACCCCGAACGTCTCAAACGATGGGCTGCGA
TCAATGCAATGATTCGTCAGAAGAATCAAGAGGCGAACCAGAAGACCACGAGTGAAGACGCCAAGAAAACAAGCAAAAAGCGGAAAA
ATCACGATGTCGACAAGACTGTGGGCTGTTCGAGTCTTTGTTGGGAAGATATCAAACCTATTTACCACTTCGACGATTCTTTCGACC
TTCAACCAAGCGATATACCTGATGCCGACATCGAGCTACTCCTCGCTCGTATTCGTGACTTGCGGGAACTCTATGGACAAATTTCGG
ATGGGAAAGAAGCCAAGAGATTGTTTTTTATCGCCCCGATCCTTGAGACAGTCAGTCGTCTGCTCAAGAACGTCCGCATATTAGTCG
AAGAAGACGTGGTTGGGAAGAACGTGCTCCTGAAAGGTCGATTCGAGTTCGTGCTCAAGCGTGGCACCAAGAGAATTTCACTTGTCG
AAGCAAAGCGTGAAGATATGCTGCAAGGAATGGTTCAGAATGTGACGGGGCTCGAAGCTCTTGCTGATGTCGAAGATCTTACTGTGA
CGTATGGCATTGTGACGAATTTTCTCGAGTGGAAGTTCTTGATCAGTGGCGACGAGAAGGTCAGAGAGCATGAGACTGTTCTTCCTC
AAGCGAATACCATACCGTCGTTTGAAGGATTGAAAGAAATTGTGGGGAAAATTTACGCGATGCTGCAATAAATATCTTCCATATGAG
TTGCAATCCTTTTGCTTATTTTCTAATTGAGATCTTCTTACATGCACCCATCTGCTTAATCATGGCATCGAAAGGTATCTCTGATCG
TCCGCTCAACGATAGCACTTAAGAGCATCTAGAGTAATCCGTCAAGCTACCGGTGAAACTAAAGAATGAGAGATCAACAGAGCGCGC
GCCGCACCTCCAGGATTGAATCAAGTAATATTTGTTTTATTACTGATAAATATAAAGTTAGTAATATACAAAGATGGCGTACTCACC
T
>PhCRN07.1_730 PLHAL7302988
ACCACCTCAAACGCAGCAAATTCACGTGCTGGTGGTGGTTCCGAAGGGTGAAAACGACCGCTTCGCAGCCATGGCAATCGGCGTGGC
ACCCTCTTTGCCACCAACTACAATACACCGGCACCCCGAACGTCTCAAACGATGGGCTGCGATCAATGCAATGATTCGTCAGAAGAA
TCAAGAGGCGAACCAGAAGACCACGAGTGAAGACGCCAAGAAAACAAGCAAAAAGCGGAAAAATCACGATGTCGACAAGACTGTGGG
CTGTTCGAGTCTTTGTTGGGAAGATATCAAACCTATTTACCACTTCGACGATTCTTTCGACCTTCAACCAAGCGATATACCTGATGC
CGACATCGAGCTACTCCTCGCTCGTATTCGTGACTTGCGGGAACTCTATGGACAAATTTCGGATGGGAAAGAAGCCAAGAGATTGTT
TTTTATCGCCCCGATCCTTGAGACAGTCAGTCGTCTGCTCAAGAACGTCCGCATATTAGTCGAAGAAGACGTGGTTGGGAAGAACGT
GCTCCTGAAAGGTCGATTCGAGTTCGTGCTCAAGCGTGGCACCAAGAGAATTTCACTTGTCGAAGCAAAGCGTGAAGATATGCTGCA
AGGAATGGTTCAGAATGTGACGGGGCTCGAAGCTCTTGCTGATGTCGAAGATCTTACTGTGACGTATGGCATTGTGACGAATTTTCT
CGAGTGGAAGTTCTTGATCAGTGGCGACGAGAAGGTCAGAGAGCATGAGACTGTTCTTCCTCAAGCGAATACCATACCGTCGTTTGA
AGGATTGAAAGAAATTGTGGGGAAAATTTACGCGATGCTGCAATAAATATCTTCCATATGAGTTGCAATCCTTTTGCTTATTTTCTA
ATTGAGATCTTCTTACATGCACCCATCTGCTTAATCATGGCATCGAAAGGTATCTCTGATCGTCCGCTCAACGATAGCACTTAAGAG
CATCTAGAGTAATCCGTCAAGCTACCGGTGAAACTAAAGAATGAGAGATCAACAGAGCGCGCGCCGCACCTCCAGGATTGAATCAAG
TAATATTTGTTTTATTACTGATAAATATAAAGTTAGTAATATACAAAGATGGCGTACTCACCT

>PhCRN07.2_100 PLHAL100A11029
ACCCCCTTTGCCACCAACTACACTACACCGTCACCCCGAACGTCTCAGACGATGGGCTGCGATCAATGCAATGATTCGCCAGAAGAA
TCAAGACGCGAACCAGAAGACGACGACTAAAGACGCCAAAAACACAAACAAGAAGCGGGAAAATCACGATATCGACAAGTCCGTGGG
CTATTCGACTCTTTGCTGGGAAGACATCAAACCTATTTACAACTTCGACTATTCTTACGAACTTCAACCAAGCGATATTCCTGATGC
CGACATCGAGCTACTCCTCGCTCGTATTCGTGACTTGCGGGAACTCTATGGACGAATCTCCGATGGAAATGAAGCCAAGAGATTGTT
TTTTATCGCCCCAATCCTTGAGACAGTCAGTCGTCTGCTCGGGGACGTCCGCATACTCGTCGAAGAAGACGTGGTTGGAAGGTACGT
GCTCCTGAAAGGTCGATTCGAGTTCGTGCTCAAGCGTGGCACCAAGAGAATTTCAATTGTCGAAGCGAAACGTGAAGATATGCTGCA
GGGAGTGGTTCAGAATGTGACGGGGCTGGAAGCGCTGGCTGATGTCGAAGATCTTACTGTGACGTATGGCATTGTGACGAATTTTCT
CGAGTGGAAGTTCTTGATCAGTGGCGACGAGAAGGTCCGAGAGCATGAGACTGTTCTTCATCAAGCGAATACCATACCGTCGTTTGA
AGGATTGAAAGATATCGTCGGGAAGATTTAC
>PhCRN07.2_334 PLHAL3341142
ACCCCCTTTGCCACCAACTACACTACACCGTCACCCCGAACGTCTCAGACGATGGGCTGCGATCAATGCAATGATTCGCCAGAAGAA
TCAAGACGCGAACCAGAAGACGACGACTAAAGACGCCAAAAACACAAACAAGAAGCGGGAAAATCACGATATCGACAAGTCCGTGGG
CTATTCGACTCTTTGCTGGGAAGACATCAAACCTATTTACAACTTCGACTATTCTTACGAACTTCAACCAAGCGATATTCCTGATGC
CGACATCGAGCTACTCCTCGCTCGTATTCGTGACTTGCGGGAACTCTATGGACGAATCTCCGATGGAAATGAAGCCAAGAGATTGTT
TTTTATCGCCCCAATCCTTGAGACAGTCAGTCGTCTGCTCGGGGACGTCCGCATACTCGTCGAAGAAGACGTGGTTGGAAGGTACGT
GCTCCTGAAAGGTCGATTCGAGTTCGTGCTCAAGCGTGGCACCAAGAGAATTT
>PhCRN07.2_703 PLHAL7031064
ACCCCCTTTGCCACCAACTACACTACACCGTCACCCCGAACGTCTCAGACGATGGGCTGCGATCAATGCAATGATTCGCCAGAAGAA
TCAAGACGCGAACCAGAAGACGACGACTAAAGACGCCAAAAACACAAACAAGAAGCGGGAAAATCACGATATCGACAAGTCCGTGGG
CTATTCGACTCTTTGCTGGGAAGACATCAAACCTATTTACAACTTCGACTATTCTTACGAACTTCAACCAAGCGATATTCCTGATGC
CGACATCGAGCTACTCCTCGCTCGTATTCGTGACTTGCGGGAACTCTATGGACGAATCTCCGATGGAAATGAAGCCAAGAGATTGTT
TTTTATCGCCCCAATCCTTGAGACAGTCAGTCTCAAGGATTGGCAGTCAGTCGTCTGCTCGGGGACGTCCGCATACTCGTCGAAGAA
GACGTGGTTGGAAGGTACGTGCTCCTGAAAGGTCGATTCGAGTTCGTGCTCAAGCGTGGCACCAAGAGAATTTCAATTGTCCAAGCG
AAACGTGAAGATATGCTGCAGGGAGTGGTTCAGAATGTGACGGGGCTGGAAGCGCTGGCTGATGTCGAAGATCTTACTGTGACGTAT
GGCATTGTGACGAATTTTCTCGAGTGGAAGTTCTTGATCAGTGGCGACGAGAAGGTCCGAGAGCATGAGACTGTTCTTCATCAAGCG
AATACCATACCGTCGTTTGAAGGATTGAAAGATATCGTCGGGAAGATTTAC
>PhCRN07.2_710 PLHAL7100977
ACCCCCTTTGCCACCAACTACACTACACCGTCACCCCGAACGTCTCAGACGATGGGCTGCGATCAATGCAATGATTCGCCAGAAGAA
TCAAGACGCGAACCAGAAGACGACGACTAAAGACGCCAAAAACACAAACAAGAAGCGGGAAAATCACGATATCGACAAGTCCGTGGG
CTATTCGACTCTTTGCTGGGAAGACATCAAACCTATTTACAACTTCGACTATTCTTACGAACTTCAACCAAGCGATATTCCTGATGC
CGACATCGAGCTACTCCTCGCTCGTATTCGTGACTTGCGGGAACTCTATGGACGAATCTCCGATGGAAATGAAGCCAAGAGATTGTT

TTTTATCGCCCCAATCCTTGAGACAGTCAGTCGTCTGCTCGGGGACGTCCGCATACTCGTCGAAGAAGACGTGGTTGGAAGGTACGT
GCTCCTGAAAGGTCGATTCGAGTTCGTGCTCAAGCGTGGCACCAAGAGAATTTCAATTGTCCAAGCGAAACGTGAAGATATGCTGCA
GGGAGTGGTTCAGAATGTGACGGGGCTGGAAGCGCTGGCTGATGTCGAAGATCTTACTGTGACGTATGGCATTGTGACGAATTTTCT
CGAGTGGAAGTTCTTGATCAGTGGCGACGAGAAGGTCCGAGAGCATGAGACTGTTCTTCATCAAGCGAATACCATACCGTCGTTTGA
AGGATTGAAAGATATCGTCGGGAAGATTTAC
>PhCRN07.2_304 PLHAL3041074
ACCCCCTTTGCCACCAACTACACTACACCGTCACCCCGAACGTCTCAGACGATGGGCTGCGATCAATGCAATGATTCGCCAGAAGAA
TCAAGACGCGAACCAGAAGACGACGACTAAAGACGCCAAAAACACAAACAAGAAGCGGGAAAATCACGATATCGACAAGTCCGTGGG
CTATTCGACTCTTTGCTGGGAAGACATCAAACCTATTTACAACTTCGACTATTCTTACGAACTTCAACCAAGCGATATTCCTGATGC
CGACATCGAGCTACTCCTCGCTCGTATTCGTGACTTGCGGGAACTCTATGGACGAATCTCCGATGGAAATGAAGCCAAGAGATTGTT
TTTTATCGCCCCAATCCTTGAGACAGTCAGTCGTCTGCTCGGGGACGTCCGCATACTCGTCGAAGAAGACGTGGTTGGAAGGTACGT
GCTCCTGAAAGGTCGATTCGAGTTCGTGCTCAAGCGTGGCACCAAGAGAATTTCAATTGTCGAAGCGAAACGTGAAGATATGCTGCA
GGGAGTGGTTCAGAATGTGACGGGGCTGGAAGCGCTGGCTGATGTCGAAGATCTTACTGTGACGTATGGCATTGTGACGAATTTTCT
CGAGTGGAAGTTCTTGATCAGTGGCGACGAGAAGGTCCGAGAGCATGAGACTGTTCTTCATCAAGCGAATACCATACCGTCGTTTGA
AGGATTGAAAGATATCGTCGGGAAGATTTAC
>PhCRN07.2_700 PLHAL7001355
ACCCCCTTTGCCACCAACTACACTACACCGTCACCCCGAACGTCTCAGACGATGGGCTGCGATCAATGCAATGATTCGCCAGAAGAA
TCAAGACGCGAACCAGAAGACGACGACTAAAGACGCCAAAAACACAAACAAGAAGCGGGAAAATCACGATATCGACAAGTCCGTGGG
CTATTCGACTCTTTGCTGGGAAGACATCAAACCTATTTACAACTTCGACTATTCTTACGAACTTCAACCAAGCGATATTCCTGATGC
CGACATCGAGCTACTCCTCGCTCGTATTCGTGACTTGCGGGAACTCTATGGACGAATCTCCGATGGAAATGAAGCCAAGAGATTGTT
TTTTATCGCCCCAATCCTTGAGACAGTCAGTCTCAAGGATTGGCAGTCAGTCGTCTGCTCGGGGACGTCCGCATACTCGTCGAAGAA
GACGTGGTTGGAAGGTACGTGCTCCTGAAAGGTCGATTCGAGTTCGTGCTCAAGCGTGGCACCAAGAGAATTT
>PhCRN07.2_730 PLHAL7301096
ACCCCCTTTGCCACCAACTACACTACACCGTCACCCCGAACGTCTCAGACGATGGGCTGCGATCAATGCAATGATTCGCCAGAAGAA
TCAAGACGCGAACCAGAAGACGACGACTAAAGACGCCAAAAACACAAACAAGAAGCGGGAAAATCACGATATCGACAAGTCCGTGGG
CTATTCGACTCTTTGCTGGGAAGACATCAAACCTATTTACAACTTCGACTATTCTTACGAACTTCAACCAAGCGATATTCCTGATGC
CGACATCGAGCTACTCCTCGCTCGTATTCGTGACTTGCGGGAACTCTATGGACGAATCTCCGATGGAAATGAAGCCAAGAGATTGTT
TTTTATCGCCCCAATCCTTGAGACAGTCAGTCGTCTGCTCGGGGACGTCCGCATACTCGTCGAAGAAGACGTGGTTGGAAGGTACGT
GCTCCTGAAAGGTCGATTCGAGTTCGTGCTCAAGCGTGGCACCAAGAGAATTTCAATTGTCCAAGCGAAACGTGAAGATATGCTGCA
GGGAGTGGTTCAGAATGTGACGGGGCTGGAAGCGCTGGCTGATGTCGAAGATCTTACTGTGACGTATGGCATTGTGACGAATTTTCT
CGAGTGGAAGTTCTTGATCAGTGGCGACGAGAAGGTCCGAGAGCATGAGACTGTTCTTCATCAAGCGAATACCATACCGTCGTTTGA
AGGATTGAAAGATATCGTCGGGAAGATTTAC

>PhCRN07.3_100 PLHAL100A12424
CGGCGTGAGACCCCCTTTGCCACCAACTACACTACACCGTCACGTCTCAGACGATGGGCTGCGATCAATGCAATGATTCGCCAGAAG
AATCAAGACGCGAACCAGAAGACGACGACTGAAGACGCCAAGAACACAAACAAGAAGCGGAAAAAACACGATATCGACAAGTCCGTG
GGCTATTCGACTCTTTGTTGGGAAGACATCAAACCTATTTACAACTTCGACGATTCTTACGAACTTCAACCAAGCGATATTCCTGAT
GCCGACATCGAGCTACTCCTCGCTCGTATTCGTGATTTGCGGGAACTCTATGGAAAAATCTCCAATGGAAAAGAAGCCAAGCGATTG
ATTTTTATCGCCCCAATCCTTGAGACAGTCAGTCGTCTGCTCGGGGACTTCCGCATACTCGTCGAAGAAGACGTGGTTGAAAGGTAC
GTGCTCCTGAAAGGTCGATTCGAGTTCGTGCTCAAGCGTGGCACCAAGAGAATTTCAATTGTCGAAGCGAAACGTGAAGATATGCTG
CAGGGAGTGGTTCAGAATGTGACGGGGCTGGAAGCGCTGGCTGATGTCGAAGATCTTACTGTGACGTATGGCATTGTGACGAATTTT
CTCGAGTGGAAGTCTTGATCAGTGGCGACGAGAAGGTCCGAGAGCATGAGACTGTTCTTTCTCAAGCGAATACCATACCGTCGTTTG
AAGGATTGAAAGAGATCGTCGGGAAGATTTA
>PhCRN07.3_334 PLHAL3342569
ACCCCCTTTGCCACCAACTACACTACACCGTCACGTCTCAGACGATGGGCTGCGATCAATGCAATGATTCGCCAGAAGAATCAAGAC
GCGAACCAGAAGACGACGACTGAAGACGCCAAGAACACAAACAAGAAGCGGAAAAAACACGATATCGACAAGTCCGTGGGCTATTCG
ACTCTTTGTTGGGAAGACATCAAACCTATTTACAACTTCGACGATTCTTACGAACTTCAACCAAGCGATATTCCTGATGCCGACATC
GAGCTACTCCTCGCTCGTATTCGTGATTTGCGGGAACTCTATGGAAAAATCTCCAATGGAAAAGAAGCCAAGCGATTGATTTTTATC
GCCCCAATCCTTGAGACAGTCAGTCGTCTGCTCGGGGACTTCCGCATACTCGTCGAAGAAGACGTGGTTGAAAGGTACGTGCTCCTG
AAAGGTCGATTCGAGTTCGTGCTCAAGCGTGGCACCAAGAGAATTT
>PhCRN07.3_703 PLHAL7032169
CGGCGTGAGACCCCCTTTGCCACCAACTACACTACACCGTCACGTCTCAGACGATGGGCTGCGATCAATGCAATGATTCGCCAGAAG
AATCAAGACGCGAACCAGAAGACGACGACTGAAGACGCCAAGAACACAAACAAGAAGCGGAAAAAACACGATATCGACAAGTCCGTG
GGCTATTCGACTCTTTGTTGGGAAGACATCAAACCTATTTACAACTTCGACGATTCTTACGAACTTCAACCAAGCGATATTCCTGAT
GCCGACATCGAGCTACTCCTCGCTCGTATTCGTGATTTGCGGGAACTCTATGGAAAAATCTCCAATGGAAAAGAAGCCAAGCGATTG
ATTTTTATCGCCCCAATCCTTGAGACAGTCAGTCGTCTGCTCGGGGACTTCCGCATACTCGTCGAAGAAGACGTGGTTGAAAGGTAC
GTGCTCCTGAAAGGTCGATTCGAGTTCGTGCTCAAGCGTGGCACCAAGAGAATTTCAATTGTCGAAGCGAAACGTGAAGATATGCTG
CAGGGAGTGGTTCAGAATGTGACGGGGCTGGAAGCGCTGGCTGATGTCGAAGATCTTACTGTGACGTATGGCATTGTGACGAATTTT
CTCGAGTGGAAGTCTTGATCAGTGGCGACGAGAAGGTCCGAGAGCATGAGACTGTTCTTTCTCAAGCGAATACCATACCGTCGTTTG
AAGGATTGAAAGAGATCGTCGGGAAGATTTA
>PhCRN07.3_710 PLHAL7101755
CGGCGTGAGACCCCCTTTGCCACCAACTACACTACACCGTCACGTCTCAGACGATGGGCTGCGATCAATGCAATGATTCGCCAGAAG
AATCAAGACGCGAACCAGAAGACGACGACTGAAGACGCCAAGAACACAAACAAGAAGCGGAAAAAACACGATATCGACAAGTCCGTG
GGCTATTCGACTCTTTGTTGGGAAGACATCAAACCTATTTACAACTTCGACGATTCTTACGAACTTCAACCAAGCGATATTCCTGAT
GCCGACATCGAGCTACTCCTCGCTCGTATTCGTGATTTGCGGGAACTCTATGGAAAAATCTCCAATGGAAAAGAAGCCAAGCGATTG
ATTTTTATCGCCCCAATCCTTGAGACAGTCAGTCGTCTGCTCGGGGACTTCCGCATACTCGTCGAAGAAGACGTGGTTGAAAGGTAC
GTGCTCCTGAAAGGTCGATTCGAGTTCGTGCTCAAGCGTGGCACCAAGAGAATTTCAATTGTCGAAGCGAAACGTGAAGATATGCTG

CAGGGAGTGGTTCAGAATGTGACGGGGCTGGAAGCGCTGGCTGATGTCGAAGATCTTACTGTGACGTATGGCATTGTGACGAATTTT
CTCGAGTGGAAGTCTTGATCAGTGGCGACGAGAAGGTCCGAGAGCATGAGACTGTTCTTTCTCAAGCGAATACCATACCGTCGTTTG
AAGGATTGAAAGAGATCGTCGGGAAGATTTA
>PhCRN07.3_304 PLHAL3042873
CGGCGTGAGACCCCCTTTGCCACCAACTACACTACACCGTCACGTCTCAGACGATGGGCTGCGATCAATGCAATGATTCGCCAGAAG
AATCAAGACGCGAACCAGAAGACGACGACTGAAGACGCCAAGAACACAAACAAGAAGCGGAAAAAACACGATATCGACAAGTCCGTG
GGCTATTCGACTCTTTGTTGGGAAGACATCAAACCTATTTACAACTTCGACGATTCTTACGAACTTCAACCAAGCGATATTCCTGAT
GCCGACATCGAGCTACTCCTCGCTCGTATTCGTGATTTGCGGGAACTCTATGGAAAAATCTCCAATGGAAAAGAAGCCAAGCGATTG
ATTTTTATCGCCCCAATCCTTGAGACAGTCAGTCGTCTGCTCGGGGACTTCCGCATACTCGTCGAAGAAGACGTGGTTGAAAGGTAC
GTGCTCCTGAAAGGTCGATTCGAGTTCGTGCTCAAGCGTGGCACCAAGAGAATTTCAATTGTCGAAGCGAAACGTGAAGATATGCTG
CAGGGAGTGGTTCAGAATGTGACGGGGCTGGAAGCGCTGGCTGATGTCGAAGATCTTACTGTGACGTATGGCATTGTGACGAATTTT
CTCGAGTGGAAGTCTTGATCAGTGGCGACGAGAAGGTCCGAGAGCATGAGACTGTTCTTTCTCAAGCGAATACCATACCGTCGTTTG
AAGGATTGAAAGAGATCGTCGGGAAGATTTA
>PhCRN07.3_700 PLHAL7002819
CGGCGTGAGACCCCCTTTGCCACCAACTACACTACACCGTCACGTCTCAGACGATGGGCTGCGATCAATGCAATGATTCGCCAGAAG
AATCAAGACGCGAACCAGAAGACGACGACTGAAGACGCCAAGAACACAAACAAGAAGCGGAAAAAACACGATATCGACAAGTCCGTG
GGCTATTCGACTCTTTGTTGGGAAGACATCAAACCTATTTACAACTTCGACGATTCTTACGAACTTCAACCAAGCGATATTCCTGAT
GCCGACATCGAGCTACTCCTCGCTCGTATTCGTGATTTGCGGGAACTCTATGGAAAAATCTCCAATGGAAAAGAAGCCAAGCGATTG
ATTTTTATCGCCCCAATCCTTGAGACAGTCAGTCGTCTGCTCGGGGACTTCCGCATACTCGTCGAAGAAGACGTGGTTGAAAGGTAC
GTGCTCCTGAAAGGTCGATTCGAGTTCGTGCTCAAGCGTGGCACCAAGAGAATTTCAATTGTCGAAGCGAAACGTGAAGATATGCTG
CAGGGAGTGGTTCAGAATGTGACGGGGCTGGAAGCGCTGGCTGATGTCGAAGATCTTACTGTGACGTATGGCATTGTGACGAATTTT
CTCGAGTGGAAGTTCTTGATCAGTGGCGACGAGAAGGTCCGAGAGCATGAGACTGTTCTTCATCAAGCGAATACCATACCGTCGTTT
GAAGGATTGAAAGATATCGTCGGGAAGATTTA
>PhCRN07.3_730 PLHAL7302106
CGGCGTGAGACCCCCTTTGCCACCAACTACACTACACCGTCACGTCTCAGACGATGGGCTGCGATCAATGCAATGATTCGCCAGAAG
AATCAAGACGCGAACCAGAAGACGACGACTGAAGACGCCAAGAACACAAACAAGAAGCGGAAAAAACACGATATCGACAAGTCCGTG
GGCTATTCGACTCTTTGTTGGGAAGACATCAAACCTATTTACAACTTCGACGATTCTTACGAACTTCAACCAAGCGATATTCCTGAT
GCCGACATCGAGCTACTCCTCGCTCGTATTCGTGATTTGCGGGAACTCTATGGAAAAATCTCCAATGGAAAAGAAGCCAAGCGATTG
ATTTTTATCGCCCCAATCCTTGAGACAGTCAGTCGTCTGCTCGGGGACTTCCGCATACTCGTCGAAGAAGACGTGGTTGAAAGGTAC
GTGCTCCTGAAAGGTCGATTCGAGTTCGTGCTCAAGCGTGGCACCAAGAGAATTTCAATTGTCCAAGCGAAACGTGAAGATATGCTG
CAGGGAGTGGTTCAGAATGTGACGGGGCTGGAAGCGCTGGCTGATGTCGAAGATCTTACTGTGACGTATGGCATTGTGACGAATTTT
CTCGAGTGGAAGTCTTGATCAGTGGCGACGAGAAGGTCCGAGAGCATGAGACTGTTCTTTCTCAAGCGAATACCATACCGTCGTTTG
AAGGATTGAAAGAGATCGTCGGGAAGATTTA

>PhCRN10.1_100 PLHAL100A10987
AGAGTAACGAGTGATCGATATTAGTAGAATTTTAAAACTGTTTAACTAACAATTGCTATTTTGCTATTTGACAATGCAACCTCTCGA
TTATGAATGAGCAACGTCTTTTCGATATGATAGCGTTTCGGCCGCCAACCTCAAGGTAAATTGAACAGATTTGACCTCTCGGATCAG
CGTCTCACGGTTAGTGGCAAGATGTCTAAAACTATCATACCAGCTCTAGTAAACCAGAAATTCTCGCTCCCTTGGAGCTCGTTCCAG
AATATACTTGCTCGAGTAAAAATAGTGAAGCTAATCTGTGCGATCGTTGGTGTGGAAGGTAATGCGTTTCCTGTGGACATTGATGCG
GATCAGCTGGTGGGAGACCTCAAGAAGGCAAGAATGGCGAAAAAACCAAAGGGAATTGACGCAGACAAGTTGAAACTTTTTTTGGCC
AAGACGGAGGGTGGCGCGTGGCTAGATGAGGCTGGCGTAGCTTTTGTGGCGCTTGATGAACGTGGACTCCTGCAAGACTACATGAAG
ATGAATCCCATGCTCTTTGGAGATAATTTTTAACTAGGTGAAGACCAAATTCACGTGCTGGTAGTGGTTCCGGTACGTGCAGTGGAG
TATGGCACATGGATGCCGATTGACTTGTCTACCTTGGATCCTCAAGTTCAATTAAAAATGTGGAAGTCAGTTGTCAGAGTTTTTTCA
GAAGTTGAGTGTTCAGGAACAGCAGTGGTTGTGGATGAAACGTCGACGCACTTGTATCTGCTGACGAACTTGTACATGTGGGTCGAC
AAAACATTCACTGATCATCTGAGTGCTGAATTCAGGAAAGAAGTCAATCGGTATCTGAGATGCCACCCCCAAATGAAAACATGTTAT
GGACGCTAATGCTGCAAAGCAACCACGTCTTCACGGACGGCAGCGATGC
>PhCRN10.1_334 PLHAL3341571
AGAGTAACGAGTGATCGATATTAGTAGAATTTTAAAACTGTTTAACTAACAATTGCTATTTTGCTATTTGACAATGCAACCTCTCGA
TTATGAATGAGCAACGTCTTTTCGATATGATAGCGTTTCGGCCGCCAACCTCAAGGTAAATTGAACAGATTTGACCTCTCGGATCAG
CGTCTCACGGTTAGTGGCAAGATGTCTAAAACTATCATACCAGCTCTAGTAAACCAGAAATTCTCGCTCCCTTGGAGCTCGTTCCAG
AATATACTTGCTCGAGTAAAAATAGTGAAGCTAATCTGTGCGATCGTTGGTGTGGAAGGTAATGCGTTTCCTGTGGACATTGATGCG
GATCAGCTGGTGGGAGACCTCAAGAAGGCAAGAATGGCGAAAAAACCAAAGGGAATTGACGCAGACAAGTTGAAACTTTTTTTGGCC
AAGACGGAGGGTGGCGCGTGGCTAGATGAGGCTGGCGTAGCTTTTGTGGCGCTTGATGAACGTGGACTCCTGCAAGACTACATGAAG
ATGAATCCCATGCTCTTTGGAGATAATTTTTAACTAGGTGAAGACCAAATTCACGTGCTGGTAGTGGTTCCGGTACGTGCAGTGGAG
TATGGCACATGGATGCCGATTGACTTGTCTACCTTGGATCCTCAAGTTCAATTAAAAATGTGGAAGTCAGTTGTCAGAGTTTTTTCA
GAAGTTGAGTGTTCAGGAACAGCAGTGGTTGTGGATGAAACGTCGACGCACTTGTATCTGCTGACGAACTTGTACATGTGGGTCGAC
AAAACATTCACTGATCATCTGAGTGCTGAATTCAGGAAAGAAGTCAATCGGTATCTGAGATGCCACCCCCAAATGAAAACATGTTAT
GGACGCTAATGCTGCAAAGCAACCACGTCTTCACGGACGGCAGCGATGC
>PhCRN10.1_703 PLHAL7030258
AGAGTAACGAGTGATCGATATTAGTAGAATTTTAAAACTGTTTAACTAACAATTGCTATTTTGCTATTTGACAATGCAACCTCTCGA
TTATGAATGAGCAACGTCTTTTCGATATGATAGCGTTTCGGCCGCCAACCTCAAGGTAAATTGAACAGATTTGACCTCTCGGATCAG
CGTCTCACGGTTAGTGGCAAGATGTCTAAAACTATCATACCAGCTCTAGTAAACCAGAAATTCTCGCTCCCTTGGAGCTCGTTCCAG
AATATACTTGCTCGAGTAAAAATAGTGAAGCTAATCTGTGCGATCGTTGGTGTGGAAGGTAATGCGTTTCCTGTGGACATTGATGCG
GATCAGCTGGTGGGAGACCTCAAGAAGGCAAGAATGGCGAAAAAACCAAAGGGAATTGACGCAGACAAGTTGAAACTTTTTTTGGCC
AAGACGGAGGGTGGCGCGTGGCTAGATGAGGCTGGCGTAGCTTTTGTGGCGCTTGATGAACGTGGACTCCTGCAAGACTACATGAAG
ATGAATCCCATGCTCTTTGGAGATAATTTTTAACTAGGTGAAGACCAAATTCACGTGCTGGTAGTGGTTCCGGTACGTGCAGTGGAG
TATGGCACATGGATGCCGATTGACTTGTCTACCTTGGATCCTCAAGTTCAATTAAAAATGTGGAAGTCAGTTGTCAGAGTTTTTTCA

GAAGTTGAGTGTTCAGGAACAGCAGTGGTTGTGGATGAAACGTCGACGCACTTGTATCTGCTGACGAACTTGTACATGTGGGTCGAC
AAAACATTCACTGATCATCTGAGTGCTGAATTCAGGAAAGAAGTCAATCGGTATCTGAGATGCCACCCCCAAATGAAAACATGTTAT
GGACGCTAATGCTGCAAAGCAACCACGTCTTCACGGACGGCAGCGATGC
>PhCRN10.1_710 PLHAL7100233
AGAGTAACGAGTGATCGATATTAGTAGAATTTTAAAACTGTTTAACTAACAATTGCTATTTTGCTATTTGACAATGCAACCTCTCGA
TTATGAATGAGCAACGTCTTTTCGATATGATAGCGTTTCGGCCGCCAACCTCAAGGTAAATTGAACAGATTTGACCTCTCGGATCAG
CGTCTCACGGTTAGTGGCAAGATGTCTAAAACTATCATACCAGCTCTAGTAAACCAGAAATTCTCGCTCCCTTGGAGCTCGTTCCAG
AATATACTTGCTCGAGTAAAAATAGTGAAGCTAATCTGTGCGATCGTTGGTGTGGAAGGTAATGCGTTTCCTGTGGACATTGATGCG
GATCAGCTGGTGGGAGACCTCAAGAAGGCAAGAATGGCGAAAAAACCAAAGGGAATTGACGCAGACAAGTTGAAACTTTTTTTGGCC
AAGACGGAGGGTGGCGCGTGGCTAGATGAGGCTGGCGTAGCTTTTGTGGCGCTTGATGAACGTGGACTCCTGCAAGACTACATGAAG
ATGAATCCCATGCTCTTTGGAGATAATTTTTAACTAGGTGAAGACCAAATTCACGTGCTGGTAGTGGTTCCGGTACGTGCAGTGGAG
TATGGCACATGGATGCCGATTGACTTGTCTACCTTGGATCCTCAAGTTCAATTAAAAATGTGGAAGTCAGTTGTCAGAGTTTTTTCA
GAAGTTGAGTGTTCAGGAACAGCAGTGGTTGTGGATGAAACGTCGACGCACTTGTATCTGCTGACGAACTTGTACATGTGGGTCGAC
AAAACATTCACTGATCATCTGAGTGCTGAATTCAGGAAAGAAGTCAATCGGTATCTGAGATGCCACCCCCAAATGAAAACATGTTAT
GGACGCTAATGCTGCAAAGCAACCACGTCTTCACGGACGGCAGCGATGC
>PhCRN10.1_304 PLHAL3041208
AGAGTAACGAGTGATCGATATTAGTAGAATTTTAAAACTGTTTAACTAACAATTGCTATTTTGCTATTTGACAATGCAACCTCTCGA
TTATGAATGAGCAACGTCTTTTCGATATGATAGCGTTTCGGCCGCCAACCTCAAGGTAAATTGAACAGATTTGACCTCTCGGATCAG
CGTCTCACGGTTAGTGGCAAGATGTCTAAAACTATCATACCAGCTCTAGTAAACCAGAAATTCTCGCTCCCTTGGAGCTCGTTCCAG
AATATACTTGCTCGAGTAAAAATAGTGAAGCTAATCTGTGCGATCGTTGGTGTGGAAGGTAATGCGTTTCCTGTGGACATTGATGCG
GATCAGCTGGTGGGAGACCTCAAGAAGGCAAGAATGGCGAAAAAACCAAAGGGAATTGACGCAGACAAGTTGAAACTTTTTTTGGCC
AAGACGGAGGGTGGCGCGTGGCTAGATGAGGCTGGCGTAGCTTTTGTGGCGCTTGATGAACGTGGACTCCTGCAAGACTACATGAAG
ATGAATCCCATGCTCTTTGGAGATAATTTTTAACTAGGTGAAGACCAAATTCACGTGCTGGTAGTGGTTCCGGTACGTGCAGTGGAG
TATGGCACATGGATGCCGATTGACTTGTCTACCTTGGATCCTCAAGTTCAATTAAAAATGTGGAAGTCAGTTGTCAGAGTTTTTTCA
GAAGTTGAGTGTTCAGGAACAGCAGTGGTTGTGGATGAAACGTCGACGCACTTGTATCTGCTGACGAACTTGTACATGTGGGTCGAC
AAAACATTCACTGATCATCTGAGTGCTGAATTCAGGAAAGAAGTCAATCGGTATCTGAGATGCCACCCCCAAATGAAAACATGTTAT
GGACGCTAATGCTGCAAAGCAACCACGTCTTCACGGACGGCAGCGATGC
>PhCRN10.1_700 PLHAL7003067
AGAGTAACGAGTGATCGATATTAGTAGAATTTTAAAACTGTTTAACTAACAATTGCTATTTTGCTATTTGACAATGCAACCTCTCGA
TTATGAATGAGCAACGTCTTTTCGATATGATAGCGTTTCGGCCGCCAACCTCAAGGTAAATTGAACAGATTTGACCTCTCGGATCAG
CGTCTCACGGTTAGTGGCAAGATGTCTAAAACTATCATACCAGCTCTAGTAAACCAGAAATTCTCGCTCCCTTGGAGCTCGTTCCAG
AATATACTTGCTCGAGTAAAAATAGTGAAGCTAATCTGTGCGATCGTTGGTGTGGAAGGTAATGCGTTTCCTGTGGACATTGATGCG
GATCAGCTGGTGGGAGACCTCAAGAAGGCAAGAATGGCGAAAAAACCAAAGGGAATTGACGCAGACAAGTTGAAACTTTTTTTGGCC
AAGACGGAGGGTGGCGCGTGGCTAGATGAGGCTGGCGTAGCTTTTGTGGCGCTTGATGAACGTGGACTCCTGCAAGACTACATGAAG
ATGAATCCCATGCTCTTTGGAGATAATTTTTAACTAGGTGAAGACCAAATTCACGTGCTGGTAGTGGTTCCGGTACGTGCAGTGGAG
TATGGCACATGGATGCCGATTGACTTGTCTACCTTGGATCCTCAAGTTCAATTAAAAATGTGGAAGTCAGTTGTCAGAGTTTTTTCA
GAAGTTGAGTGTTCAGGAACAGCAGTGGTTGTGGATGAAACGTCGACGCACTTGTATCTGCTGACGAACTTGTACATGTGGGTCGAC
AAAACATTCACTGATCATCTGAGTGCTGAATTCAGGAAAGAAGTCAATCGGTATCTGAGATGCCACCCCCAAATGAAAACATGTTAT
GGACGCTAATGCTGCAAAGCAACCACGTCTTCACGGACGGCAGCGATGC
>PhCRN10.1_730 PLHAL7300176
AGAGTAACGAGTGATCGATATTAGTAGAATTTTAAAACTGTTTAACTAACAATTGCTATTTTGCTATTTGACAATGCAACCTCTCGA
TTATGAATGAGCAACGTCTTTTCGATATGATAGCGTTTCGGCCGCCAACCTCAAGGTAAATTGAACAGATTTGACCTCTCGGATCAG
CGTCTCACGGTTAGTGGCAAGATGTCTAAAACTATCATACCAGCTCTAGTAAACCAGAAATTCTCGCTCCCTTGGAGCTCGTTCCAG
AATATACTTGCTCGAGTAAAAATAGTGAAGCTAATCTGTGCGATCGTTGGTGTGGAAGGTAATGCGTTTCCTGTGGACATTGATGCG
GATCAGCTGGTGGGAGACCTCAAGAAGGCAAGAATGGCGAAAAAACCAAAGGGAATTGACGCAGACAAGTTGAAACTTTTTTTGGCC
AAGACGGAGGGTGGCGCGTGGCTAGATGAGGCTGGCGTAGCTTTTGTGGCGCTTGATGAACGTGGACTCCTGCAAGACTACATGAAG
ATGAATCCCATGCTCTTTGGAGATAATTTTTAACTAGGTGAAGACCAAATTCACGTGCTGGTAGTGGTTCCGGTACGTGCAGTGGAG
TATGGCACATGGATGCCGATTGACTTGTCTACCTTGGATCCTCAAGTTCAATTAAAAATGTGGAAGTCAGTTGTCAGAGTTTTTTCA
GAAGTTGAGTGTTCAGGAACAGCAGTGGTTGTGGATGAAACGTCGACGCACTTGTATCTGCTGACGAACTTGTACATGTGGGTCGAC
AAAACATTCACTGATCATCTGAGTGCTGAATTCAGGAAAGAAGTCAATCGGTATCTGAGATGCCACCCCCAAATGAAAACATGTTAT
GGACGCTAATGCTGCAAAGCAACCACGTCTTCACGGACGGCAGCGATGC

>PhCRN10.2_100 PLHAL100A13001
AATTTTAAAATTGTTTATCTAAAACTTGCTATTTTGCTATTTGACGATGCAACCTCTCGATTATGAATGAGCAACGTCGTTTCGATA
TGATAGCGTTTCGGCCGCCAACTTCAAGGTAAATTGAACAGATTTGACCTCTCGGATCAGCGTCTTATGGTTAGTTGCAAAATCTAT
GTCTAAAACCATCATGTCCGCTCTAGTACACCAGGAATTCTCCTTCCCTTGTAGCTCATTCCAGAATATACCTGCTCGAGTAAAAAT
GGTGAAGCTAATCTGTGCGATCGTTGGTGTGGCAGGTAATGCGTTTCCTGTGGACATCGACGCGGGTCAGTTGGTGGGAGACCTCAA
GAAGGCAATAATGGCGAAAAAAACGAGGGAATCGACGCAGACAAGTCGAAACTTTTTTTGGCCAAGACGGAGGGTGGCGCGTGGCTA
GATGAGGCTGGCGTAG
>PhCRN10.2_334 PLHAL3343415
AATTTTAAAATTGTTTATCTAAAACTTGCTATTTTGCTATTTGACGATGCAACCTCTCGATTATGAATGAGCAACGTCGTTTCGATA
TGATAGCGTTTCGGCCGCCAACTTCAAGGTAAATTGAACAGATTTGACCTCTCGGATCAGCGTCTTATGGTTAGTTGCAAAATCTAT
GTCTAAAACCATCATGTCCGCTCTAGTACACCAGGAATTCTCCTTCCCTTGTAGCTCATTCCAGAATATACCTGCTCGAGTAAAAAT
GGTGAAGCTAATCTGTGCGATCGTTGGTGTGGCAGGTAATGCGTTTCCTGTGGACATCGACGCGGGTCAGTTGGTGGGAGACCTCAA
GAAGGCAATAATGGCGAAAAAAACGAGGGAATCGACGCAGACAAGTCGAAACTTTTTTTGGCCAAGACGGAGGGTGGCGCGTGGCTA
GATGAGGCTGGCGTAG
>PhCRN10.2_703 PLHAL7032298

AATTTTAAAATTGTTTATCTAAAACTTGCTATTTTGCTATTTGACGATGCAACCTCTCGATTATGAATGAGCAACGTCGTTTCGATA
TGATAGCGTTTCGGCCGCCAACTTCAAGGTAAATTGAACAGATTTGACCTCTCGGATCAGCGTCTTATGGTTAGTTGCAAAATCTAT
GTCTAAAACCATCATGTCCGCTCTAGTACACCAGGAATTCTCCTTCCCTTGTAGCTCATTCCAGAATATACCTGCTCGAGTAAAAAT
GGTGAAGCTAATCTGTGCGATCGTTGGTGTGGCAGGTAATGCGTTTCCTGTGGACATCGACGCGGGTCAGTTGGTGGGAGACCTCAA
GAAGGCAATAATGGCGAAAAAAACGAGGGAATCGACGCAGACAAGTCGAAACTTTTTTTGGCCAAGACGGAGGGTGGCGCGTGGCTA
GATGAGGCTGGCGTAG
>PhCRN10.2_710 PLHAL7102079
AATTTTAAAATTGTTTATCTAAAACTTGCTATTTTGCTATTTGACGATGCAACCTCTCGATTATGAATGAGCAACGTCGTTTCGATA
TGATAGCGTTTCGGCCGCCAACTTCAAGGTAAATTGAACAGATTTGACCTCTCGGATCAGCGTCTTATGGTTAGTTGCAAAATCTAT
GTCTAAAACCATCATGTCCGCTCTAGTACACCAGGAATTCTCCTTCCCTTGTAGCTCATTCCAGAATATACCTGCTCGAGTAAAAAT
GGTGAAGCTAATCTGTGCGATCGTTGGTGTGGCAGGTAATGCGTTTCCTGTGGACATCGACGCGGGTCAGTTGGTGGGAGACCTCAA
GAAGGCAATAATGGCGAAAAAAACGAGGGAATCGACGCAGACAAGTCGAAACTTTTTTTGGCCAAGACGGAGGGTGGCGCGTGGCTA
GATGAGGCTGGCGTAG
>PhCRN10.2_304 PLHAL3042989
AATTTTAAAATTGTTTATCTAAAACTTGCTATTTTGCTATTTGACGATGCAACCTCTCGATTATGAATGAGCAACGTCGTTTCGATA
TGATAGCGTTTCGGCCGCCAACTTCAAGGTAAATTGAACAGATTTGACCTCTCGGATCAGCGTCTTATGGTTAGTTGCAAAATCTAT
GTCTAAAACCATCATGTCCGCTCTAGTACACCAGGAATTCTCCTTCCCTTGTAGCTCATTCCAGAATATACCTGCTCGAGTAAAAAT
GGTGAAGCTAATCTGTGCGATCGTTGGTGTGGCAGGTAATGCGTTTCCTGTGGACATCGACGCGGGTCAGTTGGTGGGAGACCTCAA
GAAGGCAATAATGGCGAAAAAAACGAGGGAATCGACGCAGACAAGTCGAAACTTTTTTTGGCCAAGACGGAGGGTGGCGCGTGGCTA
GATGAGGCTGGCGTAG
>PhCRN10.2_700 PLHAL7004054
AATTTTAAAATTGTTTATCTAAAACTTGCTATTTTGCTATTTGACGATGCAACCTCTCGATTATGAATGAGCAACGTCGTTTCGATA
TGATAGCGTTTCGGCCGCCAACTTCAAGGTAAATTGAACAGATTTGACCTCTCGGATCAGCGTCTTATGGTTAGTTGCAAAATCTAT
GTCTAAAACCATCATGTCCGCTCTAGTACACCAGGAATTCTCCTTCCCTTGTAGCTCATTCCAGAATATACCTGCTCGAGTAAAAAT
GGTGAAGCTAATCTGTGCGATCGTTGGTGTGGCAGGTAATGCGTTTCCTGTGGACATCGACGCGGGTCAGTTGGTGGGAGACCTCAA
GAAGGCAATAATGGCGAAAAGAACGAGGGGATCGACGCAGACAAGTCGAAACTTTTTTTGGCCAAGACGGAGGGTGGCGCGTGGCTA
GATGAGGCTGGCGTAG
>PhCRN10.2_730 PLHAL7303337
AATTTTAAAATTGTTTATCTAAAACTTGCTATTTTGCTATTTGACGATGCAACCTCTCGATTATGAATGAGCAACGTCGTTTCGATA
TGATAGCGTTTCGGCCGCCAACTTCAAGGTAAATTGAACAGATTTGACCTCTCGGATCAGCGTCTTATGGTTAGTTGCAAAATCTAT
GTCTAAAACCATCATGTCCGCTCTAGTACACCAGGAATTCTCCTTCCCTTGTAGCTCATTCCAGAATATACCTGCTCGAGTAAAAAT
GGTGAAGCTAATCTGTGCGATCGTTGGTGTGGCAGGTAATGCGTTTCCTGTGGACATCGACGCGGGTCAGTTGGTGGGAGACCTCAA
GAAGGCAATAATGGCGAAAAAAACGAGGGAATCGACGCAGACAAGTCGAAACTTTTTTTGGCCAAGACGGAGGGTGGCGCGTGGCTA
GATGAGGCTGGCGTAG

>PhCRN12_100 PLHAL100A10499_RC
GAACAGATTTGACCTCTCGGATCAGCGTCTTATGGTCAGTTGCAAAAACTATGTCCAAAA
CCATATGTCCGCTCTAGTACACCAGGAATTCTCCTTCCCTTGTAGCTCATTCCAGAATAT
ACTTGCTCGAGTAAAAAATGGTGAAGCTAATCTATGCGATCGTTGGTGCGGCAAGTAATG
CGTTCCCTGTGGACATCGACGCGGGTCAGTTGGTGGGAGACCTCAAGAAGGCAATAATGG
CGAAAAGAACGAGGGGATCGACGCAGACAAGTCGAAACTTTTTTTGGCCAAGACGGAGGG
TGGCGCGTGGCTAGATGAGGCTGGCGTAGCTTTTGTGGCGCTCGATGAACCGTGGACTCC
TGCAAGACTACGTACAGATGAAATCCACACTGTGGATCAAGAATCCCATTTTCTTTGGAG
ATGATT
>PhCRN12_334 PLHAL3340491_RC
GAACAGATTTGACCTCTCGGATCAGCGTCTTATGGTCAGTTGCAAAAACTATGTCCAAAA
CCATATGTCCGCTCTAGTACACCAGGAATTCTCCTTCCCTTGTAGCTCATTCCAGAATAT
ACTTGCTCGAGTAAAAAATGGTGAAGCTAATCTATGCGATCGTTGGTGCGGCAAGTAATG
CGTTCCCTGTGGACATCGACGCGGGTCAGTTGGTGGGAGACCTCAAGAAGGCAATAATGG
CGAAAAGAACGAGGGGATCGACGCAGACAAGTCGAAACTTTTTTTGGCCAAGACGGAGGG
TGGCGCGTGGCTAGATGAGGCTGGCGTAGCTTTTGTGGCGCTCGATGAACCGTGGACTCC
TGCAAGACTACGTACAGATGAAATCCACACTGTGGATCAAGAATCCCATTTTCTTTGGAG
ATGATT
>PhCRN12_703 PLHAL7030571_RC
GAACAGATTTGACCTCTCGGATCAGCGTCTTATGGTCAGTTGCAAAAACTATGTCCAAAA
CCATATGTCCGCTCTAGTACACCAGGAATTCTCCTTCCCTTGTAACTCATTCCAGAATAT
ACTTGCTCGAGTAAAAAATGGCGAAGCTAATCTATGCGATCGTTGGTGCGGCAAGTAATG
CGTTCCCTGTGGACATCGACGCGGGTCAGTTGGTGGGAGACCTCAAGAAGGCAATAATGG
CGAAAAGAACGAGGGGATCGACGCAGACAAGTCGAAACTTTTTTTGGCCAAGACGGAGGG
TGGCGCGTGGCTAGATGAGGCTGGCGTAGCTTTTGTGGCGCTCGATGAACCGTGGACTCC
TGCAAGACTACGTACAGATGAAATCCACACTGTGGATCAAGAATCCCATTTTCTTTGGAG
ATGATT
>PhCRN12_710 PLHAL7100194_RC
GAACAGATTTGACCTCTCGGATCAGCGTCTTATGGTCAGTTGCAAAAACTATGTCCAAAA
CCATATGTCCGCTCTAGTACACCAGGAATTCTCCTTCCCTTGTAGCTCATTCCAGAATAT
ACTTGCTCGAGTAAAAAATGGTGAAGCTAATCTATGCGATCGTTGGTGCGGCAAGTAATG
CGTTCCCTGTGGACATCGACGCGGGTCAGTTGGTGGGAGACCTCAAGAAGGCAATAATGG

CGAAAAGAACGAGGGGATCGACGCAGACAAGTCGAAACTTTTTTTGGCCAAGACGGAGGG
TGGCGCGTGGCTAGATGAGGCTGGCGTAGCTTTTGTGGCGCTCGATGAACCGTGGACTCC
TGCAAGACTACGTACAGATGAAATCCACACTGTGGATCAAGAATCCCATTTTCTTTGGAG
ATGATT
>PhCRN12_304 PLHAL3040530_RC
GAACAGATTTGACCTCTCGGATCAGCGTCTTATGGTCAGTTGCAAAAACTATGTCCAAAA
CCATATGTCCGCTCTAGTACACCAGGAATTCTCCTTCCCTTGTAGCTCATTCCAGAATAT
ACTTGCTCGAGTAAAAAATGGTGAAGCTAATCTATGCGATCGTTGGTGCGGCAAGTAATG
CGTTCCCTGTGGACATCGACGCGGGTCAGTTGGTGGGAGACCTCAAGAAGGCAATAATGG
CGAAAAGAACGAGGGGATCGACGCAGACAAGTCGAAACTTTTTTTGGCCAAGACGGAGGG
TGGCGCGTGGCTAGATGAGGCTGGCGTAGCTTTTGTGGCGCTCGATGAACCGTGGACTCC
TGCAAGACTACGTACAGATGAAATCCACACTGTGGATCAAGAATCCCATTTTCTTTGGAG
ATGATT
>PhCRN12_700 PLHAL7000693_RC
GAACAGATTTGACCTCTCGGATCAGCGTCTTATGGTCAGTTGCAAAAACTATGTCCAAAA
CCATATGTCCGCTCTAGTACACCAGGAATTCTCCTTCCCTTGTAACTCATTCCAGAATAT
ACTTGCTCGAGTAAAAAATGGCGAAGCTAATCTATGCGATCGTTGGTGCGGCAAGTAATG
CGTTCCCTGTGGACATCGACGCGGGTCAGTTGGTGGGAGACCTCAAGAAGGCAATAATGG
CGAAAAGAACGAGGGGATCGACGCAGACAAGTCGAAACTTTTTTTGGCCAAGACGGAGGG
TGGCGCGTGGCTAGATGAGGCTGGCGTAGCTTTTGTGGCGCTCGATGAACCGTGGACTCC
TGCAAGACTACGTACAGATGAAATCCACACTGTGGATCAAGAATCCCATTTTCTTTGGAG
ATGATT
>PhCRN12_730 PLHAL7300137_RC
GAACAGATTTGACCTCTCGGATCAGCGTCTTATGGTCAGTTGCAAAAACTATGTCCAAAA
CCATATGTCCGCTCTAGTACACCAGGAATTCTCCTTCCCTTGTAACTCATTCCAGAATAT
ACTTGCTCGAGTAAAAAATGGCGAAGCTAATCTATGCGATCGTTGGTGCGGCAAGTAATG
CGTTCCCTGTGGACATCGACGCGGGTCAGTTGGTGGGAGACCTCAAGAAGGCAATAATGG
CGAAAAGAACGAGGGGATCGACGCAGACAAGTCGAAACTTTTTTTGGCCAAGACGGAGGG
TGGCGCGTGGCTAGATGAGGCTGGCGTAGCTTTTGTGGCGCTCGATGAACCGTGGACTCC
TGCAAGACTACGTACAGATGAAATCCACACTGTGGATCAAGAATCCCATTTTCTTTGGAG
ATGATT

>PhCRN17.1_100 PLHAL100A11882
TTCCTAGCCAAGACAGAGGGCGGCGCTTGGTTGTCAAGCCAATCAGACGACGTGAAGAAGCTCAAGAAGGGCGAGAAGACAGCTCTC
ATCGAATCGCTAATAAAGGAGGATCAACAGCTACAGGCGGAGGACCCGCTGGAATACGTGCTGAGAGAAAACAGTATGGTCACCCCA
CAGTTAACACAGATTCACGTGCTGGTGCTGGTTCCTGGACAACGCCTATCCCTAGGTGCCGCCGCTCTTCGTGAACCCCATCCCGTC
CGCAAGAAAAGGTGGAAAGAACTGAACGAAGTACTTGATCGGAACAAAAGGTCGAAGGTCAACTCTGCTGGTGAGTCCTCCACAGGG
TACTCATACGTGTCGTTCGCCGATGTCGACAAAATAATGAGGACGCGTCGCTACGAGCAGCCATCGAAAGTCGTCGAAAATGACAAA
CTTGACATGCTCTATGCGTATCTCCTGCTCCTACCTAAAGCATTCGGAGACATCGTGACGGGGAAAGAATCGAAAAAACTTCACTTC
ATCGTCCCAATACTTGCCTGCGTTTGTGGGCATTTTGATGGAGAAGTCCGGATTCTCGCCGAAAAGACAGTTACCGGGAAACGAGTA
CATGGAGACGGGTCTTTTGAATTTGTGATTGAACGTGGCAGTAAACGTGTGTGCATCGTGGAGGCGAAACGAGATGATTTTCAGCAG
GGCCTCGCGCAGGCTTACGTAGGGTGCGAAGTACTCGCAGATGTCGAAGGATTGACGAAGCTATACAGCATCGTCACAAATTACAAG
GAATGGTACTTCTCCAGAAGTCTGGATGACAGAATCGAGCGATTTGATGTCACGATGGATATCGTGAACGACATTCCTACGCGAGAA
TCGGTGAAGATGATTGCAGAGAAGATCTACTCCATGCTGTCAGATGATGATGAGCCAGCCGTGGCCTCCAACGAGGCTATCCTATGA
CGTTGGGTCGGCATAGTCAAGTCATCTCTGCGCTCCCTTTTTAAGGATCGTTTGATGCGTACGCTTAACTCAGTATGCTGGAGCCAG
GAAGCAGCAACAAGATGCGAAGGTGGCAGGGTACTATTATTAAGAGATCAGAAAAAGATAACCACAATCTAGTATTA
>PhCRN17.1_334 PLHAL3342065
TTCCTAGCCAAGACAGAGGGCGGCGCTTGGTTGTCAAGCCAATCAGACGACGTGAAGAAGCTCAAGAAGGGCGAGAAGACAGCTCTC
ATCGAATCGCTAATAAAGGAGGATCAACAGCTACAGGCGGAGGACCCGCTGGAATACGTGCTGAGAGAAAACAGTATGGTCACCCCA
CAGTTAACACAGATTCACGTGCTGGTGCTGGTTCCTGGACAACGCCTATCCCTAGGTGCCGCCGCTCTTCGTGAACCCCATCCCGTC
CGCAAGAAAAGGTGGAAAGAACTGAACGAAGTACTTGATCGGAACAAAAGGTCGAAGGTCAACTCTGCTGGTGAGTCCTCCACAGGG
TACTCATACGTGTCGTTCGCCGATGTCGACAAAATAATGAGGACGCGTCGCTACGAGCAGCCATCGAAAGTCGTCGAAAATGACAAA
CTTGACATGCTCTATGCGTATCTCCTGCTCCTACCTAAAGCATTCGGAGACATCGTGACGGGGAAAGAATCGAAAAAACTTCACTTC
ATCGTCCCAATACTTGCCTGCGTTTGTGGGCATTTTGATGGAGAAGTCCGGATTCTCGCCGAAAAGACAGTTACCGGGAAACGAGTA
CATGGAGACGGGTCTTTTGAATTTGTGATTGAACGTGGCAGTAAACGTGTGTGCATCGTGCAGGCGAAACGAGATGATTTTCAGCAG
GGCCTCGCGCAGGCTTACGTAGGGTGCGAAGTACTCGCAGATGTCGAAGGATTGACGAAGCTATACAGCATCGTCACAAATTACAAG
GAATGGTACTTCTCCAGAAGTCTGGATGACAGAATCGAGCGATTTGATGTCACGATGGATATCGTGAACGACATTCCTACGCGAGAA
TCGGTGAAGATGATTGCAGAGAAGATCTACTCCATGCTGTCAGATGATGATGAGCCAGCCGTGGCCTCCAACGAGGCTATCCTATGA
CGTTGGGTCGGCATAGTCAAGTCATCTCTGCGCTCCCTTTTTAAGGATCGTTTGATGCGTACGCTTAACTCAGTATGCTGGAGCCAG
GAAGCAGCAACAAGATGCGAAGGTGGCAGGGTACTATTATTAAGAGATCAGAAAAAGATAACCACAATCTAGTATTA
>PhCRN17.1_703 PLHAL7031836_A
TTCCTAGCCAAGACAGAGGGCGGCGCTTGGTTGTCAAGCCAATCAGACGACGTGAAGAAGCTCAAGAAGGGCGAGAAGACAGCTCTC
ATCGAATCGCTAATAAAGGAGGATCAACAGCTACAGGCGGAGGACCCGCTGGAATACGTGCTGAGAGAAAACAGTATGGTCACCCCA
CAGTTAACACAGATTCACGTGCTGGTGCTGGTTCCTGGACAACGCCTATCCCTAGGTGCCGCCGCTCTTCGTGAACCCCATCCCGTC
CGCAAGAAAAGGTGGAAAGAACTGAACGAAGTACTTGATCGGAACAAAAGGTCGAAGGTCAACTCTGCTGGTGAGTCCTCCACAGGG
TACTCATACGTGTCGTTCGCCGATGTCGACAAAATAATGAGGACGCGTCGCTACGAGCAGCCATCGAAAGTCGTCGAAAATGACAAA
CTTGACATGCTCTATGCGTATCTCCTGCTCCTACCTAAAGCATTCGGAGACATCGTGACGGGGAAAGAATCGAAAAAACTTCACTTC

ATCGTCCCAATACTTGCCTGCGTTTGTGGGCATTTTGATGGAGAAGTCCGGATTCTCGCCGAAAAGACAGTTACCGGGAAACGAGTA
CATGGAGACGGGTCTTTTGAATTTGTGATTGA
>PhCRN17.1_703 PLHAL7031836_B
GTGATTGAACGTGGCAGTAAACATTTGTGCATCTGGCAGGCGAAACGAGATGATTTTCAGCAGGGCCTCGCGCAGGCTTACGTAGGG
TGCGAAGTACTCGCAGATGTCGAAGGATTGACGAAGCTATACAGCATCGTCACAAATTACAAGGAATGGTACTTCTCCAGAAGTCTG
GATGACAGAATCGAGCGATTTGATGTCACGATGGATATCGTGAACGACATTCCTACGCGAGAATCGGTGAAGATGATTGCAGAGAAG
ATCTACTCCATGCTGTCAGATGATGATGAGCCAGCCGTGGCCTCCAACGAGGCTATCCTATGACGTTGGGTCGGCATAGTCAAGTCA
TCTCTGCGCTCCCTTTTTAAGGATCGTTTGATGCGTACGCTTAACTCAGTATGCTGGAGCCAGGAAGCAGCAACAAGATGCGAAGGT
GGCAGGGTACTATTATTAAGAGATCAGAAAAAGATAACCACAATCTAGTATTA
>PhCRN17.1_710 PLHAL7101563
TTCCTAGCCAAGACAGAGGGCGGCGCTTGGTTGTCAAGCCAATCAGACGACGTGAAGAAGCTCAAGAAGGGCGAGAAGACAGCTCTC
ATCGAATCGCTAATAAAGGAGGATCAACAGCTACAGGCGGAGGACCCGCTGGAATACGTGCTGAGAGAAAACAGTATGGTCACCCCA
CAGTTAACACAGATTCACGTGCTGGTGCTGGTTCCTGGACAACGCCTATCCCTAGGTGCCGCCGCTCTTCGTGAACCCCATCCCGTC
CGCAAGAAAAGGTGGAAAGAACTGAACGAAGTACTTGATCGGAACAAAAGGTCGAAGGTCAACTCTGCTGGTGAGTCCTCCACAGGG
TACTCATACGTGTCGTTCGCCGATGTCGACAAAATAATGAGGACGCGTCGCTACGAGCAGCCATCGAAAGTCGTCGAAAATGACAAA
CTTGACATGCTCTATGCGTATCTCCTGCTCCTACCTAAAGCATTCGGAGACATCGTGACGGGGAAAGAATCGAAAAAACTTCACTTC
ATCGTCCCAATACTTGCCTGCGTTTGTGGGCATTTTGATGGAGAAGTCCGGATTCTCGCCGAAAAGACAGTTACCGGGAAACGAGTA
CATGGAGACGGGTCTTTTGAATTTGTGATTGAACGTGGCAGTAAACGTGTGTGCATCGTGGAGGCGAAACGAGATGATTTTCAGCAG
GGCCTCGCGCAGGCTTACGTAGGGTGCGAAGTACTCGCAGATGTCGAAGGATTGACGAAGCTATACAGCATCGTCACAAATTACAAG
GAATGGTACTTCTCCAGAAGTCTGGATGACAGAATCGAGCGATTTGATGTCACGATGGATATCGTGAACGACATTCCTACGCGAGAA
TCGGTGAAGATGATTGCAGAGAAGATCTACTCCATGCTGTCAGATGATGATGAGCCAGCCGTGGCCTCCAACGAGGCTATCCTATGA
CGTTGGGTCGGCATAGTCAAGTCATCTCTGCGCTCCCTTTTTAAGGATCGTTTGATGCGTACGCTTAACTCAGTATGCTGGAGCCAG
GAAGCAGCAACAAGATGCGAAGGTGGCAGGGTACTATTATTAAGAGATCAGAAAAAGATAACCACAATCTAGTATTA
>PhCRN17.1_304 PLHAL3041959
TTCCTAGCCAAGACAGAGGGCGGCGCTTGGTTGTCAAGCCAATCAGACGACGTGAAGAAGCTCAAGAAGGGCGAGAAGACAGCTCTC
ATCGAATCGCTAATAAAGGAGGATCAACAGCTACAGGCGGAGGACCCGCTGGAATACGTGCTGAGAGAAAACAGTATGGTCACCCCA
CAGTTAACACAGATTCACGTGCTGGTGCTGGTTCCTGGACAACGCCTATCCCTAGGTGCCGCCGCTCTTCGTGAACCCCATCCCGTC
CGCAAGAAAAGGTGGAAAGAACTGAACGAAGTACTTGATCGGAACAAAAGGTCGAAGGTCAACTCTGCTGGTGAGTCCTCCACAGGG
TACTCATACGTGTCGTTCGCCGATGTCGACAAAATAATGAGGACGCGTCGCTACGAGCAGCCATCGAAAGTCGTCGAAAATGACAAA
CTTGACATGCTCTATGCGTATCTCCTGCTCCTACCTAAAGCATTCGGAGACATCGTGACGGGGAAAGAATCGAAAAAACTTCACTTC
ATCGTCCCAATACTTGCCTGCGTTTGTGGGCATTTTGATGGAGAAGTCCGGATTCTCGCCGAAAAGACAGTTACCGGGAAACGAGTA
CATGGAGACGGGTCTTTTGAATTTGTGATTGAACGTGGCAGTAAACGTGTGTGCATCGTGGAGGCGAAACGAGATGATTTTCAGCAG
GGCCTCGCGCAGGCTTACGTAGGGTGCGAAGTACTCGCAGATGTCGAAGGATTGACGAAGCTATACAGCATCGTCACAAATTTCAAG
GAATGGTACTTCTCCAGAAGTCTGGATGACAGAATCGAGCGATTTGATGTCACGATGGATATCGTGAACGACATTCCTACGCGAGAA
TCGGTGAAGATGATTGCAGAGAAGATCTACTCCATGCTGTCAGATGATGATGAGCCAGCCGTGGCCTCCAACGAGGCTATCCTATGA
CGTTGGGTCGGCATAGTCAAGTCATCTCTGCGCTCCCTTTTTAAGGATCGTTTGATGCGTACGCTTAACTCAGTATGCTGGAGCCAG
GAAGCAGCAACAAGATGCGAAGGTGGCAGGGTACTATTATTAAGAGATCAGAAAAAGATAACCACAATCTAGTATTA
>PhCRN17.1_700a PLHAL7002998
GGTGAAGATGATTGCAGAGAAGATCTACTCCATGCTGTCAGATGATGATGAGCCAGCCGTGGCCTCCAACGAGGCTATCCTATGACG
TTGGGTCGGCATAGTCAAGTCATCTCTGCGCTCCCTTTTTAAGGATCGTTTGATGCGTACGCTTAACTCAGTATGCTGGAGCCAGGA
AGCAGCAACAAGATGCGAAGGTGGCAGGGTACTATTATTAAGAGATCAGAAAAAGATAACCACAATCTAGTATTA
>PhCRN17.1_700b PLHAL7003118
TTCCTAGCCAAGACAGAGGGCGGCGCTTGGTTGTCAAGCCAATCAGACGACGTGAAGAAGCTCAAGAAGGGCGAGAAGACAGCTCTC
ATCGAATCGCTAATAAAGGAGGATCAACAGCTACAGGCGGAGGACCCGCTGGAATACGTGCTGAGAGAAAACAGTATGGTCACCCCA
CAGTTAACACAGATTCACGTGCTGGTGCTGGTTCCTGGACAACGCCTATCCCTAGGTGCCGCCGCTCTTCGTGAACCCCATCCCGTC
CGCAAGAAAAGGTGGAAAGAACTGAACGAAGTACTTGATCGGAACAAAAGGTCGAAGGTCAACTCTGCTGGTGAGTCCTCCACAGGG
TACTCATACGTGTCGTTCGCCGATGTCGACAAAATAATGAGGACGCGTCGCTACGAGCAGCCATCGAAAGTCGTCGAAAATGACAAA
CTTGACATGCTCTATGCGTATCTCCTGCTCCTACCTAAAGCATTCGGAGACATCGTGACGGGGAAAGAATCGAAAAAACTTCACTTC
ATCGTCCCAATACTTGCCTGCGTTTGTGGGCATTTTGATGGAGAAGTCCGGATTCTCGCCGAAAAGACAGTTACCGGGAAACGAGTA
CATGGAGACGGGTCTTTTGAATTTGTGATTGAACGTGGCAGTAAACGTGTGTGCATCGTGGAGGCGAAACGAGATGATTTTCAGCAG
GGCCTCGCGCAGGCTTACGTAGGGTGCGAAGTACTCGCAGATGTCGAAGGATTGACGAAGCTATACAGCATCGTCACAAATTACAAG
GAATGGTACTTCTCCAGAAGTCTGGATGACAGAATCGAGCGATTTGATGTCACGATGGATATCGTGAACGACATTCCTACGCGAGAA
TCGGTGAAGATGATTGCAGAGAAGATCTACTCC
>PhCRN17.1_730 PLHAL7301897
TTCCTAGCCAAGACAGAGGGCGGCGCTTGGTTGTCAAGCCAATCAGACGACGTGAAGAAGCTCAAGAAGGGCGAGAAGACAGCTCTC
ATCGAATCGCTAATAAAGGAGGATCAACAGCTACAGGCGGAGGACCCGCTGGAATACGTGCTGAGAGAAAACAGTATGGTCACCCCA
CAGTTAACACAGATTCACGTGCTGGTGCTGGTTCCTGGACAACGCCTATCCCTAGGTGCCGCCGCTCTTCGTGAACCCCATCCCGTC
CGCAAGAAAAGGTGGAAAGAACTGAACGAAGTACTTGATCGGAACAAAAGGTCGAAGGTCAACTCTGCTGGTGAGTCCTCCACAGGG
TACTCATACGTGTCGTTCACCGATGTCGACAAAATAATGAGGACGCGTCGCTACGAGCAGCCATCGAAAGTCGTCGAAAATGACAAA
CTTGACATGCTCTATGCGTATCTCCTGCTCCTACCTAAAGCATTCGGAGACATCGTGACGGGGAAAGAATCGAAAAAACTTCACTTC
ATCGTCCCAATACTTGCCTGCGTTTGTGGGCATTTTGATGGAGAAGTCCGGATTCTCGCCGAAAAGACAGTTACCGGGAAACGAGTA
CATGGAGACGGGTCTTTTGAATTTGTGATTGAACGTGGCAGTAAACGTGTGTGCATCGTGGAGGCGAAACGAGATGATTTTCAGCAG
GGCCTCGCGCAGGCTTACGTAGGGTGCGAAGTACTCGCAGATGTCGAAGGATTGACGAAGCTATACAGCATCGTCACAAATTACAAG
GAATGGTACTTCTCCAGAAGTCTGGATGACAGAATCGAGCGATTTGATGTCACGATGGATATCGTGAACGACATTCCTACGCGAGAA
TCGGTGAAGATGATTGCAGAGAAGATCTACTCCATGCTGTCAGATGATGATGAGCCAGCCGTGGCCTCCAACGAGGCTATCCTATGA
CGTTGGGTCGGCATAGTCAAGTCATCTCTGCGCTCCCTTTTTAAGGATCGTTTGATGCGTACGCTTAACTCAGTATGCTGGAGCCAG
GAAGCAGCAACAAGATGCGAAGGTGGCAGGGTACTATTATTAAGAGATCAGAAAAAGATAACCACAATCTAGTATTA

>PhCRN17.2_100 PLHAL100A10115
TTGCGTCACACCACCATGTTCGATTAAGTACGCCTGAAATCCTCCCTCTCACCTTCCACATTCCAGAATGTACCGGCTCGAGTAAAT
ATCGTGAAGCTTGTTTGTGCGATCGTTGGTCCGACAGGAAACGCGTTTCCTGTGGATATCGATGCGGATCAGCCGGTAGGAGACCTC
AAGAAGATAATCAAGGCGGACAGTCCAGACAGAATCAAGTGCGACGCAGCTATGTTGGAGCTTTTCCTAGCCAAGACAGAGGGCGGC
GCTTGGTTATCAAGCCAATCAGATGACGTGAAGAAGCTTAAGAAGGGCGAGAAGACAGCTCTCATCAAATCGCTAATAAAGGAGGAT
CAACAGCTACAGGCGGAGGTCCCGCTGGAATACGTGCTAAGAGAAAACAATATGGCCACCCCACAGCTGAGACAGATTCACGTTCTG
GTGGTGGTTCC
>PhCRN17.2_334 PLHAL3341375
TTGCGTCACACCACCATGTTCGATTAAGTACGCCTGAAATCCTCCCTCTCACCTTCCACATTCCAGAATGTACCGGCTCGAGTAAAT
ATCGTGAAGCTTGTTTGTGCGATCGTTGGTCCGACAGGAAACGCGTTTCCTGTGGATATCGATGCGGATCAGCCGGTAGGAGACCTC
AAGAAGATAATCAAGGCGGACAGTCCAGACAGAATCAAGTGCGACGCAGCTATGTTGGAGCTTTTCCTAGCCAAGACAGAGGGCGGC
GCTTGGTTATCAAGCCAATCAGATGACG
>PhCRN17.2_703 PLHAL7030124
TTGCGTCACACCACCATGTTCGATTAAGTACGCCTGAAATCCTCCCTCTCACCTTCCACATTCCAGAATGTACCGGCTCGAGTAAAT
ATCGTGAAGCTTGTTTGTGCGATCGTTGGTCCGACAGGAAACGCGTTTCCTGTGGATATCGATGCGGATCAGCCGGTAGGAGACCTC
AAGAAGATAATCAAGGCGGACAGTCCAGACAGAATCAAGTGCGACGCAGCTATGTTGGAGCTTTTCCTAGCCAAGACAGAGGGCGGC
GCTTGGTTATCAAGCCAATCAGATGACGTGAAGAAGCTCAAGAAGGGCGAGAAGACAGCTCTCATCAAATCGCTAATAAAGGAGGAT
CAACAGCTACAGGCGGAGGACCCGCTGGAATACGTGCTGAGAGAAAACAATATGGCCACCCCACAGCTGAGACAGATTCACGTTCTG
GTGGTGGTTCC
>PhCRN17.2_710 PLHAL7100116
TTGCGTCACACCACCATGTTCGATTAAGTACGCCTGAAATCCTCCCTCTCACCTTCCACATTCCAGAATGTACCGGCTCGAGTAAAT
ATCGTGAAGCTTGTTTGTGCGATCGTTGGTCCGACAGGAAACGCGTTTCCTGTGGATATCGATGCGGATCAGCCGGTAGGAGACCTC
AAGAAGATAATCAAGGCGGACAGTCCAGACAGAATCAAGTGCGACGCAGCTATGTTGGAGCTTTTCCTAGCCAAGACAGAGGGCGGC
GCTTGGTTATCAAGCCAATCAGATGACGTGAAGAAGCTCAAGAAGGGCGAGAAGACAGCTCTCATCAAATCGCTAATAAAGGAGGAT
CAACAGCTACAGGCGGAGGACCCGCTGGAATACGTGCTGAGAGAAAACAATATGGCCACCCCACAGCTGAGACAGATTCACGTTCTG
GTGGTGGTTCC
>PhCRN17.2_304 PLHAL3040745
TTGCGTCACACCACCATGTTCGATTAAGTACGCCTGAAATCCTCCCTCTCACCTTCCACATTCCAGAATGTACCGGCTCGAGTAAAT
ATCGTGAAGCTTGTTTGTGCGATCGTTGGTCCGACAGGAAACGCGTTTCCTGTGGATATCGATGCGGATCAGCCGGTAGGAGACCTC
AAGAAGATAATCAAGGCGGACAGTCCAGACAGAATCAAGTGCGACGCAGCTATGTTGGAGCTTTTCCTAGCCAAGACAGAGGGCGGC
GCTTGGTTATCAAGCCAATCAGATGACGTGAA
>PhCRN17.2_700 PLHAL7001006
TTGCGTCACACCACCATGTTCGATTAAGTACGCCTGAAATCCTCCCTCTCACCTTCCACATTCCAGAATGTACCGGCTCGAGTAAAT
ATCGTGAAGCTTGTGAAGGTTATTTGTGCGATCGTTGGTCCGACAGGAAACGCGTTTCCTGTGGATATCGATGCGGATCAGCCGGTA
GGAGACCTCAAGAAGATAATCAAGGCGGACAGTCCAGACAGAATCAAGTGCGACGCAGCTATGTTGGAGCTTTTCCTAGCCAAGACA
GAGGGCGGCGCTTGGTTATCAAGCCAATCAGATGACGTGAAGAAGCTCAAGAAGGGCGAGAAGACAGCTCTCATCAAATCGCTAATA
AAGGAGGATCAACAGCTACAGGC
>PhCRN17.2_730 PLHAL7300117
TTGCGTCACACCACCATGTTCGATTAAGTACGCCTGAAATCCTCCCTCTCACCTTCCACATTCCAGAATGTACCGGCTCGAGTAAAT
ATCGTGAAGCTTGTTTGTGCGATCGTTGGTCCGACAGGAAACGCGTTTCCTGTGGATATCGATGCGGATCAGCCGGTAGGAGACCTC
AAGAAGATAATCAAGGCGGACAGTCCAGACAGAATCAAGTGCGACGCAGCTATGTTGGAGCTTTTCCTAGCCAAGACAGAGGGCGGC
GCTTGGTTATCAAGCCAATCAGATGACGTGAAGAAGCTCAAGAAGGGCGAGAAGACAGCTCTCATCAAATCGCTAATAAAGGAGGAT
CAACAGCTACAGGC

>PhCRN17.3_100 PLHAL100A13544
GAACAACAGCTACAGGCGGAGGACCCGCTGGAATACGTGCTGAGAGAAAAAAGTATGGCCACCCCACAGTCGAGACAGATTCACGTT
CTGGTGGTGGTTCCTGGACAAAGCCTATCACTAGGTGCCGCCGCTCTTCGTGAACCCCATCCCGTCCGCAAGAAAAGGTGGGAAGAA
CTGAACAAAGTACTTGATCGGAACAAAAGGTCGAAGGTCAACTCTGCTGGTGAGTCCTCCACAGGGTACTCATACGTGTCGTTCACC
GATGTCGACAAAATAATGAAGGCGCGTCGCTACGAGCAGCCATCGAAAGTCGTCGAAAATGACAAACTTGACGTGCTCCATGCGTAT
CTGCTACTCCTGACCAAAGCTTTCGGAGAAATCGTGACGGGAAAAGAAGCGAAAAGACTTCACTTCATCGTCCCAATACTTGCCTGC
GTTTGTGGGCTTTTTGATGGAGAAGTCCGGATTCTCGCTGAAGAGACAGTTACCGGGAAACGAGTACATGGAGACGGGTCTTTTGAA
TTTGTGATTGAACGTGGCAGTAAACGTGTGTGCATCGTGGAGGCGAAACGAGATGATTTTCAGCAGGGCCTCGCGCAGGCTTACGTA
GGGTGCGAAGTACTCGCAGATGTCGAAGGATTGACGAAGCTATACAGCATCGTCACAAATTTCAAGGAATGGTACTTCTCCAGAAGT
CTGGATGACAGAATCGAGCGATTTGATGCAACGATTAATATCGTGAACGACATTCCTACGCGAGAATCGGTGAAGATGATTGCAGAG
AAGATCTACTCCATGCTGTCAGATGATGATGAGCCAGCCGTGGTCTCCAACGAGGCTATCCAATGACGTTGGGTCGGCATTGTCAAG
TCATCTCTGCGCTCCCTTTTGAAGGATCGTTTGATGCGTACGCTTAGATCAGTATGCTAGAGCTAGGAAGCAGCAACAA
>PhCRN17.3_334 PLHAL3343622
GAACAACAGCTACAGGCGGAGGACCCGCTGGAATACGTGCTGAGAGAAAAAAGTATGGCCACCCCACAGTCGAGACAGATTCACGTT
CTGGTGGTGGTTCCTGGACAAAGCCTATCACTAGGTGCCGCCGCTCTTCGTGAACCCCATCCCGTCCGCAAGAAAAGGTGGGAAGAA
CTGAACAAAGTACTTGATCGGAACAAAAGGTCGAAGGTCAACTCTGCTGGTGAGTCCTCCACAGGGTACTCATACGTGTCGTTCACC
GATGTCGACAAAATAATGAAGGCGCGTCGCTACGAGCAGCCATCGAAAGTCGTCGAAAATGACAAACTTGACGTGCTCCATGCGTAT
CTGCTACTCCTGACCAAAGCTTTCGGAGAAATCGTGACGGGAAAAGAAGCGAAAAGACTTCACTTCATCGTCCCAATACTTGCCTGC
GTTTGTGGGCTTTTTGATGGAGAAGTCCGGATTCTCGCTGAAGAGACAGTTACCGGGAAACGAGTACATGGAGACGGGTCTTTTGAA
TTTGTGATTGAACGTGGCAGTAAACGTGTGTGCATCGTGGAGGCGAAACGAGATGATTTTCAGCAGGGCCTCGCGCAGGCTTACGTA
GGGTGCGAAGTACTCGCAGATGTCGAAGGATTGACGAAGCTATACAGCATCGTCACAAATTTCAAGGAATGGTACTTCTCCAGAAGT
CTGGATGACAGAATCGAGCGATTTGATGCAACGATTAATATCGTGAACGACATTCCTACGCGAGAATCGGTGAAGATGATTGCAGAG

AAGATCTACTCCATGCTGTCAGATGATGATGAGCCAGCCGTGGTCTCCAACGAGGCTATCCAATGACGTTGGGTCGGCATTGTCAAG
TCATCTCTGCGCTCCCTTTTGAAGGATCGTTTGATGCGTACGCTTAGATCAGTATGCTAGAGCTAGGAAGCAGCAACAA
>PhCRN17.3_703 PLHAL7032950
GAACAACAGCTACAGGCGGAGGACCCGCTGGAATACGTGCTGAGAGAAAAAAGTATGGCCACCCCACAGTCGAGACAGATTCACGTT
CTGGTGGTGGTTCCTGGACAAAGCCTATCACTAGGTGCCGCCGCTCTTCGTGAACCCCATCCCGTCCGCAAGAAAAGGTGGGAAGAA
CTGAACAAAGTACTTGATCGGAACAAAAGGTCGAAGGTCAACTCTGCTGGTGAGTCCTCCACAGGGTACTCATACGTGTCGTTCACC
GATGTCGACAAAATAATGAAGGCGCGTCGCTACGAGCAGCCATCGAAAGTCGTCGAAAATGACAAACTTGACGTGCTCCATGCGTAT
CTGCTACTCCTGACCAAAGCTTTCGGAGAAATCGTGACGGGAAAAGAAGCGAAAAGACTTCACTTCATCGTCCCAATACTTGCCTGC
GTTTGTGGGCTTTTTGATGGAGAAGTCCGGATTCTCGCTGAAGAGACAGTTACCGGGAAACGAGTACATGGAGACGGGTCTTTTGAA
TTTGTGATTGAACGTGGCAGTAAACGTGTGTGCATCGTGGAGGCGAAACGAGATGATTTTCAGCAGGGCCTCGCGCAGGCTTACGTA
GGGTGCGAAGTACTCGCAGATGTCGAAGGATTGACGAAGCTATACAGCATCGTCACAAATTTCAAGGAATGGTACTTCTCCAGAAGT
CTGGATGACAGAATCGAGCGATTTGATGCAACGATTAATATCGTGAACGACATTCCTACGCGAGAATCGGTGAAGATGATTGCAGAG
AAGATCTACTCCATGCTGTCAGATGATGATGAGCCAGCCGTGGTCTCCAACGAGGCTATCCAATGACGTTGGGTCGGCATTGTCAAG
TCATCTCTGCGCTCCCTTTTGAAGGATCGTTTGATGCGTACGCTTAGATCAGTATGCTAGAGCTAGGAAGCAGCAACAA
>PhCRN17.3_710 PLHAL7103059
GAACAACAGCTACAGGCGGAGGACCCGCTGGAATACGTGCTGAGAGAAAAAAGTATGGCCACCCCACAGTCGAGACAGATTCACGTT
CTGGTGGTGGTTCCTGGACAAAGCCTATCACTAGGTGCCGCCGCTCTTCGTGAACCCCATCCCGTCCGCAAGAAAAGGTGGGAAGAA
CTGAACAAAGTACTTGATCGGAACAAAAGGTCGAAGGTCAACTCTGCTGGTGAGTCCTCCACAGGGTACTCATACGTGTCGTTCACC
GATGTCGACAAAATAATGAAGGCGCGTCGCTACGAGCAGCCATCGAAAGTCGTCGAAAATGACAAACTTGACGTGCTCCATGCGTAT
CTGCTACTCCTGACCAAAGCTTTCGGAGAAATCGTGACGGGAAAAGAAGCGAAAAGACTTCACTTCATCGTCCCAATACTTGCCTGC
GTTTGTGGGCTTTTTGATGGAGAAGTCCGGATTCTCGCTGAAGAGACAGTTACCGGGAAACGAGTACATGGAGACGGGTCTTTTGAA
TTTGTGATTGAACGTGGCAGTAAACGTGTGTGCATCGTGGAGGCGAAACGAGATGATTTTCAGCAGGGCCTCGCGCAGGCTTACGTA
GGGTGCGAAGTACTCGCAGATGTCGAAGGATTGACGAAGCTATACAGCATCGTCACAAATTTCAAGGAATGGTACTTCTCCAGAAGT
CTGGATGACAGAATCGAGCGATTTGATGCAACGATTAATATCGTGAACGACATTCCTACGCGAGAATCGGTGAAGATGATTGCAGAG
AAGATCTACTCCATGCTGTCAGATGATGATGAGCCAGCCGTGGTCTCCAACGAGGCTATCCAATGACGTTGGGTCGGCATTGTCAAG
TCATCTCTGCGCTCCCTTTTGAAGGATCGTTTGATGCGTACGCTTAGATCAGTATGCTAGAGCTAGGAAGCAGCAACAA
>PhCRN17.3_304 PLHAL3043498
GAACAACAGCTACAGGCGGAGGACCCGCTGGAATACGTGCTGAGAGAAAAAAGTATGGCCACCCCACAGTCGAGACAGATTCACGTT
CTGGTGGTGGTTCCTGGACAAAGCCTATCACTAGGTGCCGCCGCTCTTCGTGAACCCCATCCCGTCCGCAAGAAAAGGTGGGAAGAA
CTGAACAAAGTACTTGATCGGAACAAAAGGTCGAAGGTCAACTCTGCTGGTGAGTCCTCCACAGGGTACTCATACGTGTCGTTCACC
GATGTCGACAAAATAATGAAGGCGCGTCGCTACGAGCAGCCATCGAAAGTCGTCGAAAATGACAAACTTGACGTGCTCCATGCGTAT
CTGCTACTCCTGACCAAAGCTTTCGGAGAAATCGTGACGGGAAAAGAAGCGAAAAGACTTCACTTCATCGTCCCAATACTTGCCTGC
GTTTGTGGGCTTTTTGATGGAGAAGTCCGGATTCTCGCTGAAGAGACAGTTACCGGGAAACGAGTACATGGAGACGGGTCTTTTGAA
TTTGTGATTGAACGTGGCAGTAAACGTGTGTGCATCGTGGAGGCGAAACGAGATGATTTTCAGCAGGGCCTCGCGCAGGCTTACGTA
GGGTGCGAAGTACTCGCAGATGTCGAAGGATTGACGAAGCTATACAGCATCGTCACAAATTTCAAGGAATGGTACTTCTCCAGAAGT
CTGGATGACAAAATCGAGCGATTTGATGCAACGATTAATATCGTGAACGACATTCCTACGCGAGAATCGGTGAAGATGATTGCAGAG
AAGATCTACTCCATGCTGTCAGATGATGATGAGCCAGCCGTGGTCTCCAACGAGGCTATCCAATGACGTTGGGTCGGCATTGTCAAG
TCATCTCTGCGCTCCCTTTTGAAGGATCGTTTGATGCGTACGCTTAGATCAGTATGCTAGAGCTAGGAAGCAGCAACAA
>PhCRN17.3_700 PLHAL7002833
GAACAACAGCTACAGGCGGAGGACCCGCTGGAATACGTGCTGAGAGAAAAAAGTATGGCCACCCCACAGTCGAGACAGATTCACGTT
CTGGTGGTGGTTCCTGGACAAAGCCTATCACTAGGTGCCGCCGCTCTTCGTGAACCCCATCCCGTCCGCAAGAAAAGGTGGGAAGAA
CTGAACAAAGTACTTGATCGGAACAAAAGGTCGAAGGTCAACTCTGCTGGTGAGTCCTCCACAGGGTACTCATACGTGTCGTTCACC
GATGTCGACAAAATAATGAAGGCGCGTCGCTACGAGCAGCCATCGAAAGTCGTCGAAAATGACAAACTTGACGTGCTCCATGCGTAT
CTGCTACTCCTGACCAAAGCTTTCGGAGAAATCGTGACGGGAAAAGAAGCGAAAAGACTTCACTTCATCGTCCCAATACTTGCCTGC
GTTTGTGGGCTTTTTGATGGAGAAGTCCGGATTCTCGCTGAAG
>PhCRN17.3_730 PLHAL7303581
GAACAACAGCTACAGGCGGAGGACCCGCTGGAATACGTGCTGAGAGAAAAAAGTATGGCCACCCCACAGTCGAGACAGATTCACGTT
CTGGTGGTGGTTCCTGGACAAAGCCTATCACTAGGTGCCGCCGCTCTTCGTGAACCCCATCCCGTCCGCAAGAAAAGGTGGGAAGAA
CTGAACAAAGTACTTGATCGGAACAAAAGGTCGAAGGTCAACTCTGCTGGTGAGTCCTCCACAGGGTACTCATACGTGTCGTTCACC
GATGTCGACAAAATAATGAAGGCGCGTCGCTACGAGCAGCCATCGAAAGTCGTCGAAAATGACAAACTTGACGTGCTCCATGCGTAT
CTGCTACTCCTGACCAAAGCTTTCGGAGAAATCGTGACGGGAAAAGAAGCGAAAAGACTTCACTTCATCGTCCCAATACTTGCCTGC
GTTTGTGGGCTTTTTGATGGAGAAGTCCGGATTCTCGCTGAAGAGACAGTTACCGGGAAACGAGTACATGGAGACGGGTCTTTTGAA
TTTGTGATTGAACGTGGCAGTAAACGTGTGTGCATCGTGGAGGCGAAACGAGATGATTTTCAGCAGGGCCTCGCGCAGGCTTACGTA
GGGTGCGAAGTACTCGCAGATGTCGAAGGATTGACGAAGCTATACAGCATCGTCACAAATTTCAAGGAATGGTACTTCTCCAGAAGT
CTGGATGACAGAATCGAGCGATTTGATGCAACGATTAATATCGTGAACGACATTCCTACGCGAGAATCGGTGAAGATGATTGCAGAG
AAGATCTACTCCATGCTGTCAGATGATGATGAGCCAGCCGTGGTCTCCAACGAGGCTATCCAATGACGTTGGGTCGGCATTGTCAAG
TCATCTCTGCGCTCCCTTTTGAAGGATCGTTTGATGCGTACGCTTAGATCAGTATGCTAGAGCTAGGAAGCAGCAACAA

>PhCRN17.4_100 PLHAL100A10522
GGTTTAGAAATTGCGTTACACCACCATGTTCGCTTAAGTACGCCTGAAACTCTTCCTCACCTTCCACATTCCAAAATCTACCGGCTC
GTGTAAAGATGGTGAAGCTTATTTGTGCGATCGTTGGTCCGGCAGGAAACGCGTTTCCTGTGGACATCGATGCGGATCAGCTGGTGG
GAGACCTCAAGGACGCGATTAGGGCGAAGAATCCAGACACGATCAAGTGCGAGGCAGCTAAGTTGGAGCTCTTCCTGGCCATAACAG
ATGGCGGCGCTTGGTTATCAAGCCAATCAGATGACGTGAAGAAGCTTAAGAAGGGCGAGAAGACAGCTCTCATCAAATTGCTAATAA
AGGAGGATCAACAGCTACAGGCGGAGGACCCTTTGGAATACGTGCTGAGAGAAAACAATATGGCCACCCCACAGTCGAGACAGATTC
ACGTTCTGGTGGTGGTTCC
>PhCRN17.4_334 PLHAL3341699

GGTTTAGAAATTGCGTTACACCACCATGTTCGCTTAAGTACGCCTGAAACTCTTCCTCACCTTCCACATTCCAAAATCTACCGGCTC
GTGTAAAGATGGTGAAGCTTATTTGTGCGATCGTTGGTCCGGCAGGAAACGCGTTTCCTGTGGACATCGATGCGGATCAGCTGGTGG
GAGACCTCAAGGACGCGATTAGGGCGAAGAATCCAGACACGATCAAGTGCGAGGCAGCTAAGTTGGAGCTCTTCCTGGCCATAACAG
ATGGCGGCGCTTGGTTATCAAGCCAATCAGATGACGTGAAGAAGCTTAAGAAGGGCGAGAAGACAGCTCTCATCAAATTGCTAATAA
AGGAGGATCAACAGCTACAGGCGGAGGACCCTTTGGAATACGTGCTGAGAGAAAACAATATGGCCACCCCACAGTCGAGACAGATTC
ACGTTCTGGTGGTGGTTCC
>PhCRN17.4_703 PLHAL7031340
GGTTTAGAAATTGCGTTACACCACCATGTTCGCTTAAGTACGCCTGAAACTCTTCCTCACCTTCCACATTCCAAAATCTACCGGCTC
GTGTAAAGATGGTGAAGCTTATTTGTGCGATCGTTGGTCCGGCAGGAAACGCGTTTCCTGTGGACATCGATGCGGATCAGCTGGTGG
GAGACCTCAAGGACGCGATTAGGGCGAAGAATCCAGACACGATCAAGTGCGAGGCAGCTAAGTTGGAGCTCTTCCTGGCCATGACAG
ATGGCGGCGCTTGGTTATCAAGCCAATCAGATGACGTGAAGAAGCTTAAGAAGGGCGAGAAGACAGCTCTCATCAAATTGCTAATAA
AGGAGGATCAACAGCTACAGGCGGAGGACCCTTTGGAATACGTGCTGAGAGAAAACAATATGGCCACCCCACAGTCGAGACAGATTC
ACGTTCTGGTGGTGGTTCC
>PhCRN17.4_710 PLHAL7100553
GGTTTAGAAATTGCGTTACACCACCATGTTCGCTTAAGTACGCCTGAAACTCTTCCTCACCTTCCACATTCCAAAATCTACCGGCTC
GTGTAAAGATGGTGAAGCTTATTTGTGCGATCGTTGGTCCGGCAGGAAACGCGTTTCCTGTGGACATCGATGCGGATCAGCTGGTGG
GAGACCTCAAGGACGCGATTAGGGCGAAGAATCCAGACACGATCAAGTGCGAGGCAGCTAAGTTGGAGCTCTTCCTGGCCATGACAG
ATGGCGGCGCTTGGTTATCAAGCCAATCAGATGACGTGAAGAAGCTTAAGAAGGGCGAGAAGACAGCTCTCATCAAATTGCTAATAA
AGGAGGATCAACAGCTACAGGCGGAGGACCCTTTGGAATACGTGCTGAGAGAAAACAATATGGCCACCCCACAGTCGAGACAGATTC
ACGTTCTGGTGGTGGTTCC
>PhCRN17.4_304 PLHAL3040986
GGTTTAGAAATTGCGTTACACCACCATGTTCGCTTAAGTACGCCTGAAACTCTTCCTCACCTTCCACATTCCAAAATCTACCGGCTC
GTGTAAAGATGGTGAAGCTTATTTGTGCGATCGTTGGTCCGGCAGGAAACGCGTTTCCTGTGGACATCGATGCGGATCAGCTGGTGG
GAGACCTCAAGGACGCGATTAGGGCGAAGAATCCAGACACGATCAAGTGCGAGGCAGCTAAGTTGGAGCTCTTCCTGGCCATAACAG
ATGGCGGCGCTTGGTTATCAAGCCAATCAGATGACGTGAAGAAGCTTAAGAAGGGCGAGAAGACAGCTCTCATCAAATTGCTAATAA
AGGAGGATCAACAGCTACAGGCGGAGGACCCTTTGGAATACGTGCTGAGAGAAAACAATATGGCCACCCCACAGTCGAGACAGATTC
ACGTTCTGGTGGTGGTTCC
>PhCRN17.4_700 PLHAL7002876
GGTTTAGAAATTGCGTTACACCACCATGTTCGCTTAAGTACGCCTGAAACTCTTCCTCACCTTCCACATTCCAAAATCTACCGGCTC
GTGTAAAGATGGTGAAGCTTATTTGTGCGATCGTTGGTCCGGCAGGAAACGCGTTTCCTGTGGACATCGATGCGGATCAGCTGGTGG
GAGACCTCAAGGACGCGATTAGGGCGAAGAATCCAGACACGATCAAGTGCGAGGCAGCTAAGTTGGAGCTCTTCCTGGCCATGACAG
ATGGCGGCGCTTGGTTATCAAGCCAATCAGATGACGTGAAGAAGCTTAAGAAGGGCGAGAAGACAGCTCTCATCAAATTGCTAATAA
AGGAGGATCAACAGCTACAGGCGGAGGACCCTTTGGAATACGTGCTGAGAGAAAACAATATGGCCACCCCACAGTCGAGACAGATTC
ACGTTCTGGTGGTGGTTCC
>PhCRN17.4_730 PLHAL7301376
GGTTTAGAAATTGCGTTACACCACCATGTTCGCTTAAGTACGCCTGAAACTCTTCCTCACCTTCCACATTCCAAAATCTACCGGCTC
GTGTAAAGATGGTGAAGCTTATTTGTGCGATCGTTGGTCCGGCAGGAAACGCGTTTCCTGTGGACATCGATGCGGATCAGCTGGTGG
GAGACCTCAAGGACGCGATTAGGGCGAAGAATCCAGACACGATCAAGTGCGAGGCAGCTAAGTTGGAGCTCTTCCTGGCCATGACAG
ATGGCGGCGCTTGGTTATCAAGCCAATCAGATGACGTGAAGAAGCTTAAGAAGGGCGAGAAGACAGCTCTCATCAAATTGCTAATAA
AGGAGGATCAACAGCTACAGGCGGAGGACCCTTTGGAATACGTGCTGAGAGAAAACAATATGGCCACCCCACAGTCGAGACAGATTC
ACGTTCTGGTGGTGGTTCC

>PhCRN18_100 PLHAL100A12060
ATTCCAGAATCAACCAGCTCGAGTAATGATGGTTAATCTGTTGTGTGCGATTGTTGGTGCGGCAAGTTATGCGTTTGAGGTGAACAT
TGACGATACTGCGTCAGTATCGGCTTTGAAGAAGGCAGTAAAAGCGGAGAATCCAGACACGATCAAGTGCGAAGCAGCTAAGTTGGA
GCTTTTTCTAGCCAAGACAGAGGGCGGCGCGTGGTTGGATTCTACCACCGACGATGTGAAGAAGCTGAAGAAGGGCGAAAAGACTGC
TTTGATCGAAGCTCTGACACATGAAGACAATGAACTTGATGGCACATTTGGCCTCGAAGACTATCTCGTGGGTATGCCAGAGCCGAA
AACCAGGCAAATCCACGTACTGGTGGTGATTCCGGCTGGTAATTCCGCTGACATTGGTGTAACTCCTTCCAGATTGCTGAAATATAA
GCGTACGTATTCTCGACCCAAGCTGTTTGAATATGTTGTTGCTAAAGGTTTTGTGCAGAGCAGGATACAGACGCTGGATCGTACGTG
CGCAATTCATCTCAATTCTTATATCGATTATCGCTACCATCATTGATGTGTCATACGGGCAGAATCCAACCGACGAAAAAGGATGTC
TAGCAGCTGATGTTCCGCATTTCGTGTGTTACTTGGCTGGTGTTTCTCTGGCGCTAATAGCTGTCCAAATATTTTTGAAACTTTGCA
AAATTAAGCTTGACGGTAAAATCAATGACGCTAGCGACCAGACACTTTATCAAGCCATTTAAGAAATGACCGACCTGACTTTTGTTG
CTGAAGCGCAGCACATTGCAGCTGTCTGTTTCTGAAGATCAGCGATAACCTCAACAACTCTTATAGGGCACAAAGAGAAACAGAGGA
AGAGAAATTAGGTAAATTAATGAAAGAATTGTGGCTTTAA
>PhCRN18_334 PLHAL3342172
ATTCCAGAATCAACCAGCTCGAGTAATGATGGTTAATCTGTTGTGTGCGATTGTTGGTGCGGCAAGTTATGCGTTTGAGGTGAACAT
TGACGATACTGCGTCAGTATCGGCTTTGAAGAAGGCAGTAAAAGCGGAGAATCCAGACACGATCAAGTGCGAAGCAGCTAAGTTGGA
GCTTTTTCTAGCCAAGACAGAGGGCGGCGCGTGGTTGGATTCTACCACCGACGATGTGAAGAAGCTGAAGAAGGGCGAAAAGACTGC
TTTGATCGAAGCTCTGACACATGAAGACAATGAACTTGATGGCACATTTGGCCTCGAAGACTATCTCGTGGGTATGCCAGAGCCGAA
AACCAGGCAAATCCACGTACTGGTGGTGATTCCGGCTGGTAATTCCGCTGACATTGGTGTAACTCCTTCCAGATTGCTGAAATATAA
GCGTACGTATTCTCGACCCAAGCTGTTTGAATATGTTGTTGCTAAAGGTTTTGTGCAGAGCAGGATACAGACGCTGGATCGTACGTG
CGCAATTCATCTCAATTCTTATATCGATTATCGCTACCATCATTGATGTGTCATACGGGCAGAATCCAACCGACGAAAAAGGATGTC
TAGCAGCTGATGTTCCGCATTTCGTGTGTTACTTGGCTGGTGTTTCTCTGGCGCTAATAGCTGTCCAAATATTTTTGAAACTTTGCA
AAATTAAGCTTGACGGTAAAATCAATGACGCTAGCGACCAGACACTTTATCAAGCCATTTAAGAAATGACCGACCTGACTTTTGTTG
CTGAAGCGCAGCACATTGCAGCTGTCTGTTTCTGAAGATCAGCGATAACCTCAACAACTCTTATAGGGCACAAAGAGAAACAGAGGA
AGAGAAATTAGGTAAATTAATGAAAGAATTGTGGCTTTAA
>PhCRN18_703 PLHAL7032034

ATTCCAGAATCAACCAGCTCGAGTAATGATGGTTAATCTGTTGTGTGCGATTGTTGGTGCGGCAAGTTATGCGTTTGAGGTGAACAT
TGACGATACTGCGTCAGTATCGGCTTTGAAGAAGGCAGTAAAAGCGGAGAATCCAGACACGATCAAGTGCGAAGCAGCTAAGTTGGA
GCTTTTTCTAGCCAAGACAGAGGGCGGCGCGTGGTTGGATTCTACCACCGACGATGTGAAGAAGCTGAAGAAGGGCGAAAAGACTGC
TTTGATCGAAGCTCTGACACATGAAGACAATGAACTTGATGGCACATTTGGCCTCGAAGACTATCTCGTGGGTATGCCAGAGCCGAA
AACCAGGCAAATCCACGTACTGGTGGTGATTCCGGCTGGTAATTCCGCTGACATTGGTGTAACTCCTTCCAGATTGCTGAAATATAA
GCGTACGTATTCTCGACCCAAGCTGTTTGAATATGTTGTTGCTAAAGGTTTTGTGCAGAGCAGGATACAGACGCTGGATCGTACGTG
CGCAATTCATCTCAATTCTTATATCGATTATCGCTACCATCATTGATGTGTCATACGGGCAGAATCCAACCGACGAAAAAGGATGTC
TAGCAGCTGATGTTCCGCATTTCGTGTGTTACTTGGCTGGTGTTTCTCTGGCGCTAATAGCTGTCCAAATATTTTTGAAACTTTGCA
AAATTAAGCTTGACGGTAAAATCAATGACGCTAGCGACCAGACACTTTATCAAGCCATTTAAGAAATGACCGACCTGACTTTTGTTG
CTGAAGCGCAGCACATTGCAGCTGTCTGTTTCTGAAGATCAGCGATAACCTCAACAACTCTTATAGGGCACAAAGAGAAACAGAGGA
AGAGAAATTAGGTAAATTAATGAAAGAATTGTGGCTTTAA
>PhCRN18_710 PLHAL7101728
ATTCCAGAATCAACCAGCTCGAGTAATGATGGTTAATCTGTTGTGTGCGATTGTTGGTGCGGCAAGTTATGCGTTTGAGGTGAACAT
TGACGATACTGCGTCAGTATCGGCTTTGAAGAAGGCAGTAAAAGCGGAGAATCCAGACACGATCAAGTGCGAAGCAGCTAAGTTGGA
GCTTTTTCTAGCCAAGACAGAGGGCGGCGCGTGGTTGGATTCTACCACCGACGATGTGAAGAAGCTGAAGAAGGGCGAAAAGACTGC
TTTGATCGAAGCTCTGACACATGAAGACAATGAACTTGATGGCACATTTGGCCTCGAAGACTATCTCGTGGGTATGCCAGAGCCGAA
AACCAGGCAAATCCACGTACTGGTGGTGATTCCGGCTGGTAATTCCGCTGACATTGGTGTAACTCCTTCCAGATTGCTGAAATATAA
GCGTACGTATTCTCGACCCAAGCTGTTTGAATATGTTGTTGCTAAAGGTTTTGTGCAGAGCAGGATACAGACGCTGGATCGTACGTG
CGCAATTCATCTCAATTCTTATATCGATTATCGCTACCATCATTGATGTGTCATACGGGCAGAATCCAACCGACGAAAAAGGATGTC
TAGCAGCTGATGTTCCGCATTTCGTGTGTTACTTGGCTGGTGTTTCTCTGGCGCTAATAGCTGTCCAAATATTTTTGAAACTTTGCA
AAATTAAGCTTGACGGTAAAATCAATGACGCTAGCGACCAGACACTTTATCAAGCCATTTAAGAAATGACCGACCTGACTTTTGTTG
CTGAAGCGCAGCACATTGCAGCTGTCTGTTTCTGAAGATCAGCGATAACCTCAACAACTCTTATAGGGCACAAAGAGAAACAGAGGA
AGAGAAATTAGGTAAATTAATGAAAGAATTGTGGCTTTAA
>PhCRN18_304 PLHAL3042063
ATTCCAGAATCAACCAGCTCGAGTAATGATGGTTAATCTGTTGTGTGCGATTGTTGGTGCGGCAAGTTATGCGTTTGAGGTGAACAT
TGACGATACTGCGTCAGTATCGGCTTTGAAGAAGGCAGTAAAAGCGGAGAATCCAGACACGATCAAGTGCGAAGCAGCTAAGTTGGA
GCTTTTTCTAGCCAAGACAGAGGGCGGCGCGTGGTTGGATTCTACCACCGACGATGTGAAGAAGCTGAAGAAGGGCGAAAAGACTGC
TTTGATCGAAGCTCTGACACATGAAGACAATGAACTTGATGGCACATTTGGCCTCGAAGACTATCTCGTGGGTATGCCAGAGCCGAA
AACCAGGCAAATCCACGTACTGGTGGTGATTCCGGCTGGTAATTCCGCTGACATTGGTGTAACTCCTTCCAGATTGCTGAAATATAA
GCGTACGTATTCTCGACCCAAGCTGTTTGAATATGTTGTTGCTAAAGGTTTTGTGCAGAGCAGGATACAGACGCTGGATCGTACGTG
CGCAATTCATCTCAATTCTTATATCGATTATCGCTACCATCATTGATGTGTCATACGGGCAGAATCCAACCGACGAAAAAGGATGTC
TAGCAGCTGATGTTCCGCATTTCGTGTGTTACTTGGCTGGTGTTTCTCTGGCGCTAATAGCTGTCCAAATATTTTTGAAACTTTGCA
AAATTAAGCTTGACGGTAAAATCAATGACGCTAGCGACCAGACACTTTATCAAGCCATTTAAGAAATGACCGACCTGACTTTTGTTG
CTGAAGCGCAGCACATTGCAGCTGTCTGTTTCTGAAGATCAGCGATAACCTCAACAACTCTTATAGGGCACAAAGAGAAACAGAGGA
AGAGAAATTAGGTAAATTAATGAAAGAATTGTGGCTTTAA
>PhCRN18_700 PLHAL7002549
ATTCCAGAATCAACCAGCTCGAGTAATGATGGTTAATCTGTTGTGTGCGATTGTTGGTGCGGCAAGTTATGCGTTTGAGGTGAACAT
TGACGATACTGCGTCAGTATCGGCTTTGAAGAAGGCAGTAAAAGCGGAGAATCCAGACACGATCAAGTGCGAAGCAGCTAAGTTGGA
GCTTTTTCTAGCCAAGACAGAGGGCGGCGCGTGGTTGGATTCTACCACCGACGATGTGAAGAAGCTGAAGAAGGGCGAAAAGACTGC
TTTGATCGAAGCTCTGACACATGAAGACAATGAACTTGATGGCACATTTGGCCTCGAAGACTATCTCGTGGGTATGCCAGAGCCGAA
AACCAGGCAAATCCACGTACTGGTGGTGATTCCGGCTGGTAATTCCGCTGACATTGGTGTAACTCCTTCCAGATTGCTGAAATATAA
GCGTACGTATTCTCGACCCAAGCTGTTTGAATATGTTGTTGCTAAAGGTTTTGTGCAGAGCAGGATACAGACGCTGGATCGTACGTG
CGCAATTCATCTCAATTCTTATATCGATTATCGCTACCATCATTGATGTGTCATACGGGCAGAATCCAACCGACGAAAAAGGATGTC
TAGCAGCTGATGTTCCGCATTTCGTGTGTTACTTGGCTGGTGTTTCTCTGGCGCTAATAGCTGTCCAAATATTTTTGAAACTTTGCA
AAATTAAGCTTGACGGTAAAATCAATGACGCTAGCGACCAGACACTTTATCAAGCCATTTAAGAAATGACCGACCTGACTTTTGTTG
CTGAAGCGCAGCACATTGCAGCTGTCTGTTTCTGAAGATCAGCGATAACCTCAACAACTCTTATAGGGCACAAAGAGAAACAGAGGA
AGAGAAATTAGGTAAATTAATGAAAGAATTGTGGCTTTAA
>PhCRN18_730 PLHAL7302011
ATTCCAGAATCAACCAGCTCGAGTAATGATGGTTAATCTGTTGTGTGCGATTGTTGGTGCGGCAAGTTATGCGTTTGAGGTGAACAT
TGACGATACTGCGTCAGTATCGGCTTTGAAGAAGGCAGTAAAAGCGGAGAATCCAGACACGATCAAGTGCGAAGCAGCTAAGTTGGA
GCTTTTTCTAGCCAAGACAGAGGGCGGCGCGTGGTTGGATTCTACCACCGACGATGTGAAGAAGCTGAAGAAGGGCGAAAAGACTGC
TTTGATCGAAGCTCTGACACATGAAGACAATGAACTTGATGGCACATTTGGCCTCGAAGACTATCTCGTGGGTATGCCAGAGCCGAA
AACCAGGCAAATCCACGTACTGGTGGTGATTCCGGCTGGTAATTCCGCTGACATTGGTGTAACTCCTTCCAGATTGCTGAAATATAA
GCGTACGTATTCTCGACCCAAGCTGTTTGAATATGTTGTTGCTAAAGGTTTTGTGCAGAGCAGGATACAGACGCTGGATCGTACGTG
CGCAATTCATCTCAATTCTTATATCGATTATCGCTACCATCATTGATGTGTCATACGGGCAGAATCCAACCGACGAAAAAGGATGTC
TAGCAGCTGATGTTCCGCATTTCGTGTGTTACTTGGCTGGTGTTTCTCTGGCGCTAATAGCTGTCCAAATATTTTTGAAACTTTGCA
AAATTAAGCTTGACGGTAAAATCAATGACGCTAGCGACCAGACACTTTATCAAGCCATTTAAGAAATGACCGACCTGACTTTTGTTG
CTGAAGCGCAGCACATTGCAGCTGTCTGTTTCTGAAGATCAGCGATAACCTCAACAACTCTTATAGGGCACAAAGAGAAACAGAGGA
AGAGAAATTAGGTAAATTAATGAAAGAATTGTGGCTTTAA

>PhCRN19_100 PLHAL100A12404
GGTGTCATTCAACCACGCAAGTCTATGTGACACGCCTTCATTTCCGCTTAGATACGAACAGAAATTTTCTCCCTTTCATTTTCATTT
CCGAATCCGCCGGCTCGAAGAAAGTTGTTGACGCTGTACTGTGCTGTTGTTGGTGAGGTGGGAAGCGCGTTTCCCGTGGACATCGAT
GCGAGCCTTTCGGTGGGCCATTTGAAAGATGCGATCAAGAATAAGAACTTGGATGACCCAACTTTAAGGAATGTCGCTCCGAAGAAC
CTGCAGCTATTTCTGGCGAAGAAAGACAAGGGCCATGGCGCGTGGGTGACGGAAGAAGAAGCGATGAGGGGTGTGGATGACACAACT
GATCTGAAATTACTAAAATCAGCGCGAGCGGCAATCGGTGATGTTGGGCTTTCAGAAGATGAAGTGCGGATTCAAGTGAGCAAAGAA
GAAGTGGAGGATCTGAAGGGCCCTGTTCACGTGCTGGTGGTGGTTCCGACCGAAGTAGCTGTAGCTCCACCAAGAAAGCGAGTTAAA

ATGTTGCCAGAAGAAGTTAAGCCCCCAAGTTTGTTCGCTAATCGCCGTGTCTGGTCGGCTTTTTTGATGGAAGATGGTCGCATCATT
TGCGATCGAGTTGATCGCCCTGAGAACGTCCGTCCCTTGACGTTGCTCATCCCATCTTTAGCGAGTTTGTGGGTGGGTGTCAAGGAA
AGATTGAAATAACCGGTGAAGACGTGACTTTTGCAGAAAATGTTGCAAA
>PhCRN19_334 PLHAL3342408
GTTTCAAAGCTTCTTCAACAACAGCTGCGGTTTTTAAAGACGCTATCTTTAGACGCCCTCTGGTGCGAGGAAGACCTCCTTCGTACC
TTCAAACACCGCACATAGCAACAGTGGGATTATATGCAAAGGAGGATTTATTCGAAGCGAAAATCATGCTAACCTCGTTGAGCGCAG
CACATTACGTCGGCATTTTTTCTTATCGCCAGGTGTCATTCAACCACGCAAGTCTATGTGACACGCCTTCATTTCCGCTTAGATACG
AACAGAAATTTTCTCCCTTTCATTTTCATTTCCGAATCCGCCGGCTCGAAGAAAGTTGTTGACGCTGTACTGTGCTGTTGTTGGTGA
GGTGGGAAGCGCGTTTCCCGTGGACATCGATGCGAGCCTTTCGGTGGGCCATTTGAAAGATGCGATCAAGAATAAGAACTTGGATGA
CCCAACTTTAAGGAATGTCGCTCCGAAGAACCTGCAGCTATTTCTGGCGAAGAAAGACAAGGGCCATGGCGCGTGGGTGACGGAAGA
AGAAGCGATGAGGGGTGTGGATGACACAACTGATCTGAAATTACTAAAATCAGCGCGAGCGGCAATCGGTGATGTTGGGCTTTCAGA
AGATGAAGTGCGGATTCAAGTGAGCAAAGAAGAAGTGGAGGATCTGAAGGGCCCTGTTCACGTGCTGGTGGTGGTTCCGACCGAAGT
AGCTGTAGCTCCACCAAGAAAGCGAGTTAAAATGTTGCCAGAAGAAGTTAAGCCCCCAAGTTTGTTCGCTAATCGCCGTGTCTGGTC
GGCTTTTTTGATGGAAGATGGTCGCATCATTTGCGATCGAGTTGATCGCCCTGAGAACGTCCGTCCCTTGACGTTGCTCATCCCATC
TTTAGCGAGTTTGTGGGTGGGTGTCAAGGAAAGATTGAAATAACCGGTGAAGACGTGACTTTTGCAGAAAATGTTGCAAA
>PhCRN19_703 PLHAL7032149
GTTTCAAAGCTTCTTCAACAACAGCTGCGGTTTTTAAAGACGCTATCTTTAGACGCCCTCTGGTGCGAGGAAGACCTCCTTCGTACC
TTCAAACACCGCACATAGCAACAGTGGGATTATATGCAAAGGAGGATTTATTCGAAGCGAAAATCATGCTAACCTCGTTGAGCGCAG
CACATTACGTCGGCATTTTTTCTTATCGCCAGGTGTCATTCAACCACGCAAGTCTATGTGACACGCCTTCATTTCCGCTTAGATACG
AACAGAAATTTTCTCCCTTTCATTTTCATTTCCGAATCCGCCGGCTCGAAGAAAGTTGTTGACGCTGTACTGTGCTGTTGTTGGTGA
GGTGGGAAGCGCGTTTCCCGTGGACATCGATGCGAGCCTTTCGGTGGGCCATTTGAAAGATGCGATCAAGAATAAGAACTTGGATGA
CCCAACTTTAAGGAATGTCGCTCCGAAGAACCTGCAGCTATTTCTGGCGAAGAAAGACAAGGGCCATGGCGCGTGGGTGACGGAAGA
AGAAGCGATGAGGGGTGTGGATGACACAACTGATCTGAAATTACTAAAATCAGCGCGAGCGGCAATCGGTGATGTTGGGCTTTCAGA
AGATGAAGTGCGGATTCAAGTGAGCAAAGAAGAAGTGGAGGATCTGAAGGGCCCTGTTCACGTGCTGGTGGTGGTTCCGACCGAAGT
AGCTGTAGCTCCACCAAGAAAGCGAGTTAAAATGTTGCCAGAAGAAGTTAAGCCCCCAAGTTTGTTCGCTAATCGCCGTGTCTGGTC
GGCTTTTTTGATGGAAGATGGTCGCATCATTTGCGATCGAGTTGATCGCCCTGAGAACGTCCGTCCCTTGACGTTGCTCATCCCATC
TTTAGCGAGTTTGTGGGTGGGTGTCAAGGAAAGATTGAAATAACCGGTGAAGACGTGACTTTTGCAGAAAATGTTGCAAA
>PhCRN19_710 PLHAL7101681
GTTTCAAAGCTTCTTCAACAACAGCTGCGGTTTTTAAAGACGCTATCTTTAGACGCCCTCTGGTGCGAGGAAGACCTCCTTCGTACC
TTCAAACACCGCACATAGCAACAGTGGGATTATATGCAAAGGAGGATTTATTCGAAGCGAAAATCATGCTAACCTCGTTGAGCGCAG
CACATTACGTCGGCATTTTTTCTTATCGCCAGGTGTCATTCAACCACGCAAGTCTATGTGACACGCCTTCATTTCCGCTTAGATACG
AACAGAAATTTTCTCCCTTTCATTTTCATTTCCGAATCCGCCGGCTCGAAGAAAGTTGTTGACGCTGTACTGTGCTGTTGTTGGTGA
GGTGGGAAGCGCGTTTCCCGTGGACATCGATGCGAGCCTTTCGGTGGGCCATTTGAAAGATGCGATCAAGAATAAGAACTTGGATGA
CCCAACTTTAAGGAATGTCGCTCCGAAGAACCTGCAGCTATTTCTGGCGAAGAAAGACAAGGGCCATGGCGCGTGGGTGACGGAAGA
AGAAGCGATGAGGGGTGTGGATGACACAACTGATCTGAAATTACTAAAATCAGCGCGAGCGGCAATCGGTGATGTTGGGCTTTCAGA
AGATGAAGTGCGGATTCAAGTGAGCAAAGAAGAAGTGGAGGATCTGAAGGGCCCTGTTCACGTGCTGGTGGTGGTTCCGACCGAAGT
AGCTGTAGCTCCACCAAGAAAGCGAGTTAAAATGTTGCCAGAAGAAGTTAAGCCCCCAAGTTTGTTCGCTAATCGCCGTGTCTGGTC
GGCTTTTTTGATGGAAGATGGTCGCATCATTTGCGATCGAGTTGATCGCCCTGAGAACGTCCGTCCCTTGACGTTGCTCATCCCATC
TTTAGCGAGTTTGTGGGTGGGTGTCAAGGAAAGATTGAAATAACCGGTGAAGACGTGACTTTTGCAGAAAATGTTGCAAA
>PhCRN19_304 PLHAL3042351
GTTTCAAAGCTTCTTCAACAACAGCTGCGGTTTTTAAAGACGCTATCTTTAGACGCCCTCTGGTGCGAGGAAGACCTCCTTCGTACC
TTCAAACACCGCACATAGCAACAGTGGGATTATATGCAAAGGAGGATTTATTCGAAGCGAAAATCATGCTAACCTCGTTGAGCGCAG
CACATTACGTCGGCATTTTTTCTTATCGCCAGGTGTCATTCAACCACGCAAGTCTATGTGACACGCCTTCATTTCCGCTTAGATACG
AACAGAAATTTTCTCCCTTTCATTTTCATTTCCGAATCCGCCGGCTCGAAGAAAGTTGTTGACGCTGTACTGTGCTGTTGTTGGTGA
GGTGGGAAGCGCGTTTCCCGTGGACATCGATGCGAGCCTTTCGGTGGGCCATTTGAAAGATGCGATCAAGAATAAGAACTTGGATGA
CCCAACTTTAAGGAATGTCGCTCCGAAGAACCTGCAGCTATTTCTGGCGAAGAAAGACAAGGGCCATGGCGCGTGGGTGACGGAAGA
AGAAGCGATGAGGGGTGTGGATGACACAACTGATCTGAAATTACTAAAATCAGCGCGAGCGGCAATCGGTGATGTTGGGCTTTCAGA
AGATGAAGTGCGGATTCAAGTGAGCAAAGAAGAAGTGGAGGATCTGAAGGGCCCTGTTCACGTGCTGGTGGTGGTTCCGACCGAAGT
AGCTGTAGCTCCACCAAGAAAGCGAGTTAAAATGTTGCCAGAAGAAGTTAAGCCCCCAAGTTTGTTCGCTAATCGCCGTGTCTGGTC
GGCTTTTTTGATGGAAGATGGTCGCATCATTTGCGATCGAGTTGATCGCCCTGAGAACGTCCGTCCCTTGACGTTGCTCATCCCATC
TTTAGCGAGTTTGTGGGTGGGTGTCAAGGAAAGATTGAAATAACCGGTGAAGACGTGACTTTTGCAGAAAATGTTGCAAA
>PhCRN19_700 PLHAL7002856
GTTTCAAAGCTTCTTCAACAACAGCTGCGGTTTTTAAAGACGCTATCTTTAGACGCCCTCTGGTGCGAGGAAGACCTCCTTCGTACC
TTCAAACACCGCACATAGCAACAGTGGGATTATATGCAAAGGAGGATTTATTCGAAGCGAAAATCATGCTAACCTCGTTGAGCGCAG
CACATTACGTCGGCATTTTTTCTTATCGCCAGGTGTCATTCAACCACGCAAGTCTATGTGACACGCCTTCATTTCCGCTTAGATACG
AACAGAAATTTTCTCCCTTTCATTTTCATTTCCGAATCCGCCGGCTCGAAGAAAGTTGTTGACGCTGTACTGTGCTGTTGTTGGTGA
GGTGGGAAGCGCGTTTCCCGTGGACATCGATGCGAGCCTTTCGGTGGGCCATTTGAAAGATGCGATCAAGAATAAGAACTTGGATGA
CCCAACTTTAAGGAATGTCGCTCCGAAGAACCTGCAGCTATTTCTGGCGAAGAAAGACAAGGGCCATGGCGCGTGGGTGACGGAAGA
AGAAGCGATGAGGGGTGTGGATGACACAACTGATCTGAAATTACTAAAATCAGCGCGAGCGGCAATCGGTGATGTTGGGCTTTCAGA
AGATGAAGTGCGGATTCAAGTGAGCAAAGAAGAAGTGGAGGATCTGAAGGGCCCTGTTCACGTGCTGGTGGTGGTTCCGACCGAAGT
AGCTGTAGCTCCACCAAGAAAGCGAGTTAAAATGTTGCCAGAAGAAGTTAAGCCCCCAAGTTTGTTCGCTAATCGCCGTGTCTGGTC
GGCTTTTTTGATGGAAGATGGTCGCATCATTTGCGATCGAGTTGATCGCCCTGAGAACGTCCGTCCCTTGACGTTGCTCATCCCATC
TTTAGCGAGTTTGTGGGTGGGTGTCAAGGAAAGATTGAAATAACCGGTGAAGACGTGACTTTTGCAGAAAATGTTGCAAA
>PhCRN19_730 PLHAL7302253
GTTTCAAAGCTTCTTCAACAACAGCTGCGGTTTTTAAAGACGCTATCTTTAGACGCCCTCTGGTGCGAGGAAGACCTCCTTCGTACC
TTCAAACACCGCACATAGCAACAGTGGGATTATATGCAAAGGAGGATTTATTCGAAGCGAAAATCATGCTAACCTCGTTGAGCGCAG
CACATTACGTCGGCATTTTTTCTTATCGCCAGGTGTCATTCAACCACGCAAGTCTATGTGACACGCCTTCATTTCCGCTTAGATACG

AACAGAAATTTTCTCCCTTTCATTTTCATTTCCGAATCCGCCGGCTCGAAGAAAGTTGTTGACGCTGTACTGTGCTGTTGTTGGTGA
GGTGGGAAGCGCGTTTCCCGTGGACATCGATGCGAGCCTTTCGGTGGGCCATTTGAAAGATGCGATCAAGAATAAGAACTTGGATGA
CCCAACTTTAAGGAATGTCGCTCCGAAGAACCTGCAGCTATTTCTGGCGAAGAAAGACAAGGGCCATGGCGCGTGGGTGACGGAAGA
AGAAGCGATGAGGGGTGTGGATGACACAACTGATCTGAAATTACTAAAATCAGCGCGAGCGGCAATCGGTGATGTTGGGCTTTCAGA
AGATGAAGTGCGGATTCAAGTGAGCAAAGAAGAAGTGGAGGATCTGAAGGGCCCTGTTCACGTGCTGGTGGTGGTTCCGACCGAAGT
AGCTGTAGCTCCACCAAGAAAGCGAGTTAAAATGTTGCCAGAAGAAGTTAAGCCCCCAAGTTTGTTCGCTAATCGCCGTGTCTGGTC
GGCTTTTTTGATGGAAGATGGTCGCATCATTTGCGATCGAGTTGATCGCCCTGAGAACGTCCGTCCCTTGACGTTGCTCATCCCATC
TTTAGCGAGTTTGTGGGTGGGTGTCAAGGAAAGATTGAAATAACCGGTGAAGACGTGACTTTTGCAGAAAATGTTGCAAA

>PhCRN20_100 PLHAL100A12505
CCGGACAACGAAGACCTGGACACGTTGCTTCAGAGCGAAATCGACACTTCATCGTATTTGCACATGCGTGCGTCGTGGAAGCTAAGC
AAGCCGAACCTTTTCGGGCCTGACGTTTCGCTAGGAGAGGACGTAGTTCACGTGCTGGTGGTGGTTCCAGAGGGTGCTGTTGGTTCA
GCATTATTGCAGCCAGCGAATAGCGCTACAATTCAAAATATCGCCTGGTATGGCACGCGTGGATCGATTGTGGAAGGAGAAGGCGTT
GATCTGAAAGATCCACGAACACTTTCTCGAGCCATTCTCACCGCGGATATCCTCCGCCGACTCGAAGAAACGCATGTCCTGCTGGTG
AAAAGCCCACCAATGACTGGGAAAACGTCACTAGCGACACTTGTGAGCCGATCGTTGGTGGATAGGCATATTATGGACAATAAGAAG
ATGACTCTGTTCAACTTCTCCGCCTTGGCCATACCCGACGATTCGACATTTGAGCAAGTTTTCAAGAAGCGTTGTACGATCGACTGG
AGCGAAGCAACTTTAAATCTTCCCACGCCAGATCGTATGGTGTATTTGGTGGTGGACGAAGTACAAGTGATCTACAAAGAAGGAACA
AATCCTCCGAGACGCAAATCCACTGTTTTTTGGGAGCTGGTCAAGTACGTTCTCAGCAACGGGAACTACAGCATCAGAATATTGATG
TTTGCAGCGTATGGATCTGGTGTGGAGTACACGCGACTCGCTACTCCAATACAGTTCGACGATAGGATTGTGTTGGGCATCGACCAG
CTGAAATTTAGTCATGCCGAAGTCTCTGAGTACGTCCAAAAGTGGTTTAAGGGTATCACGTGCTTTGAAGGATCATCGTCATCTGCG
ATGAAGGATTTCTGTGCCAATTTGGAAGAGGTGACGGGTAGGCACGTTGGTCTGTGTGCGACTACCATCTTTGAACTGAATAGAGTC
CATGCCTCACGTAATAGGAGTGCGAGTCGTCAACCATTGCCAGCCGAGTGGATCCGCATGCTGCAAAGTGGGTCACTGTATGAAGCC
AACGATAAAGCGCTGTTTAAAGCGCTGACATCAACACGCGCTGTGAAGGTGTTAAATACCCTTGACAAGGACGAGCTGGATCGGCTA
GAACGCATTGCTTATGGTGCCAACTCTGATTTTGATACTGATATAGTTGAGCAATGTCTTCGAAAGGGCATTCTTGTTCAAACTGAG
CGGCGTTTTGAGTTCTCGTCACCAGTGATGTGGCGATTCTTTGTCAAGATGCGCGTTGGTCACATCGTCCGAGCACTTCATGTCCCC
AAAACCCTTCCAGAGATGGTCGCTCGAGTGATGCGATCGATCGATTATGATAGCATCCGCCAGACGCTTGGCAGAAGTTTGTCGAGC
GATATTCCTCTGGAAAGAGCGTGGCAAATGGAATTTTACAAGGCTGCGTATCGCTGCACACCAAGCACTTTCGTGACGTCTGCGGAT
GTGGGTGCGTTGTTTGGATCCAGTGGGTTCGTCGATTTTACAATTCATGGAGGTGACATCTTTTGGGGCATCGAGTTGTTGCGAGAA
GCCAGCAACTTGGCTGAACACATCAAACGATTTTCCCCTGGTGGTCGCTATTCCTCGCTCCCGTTGACCGAGTTCTGTCTGGTTGAC
TTTAGACGTGTTGCTTCGATTGACGATGTGCCGATTGAACGTATAGCGGAAAATATGCGCGACTGCGACAAGCTCTTCGTCGTGTGT
TATGACGCACGGATGGCAGGTGTTATGGTGATTAATTCCGCAATGGATGTCGTCTACAGGACTCAATCTTAAAAAAGTTTTGTGATC
TTGCCAATTTGGTTGTTGCATTTGAAAACTTGATGCCATTGATGAAGAAAATAAGGTGAACTTTTTGAGGTCTACCGCATCGATTAA
TTTTTCATTCTAACAAATATAAAGTTAGTAATATACTTAGATGG
>PhCRN20_334 PLHAL3341451
CCGGACAACGAAGACCTGGACACGTTGCTTCAGAGCGAAATCGACACTTCATCGTATTTGCACATGCGTGCGTCGTGGAAGCTAAGC
AAGCCGAACCTTTTCGGGCCTGACGTTTCGCTAGGAGAGGACGTAGTTCACGTGCTGGTGGTGGTTCCAGAGGGTGCTGTTGGTTCA
GCATTATTGCAGCCAGCGAATAGCGCTACAATTCAAAATATCGCCTGGTATGGCACGCGTGGATCGATTGTGGAAGGAGAAGGCGTT
GATCTGAAAGATCCACGAACACTTTCTCGAGCCATTCTCACCGCGGATATCCTCCGCCGACTCGAAGAAACGCATGTCCTGCTGGTG
ACAAGCCCACCAATGACTGGGAAAACGTCACTAGCGACACTTGTGAGCCGATCGTTGGTGGATAGGCATATTATGGACAATAAGAAG
ATGACTCTGTTCAACTTCTCCGCCTTGGCCATACCCGACGATTCGACATTTGAGCAAGTTTTCAAGAAGCGTTGTACGATCGACTGG
AGCGAAGCAACTTTAAATCTTCCCACGCCAGATCGTATGGTGTATTTGGTGGTGGACGAAGTACAAGTGATCTACAAAGAAGGAACA
AATCCTCCGAGACGCAAATCCACTGTTTTTTGGGAGCTGGTCAAGTACGTTCTCAGCAACGGGAACTACAGCATCAGAATATTGATG
TTTGCAGCGTATGGATCTGGTGTGGAGTACACGCGACTCGCTACTCCAATACAGTTCGACGATAGGATTGTGTTGGGCATCGACCAG
CTGAAATTTAGTCATGCCGAAGTCTCTGAGTACGTCCAAAAGTGGTTTAAGGGTATCACGTGCTTTGAAGGATCATCGTCATCTGCG
ATGAAGGATTTCTGTGCCAATTTGGAAGAGGTGACGGGTAGGCACGTTGGTCTGTGTGCGACTACCATCTTTGAACTGAATAGAGTC
CATGCCTCACGTAATAGGAGTGCGAGTCGTCAACCATTGCCAGCCGAGTGGATCCGCATGCTGCAAAGTGGGTCACTGTATGAAGCC
AACGATAAAGCGCTGTTTAAAGCGCTGACATCAACACGCGCTGTGAAGGTGTTAAATACCCTTGACAAGGACGAGCTGGATCGGCTA
GAACGCATTGCTTATGGTGCCAACTCTGATTTTGATACTGATATAGTTGAGCAATGTCTTCGAAAGGGCATTCTTGTTCAAACTGAG
CGGCGTTTTGAGTTCTCGTCACCAGTGATGTGGCGATTCTTTGTCAAGATGCGCGTTGGTCACATCGTCCGAGCACTTCATGTCCCC
AAAACCCTTCCAGAGATGGTCGCTCGAGTGATGCGATCGATCGATTATGATAGCATCCGCCAGACGCTTGGCAGAAGTTTGTCGAGC
GATATTCCTCTGGAAAGAGCGTGGCAAATGGAATTTTACAAGGCTGCGTATCGCTGCACACCAAGCACTTTCGTGACGTCTGCGGAT
GTGGGTGCGTTGTTTGGATCCAGTGGGTTCGTCGATTTTACAATTCATGGAGGTGACATCTTTTGGGGCATCGAGTTGTTGCGAGAA
GCCAGCAACTTGGCTGAACACATCAAACGATTTTCCCCTGGTGGTCGCTATTCCTCGCTCCCGTTGACCGAGTTCTGTCTGGTTGAC
TTTAGACGTGTTGCTTCGATTGACGATGTGCCGATTGAACGTATAGCGGAAAATATGCGCGACTGCGACAAGCTCTTCGTCGTGTGT
TATGACGCACGGATGGCAGGTGTTATGGTGATTAATTCCGCAATGGATGTCGTCTACAGGACTCAATCTTAAAAAAGTTTTGTGATC
TTGCCAATTTGGTTGTTGCATTTGAAAACTTGATGCCATTGATGAAGAAAATAAGGTGAACTTTTTGAGGTCTACCGCATCGATTAA
TTTTTCATTCTAACAAATATAAAGTTAGTAATATACTTAGATGG
>PhCRN20_703 PLHAL7032332
CCGGACAACGAAGACCTGGACACGTTGCTTCAGAGCGAAATCGACACTTCATCGTATTTGCACATGCGTGCGTCGTGGAAGCTAAGC
AAGCCGAACCTTTTCGGGCCTGACGTTTCGCTAGGAGAGGACGTAGTTCACGTGCTGGTGGTGGTTCCAGAGGGTGCTGTTGGTTCA
GCATTATTGCAGCCAGCGAATAGCGCTACAATTCAAAATATCGCCTGGTATGGCACGCGTGGATCGATTGTGGAAGGAGAAGGCGTT
GATCTGAAAGATCCACGAACACTTTCTCGAGCCATTCTCACCGCGGATATCCTCCGCCGACTCGAAGAAACGCATGTCCTGCTGGTG
AAAAGCCCACCAATGACTGGGAAAACGTCACTAGCGACACTTGTGAGCCGATCGTTGGTGGATAGGCATATTATGGACAATAAGAAG
ATGACTCTGTTCAACTTCTCCGCCTTGGCCATACCCGACGATTCGACATTTGAGCAAGTTTTCAAGAAGCGTTGTACGATCGACTGG
AGCGAAGCAACTTTAAATCTTCCCACGCCAGATCGTATGGTGTATTTGGTGGTGGACGAAGTACAAGTGATCTACAAAGAAGGAACA
AATCCTCCGAGACGCAAATCCACTGTTTTTTGGGAGCTGGTCAAGTACGTTCTCAGCAACGGGAACTACAGCATCAGAATATTGATG
TTTGCAGCGTATGGATCTGGTGTGGAGTACACGCGACTCGCTACTCCAATACAGTTCGACGATAGGATTGTGTTGGGCATCGACCAG

CTGAAATTTAGTCATGCCGAAGTCTCTGAGTACGTCCAAAAGTGGTTTAAGGGTATCACGTGCTTTGAAGGATCATCGTCATCTGCG
ATGAAGGATTTCTGTGCCAATTTGGAAGAGGTGACGGGTAGGCACGTTGGTCTGTGTGCGACTACCATCTTTGAACTGAATAGAGTC
CATGCCTCACGTAATAGGAGTGCGAGTCGTCAACCATTGCCAGCCGAGTGGATCCGCATGCTGCAAAGTGGGTCACTGTATGAAGCC
AACGATAAAGCGCTGTTTAAAGCGCTGACATCAACACGCGCTGTGAAGGTGTTAAATACCCTTGACAAGGACGAGCTGGATCGGCTA
GAACGCATTGCTTATGGTGCCAACTCTGATTTTGATACTGATATAGTTGAGCAATGTCTTCGAAAGGGCATTCTTGTTCAAACTGAG
CGGCGTTTTGAGTTCTCGTCACCAGTGATGTGGCGATTCTTTGTCAAGATGCGCGTTGGTCACATCGTCCGAGCACTTCATGTCCCC
AAAACCCTTCCAGAGATGGTCGCTCGAGTGATGCGATCGATCGATTATGATAGCATCCGCCAGACGCTTGGCAGAAGTTTGTCGAGC
GATATTCCTCTGGAAAGAGCGTGGCAAATGGAATTTTACAAGGCTGCGTATCGCTGCACACCAAGCACTTTCGTGACGTCTGCGGAT
GTGGGTGCGTTGTTTGGATCCAGTGGGTTCGTCGATTTTACAATTCATGGAGGTGACATCTTTTGGGGCATCGAGTTGTTGCGAGAA
GCCAGCAACTTGGCTGAACACATCAAACGATTTTCCCCTGGTGGTCGCTATTCCTCGCTCCCGTTGACCGAGTTCTGTCTGGTTGAC
TTTAGACGTGTTGCTTCGATTGACGATGTGCCGATTGAACGTATAGCGGAAAATATGCGCGACTGCGACAAGCTCTTCGTCGTGTGT
TATGACGCACGGATGGCAGGTGTTATGGTGATTAATTCCGCAATGGATGTCGTCTACAGGACTCAATCTTAAAAAAGTTTTGTGATC
TTGCCAATTTGGTTGTTGCATTTGAAAACTTGATGCCATTGATGAAGAAAATAAGGTGAACTTTTTGAGGTCTACCGCATCGATTAA
TTTTTCATTCTAACAAATATAAAGTTAGTAATATACTTAGATGG
>PhCRN20_710 PLHAL7102214
CCGGACAACGAAGACCTGGACACGTTGCTTCAGAGCGAAATCGACACTTCATCGTATTTGCACATGCGTGCGTCGTGGAAGCTAAGC
AAGCCGAACCTTTTCGGGCCTGACGTTTCGCTAGGAGAGGACGTAGTTCACGTGCTGGTGGTGGTTCCAGAGGGTGCTGTTGGTTCA
GCATTATTGCAGCCAGCGAATAGCGCTACAATTCAAAATATCGCCTGGTATGGCACGCGTGGATCGATTGTGGAAGGAGAAGGCGTT
GATCTGAAAGATCCACGAACACTTTCTCGAGCCATTCTCACCGCGGATATCCTCCGCCGACTCGAAGAAACGCATGTCCTGCTGGTG
ACAAGCCCACCAATGACTGGGAAAACGTCACTAGCGACACTTGTGAGCCGATCGTTGGTGGATAGGCATATTATGGACAATAAGAAG
ATGACTCTGTTCAACTTCTCCGCCTTGGCCATACCCGACGATTCGACATTTGAGCAAGTTTTCAAGAAGCGTTGTACGATCGACTGG
AGCGAAGCAACTTTAAATCTTCCCACGCCAGATCGTATGGTGTATTTGGTGGTGGACGAAGTACAAGTGATCTACAAAGAAGGAACA
AATCCTCCGAGACGCAAATCCACTGTTTTTTGGGAGCTGGTCAAGTACGTTCTCAGCAACGGGAACTACAGCATCAGAATATTGATG
TTTGCAGCGTATGGATCTGGTGTGGAGTACACGCGACTCGCTACTCCAATACAGTTCGACGATAGGATTGTGTTGGGCATCGACCAG
CTGAAATTTAGTCATGCCGAAGTCTCTGAGTACGTCCAAAAGTGGTTTAAGGGTATCACGTGCTTTGAAGGATCATCGTCATCTGCG
ATGAAGGATTTCTGTGCCAATTTGGAAGAGGTGACGGGTAGGCACGTTGGTCTGTGTGCGACTACCATCTTTGAACTGAATAGAGTC
CATGCCTCACGTAATAGGAGTGCGAGTCGTCAACCATTGCCAGCCGAGTGGATCCGCATGCTGCAAAGTGGGTCACTGTATGAAGCC
AACGATAAAGCGCTGTTTAAAGCGCTGACATCAACACGCGCTGTGAAGGTGTTAAATACCCTTGACAAGGACGAGCTGGATCGGCTA
GAACGCATTGCTTATGGTGCCAACTCTGATTTTGATACTGATATAGTTGAGCAATGTCTTCGAAAGGGCATTCTTGTTCAAACTGAG
CGGCGTTTTGAGTTCTCGTCACCAGTGATGTGGCGATTCTTTGTCAAGATGCGCGTTGGTCACATCGTCCGAGCACTTCATGTCCCC
AAAACCCTTCCAGAGATGGTCGCTCGAGTGATGCGATCGATCGATTATGATAGCATCCGCCAGACGCTTGGCAGAAGTTTGTCGAGC
GATATTCCTCTGGAAAGAGCGTGGCAAATGGAATTTTACAAGGCTGCGTATCGCTGCACACCAAGCACTTTCGTGACGTCTGCGGAT
GTGGGTGCGTTGTTTGGATCCAGTGGGTTCGTCGATTTTACAATTCATGGAGGTGACATCTTTTGGGGCATCGAGTTGTTGCGAGAA
GCCAGCAACTTGGCTGAACACATCAAACGATTTTCCCCTGGTGGTCGCTATTCCTCGCTCCCGTTGACCGAGTTCTGTCTGGTTGAC
TTTAGACGTGTTGCTTCGATTGACGATGTGCCGATTGAACGTATAGCGGAAAATATGCGCGACTGCGACAAGCTCTTCGTCGTGTGT
TATGACGCACGGATGGCAGGTGTTATGGTGATTAATTCCGCAATGGATGTCGTCTACAGGACTCAATCTTAAAAAAGTTTTGTGATC
TTGCCAATTTGGTTGTTGCATTTGAAAACTTGATGCCATTGATGAAGAAAATAAGGTGAACTTTTTGAGGTCTACCGCATCGATTAA
TTTTTCATTCTAACAAATATAAAGTTAGTAATATACTTAGATGG
>PhCRN20_304 PLHAL3042491
CCGGACAACGAAGACCTGGACACGTTGCTTCAGAGCGAAATCGACACTTCATCGTATTTGCACATGCGTGCGTCGTGGAAGCTAAGC
AAGCCGAACCTTTTCGGGCCTGACGTTTCGCTAGGAGAGGACGTAGTTCACGTGCTGGTGGTGGTTCCAGAGGGTGCTGTTGGTTCA
GCATTATTGCAGCCAGCGAATAGCGCTACAATTCAAAATATCGCCTGGTATGGCACGCGTGGATCGATTGTGGAAGGAGAAGGCGTT
GATCTGAAAGATCCACGAACACTTTCTCGAGCCATTCTCACCGCGGATATCCTCCGCCGACTCGAAGAAACGCATGTCCTGCTGGTG
AAAAGCCCACCAATGACTGGGAAAACGTCACTAGCGACACTTGTGAGCCGATCGTTGGTGGATAGGCATATTATGGACAATAAGAAG
ATGACTCTGTTCAACTTCTCCGCCTTGGCCATACCCGACGATTCGACATTTGAGCAAGTTTTCAAGAAGCGTTGTACGATCGACTGG
AGCGAAGCAACTTTAAATCTTCCCACGCCAGATCGTATGGTGTATTTGGTGGTGGACGAAGTACAAGTGATCTACAAAGAAGGAACA
AATCCTCCGAGACGCAAATCCACTGTTTTTTGGGAGCTGGTCAAGTACGTTCTCAGCAACGGGAACTACAGCATCAGAATATTGATG
TTTGCAGCGTATGGATCTGGTGTGGAGTACACGCGACTCGCTACTCCAATACAGTTCGACGATAGGATTGTGTTGGGCATCGACCAG
CTGAAATTTAGTCATGCCGAAGTCTCTGAGTACGTCCAAAAGTGGTTTAAGGGTATCACGTGCTTTGAAGGATCATCGTCATCTGCG
ATGAAGGATTTCTGTGCCAATTTGGAAGAGGTGACGGGTAGGCACGTTGGTCTGTGTGCGACTACCATCTTTGAACTGAATAGAGTC
CATGCCTCACGTAATAGGAGTGCGAGTCGTCAACCATTGCCAGCCGAGTGGATCCGCATGCTGCAAAGTGGGTCACTGTATGAAGCC
AACGATAAAGCGCTGTTTAAAGCGCTGACATCAACACGCGCTGTGAAGGTGTTAAATACCCTTGACAAGGACGAGCTGGATCGGCTA
GAACGCATTGCTTATGGTGCCAACTCTGATTTTGATACTGATATAGTTGAGCAATGTCTTCGAAAGGGCATTCTTGTTCAAACTGAG
CGGCGTTTTGAGTTCTCGTCACCAGTGATGTGGCGATTCTTTGTCAAGATGCGCGTTGGTCACATCGTCCGAGCACTTCATGTCCCC
AAAACCCTTCCAGAGATGGTCGCTCGAGTGATGCGATCGATCGATTATGATAGCATCCGCCAGACGCTTGGCAGAAGTTTGTCGAGC
GATATTCCTCTGGAAAGAGCGTGGCAAATGGAATTTTACAAGGCTGCGTATCGCTGCACACCAAGCACTTTCGTGACGTCTGCGGAT
GTGGGTGCGTTGTTTGGATCCAGTGGGTTCGTCGATTTTACAATTCATGGAGGTGACATCTTTTGGGGCATCGAGTTGTTGCGAGAA
GCCAGCAACTTGGCTGAACACATCAAACGATTTTCCCCTGGTGGTCGCTATTCCTCGCTCCCGTTGACCGAGTTCTGTCTGGTTGAC
TTTAGACGTGTTGCTTCGATTGACGATGTGCCGATTGAACGTATAGCGGAAAATATGCGCGACTGCGACAAGCTCTTCGTCGTGTGT
TATGACGCACGGATGGCAGGTGTTATGGTGATTAATTCCGCAATGGATGTCGTCTACAGGACTCAATCTTAAAAAAGTTTTGTGATC
TTGCCAATTTGGTTGTTGCATTTGAAAACTTGATGCCATTGATGAAGAAAATAAGGTGAACTTTTTGAGGTCTACCGCATCGATTAA
TTTTTCATTCTAACAAATATAAAGTTAGTAATATACTTAGATGG
>PhCRN20_700 PLHAL7003053
CCGGACAACGAAGACCTGGACACGTTGCTTCAGAGCGAAATCGACACTTCATCGTATTTGCACATGCGTGCGTCGTGGAAGCTAAGC
AAGCCGAACCTTTTCGGGCCTGACGTTTCGCTAGGAGAGGACGTAGTTCACGTGCTGGTGGTGGTTCCAGAGGGTGCTGTTGGTTCA
GCATTATTGCAGCCAGCGAATAGCGCTACAATTCAAAATATCGCCTGGTATGGCACGCGTGGATCGATTGTGGAAGGAGAAGGCGTT
GATCTGAAAGATCCACGAACACTTTCTCGAGCCATTCTCACCGCGGATATCCTCCGCCGACTCGAAGAAACGCATGTCCTGCTGGTG

ACAAGCCCACCAATGACTGGGAAAACGTCACTAGCGACACTTGTGAGCCGATCGTTGGTGGATAGGCATATTATGGACAATAAGAAG
ATGACTCTGTTCAACTTCTCCGCCTTGGCCATACCCGACGATTCGACATTTGAGCAAGTTTTCAAGAAGCGTTGTACGATCGACTGG
AGCGAAGCAACTTTAAATCTTCCCACGCCAGATCGTATGGTGTATTTGGTGGTGGACGAAGTACAAGTGATCTACAAAGAAGGAACA
AATCCTCCGAGACGCAAATCCACTGTTTTTTGGGAGCTGGTCAAGTACGTTCTCAGCAACGGGAACTACAGCATCAGAATATTGATG
TTTGCAGCGTATGGATCTGGTGTGGAGTACACGCGACTCGCTACTCCAATACAGTTCGACGATAGGATTGTGTTGGGCATCGACCAG
CTGAAATTTAGTCATGCCGAAGTCTCTGAGTACGTCCAAAAGTGGTTTAAGGGTATCACGTGCTTTGAAGGATCATCGTCATCTGCG
ATGAAGGATTTCTGTGCCAATTTGGAAGAGGTGACGGGTAGGCACGTTGGTCTGTGTGCGACTACCATCTTTGAACTGAATAGAGTC
CATGCCTCACGTAATAGGAGTGCGAGTCGTCAACCATTGCCAGCCGAGTGGATCCGCATGCTGCAAAGTGGGTCACTGTATGAAGCC
AACGATAAAGCGCTGTTTAAAGCGCTGACATCAACACGCGCTGTGAAGGTGTTAAATACCCTTGACAAGGACGAGCTGGATCGGCTA
GAACGCATTGCTTATGGTGCCAACTCTGATTTTGATACTGATATAGTTGAGCAATGTCTTCGAAAGGGCATTCTTGTTCAAACTGAG
CGGCGTTTTGAGTTCTCGTCACCAGTGATGTGGCGATTCTTTGTCAAGATGCGCGTTGGTCACATCGTCCGAGCACTTCATGTCCCC
AAAACCCTTCCAGAGATGGTCGCTCGAGTGATGCGATCGATCGATTATGATAGCATCCGCCAGACGCTTGGCAGAAGTTTGTCGAGC
GATATTCCTCTGGAAAGAGCGTGGCAAATGGAATTTTACAAGGCTGCGTATCGCTGCACACCAAGCACTTTCGTGACGTCTGCGGAT
GTGGGTGCGTTGTTTGGATCCAGTGGGTTCGTCGATTTTACAATTCATGGAGGTGACATCTTTTGGGGCATCGAGTTGTTGCGAGAA
GCCAGCAACTTGGCTGAACACATCAAACGATTTTCCCCTGGTGGTCGCTATTCCTCGCTCCCGTTGACCGAGTTCTGTCTGGTTGAC
TTTAGACGTGTTGCTTCGATTGACGATGTGCCGATTGAACGTATAGCGGAAAATATGCGCGACTGCGACAAGCTCTTCGTCGTGTGT
TATGACGCACGGATGGCAGGTGTTATGGTGATTAATTCCGCAATGGATGTCGTCTACAGGACTCAATCTTAAAAAAGTTTTGTGATC
TTGCCAATTTGGTTGTTGCATTTGAAAACTTGATGCCATTGATGAAGAAAATAAGGTGAACTTTTTGAGGTCTACCGCATCGATTAA
TTTTTCATTCTAACAAATATAAAGTTAGTAATATACTTAGATGG
>PhCRN20_730 PLHAL7301301
CCGGACAACGAAGACCTGGACACGTTGCTTCAGAGCGAAATCGACACTTCATCGTATTTGCACATGCGTGCGTCGTGGAAGCTAAGC
AAGCCGAACCTTTTCGGGCCTGACGTTTCGCTAGGAGAGGACGTAGTTCACGTGCTGGTGGTGGTTCCAGAGGGTGCTGTTGGTTCA
GCATTATTGCAGCCAGCGAATAGCGCTACAATTCAAAATATCGCCTGGTATGGCACGCGTGGATCGATTGTGGAAGGAGAAGGCGTT
GATCTGAAAGATCCACGAACACTTTCTCGAGCCATTCTCACCGCGGATATCCTCCGCCGACTCGAAGAAACGCATGTCCTGCTGGTG
ACAAGCCCACCAATGACTGGGAAAACGTCACTAGCGACACTTGTGAGCCGATCGTTGGTGGATAGGCATATTATGGACAATAAGAAG
ATGACTCTGTTCAACTTCTCCGCCTTGGCCATACCCGACGATTCGACATTTGAGCAAGTTTTCAAGAAGCGTTGTACGATCGACTGG
AGCGAAGCAACTTTAAATCTTCCCACGCCAGATCGTATGGTGTATTTGGTGGTGGACGAAGTACAAGTGATCTACAAAGAAGGAACA
AATCCTCCGAGACGCAAATCCACTGTTTTTTGGGAGCTGGTCAAGTACGTTCTCAGCAACGGGAACTACAGCATCAGAATATTGATG
TTTGCAGCGTATGGATCTGGTGTGGAGTACACGCGACTCGCTACTCCAATACAGTTCGACGATAGGATTGTGTTGGGCATCGACCAG
CTGAAATTTAGTCATGCCGAAGTCTCTGAGTACGTCCAAAAGTGGTTTAAGGGTATCACGTGCTTTGAAGGATCATCGTCATCTGCG
ATGAAGGATTTCTGTGCCAATTTGGAAGAGGTGACGGGTAGGCACGTTGGTCTGTGTGCGACTACCATCTTTGAACTGAATAGAGTC
CATGCCTCACGTAATAGGAGTGCGAGTCGTCAACCATTGCCAGCCGAGTGGATCCGCATGCTGCAAAGTGGGTCACTGTATGAAGCC
AACGATAAAGCGCTGTTTAAAGCGCTGACATCAACACGCGCTGTGAAGGTGTTAAATACCCTTGACAAGGACGAGCTGGATCGGCTA
GAACGCATTGCTTATGGTGCCAACTCTGATTTTGATACTGATATAGTTGAGCAATGTCTTCGAAAGGGCATTCTTGTTCAAACTGAG
CGGCGTTTTGAGTTCTCGTCACCAGTGATGTGGCGATTCTTTGTCAAGATGCGCGTTGGTCACATCGTCCGAGCACTTCATGTCCCC
AAAACCCTTCCAGAGATGGTCGCTCGAGTGATGCGATCGATCGATTATGATAGCATCCGCCAGACGCTTGGCAGAAGTTTGTCGAGC
GATATTCCTCTGGAAAGAGCGTGGCAAATGGAATTTTACAAGGCTGCGTATCGCTGCACACCAAGCACTTTCGTGACGTCTGCGGAT
GTGGGTGCGTTGTTTGGATCCAGTGGGTTCGTCGATTTTACAATTCATGGAGGTGACATCTTTTGGGGCATCGAGTTGTTGCGAGAA
GCCAGCAACTTGGCTGAACACATCAAACGATTTTCCCCTGGTGGTCGCTATTCCTCGCTCCCGTTGACCGAGTTCTGTCTGGTTGAC
TTTAGACGTGTTGCTTCGATTGACGATGTGCCGATTGAACGTATAGCGGAAAATATGCGCGACTGCGACAAGCTCTTCGTCGTGTGT
TATGACGCACGGATGGCAGGTGTTATGGTGATTAATTCCGCAATGGATGTCGTCTACAGGACTCAATCTTAAAAAAGTTTTGTGATC
TTGCCAATTTGGTTGTTGCATTTGAAAACTTGATGCCATTGATGAAGAAAATAAGGTGAACTTTTTGAGGTCTACCGCATCGATTAA
TTTTTCATTCTAACAAATATAAAGTTAGTAATATACTTAGATGG

>PhCRN30.1_100 PLHAL100A10010
GCGAAGAAAGACAAGGGCCGCGGCGCGTGGGTGACGGAAGAAGAAGCGATGAGCGGTGTGAATGACACAACTGATCTGAAGTTACTA
AAATCTGCGCGAGCGACAATCGGTGATGTTGGGCTTTCAGAAGATAAAGTGCGGATTCAAGTGCCCAAAAAAGTAGTGAAGGATCTG
AAGGGCCCTGTTCACGTGCTAGTGGTGGTTCCGGGGCGTGCAGTGGAGGATGGCGCTTGGAGCGCAAAGGACTTGTCTACTGTGGAC
CCTCAAGCTCAATCGAAAATGTGGAAGTCAGTTGTCAGAGTTTCCTCAGGAGATGTATGCTCAGGGACAGCGGTGGTTGTGGATCTA
ACGTCGACGCACTTGTATCTGCTGACAAACTTGCATTTTTGGGTCGACGACACATTCACTGATCACTTGAGTGCTGACTTCACGACG
GAAATCAAACGGTATCTGAGACGCCACCCCCGGATGAAAACAAGTGGAAGGAAAAAGAATGTCACCAATAATAAGGATGCTGATGTT
GCAAAGCGACCACGTCGTAAATCTCTACGGACGGCAGCAATGAAATCATCAGCTAAATTAGACAAGCCTCAAGTTGTGGTGGAGCAA
CTCCTTCCCGATAGGCCGAAGCCGGAAGAAGTCAACCGATTCAGTCTCGACAGTGACATTTGCTGGTGTAGTTCGGCTGCGTTCGAC
TTCGCAATCTTCGAAGTTGTTTTGCCACGAAATAACAAGCTCGACCGATGCGACATGTCCTTCAATGTGTCTTACGGAATGAGTGTT
GACGTGTTCGGTTTTCCTGGCGCTTTTGAAGATCAAGCTTTTGATCATGCTTATGCCATCATTCCAGCTAGAATCACCGGTTGGAGC
GGAAACCGAATGGTTCTCTCCTCTTTGTCAGCTCCTGGTGTATCTGGAAGCGCGTTAGTGTGCACCAAAAATGAAGTAGCAGCGGGA
TATCTTGGTGCTGGACTTTATGGCTCAGCAAAAAACGAGCGGTATCAATCCTATTTCTACACATTTCACGGAGTAATCCCCGAATTA
CCGTCTTCGTTGCCGCGATACCGAAGTCCAAACAAAGGATGAGCTATAGTCTGTATACCGTGTCATGTAATGAATGCATCTGCGAGT
ATCTTTCAATTTCAGTGATGAGCTCATCGAATGTTTTGCATGCGATTGTGGTTGAAATGTAGTGAATTGCCATCTGAAGAGTCGGCA
ACTAATCAATGCTTTTTC
>PhCRN30.1_334 PLHAL3340246
GCGAAGAAAGACAAGGGCCGCGGCGCGTGGGTGACGGAAGAAGAAGCGATGAGCGGTGTGAATGACACAACTGATCTGAAGTTACTA
AAATCTGCGCGAGCGACAATCGGTGATGTTGGGCTTTCAGAAGATAAAGTGCGGATTCAAGTGCCCAAAAAAGTAGTGAAGGATCTG
AAGGGCCCTGTTCACGTGCTGGTGGTGGTTCCGGGGCGTGCAGTGGAGGATGGCGCTTGGAGCGCAAAGGACTTGTCTACTGTGGAC
CCTCAAGCTCAATCGAAAATGTGGAAGTCAGTTGTCAGAGTTTCCTCAGGAGATGTATGCTCAGGGACAGCGGTGGTTGTGGATCTA
ACGTCGACGCACTTGTATCTGCTGACAAACTTGCATTTTTGGGTCGACGACACATTCACTGATCACTTGAGTGCTGACTTCACGACG
GAAATCAAACGGTATCTGAGACGCCACCCCCGGATGAAAACAAGTGGAAGGAAAAAGAATGTCACCAATAATAAGGATGCTGATGTT

GCAAAGCGACCACGTCGTAAATCTCTACGGACGGCAGCAATGAAATCATCAGCTAAATTAGACAAGCCTCAAGTTGTGGTGGAGCAA
CTCCTTCCCGATAGGCCGAAGCCGGAAGAAGTCAACCGATTCAGTCTCGACAGTGACATTTGCTGGTGTAGTTCGGCTGCGTTCGAC
TTCGCAATCTTCGAAGTTGTTTTGCCACGAAATAACAAGCTCGACCGATGCGACATGTCCTTCAATGTGTCTTACGGAATGAGTGTT
GACGTGTTCGGTTTTCCTGGCGCTTTTGAAGATCAAGCTTTTGATCATGCTTATGCCATCATTCCAGCTAGAATCACCGGTTGGAGC
GGAAACCGAATGGTTCTCTCCTCTTTGTCAGCTCCTGGTGTATCTGGAAGCGCGTTAGTGTGCACCAAAAATGAAGTAGCAGCGGGA
TATCTTGGTGCTGGACTTTATGGCTCAGCAAAAAACGAGCGGTATCAATCCTATTTCTACACATTTCACGGAGTAATCCCCGAATTA
CCGTCTTCGTTGCCGCGATACCGAAGTCCAAACAAAGGATGAGCTATAGTCTGTATACCGTGTCATGTAATGAATGCATCTGCGAGT
ATCTTTCAATTTCAGTGATGAGCTCATCGAATGTTTTGCATGCGATTGTGGTTGAAATGTAGTGAATTGCCATCTGAAGAGTCGGCA
ACTAATCAATGCTTTTTC
>PhCRN30.1_703 PLHAL7030055
GCGAAGAAAGACAAGGGCCGCGGCGCGTGGGTGACGGAAGAAGAAGCGATGAGCGGTGTGAATGACACAACTGATCTGAAGTTACTA
AAATCTGCGCGAGCGACAATCGGTGATGTTGGGCTTTCAGAAGATAAAGTGCGGATTCAAGTGCCCAAAAAAGTAGTGAAGGATCTG
AAGGGCCCTGTTCACGTGCTGGTGGTGGTTCCGGGGCGTGCAGTGGAGGATGGCGCTTGGAGCGCAAAGGACTTGTCTACTGTGGAC
CCTCAAGCTCAATCGAAAATGTGGAAGTCAGTTGTCAGAGTTTCCTCAGGAGATGTATGCTCAGGGACAGCGGTGGTTGTGGATCTA
ACGTCGACGCACTTGTATCTGCTGACAAACTTGCATTTTTGGGTCGACGACACATTCACTGATCACTTGAGTGCTGACTTCACGACG
GAAATCAAACGGTATCTGAGACGCCACCCCCGGATGAAAACAAGTGGAAGGAAAAAGAATGTCACCAATAATAAGGATGCTGATGTT
GCAAAGCGACCACGTCGTAAATCTCTACGGACGGCAGCAATGAAATCATCAGCTAAATTAGACAAGCCTCAAGTTGTGGTGGAGCAA
CTCCTTCCCGATAGGCCGAAGCCGGAAGAAGTCAACCGATTCAGTCTCGACAGTGACATTTGCTGGTGTAGTTCGGCTGCGTTCGAC
TTCGCAATCTTCGAAGTTGTTTTGCCACGAAATAACAAGCTCGACCGATGCGACATGTCCTTCAATGTGTCTTACGGAATGAGTGTT
GACGTGTTCGGTTTTCCTGGCGCTTTTGAAGATCAAGCTTTTGATCATGCTTATGCCATCATTCCAGCTAGAATCACCGGTTGGAGC
GGAAACCGAATGGTTCTCTCCTCTTTGTCAGCTCCTGGTGTATCTGGAAGCGCGTTAGTGTGCACCAAAAATGAAGTAGCAGCGGGA
TATCTTGGTGCTGGACTTTATGGCTCAGCAAAAAACGAGCGGTATCAATCCTATTTCTACACATTTCACGGAGTAATCCCCGAATTA
CCGTCTTCGTTGCCGCGATACCGAAGTCCAAACAAAGGATGAGCTATAGTCTGTATACCGTGTCATGTAATGAATGCATCTGCGAGT
ATCTTTCAATTTCAGTGATGAGCTCATCGAATGTTTTGCATGCGATTGTGGTTGAAATGTAGTGAATTGCCATCTGAAGAGTCGGCA
ACTAATCAATGCTTTTTC
>PhCRN30.1_710 PLHAL7100086
GCGAAGAAAGACAAGGGCCGCGGCGCGTGGGTGACGGAAGAAGAAGCGATGAGCGGTGTGAATGACACAACTGATCTGAAGTTACTA
AAATCTGCGCGAGCGACAATCGGTGATGTTGGGCTTTCAGAAGATAAAGTGCGGATTCAAGTGCCCAAAAAAGTAGTGAAGGATCTG
AAGGGCCCTGTTCACGTGCTGGTGGTGGTTCCGGGGCGTGCAGTGGAGGATGGCGCTTGGAGCGCAAAGGACTTGTCTACTGTGGAC
CCTCAAGCTCAATCGAAAATGTGGAAGTCAGTTGTCAGAGTTTCCTCAGGAGATGTATGCTCAGGGACAGCGGTGGTTGTGGATCTA
ACGTCGACGCACTTGTATCTGCTGACAAACTTGCATTTTTGGGTCGACGACACATTCACTGATCACTTGAGTGCTGACTTCACGACG
GAAATCAAACGGTATCTGAGACGCCACCCCCGGATGAAAACAAGTGGAAGGAAAAAGAATGTCACCAATAATAAGGATGCTGATGTT
GCAAAGCGACCACGTCGTAAATCTCTACGGACGGCAGCAATGAAATCATCAGCTAAATTAGACAAGCCTCAAGTTGTGGTGGAGCAA
CTCCTTCCCGATAGGCCGAAGCCGGAAGAAGTCAACCGATTCAGTCTCGACAGTGACATTTGCTGGTGTAGTTCGGCTGCGTTCGAC
TTCGCAATCTTCGAAGTTGTTTTGCCACGAAATAACAAGCTCGACCGATGCGACATGTCCTTCAATGTGTCTTACGGAATGAGTGTT
GACGTGTTCGGTTTTCCTGGCGCTTTTGAAGATCAAGCTTTTGATCATGCTTATGCCATCATTCCAGCTAGAATCACCGGTTGGAGC
GGAAACCGAATGGTTCTCTCCTCTTTGTCAGCTCCTGGTGTATCTGGAAGCGCGTTAGTGTGCACCAAAAATGAAGTAGCAGCGGGA
TATCTTGGTGCTGGACTTTATGGCTCAGCAAAAAACGAGCGGTATCAATCCTATTTCTACACATTTCACGGAGTAATCCCCGAATTA
CCGTCTTCGTTGCCGCGATACCGAAGTCCAAACAAAGGATGAGCTATAGTCTGTATACCGTGTCATGTAATGAATGCATCTGCGAGT
ATCTTTCAATTTCAGTGATGAGCTCATCGAATGTTTTGCATGCGATTGTGGTTGAAATGTAGTGAATTGCCATCTGAAGAGTCGGCA
ACTAATCAATGCTTTTTC
>PhCRN30.1_304 PLHAL3040004
GCGAAGAAAGACAAGGGCCGCGGCGCGTGGGTGACGGAAGAAGAAGCGATGAGCGGTGTGAATGACACAACTGATCTGAAGTTACTA
AAATCTGCGCGAGCGACAATCGGTGATGTTGGGCTTTCAGAAGATAAAGTGCGGATTCAAGTGCCCAAAAAAGTAGTGAAGGATCTG
AAGGGCCCTGTTCACGTGCTAGTGGTGGTTCCGGGGCGTGCAGTGGAGGATGGCGCTTGGAGCGCAAAGGACTTGTCTACTGTGGAC
CCTCAAGCTCAATCGAAAATGTGGAAGTCAGTTGTCAGAGTTTCCTCAGGAGATGTATGCTCAGGGACAGCGGTGGTTGTGGATCTA
ACGTCGACGCACTTGTATCTGCTGACAAACTTGCATTTTTGGGTCGACGACACATTCACTGATCACTTGAGTGCTGACTTCACGACG
GAAATCAAACGGTATCTGAGACGCCACCCCCGGATGAAAACAAGTGGAAGGAAAAAGAATGTCACCAATAATAAGGATGCTGATGTT
GCAAAGCGACCACGTCGTAAATCTCTACGGACGGCAGCAATGAAATCATCAGCTAAATTAGACAAGCCTCAAGTTGTGGTGGAGCAA
CTCCTTCCCGATAGGCCGAAGCCGGAAGAAGTCAACCGATTCAGTCTCGACAGTGACATTTGCTGGTGTAGTTCGGCTGCGTTCGAC
TTCGCAATCTTCGAAGTTGTTTTGCCACGAAATAACAAGCTCGACCGATGCGACATGTCCTTCAATGTGTCTTACGGAATGAGTGTT
GACGTGTTCGGTTTTCCTGGCGCTTTTGAAGATCAAGCTTTTGATCATGCTTATGCCATCATTCCAGCTAGAATCACCGGTTGGAGC
GGAAACCGAATGGTTCTCTCCTCTTTGTCAGCTCCTGGTGTATCTGGAAGCGCGTTAGTGTGCACCAAAAATGAAGTAGCAGCGGGA
TATCTTGGTGCTGGACTTTATGGCTCAGCAAAAAACGAGCGGTATCAATCCTATTTCTACACATTTCACGGAGTAATCCCCGAATTA
CCGTCTTCGTTGCCGCGATACCGAAGTCCAAACAAAGGATGAGCTATAGTCTGTATACCGTGTCATGTAATGAATGCATCTGCGAGT
ATCTTTCAATTTCAGTGATGAGCTCATCGAATGTTTTGCATGCGATTGTGGTTGAAATGTAGTGAATTGCCATCTGAAGAGTCGGCA
ACTAATCAATGCTTTTTC
>PhCRN30.1_700 PLHAL7000187
GCGAAGAAAGACAAGGGCCGCGGCGCGTGGGTGACGGAAGAAGAAGCGATGAGCGGTGTGAATGACACAACTGATCTGAAGTTACTA
AAATCTGCGCGAGCGACAATCGGTGATGTTGGGCTTTCAGAAGATAAAGTGCGGATTCAAGTGCCCAAAAAAGTAGTGAAGGATCTG
AAGGGCCCTGTTCACGTGCTGGTGGTGGTTCCGGGGCGTGCAGTGGAGGATGGCGCTTGGAGCGCAAAGGACTTGTCTACTGTGGAC
CCTCAAGCTCAATCGAAAATGTGGAAGTCAGTTGTCAGAGTTTCCTCAGGAGATGTATGCTCAGGGACAGCGGTGGTTGTGGATCTA
ACGTCGACGCACTTGTATCTGCTGACAAACTTGCATTTTTGGGTCGACGACACATTCACTGATCACTTGAGTGCTGACTTCACGACG
GAAATCAAACGGTATCTGAGACGCCACCCCCGGATGAAAACAAGTGGAAGGAAAAAGAATGTCACCAATAATAAGGATGCTGATGTT
GCAAAGCGACCACGTCGTAAATCTCTACGGACGGCAGCAATGAAATCATCAGCTAAATTAGACAAGCCTCAAGTTGTGGTGGAGCAA
CTCCTTCCCGATAGGCCGAAGCCGGAAGAAGTCAACCGATTCAGTCTCGACAGTGACATTTGCTGGTGTAGTTCGGCTGCGTTCGAC
TTCGCAATCTTCGAAGTTGTTTTGCCACGAAATAACAAGCTCGACCGATGCGACATGTCCTTCAATGTGTCTTACGGAATGAGTGTT

GACGTGTTCGGTTTTCCTGGCGCTTTTGAAGATCAAGCTTTTGATCATGCTTATGCCATCATTCCAGCTAGAATCACCGGTTGGAGC
GGAAACCGAATGGTTCTCTCCTCTTTGTCAGCTCCTGGTGTATCTGGAAGCGCGTTAGTGTGCACCAAAAATGAAGTAGCAGCGGGA
TATCTTGGTGCTGGACTTTATGGCTCAGCAAAAAACGAGCGGTATCAATCCTATTTCTACACATTTCACGGAGTAATCCCCGAATTA
CCGTCTTCGTTGCCGCGATACCGAAGTCCAAACAAAGGATGAGCTATAGTCTGTATACCGTGTCATGTAATGAATGCATCTGCGAGT
ATCTTTCAATTTCAGTGATGAGCTCATCGAATGTTTTGCATGCGATTGTGGTTGAAATGTAGTGAATTGCCATCTGAAGAGTCGGCA
ACTAATCAATGCTTTTTC
>PhCRN30.1_730 PLHAL7300008
GCGAAGAAAGACAAGGGCCGCGGCGCGTGGGTGACGGAAGAAGAAGCGATGAGCGGTGTGAATGACACAACTGATCTGAAGTTACTA
AAATCTGCGCGAGCGACAATCGGTGATGTTGGGCTTTCAGAAGATAAAGTGCGGATTCAAGTGCCCAAAAAAGTAGTGAAGGATCTG
AAGGGCCCTGTTCACGTGCTGGTGGTGGTTCCGGGGCGTGCAGTGGAGGATGGCGCTTGGAGCGCAAAGGACTTGTCTACTGTGGAC
CCTCAAGCTCAATCGAAAATGTGGAAGTCAGTTGTCAGAGTTTCCTCAGGAGATGTATGCTCAGGGACAGCGGTGGTTGTGGATCTA
ACGTCGACGCACTTGTATCTGCTGACAAACTTGCATTTTTGGGTCGACGACACATTCACTGATCACTTGAGTGCTGACTTCACGACG
GAAATCAAACGGTATCTGAGACGCCACCCCCGGATGAAAACAAGTGGAAGGAAAAAGAATGTCACCAATAATAAGGATGCTGATGTT
GCAAAGCGACCACGTCGTAAATCTCTACGGACGGCAGCAATGAAATCATCAGCTAAATTAGACAAGCCTCAAGTTGTGGTGGAGCAA
CTCCTTCCCGATAGGCCGAAGCCGGAAGAAGTCAACCGATTCAGTCTCGACAGTGACATTTGCTGGTGTAGTTCGGCTGCGTTCGAC
TTCGCAATCTTCGAAGTTGTTTTGCCACGAAATAACAAGCTCGACCGATGCGACATGTCCTTCAATGTGTCTTACGGAATGAGTGTT
GACGTGTTCGGTTTTCCTGGCGCTTTTGAAGATCAAGCTTTTGATCATGCTTATGCCATCATTCCAGCTAGAATCACCGGTTGGAGC
GGAAACCGAATGGTTCTCTCCTCTTTGTCAGCTCCTGGTGTATCTGGAAGCGCGTTAGTGTGCACCAAAAATGAAGTAGCAGCGGGA
TATCTTGGTGCTGGACTTTATGGCTCAGCAAAAAACGAGCGGTATCAATCCTATTTCTACACATTTCACGGAGTAATCCCCGAATTA
CCGTCTTCGTTGCCGCGATACCGAAGTCCAAACAAAGGATGAGCTATAGTCTGTATACCGTGTCATGTAATGAATGCATCTGCGAGT
ATCTTTCAATTTCAGTGATGAGCTCATCGAATGTTTTGCATGCGATTGTGGTTGAAATGTAGTGAATTGCCATCTGAAGAGTCGGCA
ACTAATCAATGCTTTTTC

>PhCRN30.2_100 PLHAL100A11176
CAATTAAGAAGGAGCAGGAGTTTGACTTCGCGGCCAACAAATTAGAGCTATATCTCGCGAAGAAAGACAAGGGCCGTGGCGCGTGGG
TGACGGAAGAAGAAGCGAGGGGTTTGAATGGCACAAGTGATTTGGAGATATTGGACGTTGCGAGAGCACCGCTTAAATTTGTTGGCT
TATCGGAGGAAGACGTCCGGTTTCAAGTCACGAAGGAAGACGTGAAGGCAAAAACGACTCCTGTCCACGTGCTAGTGGTGGTTCCGG
GGAGTGCAGTGGATGATGGCGCTTGGAGCGCTAAGGAATTTTCTACCGTGGACCCTCAAGTTCAACTAAAAATGTGGAGGTCAGTTG
TCAGAGTTTCCTCAGAAGATGTATGCTCAGGAACAGGAGTGGTTGTGGATTTAACGTCGACGCACTTGTATCTGCTGACGAACTTGC
ACCTTTGGGTCGACGACACATTCACTGATCACTTGAGTTCTGACTTCACGAAGGAAATCAAACGGTATCGGAGACGCCACCCCCGGA
TAAAAACAAGTGGAAGGAAAAGGAACGCCACCAATGTAAAGGACGCCGATGTTGCAAAGCAACCACGTCGTAAATCTACACGGACGG
CAGCGATGAAATCATCAGCTGGATTAGACAAGCCTCAAGTTATGGTGGAGCAACTCCTTTTCGATAGGACGAAGCCCGAAGAAGTCC
ACCGATTCAGTCTCGACAGTGACATTTGCTGGTGTAGTTCGGCTGCGTTCGACTTCGCAATCTTCAAAGTTAGTTTGCCACGAGATA
A
>PhCRN30.2_334 PLHAL3341207
CAATTAAGAAGGAGCAGGAGTTTGACTTCGCGGCCAACAAATTAGAGCTATATCTCGCGAAGAAAGACAAGGGCCGTGGCGCGTGGG
TGACGGAAGAAGAAGCGAGGGGTTTGAATGGCACAAGTGATTTGGAGATATTGGACGTTGCGAGAGCACCGCTTAAATTTGTTGGCT
TATCGGAGGAAGACGTCCGGTTTCAAGTCACGAAGGAAGACGTGAAGGCAAAAACGACTCCTGTCCACGTGCTAGTGGTGGTTCCGG
GGAGTGCAGTGGATGATGGCGCTTGGAGCGCTAAGGAATTTTCTACCGTGGACCCTCAAGTTCAACTAAAAATGTGGAGGTCAGTTG
TCAGAGTTTCCTCAGAAGATGTATGCTCAGGAACAGGAGTGGTTGTGGATTTAACGTCGACGCACTTGTATCTGCTGACGAACTTGC
ACCTTTGGGTCGACGACACATTCACTGATCACTTGAGTTCTGACTTCACGAAGGAAATCAAACGGTATCGGAGACGCCACCCCCGGA
TAAAAACAAGTGGAAGGAAAAGGAACGCCACCAATGTAAAGGACGCCGATGTTGCAAAGCAACCACGACGTAAATCTACACGGACGG
CAGCGATGAAATCATCAGCTGGATTAGACAAGCCTCAAGTTATGGTGGAGCAACTCCTTTTCGATAGGACGAAGCCCGAAGAAGTCC
ACCGATTCAGTCTCGACAGTGACATTTGCTGGTGTAGTTCGGCTGCGTTCGACTTCGCAATCTTCAAAGTTAGTTTGCCACGAGATA
A
>PhCRN30.2_703 PLHAL7031162
CAATTAAGAAGGAGCAGGAGTTTGACTTCGCGGCCAACAAATTAGAGCTATATCTCGCGAAGAAAGACAAGGGCCGTGGCGCGTGGG
TGACGGAAGAAGAAGCGAGGGGTTTGAATGGCACAAGTGATTTGGAGATATTGGACGTTGCGAGAGCACCGCTTAAATTTGTTGGCT
TATCGGAGGAAGACGTCCGGTTTCAAGTCACGAAGGAAGACGTGAAGGCAAAAACGACTCCTGTCCACGTGCTAGTGGTGGTTCCGG
GGAGTGCAGTGGATGATGGCGCTTGGAGCGCTAAGGAATTTTCTACCGTGGACCCTCAAGTTCAACTAAAAATGTGGAGGTCAGTTG
TCAGAGTTTCCTCAGAAGATGTATGCTCAGGAACAGGAGTGGTTGTGGATTTAACGTCGACGCACTTGTATCTGCTGACGAACTTGC
ACCTTTGGGTCGACGACACATTCACTGATCACTTGAGTTCTGACTTCACGAAGGAAATCAAACGGTATCGGAGACGCCACCCCCGGA
TAAAAACAAGTGGAAGGAAAAGGAACGCCACCAATGTAAAGGACGCCGATGTTGCAAAGCAACCACGACGTAAATCTACACGGACGG
CAGCGATGAAATCATCAGCTGGATTAGACAAGCCTCAAGTTATGGTGGAGCAACTCCTTTTCGATAGGACGAAGCCCGAAGAAGTCC
ACCGATTCAGTCTCGACAGTGACATTTGCTGGTGTAGTTCGGCTGCGTTCGACTTCGCAATCTTCAAAGTTGCTTTGCCACGAGATA
ACAAGCTCGACCGATGCACAATATCCCTCAAGGTGACAGACACAATGAG
>PhCRN30.2_710 PLHAL7101032
CAATTAAGAAGGAGCAGGAGTTTGACTTCGCGGCCAACAAATTAGAGCTATATCTCGCGAAGAAAGACAAGGGCCGTGGCGCGTGGG
TGACGGAAGAAGAAGCGAGGGGTTTGAATGGCACAAGTGATTTGGAGATATTGGACGTTGCGAGAGCACCGCTTAAATTTGTTGGCT
TATCGGAGGAAGACGTCCGGTTTCAAGTCACGAAGGAAGACGTGAAGGCAAAAACGACTCCTGTCCACGTGCTAGTGGTGGTTCCGG
GGAGTGCAGTGGATGATGGCGCTTGGAGCGCTAAGGAATTTTCTACCGTGGACCCTCAAGTTCAACTAAAAATGTGGAGGTCAGTTG
TCAGAGTTTCCTCAGAAGATGTATGCTCAGGAACAGGAGTGGTTGTGGATTTAACGTCGACGCACTTGTATCTGCTGACGAACTTGC
ACCTTTGGGTCGACGACACATTCACTGATCACTTGAGTTCTGACTTCACGAAGGAAATCAAACGGTATCGGAGACGCCACCCCCGGA
TAAAAACAAGTGGAAGGAAAAGGAACGCCACCAATGTAAAGGACGCCGATGTTGCAAAGCAACCACGACGTAAATCTACACGGACGG
CAGCGATGAAATCATCAGCTGGATTAGACAAGCCTCAAGTTATGGTGGAGCAACTCCTTTTCGATAGGACGAAGCCCGAAGAAGTCC
ACCGATTCAGTCTCGACAGTGACATTTGCTGGTGTAGTTCGGCTGCGTTCGACTTCGCAATCTTCAAAGTTGCTTTGCCACGAGATA
ACAAGCTCGACCGATGCACAATATCCCTCAAGGTGACAGACACAATGAGTGTTGACGTGTTCGGATTCCCCGGCGCTCTTGAAGATC

AAGCTTTTGATCATGCTTATGCCATCATTCCAGCTAGAATCACAGGTTGGAGCGGAAACCAAATGGTTCTCTCCTCTTTGTCAGCTC
CTGGTCTATCTGGAAGCGCGATTGTGTGCACCAAAAGTGGAGTTCCAGTGGGATATCTTGGTGGTGGACTTGATGGCTCAGCAAAGA
ACGAGCTGTATCAATCCTATGGCTACACATTTCACGGAGTTATCCCCGAATTACCGTCTTCGTTGCCGCCAGATACCTAAGTTGAAA
CAAAGGATGAGCTATAATCTGTATACAGTGCCATGTAGTGAAAGCATCTATGAGTATCCTTCAGTATCTGTGATGAGCTCATTGGTC
GCGATTGTTTTGCATGTAATTGTGGTTGAAAGGTAGTGAATTTGCCATCTGAATCAGTCGGCAACTAATTAATGCTTTTTCTATTAT
ATAAAGTATAGATTGTACTA
>PhCRN30.2_304 PLHAL3041131
CAATTAAGAAGGAGCAGGAGTTTGACTTCGCGGCCAACAAATTAGAGCTATATCTCGCGAAGAAAGACAAGGGCCGTGGCGCGTGGG
TGACGGAAGAAGAAGCGAGGGGTTTGAATGGCACAAGTGATTTGGAGATATTGGACGTTGCGAGAGCACCGCTTAAATTTGTTGGCT
TATCGGAGGAAGACGTCCGGTTTCAAGTCACGAAGGAAGACGTGAAGGCAAAAACGACTCCTGTCCACGTGCTAGTGGTGGTTCCGG
GGAGTGCAGTGGATGATGGCGCTTGGAGCGCTAAGGAATTTTCTACCGTGGACCCTCAAGTTCAACTAAAAATGTGGAGGTCAGTTG
TCAGAGTTTCCTCAGAAGATGTATGCTCAGGAACAGGAGTGGTTGTGGATTTAACGTCGACGCACTTGTATCTGCTGACGAACTTGC
ACCTTTGGGTCGACGACACATTCACTGATCACTTGAGTTCTGACTTCACGAAGGAAATCAAACGGTATCGGAGACGCCACCCCCGGA
TAAAAACAAGTGGAAGGAAAAGGAACGCCACCAATGTAAAGGACGCCGATGTTGCAAAGCAACCACGTCGTAAATCTACACGGACGG
CAGCGATGAAATCATCAGCTGGATTAGACAAGCCTCAAGTTATGGTGGAGCAACTCCTTTTCGATAGGACGAAGCCCGAAGAAGTCC
ACCGATTCAGTCTCGACAGTGACATTTGCTGGTGTAGTTCGGCTGCGTTCGACTTCGCAATCTTCAAAGTTGCTTTGCCACGAGATA
ACAAGCTCGACCGATGCACAATATCCCTCAAGGTGACAGACACAATGAGTGTTGACGTGTTCGGATTCCCCGGCGCTCTTGAAGATC
AAGCTTTTGATCATGCTTATGCCATCATTCCAGCTAGAATCACAGGTTGGAGCGGAAACCAAATGGTTCTCTCCTCTTTGTCAGCTC
CTGGTCTATCTGGAAGCGCGATTGTGTGCACCAAAAGTGGAGTTCCAGTGGGATATCTTGGTGGTGGACTTGATGGCTCAGCAAAGA
ACGAGCTGTATCAATCCTATGGCTACACATTTCACGGAGTTATCCCCGAATTACCGTCTTCGTTGCCGCCAGATACCTAAGTTGAAA
CAAAGGATGAGCTATAATCTGTATACAGTGCCATGTAGTGAAAGCATCTATGAGTATCCTTCAGTATCTGTGATGAGCTCATTGGTC
GCGATTGTTTTGCATGTAATTGTGGTTGAAAGGTAGTGAATTTGCCATCTGAATCAGTCGGCAACTAATTAATGCTTTTTCTATTAT
ATAAAGTATAGATTGTACTA
>PhCRN30.2_700 PLHAL7001291
CAATTAAGAAGGAGCAGGAGTTTGACTTCGCGGCCAACAAATTAGAGCTATATCTCGCGAAGAAAGACAAGGGCCGTGGCGCGTGGG
TGACGGAAGAAGAAGCGAGGGGTTTGAATGGCACAAGTGATTTGGAGATATTGGACGTTGCGAGAGCACCGCTTAAATTTGTTGGCT
TATCGGAGGAAGACGTCCGGTTTCAAGTCACGAAGGAAGACGTGAAGGCAAAAACGACTCCTGTCCACGTGCTAGTGGTGGTTCCGG
GGAGTGCAGTGGATGATGGCGCTTGGAGCGCTAAGGAATTTTCTACCGTGGACCCTCAAGTTCAACTAAAAATGTGGAGGTCAGTTG
TCAGAGTTTCCTCAGAAGATGTATGCTCAGGAACAGGAGTGGTTGTGGATTTAACGTCGACGCACTTGTATCTGCTGACGAACTTGC
ACCTTTGGGTCGACGACACATTCACTGATCACTTGAGTTCTGACTTCACGAAGGAAATCAAACGGTATCGGAGACGCCACCCCCGGA
TAAAAACAAGTGGAAGGAAAAGGAACGCCACCAATGTAAAGGACGCCGATGTTGCAAAGCAACCACGACGTAAATCTACACGGACGG
CAGCGATGAAATCATCAGCTGGATTAGACAAGCCTCAAGTTATGGTGGAGCAACTCCTTTTCGATAGGACGAAGCCCGAAGAAGTCC
ACCGATTCAGTCTCGACAGTGACATTTGCTGGTGTAGTTCGGCTGCGTTCGACTTCGCAATCTTCAAAGTTAGTTTGCCACGAGATA
A
>PhCRN30.2_730 PLHAL7301188
CAATTAAGAAGGAGCAGGAGTTTGACTTCGCGGCCAACAAATTAGAGCTATATCTCGCGAAGAAAGACAAGGGCCGTGGCGCGTGGG
TGACGGAAGAAGAAGCGAGGGGTTTGAATGGCACAAGTGATTTGGAGATATTGGACGTTGCGAGAGCACCGCTTAAATTTGTTGGCT
TATCGGAGGAAGACGTCCGGTTTCAAGTCACGAAGGAAGACGTGAAGGCAAAAACGACTCCTGTCCACGTGCTAGTGGTGGTTCCGG
GGAGTGCAGTGGATGATGGCGCTTGGAGCGCTAAGGAATTTTCTACCGTGGACCCTCAAGTTCAACTAAAAATGTGGAGGTCAGTTG
TCAGAGTTTCCTCAGAAGATGTATGCTCAGGAACAGGAGTGGTTGTGGATTTAACGTCGACGCACTTGTATCTGCTGACGAACTTGC
ACCTTTGGGTCGACGACACATTCACTGATCACTTGAGTTCTGACTTCACGAAGGAAATCAAACGGTATCGGAGACGCCACCCCCGGA
TAAAAACAAGTGGAAGGAAAAGGAACGCCACCAATGTAAAGGACGCCGATGTTGCAAAGCAACCACGACGTAAATCTACACGGACGG
CAGCGATGAAATCATCAGCTGGATTAGACAAGCCTCAAGTTATGGTGGAGCAACTCCTTTTCGATAGGACGAAGCCCGAAGAAGTCC
ACCGATTCAGTCTCGACAGTGACATTTGCTGGTGTAGTTCGGCTGCGTTCGACTTCGCAATCTTCAAAGTTGCTTTGCCACGAGATA
ACAAGCTCGACCGATGCACAATATCCCTCAAGGTGAC

>PhCRN31_100 PLHAL100A10253
GCTTTAATCAAAGCGCTGACACATGAAGACTGTGAACTTGAGGGGAGTTTGGTCTTGAGGACATTTTTGAGGGTATGCCAGAACCGA
AAATCAAGCAAATTGACGTGTTAGTGATATTTTCCACCGCATCACGACCCCACTTCTTAGCCTTGACTCTTCGTGCCCCAAGTTCTC
ACAAACGCCTCAACGACTCTGTCTGAATCAAATCGATACTTCAAAACAATTTGTGTAACTACTATGGATGCTACAGCCAAAAGAAAT
CAAGGGCGCGATGTATGTTGCTCAATGTTGCTTTTTAAAAATCGCTTGTGACTGCGTCACACCTATTTCGTCGCAGCAACGGATAAA
TGTTGCTAGTAATGATGCAGATTTCTGACATCGACGATGTGAAGAATGGATTGTTATTGTTCAGGCCTTTGAAGTACGCATTCAATC
ATTTCCAAATTAGCTTCATTCGCGACGAAGCGGACGCTTTTCGCTTGAGAATCTTTGATCCATCTATCCGTGGCATCTCTCTCATTG
ATCTGGCAGACCACAACGGCATCATAGTGTTTAGCGCAGATCAGATACGATTGCTCCACATCAGCGTGTCGTCGTCGAAGAAACCAT
GCCGCTTCGATGTCCGGACGACGTTTGGTGACGTAAATGGCAGTACTCTGACATTCACTGGTTTAGAACGACCGTTCTACCGATGTT
TAAATTTACAGGCAAGAGTGGCGCGCATGGTTGCTCTGATGAAGAACTGAAACGATGCATCAGATGACTTTCAAGATTTCTGAAAGG
AAGTTTCTTTGCACGACAAAATGGAGGGATTTCACCGTAGCATGTTGGCTTCTGATACAGCGTAATTAAGCATCATATTTTCCGTGT
GTATTATGCGTGCGAGTGAATTTTCCGCTTTGATCATTTTTTCGTTCCCGACCTGTTGCCTATCCAACAAATTGATCCGAATCCCGA
TTTTGCCGATTAATCCAAACGAAACTGAGCAGCTCCACTTCGTCTTATGTGACCTTCGCACAGGTTTTTGGTTATTGTGGGTGAAGC
AGTATGTCTTAATGATCAGCTCCACTGGGTTGTTGCATGCGGACCCTTATCTTTCAAAACTTCGCGC
>PhCRN31_334 PLHAL3340276
GCTTTAATCAAAGCGCTGACACATGAAGACTGTGAACTTGAGGGGAGTTTAGTCTTGAGGACATTTTTGAGGGTATGCCAGAACCGA
AAATCAAGCAAATTGACGTGTTAGTGATATTTTCCACCGCATCACGACCCCACTTCTTAGCCTTGACTCTTCGTGCCCCAAGTTCTC
ACAAACGCCTCAACGACTCTGTCTGAATCAAATCGATACTTCAAAACAATTTGTGTAACTACTATGGATGCTACAGCCAAAAGAAAT
CAAGGGCGCGATGTATGTTGCTCAATGTTGCTTTTTAAAAATCGCTTGTGACTGCGTCACACCTATTTCGTCGCAGCAACGGATAAA
TGTTGCTAGTAATGATGCAGATTTCTGACATCGACGATGTGAAGAATGGATTGTTATTGTTCAGGCCTTTGAAGTACGCATTCAATC
ATTTCCAAATTAGCTTCATTCGCGACGAAGCGGACGCTTTTCGCTTGAGAATCTTTGATCCATCTATCCGTGGCATCTCTCTCATTG

ATCTGGCAGACCACAACGGCATCATAGTGTTTAGCGCAGATCAGATACGATTGCTCCACATCAGCGTGTCGTCGTCGAAGAAACCAT
GCCGCTTCGATGTCCGGACGACGTTTGGTGACGTAAATGGCAGTACTCTGACATTCACTGGTTTAGAACGACCGTTCTACCGATGTT
TAAATTTACAGGCAAGAGTGGCGCGCATGGTTGTTCTGATGAAGAACTGAAACGATGCATCAGATGACTTTCAAGATTTCTGAAAGG
AAGTTTCTTTGCACGACAAAATGGAGGGATTTCACCGTAGCATGTTGGCTTCTGATACAGCGTAATTAAGCATCATATTTTCCGTGT
GTATTATGCGTGCGAGTGAATTTTCCGCTTTGATCATTTTTTCGTTCCCGACCTGTTGCCTATCCAACAAATTGATCCGAATCCCGA
TTTTGCCGATTAATCCAAACGAAACTGAGCAGCTCCACTTCGTCTTATGTGACCTTCGCACACGTTTTTGGTTATTGTGGGTGAAGC
AGTATGTCTTAATGATCAGCTCCACTGGGTTGTTGCATGCGGACCCTTATCTTTCAAAACTTCGCGC
>PhCRN31_703 PLHAL7030280
GCTTTAATCAAAGCGCTGACACATGAAGACTGTGAACTTGAGGGGAGTTTGGTCTTGAGGACATTTTTGAGGGTATGCCAGAACCGA
AAATCAAGCAAATTGACGTGTTAGTGATATTTTCCACCGCATCACGACCCCACTTCTTAGCCTTGACTCTTCGTGCCCCAAGTTCTC
ACAAACGCCTCAACGACTCTGTCTGAATCAAATCGATACTTCAAAACAATTTGTGTAACTACTATGGATGCTACAGCCAAAAGAAAT
CAAGGGCGCGATGTATGTTGCTCAATGTTGCTTTTTAAAAATCGCTTGTGACTGCGTCACACCTATTTCGTCGCAGCAACGGATAAA
TGTTGCTAGTAATGATGCAGATTTCTGACATCGACGATGTGAAGAATGGATTGTTATTGTTCAGGCCTTTGAAGTACGCATTCAATC
ATTTCCAAATTAGCTTCATTCGCGACGAAGCGGACGCTTTTCGCTTGAGAATCTTTGATCCATCTATCCGTGGCATCTCTCTCATTG
ATCTGGCAGACCACAACGGCATCATAGTGTTTAGCGCAGATCAGATACGATTGCTCCACATCAGCGTGTCGTCGTCGAAGAAACCAT
GCCGCTTCGATGTCCGGACGACGTTTGGTGACGTAAATGGCAGTACTCTGACATTCACTGGTTTAGAACGACCGTTCTACCGATGTT
TAAATTTACAGGCAAGAGTGGCGCGCATGGTTGCTCTGATGAAGAACTGAAACGATGCATCAGATGACTTTCAAGATTTCTGAAAGG
AAGTTTCTTTGCACGACAAAATGGAGGGATTTCACCGTAGCATGTTGGCTTCTGATACAGCGTAATTAAGCATCATATTTTCCGTGT
GTATTATGCGTGCGAGTGAATTTTCCGCTTTGATCATTTTTTCGTTCCCGACCTGTTGCCTATCCAACAAATTGATCCGAATCCCGA
TTTTGCCGATTAATCCAAACGAAACTGAGCAGCTCCACTTCGTCTTATGTGACCTTCGCACAGGTTTTTGGTTATTGTGGGTGAAGC
AGTATGTCTTAATGATCAGCTCCACTGGGTTGTTGCATGCGGACCCTTATCTTTCAAAACTTCGCGC
>PhCRN31_710 PLHAL7100031
GCTTTAATCAAAGCGCTGACACATGAAGACTGTGAACTTGAGGGGAGTTTGGTCTTGAGGACATTTTTGAGGGTATGCCAGAACCGA
AAATCAAGCAAATTGACGTGTTAGTGATATTTTCCACCGCATCACGACCCCACTTCTTAGCCTTGACTCTTCGTGCCCCAAGTTCTC
ACAAACGCCTCAACGACTCTGTCTGAATCAAATCGATACTTCAAAACAATTTGTGTAACTACTATGGATGCTACAGCCAAAAGAAAT
CAAGGGCGCGATGTATGTTGCTCAATGTTGCTTTTTAAAAATCGCTTGTGACTGCGTCACACCTATTTCGTCGCAGCAACGGATAAA
TGTTGCTAGTAATGATGCAGATTTCTGACATCGACGATGTGAAGAATGGATTGTTATTGTTCAGGCCTTTGAAGTACGCATTCAATC
ATTTCCAAATTAGCTTCATTCGCGACGAAGCGGACGCTTTTCGCTTGAGAATCTTTGATCCATCTATCCGTGGCATCTCTCTCATTG
ATCTGGCAGACCACAACGGCATCATAGTGTTTAGCGCAGATCAGATACGATTGCTCCACATCAGCGTGTCGTCGTCGAAGAAACCAT
GCCGCTTCGATGTCCGGACGACGTTTGGTGACGTAAATGGCAGTACTCTGACATTCACTGGTTTAGAACGACCGTTCTACCGATGTT
TAAATTTACAGGCAAGAGTGGCGCGCATGGTTGCTCTGATGAAGAACTGAAACGATGCATCAGATGACTTTCAAGATTTCTGAAAGG
AAGTTTCTTTGCACGACAAAATGGAGGGATTTCACCGTAGCATGTTGGCTTCTGATACAGCGTAATTAAGCATCATATTTTCCGTGT
GTATTATGCGTGCGAGTGAATTTTCCGCTTTGATCATTTTTTCGTTCCCGACCTGTTGCCTATCCAACAAATTGATCCGAATCCCGA
TTTTGCCGATTAATCCAAACGAAACTGAGCAGCTCCACTTCGTCTTATGTGACCTTCGCACAGGTTTTTGGTTATTGTGGGTGAAGC
AGTATGTCTTAATGATCAGCTCCACTGGGTTGTTGCATGCGGACCCTTATCTTTCAAAACTTCGCGC
>PhCRN31_304 PLHAL3040005
GCTTTAATCAAAGCGCTGACACATGAAGACTGTGAACTTGAGGGGAGTTTGGTCTTGAGGACATTTTTGAGGGTATGCCAGAACCGA
AAATCAAGCAAATTGACGTGTTAGTGATATTTTCCACCGCATCACGACCCCACTTCTTAGCCTTGACTCTTCGTGCCCCAAGTTCTC
ACAAACGCCTCAACGACTCTGTCTGAATCAAATCGATACTTCAAAACAATTTGTGTAACTACTATGGATGCTACAGCCAAAAGAAAT
CAAGGGCGCGATGTATGTTGCTCAATGTTGCTTTTTAAAAATCGCTTGTGACTGCGTCACACCTATTTCGTCGCAGCAACGGATAAA
TGTTGCTAGTAATGATGCAGATTTCTGACATCGACGATGTGAAGAATGGATTGTTATTGTTCAGGCCTTTGAAGTACGCATTCAATC
ATTTCCAAATTAGCTTCATTCGCGACGAAGCGGACGCTTTTCGCTTGAGAATCTTTGATCCATCTATCCGTGGCATCTCTCTCATTG
ATCTGGCAGACCACAACGGCATCATAGTGTTTAGCGCAGATCAGATACGATTGCTCCACATCAGCGTGTCGTCGTCGAAGAAACCAT
GCCGCTTCGATGTCCGGACGACGTTTGGTGACGTAAATGGCAGTACTCTGACATTCACTGGTTTAGAACGACCGTTCTACCGATGTT
TAAATTTACAGGCAAGAGTGGCGCGCATGGTTGCTCTGATGAAGAACTGAAACGATGCATCAGATGACTTTCAAGATTTCTGAAAGG
AAGTTTCTTTGCACGACAAAATGGAGGGATTTCACCGTAGCATGTTGGCTTCTGATACAGCGTAATTAAGCATCATATTTTCCGTGT
GTATTATGCGTGCGAGTGAATTTTCCGCTTTGATCATTTTTTCGTTCCCGACCTGTTGCCTATCCAACAAATTGATCCGAATCCCGA
TTTTGCCGATTAATCCAAACGAAACTGAGCAGCTCCACTTCGTCTTATGTGACCTTCGCACAGGTTTTTGGTTATTGTGGGTGAAGC
AGTATGTCTTAATGATCAGCTCCACTGGGTTGTTGCATGCGGACCCTTATCTTTCAAAACTTCGCGC
>PhCRN31_700 PLHAL7000114
GCTTTAATCAAAGCGCTGACACATGAAGACTGTGAACTTGAGGGGAGTTTGGTCTTGAGGACATTTTTGAGGGTATGCCAGAACCGA
AAATCAAGCAAATTGACGTGTTAGTGATATTTTCCACCGCATCACGACCCCACTTCTTAGCCTTGACTCTTCGTGCCCCAAGTTCTC
ACAAACGCCTCAACGACTCTGTCTGAATCAAATCGATACTTCAAAACAATTTGTGTAACTACTATGGATGCTACAGCCAAAAGAAAT
CAAGGGCGCGATGTATGTTGCTCAATGTTGCTTTTTAAAAATCGCTTGTGACTGCGTCACACCTATTTCGTCGCAGCAACGGATAAA
TGTTGCTAGTAATGATGCAGATTTCTGACATCGACGATGTGAAGAATGGATTGTTATTGTTCAGGCCTTTGAAGTACGCATTCAATC
ATTTCCAAATTAGCTTCATTCGCGACGAAGCGGACGCTTTTCGCTTGAGAATCTTTGATCCATCTATCCGTGGCATCTCTCTCATTG
ATCTGGCAGACCACAACGGCATCATAGTGTTTAGCGCAGATCAGATACGATTGCTCCACATCAGCGTGTCGTCGTCGAAGAAACCAT
GCCGCTTCGATGTCCGGACGACGTTTGGTGACGTAAATGGCAGTACTCTGACATTCACTGGTTTAGAACGACCGTTCTACCGATGTT
TAAATTTACAGGCAAGAGTGGCGCGCATGGTTGTTCTGATGAAGAACTGAAACGATGCATCAGATGACTTTCAAGATTTCTGAAAGG
AAGTTTCTTTGCACGACAAAATGGAGGGATTTCACCGTAGCATGTTGGCTTCTGATACAGCGTAATTAAGCATCATATTTTCCGTGT
GTATTATGCGTGCGAGTGAATTTTCCGCTTTGATCATTTTTTCGTTCCCGACCTGTTGCCTATCCAACAAATTGATCCGAATCCCGA
TTTTGCCGATTAATCCAAACGAAACTGAGCAGCTCCACTTCGTCTTATGTGACCTTCGCACACGTTTTTGGTTATTGTGGGTGAAGC
AGTATGTCTTAATGATCAGCTCCACTGGGTTGTTGCATGCGGACCCTTATCTTTCAAAACTTCGCGC
>PhCRN31_730 PLHAL7300264
GCTTTAATCAAAGCGCTGACACATGAAGACTGTGAACTTGAGGGGAGTTTAGTCTTGAGGACATTTTTGAGGGTATGCCAGAACCGA
AAATCAAGCAAATTGACGTGTTAGTGATATTTTCCACCGCATCACGACCCCACTTCTTAGCCTTGACTCTTCGTGCCCCAAGTTCTC
ACAAACGCCTCAACGACTCTGTCTGAATCAAATCGATACTTCAAAACAATTTGTGTAACTACTATGGATGCTACAGCCAAAAGAAAT

CAAGGGCGCGATGTATGTTGCTCAATGTTGCTTTTTAAAAATCGCTTGTGACTGCGTCACACCTATTTCGTCGCAGCAACGGATAAA
TGTTGCTAGTAATGATGCAGATTTCTGACATCGACGATGTGAAGAATGGATTGTTATTGTTCAGGCCTTTGAAGTACGCATTCAATC
ATTTCCAAATTAGCTTCATTCGCGACGAAGCGGACGCTTTTCGCTTGAGAATCTTTGATCCATCTATCCGTGGCATCTCTCTCATTG
ATCTGGCAGACCACAACGGCATCATAGTGTTTAGCGCAGATCAGATACGATTGCTCCACATCAGCGTGTCGTCGTCGAAGAAACCAT
GCCGCTTCGATGTCCGGACGACGTTTGGTGACGTAAATGGCAGTACTCTGACATTCACTGGTTTAGAACGACCGTTCTACCGATGTT
TAAATTTACAGGCAAGAGTGGCGCGCATGGTTGTTCTGATGAAGAACTGAAACGATGCATCAGATGACTTTCAAGATTTCTGAAAGG
AAGTTTCTTTGCACGACAAAATGGAGGGATTTCACCGTAGCATGTTGGCTTCTGATACAGCGTAATTAAGCATCATATTTTCCGTGT
GTATTATGCGTGCGAGTGAATTTTCCGCTTTGATCATTTTTTCGTTCCCGACCTGTTGCCTATCCAACAAATTGATCCGAATCCCGA
TTTTGCCGATTAATCCAAACGAAACTGAGCAGCTCCACTTCGTCTTATGTGACCTTCGCACACGTTTTTGGTTATTGTGGGTGAAGC
AGTATGTCTTAATGATCAGCTCCACTGGGTTGTTGCATGCGGACCCTTATCTTTCAAAACTTCGCGC

>PhCRN32_100 PLHAL100A10253
GAGAACTCAAAGAGCAACTGGAGACATGTTGAAGCTGTTCTGGCGCAGTCGTTGGCGTGAAAGGTAGCGCGTTTCCAAATGACTATC
GACGCGACTGAGTCTGTGGATGACTTGAAGAAAGCCATCAAGAAGGAGAAGAAGAAAAGTCTGAAAATGGTCGATGCGGACAAGCTG
CAGCTGTTCCTCGCCAGACAGGGCGACAATACATGGCTCGAAACAAGCACAGATGATGGGGAGAAGCTTAAGAAAGGGGCAACGACT
GCTTTAATCAAAGCGCTGACACATGAAGACTGTGAACTTGAGGGGAGTTTGGTCTTGAGGACATTTTTGAGGGTATGCCAGAACCGA
AAATCAAGCAAATTGACGTGTTAGTGATATTTTCCACCGCATCACGACCCCACTTCTTAGCCTTGACTCTTCGTGCCC
>PhCRN32_334 PLHAL3340276
GAGAACTCAAAGAGCAACTGGAGACATGTTGAAGCTGTTCTGGCGCAGTCGTTGGCGTGAAAGGTAGCGCGTTTCCAAATGACTATC
GACGCGACTGAGTCTGTGGATGACTTGAAGAAAGCCATCAAGAAGGAGAAGAAGAAAAGTCTGAAAATGGTCGATGCGGACAAGCTG
CAGCTGTTCCTCGCCAGACAGGGCGACAATACATGGCTCGAAACAAGCACAGATGATGGGGAGAAGCTTAAGAAAGGGGCAACGACT
GCTTTAATCAAAGCGCTGACACATGAAGACTGTGAACTTGAGGGGAGTTTAGTCTTGAGGACATTTTTGAGGGTATGCCAGAACCGA
AAATCAAGCAAATTGACGTGTTAGTGATATTTTCCACCGCATCACGACCCCACTTCTTAGCCTTGACTCTTCGTGCCC
>PhCRN32_703 PLHAL7030280
GAGAACTCAAAGAGCAACTGGAGACATGTTGAAGCTGTTCTGGCGCAGTCGTTGGCGTGAAAGGTAGCGCGTTTCCAAATGACTATC
GACGCGACTGAGTCTGTGGATGACTTGAAGAAAGCCATCAAGAAGGAGAAGAAGAAAAGTCTGAAAATGGTCGATGCGGACAAGCTG
CAGCTGTTCCTCGCCAGACAGGGCGACAATACATGGCTCGAAACAAGCACAGATGATGGGGAGAAGCTTAAGAAAGGGGCAACGACT
GCTTTAATCAAAGCGCTGACACATGAAGACTGTGAACTTGAGGGGAGTTTGGTCTTGAGGACATTTTTGAGGGTATGCCAGAACCGA
AAATCAAGCAAATTGACGTGTTAGTGATATTTTCCACCGCATCACGACCCCACTTCTTAGCCTTGACTCTTCGTGCCC
>PhCRN32_710 PLHAL7100031
GAGAACTCAAAGAGCAACTGGAGACATGTTGAAGCTGTTCTGGCGCAGTCGTTGGCGTGAAAGGTAGCGCGTTTCCAAATGACTATC
GACGCGACTGAGTCTGTGGATGACTTGAAGAAAGCCATCAAGAAGGAGAAGAAGAAAAGTCTGAAAATGGTCGATGCGGACAAGCTG
CAGCTGTTCCTCGCCAGACAGGGCGACAATACATGGCTCGAAACAAGCACAGATGATGGGGAGAAGCTTAAGAAAGGGGCAACGACT
GCTTTAATCAAAGCGCTGACACATGAAGACTGTGAACTTGAGGGGAGTTTGGTCTTGAGGACATTTTTGAGGGTATGCCAGAACCGA
AAATCAAGCAAATTGACGTGTTAGTGATATTTTCCACCGCATCACGACCCCACTTCTTAGCCTTGACTCTTCGTGCCC
>PhCRN32_304 PLHAL3040005
GAGAACTCAAAGAGCAACTGGAGACATGTTGAAGCTGTTCTGGCGCAGTCGTTGGCGTGAAAGGTAGCGCGTTTCCAAATGACTATC
GACGCGACTGAGTCTGTGGATGACTTGAAGAAAGCCATCAAGAAGGAGAAGAAGAAAAGTCTGAAAATGGTCGATGCGGACAAGCTG
CAGCTGTTCCTCGCCAGACAGGGCGACAATACATGGCTCGAAACAAGCACAGATGATGGGGAGAAGCTTAAGAAAGGGGCAACGACT
GCTTTAATCAAAGCGCTGACACATGAAGACTGTGAACTTGAGGGGAGTTTGGTCTTGAGGACATTTTTGAGGGTATGCCAGAACCGA
AAATCAAGCAAATTGACGTGTTAGTGATATTTTCCACCGCATCACGACCCCACTTCTTAGCCTTGACTCTTCGTGCCC
>PhCRN32_700 PLHAL7000114
GAGAACTCAAAGAGCAACTGGAGACATGTTGAAGCTGTTCTGGCGCAGTCGTTGGCGTGAAAGGTAGCGCGTTTCCAAATGACTATC
GACGCGACTGAGTCTGTGGATGACTTGAAGAAAGCCATCAAGAAGGAGAAGAAGAAAAGTCTGAAAATGGTCGATGCGGACAAGCTG
CAGCTGTTCCTCGCCAGACAGGGCGACAATACATGGCTCGAAACAAGCACAGATGATGGGGAGAAGCTTAAGAAAGGGGCAACGACT
GCTTTAATCAAAGCGCTGACACATGAAGACTGTGAACTTGAGGGGAGTTTGGTCTTGAGGACATTTTTGAGGGTATGCCAGAACCGA
AAATCAAGCAAATTGACGTGTTAGTGATATTTTCCACCGCATCACGACCCCACTTCTTAGCCTTGACTCTTCGTGCCC
>PhCRN32_730 PLHAL7300264
GAGAACTCAAAGAGCAACTGGAGACATGTTGAAGCTGTTCTGGCGCAGTCGTTGGCGTGAAAGGTAGCGCGTTTCCAAATGACTATC
GACGCGACTGAGTCTGTGGATGACTTGAAGAAAGCCATCAAGAAGGAGAAGAAGAAAAGTCTGAAAATGGTCGATGCGGACAAGCTG
CAGCTGTTCCTCGCCAGACAGGGCGACAATACATGGCTCGAAACAAGCACAGATGATGGGGAGAAGCTTAAGAAAGGGGCAACGACT
GCTTTAATCAAAGCGCTGACACATGAAGACTGTGAACTTGAGGGGAGTTTAGTCTTGAGGACATTTTTGAGGGTATGCCAGAACCGA
AAATCAAGCAAATTGACGTGTTAGTGATATTTTCCACCGCATCACGACCCCACTTCTTAGCCTTGACTCTTCGTGCCC

>PhCRN33_100 PLHAL100A12150
GTCGCAATCTCTTCATTTAATTCATCAGCATGGTCACTCGCATTCACTAAGAATGGCTCTATTGTCCCCACGTCTCATATTAAAGCT
AATAAGATGCAACAACACATCTGTAGATCAATTAATATCTATTATATTGACTAGTTTGATTGACTGAGTAGGTCAAAAGATGGTTGA
CAACTTCAACATTATCAGTACGTAATATTCATCATTATGTGTCAAAATGAATACATCACTGTAGAGCTACTTCACAGTTGAAAAAAA
TCAGCAAGTGGGAACATGGTGACGCTTTACTGCGCGATCGCTGGTTTGAAAAGAAATGCTTTCTCTGTTGACATCGACACTAGCCAG
TCGGTGGGCCACTTAAAGGATGCTATTGCGCAAGAGCAGAAGTTCGATTTTGCTGCCAGCAAATTAGAGCTATATCTCGCGAAGAAG
GGCGAATCGTGGCTGACTGAGAATGAGGTGAAGAATGGTGTTAGAGACATAAGTGGCCTGAAACATCTTAGTGCCGCGGGAGCAATA
CTTCACCGCATTGGGTTGTCTGATGAGCAAGTAGTGGGGGAAGTTGATGGAATTGAGGTAGATGCAGGAAATGGTCCTGTGAATGTG
TTGGTTGTTGTCCCGACCTTCGTCATAGTTGAAGAAGACGAAAGCTCAGAGAAATATCTGTCAGAGTTGACGTACTATCAACACTGT
GGATGTCTTATCCAATACAAATACCGAGATTATTGTGCACACATTTTGGACAAAGTCGACAAGTTCTATGACGAGAACGAGCGCCCA
ATCCCGTTCATCTGTGTGGAAGGGTC
>PhCRN33_334 PLHAL3342360

GTCGCAATCTCTTCATTTAATTCATCAGCATGGTCACTCGCATTCACTAAGAATGCTCTATTGTCCCCACGTCTCATATTAAAGCTA
ATAAGATGCAACAACACATCTGTAGATCAATTAATATCTATTATATTGACTAGTTTGATTGACTGAGTAGGTCAAAAGATGGTTGAC
AACTTCAACATTATCAGTACGTAATATTCATCATTATGTGTCAAAATGAATACATCACTGTAGAGCTACTTCACAGTTGAAAAAAAT
CAGCAAGTGGGAACATGGTGACGCTTTACTGCGCGATCGCTGGTTTGAAAAGAAATGCTTTCTCTGTTGACATCGACACTAGCCAGT
CGGTGGGCCACTTAAAGGATGCTATTGCGCAAGAGCAGAAGTTCGATTTTGCTGCCAGCAAATTAGAGCTATATCTCGCGAAGAAGG
GCGAATCGTGGCTGACTGAGAATGAGGTGAAGAATGGTGTTAGAGACATAAGTGGCCTGAAACATCTTAGTGCCGCGGGAGCAATAC
TTCACCGCATTGGGTTGTCTGATGAGCAAGTAGTGGGGGAAGTTGATGGAATTGAGGTAGATGCAGGAAATGGTCCTGTGAATGTGT
TGGTTGTTGTCCCGACCTTCGTCATAGTTGAAGAAGACGAAAGCTCAGAGAAATATCTGTCAGAGTTGACGTACTATCAACACTGTG
GACGTCTTATCCAATACAAATACCGAGATTATTGTGCACACATTTTGGACAAAGTCGACAAGTTCTATGACGAGAACGAGCGCCCAA
TCCCGTTCATCTGTGTGGAAGGGTC
>PhCRN33_703 PLHAL7030233
GTCGCAATCTCTTCATTTAATTCATCAGCATGGTCACTCGCATTCACTAAGAATGGCTCTATTGTCCCCACGTCTCATATTAAAGCT
AATAAGATGCAACAACACATCTGTAGATCAATTAATATCTATTATATTGACTAGTTTGATTGACTGAGTAGGTCAAAAGATGGTTGA
CAACTTCAACATTATCAGTACGTAATATTCATCATTATGTGTCAAAATGAATACATCACTGTAGAGCTACTTCACAGTTGAAAAAAA
TCAGCAAGTGGGAACATGGTGACGCTTTACTGCGCGATCGCTGGTTTGAAAAGAAATGCTTTCTCTGTTGACATCGACACTAGCCAG
TCGGTGGGCCACTTAAAGGATGCTATTGCGCAAGAGCAGAAGTTCGATTTTGCTGCCAGCAAATTAGAGCTATATCTCGCGAAGAAG
GGCGAATCGTGGCTGACTGAGAATGAGGTGAAGAATGGTGTTAGAGACATAAGTGGCCTGAAACATCTTAGTGCCGCGGGAGCAATA
CTTCACCGCATTGGGTTGTCTGATGAGCAAGTAGTGGGGGAAGTTGATGGAATTGAGGTAGATGCAGGAAATGGTCCTGTGAATGTG
TTGGTTGTTGTCCCGACCTTCGTCATAGTTGAAGAAGACGAAAGCTCAGAGAAATATCTGTCAGAGTTGACGTACTATCAACACTGT
GGATGTCTTATCCAATACAAATACCGAGATTATTGTGCACACATTTTGGACAAAGTCGACAAGTTCTATGACGAGAACGAGCGCCCA
ATCCCGTTCATCTGTGTGGAAGGGTC
>PhCRN33_710 PLHAL7101177
GTCGCAATCTCTTCATTTAATTCATCAGCATGGTCACTCGCATTCACTAAGAATGCTCTATTGTCCCCACGTCTCATATTAAAGCTA
ATAAGATGCAACAACACATCTGTAGATCAATTAATATCTATTATATTGACTAGTTTGATTGACTGAGTAGGTCAAAAGATGGTTGAC
AACTTCAACATTATCAGTACGTAATATTCATCATTATGTGTCAAAATGAATACATCACTGTAGAGCTACTTCACAGTTGAAAAAAAT
CAGCAAGTGGGAACATGGTGACGCTTTACTGCGCGATCGCTGGTTTGAAAAGAAATGCTTTCTCTGTTGACATCGACACTAGCCAGT
CGGTGGGCCACTTAAAGGATGCTATTGCGCAAGAGCAGAAGTTCGATTTTGCTGCCAGCAAATTAGAGCTATATCTCGCGAAGAAGG
GCGAATCGTGGCTGACTGAGAATGAGGTGAAGAATGGTGTTAGAGACATAAGTGGCCTGAAACATCTTAGTGCCGCGGGAGCAATAC
TTCACCGCATTGGGTTGTCTGATGAGCAAGTAGTGGGGGAAGTTGATGGAATTGAGGTAGATGCAGGAAATGGTCCTGTGAATGTGT
TGGTTGTTGTCCCGACCTTCGTCATAGTTGAAGAAGACGAAAGCTCAGAGAAATATCTGTCAGAGTTGACGTACTATCAACACTGTG
GACGTCTTATCCAATACAAATACCGAGATTATTGTGCACACATTTTGGACAAAGTCGACAAGTTCTATGACGAGAACGAGCGCCCAA
TCCCGTTCATCTGTGTGGAAGGGTC
>PhCRN33_304 PLHAL3041339
GTCGCAATCTCTTCATTTAATTCATCAGCATGGTCACTCGCATTCACTAAGAATGGCTCTATTGTCCCCACGTCTCATATTAAAGCT
AATAAGATGCAACAACACATCTGTAGATCAATTAATATCTATTATATTGACTAGTTTGATTGACTGAGTAGGTCAAAAGATGGTTGA
CAACTTCAACATTATCAGTACGTAATATTCATCATTATGTGTCAAAATGAATACATCACTGTAGAGCTACTTCACAGTTGAAAAAAA
TCAGCAAGTGGGAACATGGTGACGCTTTACTGCGCGATCGCTGGTTTGAAAAGAAATGCTTTCTCTGTTGACATCGACACTAGCCAG
TCGGTGGGCCACTTAAAGGATGCTATTGCGCAAGAGCAGAAGTTCGATTTTGCTGCCAGCAAATTAGAGCTATATCTCGCGAAGAAG
GGCGAATCGTGGCTGACTGAGAATGAGGTGAAGAATGGTGTTAGAGACATAAGTGGCCTGAAACATCTTAGTGCCGCGGGAGCAATA
CTTCACCGCATTGGGTTGTCTGATGAGCAAGTAGTGGGGGAAGTTGATGGAATTGAGGTAGATGCAGGAAATGGTCCTGTGAATGTG
TTGGTTGTTGTCCCGACCTTCGTCATAGTTGAAGAAGACGAAAGCTCAGAGAAATATCTGTCAGAGTTGACGTACTATCAACACTGT
GGATGTCTTATCCAATACAAATACCGAGATTATTGTGCACACATTTTGGACAAAGTCGACAAGTTCTATGACGAGAACGAGCGCCCA
ATCCCGTTCATCTGTGTGGAAGGGTC
>PhCRN33_700 PLHAL7001118
GTCGCAATCTCTTCATTTAATTCATCAGCATGGTCACTCGCATTCACTAAGAATGGCTCTATTGTCCCCACGTCTCATATTAAAGCT
AATAAGATGCAACAACACATCTGTAGATCAATTAATATCTATTATATTGACTAGTTTGATTGACTGAGTAGGTCAAAAGATGGTTGA
CAACTTCAACATTATCAGTACGTAATATTCATCATTATGTGTCAAAATGAATACATCACTGTAGAGCTACTTCACAGTTGAAAAAAA
TCAGCAAGTGGGAACATGGTGACGCTTTACTGCGCGATCGCTGGTTTGAAAAGAAATGCTTTCTCTGTTGACATCGACACTAGCCAG
TCGGTGGGCCACTTAAAGGATGCTATTGCGCAAGAGCAGAAGTTCGATTTTGCTGCCAGCAAATTAGAGCTATATCTCGCGAAGAAG
GGCGAATCGTGGCTGACTGAGAATGAGGTGAAGAATGGTGTTAGAGACATAAGTGGCCTGAAACATCTTAGTGCCGCGGGAGCAATA
CTTCACCGCATTGGGTTGTCTGATGAGCAAGTAGTGGGGGAAGTTGATGGAATTGAGGTAGATGCAGGAAATGGTCCTGTGAATGTG
TTGGTTGTTGTCCCGACCTTCGTCATAGTTGAAGAAGACGAAAGCTCAGAGAAATATCTGTCAGAGTTGACGTACTATCAACACTGT
GGATGTCTTATCCAATACAAATACCGAGATTATTGTGCACACATTTTGGACAAAGTCGACAAGTTCTATGACGAGAACGAGCGCCCA
ATCCCGTTCATCTGTGTGGAAGGGTC
>PhCRN33_730 PLHAL7301861
GTCGCAATCTCTTCATTTAATTCATCAGCATGGTCACTCGCATTCACTAAGAATGCTCTATTGTCCCCACGTCTCATATTAAAGCTA
ATAAGATGCAACAACACATCTGTAGATCAATTAATATCTATTATATTGACTAGTTTGATTGACTGAGTAGGTCAAAAGATGGTTGAC
AACTTCAACATTATCAGTACGTAATATTCATCATTATGTGTCAAAATGAATACATCACTGTAGAGCTACTTCACAGTTGAAAAAAAT
CAGCAAGTGGGAACATGGTGACGCTTTACTGCGCGATCGCTGGTTTGAAAAGAAATGCTTTCTCTGTTGACATCGACACTAGCCAGT
CGGTGGGCCACTTAAAGGATGCTATTGCGCAAGAGCAGAAGTTCGATTTTGCTGCCAGCAAATTAGAGCTATATCTCGCGAAGAAGG
GCGAATCGTGGCTGACTGAGAATGAGGTGAAGAATGGTGTTAGAGACATAAGTGGCCTGAAACATCTTAGTGCCGCGGGAGCAATAC
TTCACCGCATTGGGTTGTCTGATGAGCAAGTAGTGGGGGAAGTTGATGGAATTGAGGTAGATGCAGGAAATGGTCCTGTGAATGTGT
TGGTTGTTGTCCCGACCTTCGTCATAGTTGAAGAAGACGAAAGCTCAGAGAAATATCTGTCAGAGTTGACGTACTATCAACACTGTG
GACGTCTTATCCAATACAAATACCGAGATTATTGTGCACACATTTTGGACAAAGTCGACAAGTTCTATGACGAGAACGAGCGCCCAA
TCCCGTTCATCTGTGTGGAAGGGTC

>PhCRN34_100 PLHAL100A10975

TAAGATGCGAGACCTGGTCCACATCTAAAAAAGGTATCAGCAAGAAAAACATGGTGACGCTTTTCTGCGCAGTTGTCGGTGAGAACG
GAAATGCATTCCCTGTGGATATCGACACTAGCCAGTCGGTGGGTGACTTGAAAGAAGCCATTAAAGAAGAGCAGAAGTTTGATTTTG
CTGCCAGCAAATTAGAGCTATATCTCGCGAAGAAGGGCGAATCGTGGCTGACTGAGATTGAGGTGAAGGATATAAGTGACATAAGTG
GCCTGAAACATCTTAGTGCCGCGCGAGCTAGACTTCACCACATTGGGCTGTCTGATGATCAAGTAGTAGAGGAAGTTGATGAAATTG
AGGTAGATGCAGGAAATGGTTCTGTGAATGTGTTGGCGATTATTCCGATCGGAATGAAATATGCACTGGCTGCAAATCGAAGAGCCG
C
>PhCRN34_334 PLHAL3340990
TAAGATGCGAGACCTGGTCCACATCTAAAAAAGGTATCAGCAAGAAAAACATGGTGACGCTTTTCTGCGCAGTTGTCGGTGAGAACG
GAAATGCATTCCCTGTGGATATCGACACTAGCCAGTCGGTGGGTGACTTGAAAGAAGCCATTAAAGAAGAGCAGAAGTTTGATTTTG
CTGCCAGCAAATTAGAGCTATATCTCGCGAAGAAGGGCGAATCGTGGCTGACTGAGATTGAGGTGAAGGATATAAGTGACATAAGTG
ACCTGAAACATCTTAGTGCCGCGCGAGCTAGACTTCACCACATTGGGCTGTCTGATGATCAAGTAGTAGAGGAAGTTGATGAAATTG
AGGTAGATGCAGGAAATGGTTCAGTGAATGTGTTGGCGATTATTCCGATCGGAATGAAATATGCACTGGCTGCAAATCGAAGAGCCG
C
>PhCRN34_703 PLHAL7030960
TAAGATGCGAGACCTGGTCCACATCTAAAAAAGGTATCAGCAAGAAAAACATGGTGACGCTTTTCTGCGCAGTTGTCGGTGAGAACG
GAAATGCATTCCCTGTGGATATCGACACTAGCCAGTCGGTGGGTGACTTGAAAGAAGCCATTAAAGAAGAGCAGAAGTTTGATTTTG
CTGCCAGCAAATTAGAGCTATATCTCGCGAAGAAGGGCGAATCGTGGCTGACTGAGATTGAGGTGAAGGATATAAGTGACATAAGTG
ACCTGAAACATCTTAGTGCCGCGCGAGCTAGACTTCACCACATTGGGCTGTCTGATGATCAAGTAGTAGAGGAAGTTGATGAAATTG
AGGTAGATGCAGGAAATGGTTCAGTGAATGTGTTGGCGATTATTCCGATCGGAATGAAATATGCACTGGCTGCAAATCGAAGAGCCG
C
>PhCRN34_710 PLHAL7100892
TAAGATGCGAGACCTGGTCCACATCTAAAAAAGGTATCAGCAAGAAAAACATGGTGACGCTTTTCTGCGCAGTTGTCGGTGAGAACG
GAAATGCATTCCCTGTGGATATCGACACTAGCCAGTCGGTGGGTGACTTGAAAGAAGCCATTAAAGAAGAGCAGAAGTTTGATTTTG
CTGCCAGCAAATTAGAGCTATATCTCGCGAAGAAGGGCGAATCGTGGCTGACTGAGATTGAGGTGAAGGATATAAGTGACATAAGTG
GCCTGAAACATCTTAGTGCCGCGCGAGCTAGACTTCACCACATTGGGCTGTCTGATGATCAAGTAGTAGAGGAAGTTGATGAAATTG
AGGTAGATGCAGGAAATGGTTCTGTGAATGTGTTGGCGATTATTCCGATCGGAATGAAATATGCACTGGCTGCAAATCGAAGAGCCG
C
>PhCRN34_304 PLHAL3040955
TAAGATGCGAGACCTGGTCCACATCTAAAAAAGGTATCAGCAAGAAAAACATGGTGACGCTTTTCTGCGCAGTTGTCGGTGAGAACG
GAAATGCATTCCCTGTGGATATCGACACTAGCCAGTCGGTGGGTGACTTGAAAGAAGCCATTAAAGAAGAGCAGAAGTTTGATTTTG
CTGCCAGCAAATTAGAGCTATATCTCGCGAAGAAGGGCGAATCGTGGCTGACTGAGATTGAGGTGAAGGATATAAGTGACATAAGTG
GCCTGAAACATCTTAGTGCCGCGCGAGCTAGACTTCACCACATTGGGCTGTCTGATGATCAAGTAGTAGAGGAAGTTGATGAAATTG
AGGTAGATGCAGGAAATGGTTCTGTGAATGTGTTGGCGATTATTCCGATCGGAATGAAATATGCACTGGCTGCAAATCGAAGAGCCG
C
>PhCRN34_700 PLHAL7001016
TAAGATGCGAGACCTGGTCCACATCTAAAAAAGGTATCAGCAAGAAAAACATGGTGACGCTTTTCTGCGCAGTTGTCGGTGAGAACG
GAAATGCATTCCCTGTGGATATCGACACTAGCCAGTCGGTGGGTGACTTGAAAGAAGCCATTAAAGAAGAGCAGAAGTTTGATTTTG
CTGCCAGCAAATTAGAGCTATATCTCGCGAAGAAGGGCGAATCGTGGCTGACTGAGATTGAGGTGAAGGATATAAGTGACATAAGTG
ACCTGAAACATCTTAGTGCCGCGCGAGCTAGACTTCACCACATTGGGCTGTCTGATGATCAAGTAGTAGAGGAAGTTGATGAAATTG
AGGTAGATGCAGGAAATGGTTCAGTGAATGTGTTGGCGATTATTCCGATCGGAATGAAATATGCACTGGCTGCAAATCGAAGAGCCG
C
>PhCRN34_730 PLHAL7300987
TAAGATGCGAGACCTGGTCCACATCTAAAAAAGGTATCAGCAAGAAAAACATGGTGACGCTTTTCTGCGCAGTTGTCGGTGAGAACG
GAAATGCATTCCCTGTGGATATCGACACTAGCCAGTCGGTGGGTGACTTGAAAGAAGCCATTAAAGAAGAGCAGAAGTTTGATTTTG
CTGCCAGCAAATTAGAGCTATATCTCGCGAAGAAGGGCGAATCGTGGCTGACTGAGATTGAGGTGAAGGATATAAGTGACATAAGTG
ACCTGAAACATCTTAGTGCCGCGCGAGCTAGACTTCACCACATTGGGCTGTCTGATGATCAAGTAGTAGAGGAAGTTGATGAAATTG
AGGTAGATGCAGGAAATGGTTCAGTGAATGTGTTGGCGATTATTCCGATCGGAATGAAATATGCACTGGCTGCAAATCGAAGAGCCG
C

>PhCRN35_100 PLHAL100A11475
AGAAGTCGCCGCCTTGAGGTAAAATGATAAAGCTCAAGTGTGCGATCGTTGGTGCGGCGAGAAGCGTGTTCGAGGTGAAGATTGACG
ATGCTGAGTCCGTTTCAGCGTTGAAGGGGGTGATTAAGAAGAAGAAGATAGATACGATTAAGTGCGAAGCAGATAAGTTGGAGCTCT
ATCTAGCGAAGATTAAGGACGGGGGATGGTTGACGGATGACGATGCTTTGGATGCGATGTTACAGAGTGGAATTGATGTCACCTCCT
ATTTGAAGATGCGATCGTCGTGGAAGTTGAACAAACCAAATCTTTTTGGTCCTAATAATTCCCGAGAAGAGGATGTGGTTCACGTGT
TGGTGGTGGTTCCGGAGGG
>PhCRN35_334 PLHAL3341531
AGAAGTCGCCGCCTTGAGGTAAAATGATAAAGCTCAAGTGTGCGATCGTTGGTGCGGCGAGAAGCGTGTTCGAGGTGAAGATTGACG
ATGCTGAGTCCGTTTCAGCGTTGAAGGGGGTGATTAAGAAGAAGAAGATAGATACGATTAAGTGCGAAGCAGATAAGTTGGAGCTCT
ATCTAGCGAAGATTAAGGACGGGGGATGGTTGACGGATGACGATGCTTTGGATGCGATGTTACAGAGTGGAATTGATGTCACCTCCT
ATTTGAAGATGCGATCGTCGTGGAAGTTGAACAAACCAAATCTTTTTGGTCCTAATAATTCCCGAGAAGAGGATGTGGTTCACGTGT
TGGTGGTGGTTCCGGAGGG
>PhCRN35_703 PLHAL7031428
AGAAGTCGCCGCCTTGAGGTAAAATGATAAAGCTCAAGTGTGCGATCGTTGGTGCGGCGAGAAGCGTGTTCGAGGTGAAGATTGACG
ATGCTGAGTCCGTTTCAGCGTTGAAGGGGGTGATTAAGAAGAAGAAGATAGATACGATTAAGTGCGAAGCAGATAAGTTGGAGCTCT
ATCTAGCGAAGATTAAGGACGGGGGATGGTTGACGGATGACGATGCTTTGGATGCGATGTTACAGAGTGGAATTGATGTCACCTCCT
ATTTGAAGATGCGATCGTCGTGGAAGTTGAACAAACCAAATCTTTTTGGTCCTAATAATTCCCGAGAAGAGGATGTGGTTCACGTGT
TGGTGGTGGTTCCGGAGGG

>PhCRN35_710 PLHAL7101283
AGAAGTCGCCGCCTTGAGGTAAAATGATAAAGCTCAAGTGTGCGATCGTTGGTGCGGCGAGAAGCGTGTTCGAGGTGAAGATTGACG
ATGCTGAGTCCGTTTCAGCGTTGAAGGGGGTGATTAAGAAGAAGAAGATAGATACGATTAAGTGCGAAGCAGATAAGTTGGAGCTCT
ATCTAGCGAAGATTAAGGACGGGGGATGGTTGACGGATGACGATGCTTTGGATGCGATGTTACAGAGTGGAATTGATGTCACCTCCT
ATTTGAAGATGCGATCGTCGTGGAAGTTGAACAAACCAAATCTTTTTGGTCCTAATAATTCCCGAGAAGAGGATGTGGTTCACGTGT
TGGTGGTGGTTCCGGAGGG
>PhCRN35_304 PLHAL3041486
AGAAGTCGCCGCCTTGAGGTAAAATGATAAAGCTCAAGTGTGCGATCGTTGGTGCGGCGAGAAGCGTGTTCGAGGTGAAGATTGACG
ATGCTGAGTCCGTTTCAGCGTTGAAGGGGGTGATTAAGAAGAAGAAGATAGATACGATTAAGTGCGAAGCAGATAAGTTGGAGCTCT
ATCTAGCGAAGATTAAGGACGGGGGATGGTTGACGGATGACGATGCTTTGGATGCGATGTTACAGAGTGGAATTGATGTCACCTCCT
ATTTGAAGATGCGATCGTCGTGGAAGTTGAACAAACCAAATCTTTTTGGTCCTAATAATTCCCGAGAAGAGGATGTGGTTCACGTGT
TGGTGGTGGTTCCGGAGGG
>PhCRN35_700 PLHAL7001645
AGAAGTCGCCGCCTTGAGGTAAAATGATAAAGCTCAAGTGTGCGATCGTTGGTGCGGCGAGAAGCGTGTTCGAGGTGAAGATTGACG
ATGCTGAGTCCGTTTCAGCGTTGAAGGGGGTGATTAAGAAGAAGAAGATAGATACGATTAAGTGCGAAGCAGATAAGTTGGAGCTCT
ATCTAGCGAAGATTAAGGACGGGGGATATTTGACGGATGACGATGCTTTGGATGCGATGTTACAGAGTGGAATTGATGTCACCTCCT
ATTTGAAGATGCGGTCGTCGTGGAAGTTGAACAAACCAAATCTTTTTGGTCCTAATAATTCCCGAGAAGAGGATGTGGTTCACGTGT
TGGTGGTGGTTCCGGAGGG
>PhCRN35_730 PLHAL7301456
AGAAGTCGCCGCCTTGAGGTAAAATGATAAAGCTCAAGTGTGCGATCGTTGGTGCGGCGAGAAGCGTGTTCGAGGTGAAGATTGACG
ATGCTGAGTCCGTTTCAGCGTTGAAGGGGGTGATTAAGAAGAAGAAGATAGATACGATTAAGTGCGAAGCAGATAAGTTGGAGCTCT
ATCTAGCGAAGATTAAGGACGGGGGATATTTGACGGATGACGATGCTTTGGATGCGATGTTACAGAGTGGAATTGATGTCACCTCCT
ATTTGAAGATGCGATCGTCGTGGAAGTTGAACAAACCAAATCTTTTTGGTCCTAATAATTCCCGAGAAGAGGATGTGGTTCACGTGT
TGGTGGTGGTTCCGGAGGG

>PhCRN36_100 PLHAL100A13632
AGAATCCGCCGGCTCAAATACAAGATGGTAGAGGTTACCCTGATGTGTGCGGTAGTTCGGGAAGGGGGATTGGACGTCGTCCCTGTG
GATATCGACGAGCAGAAGACAGTAGGCCACCTGAAGGATGCGATTAAGGCGAAGAAGCCTGACACGATCAAGTGTGAAGCTGACCAG
CTGCGGCTGTTCCTGGCTAAGACAGCGGCGGGCGCGTGGCTCGAATCAAGCACAGATGATGTGAAGAAGCTCAAGAAGGGGGAAAAG
ACTGCGCTAATCGAAGCGCTGACACACGAAGACAAAGAGCTACAGGGAGAGGATTCGCTGGAGTACGTGCTGGAAGAAAACAATATG
CCCACCCCACAGTCGAGACAGAGTCACGTGCTGGTGGTGGTTCCAGAGGCGGTTGATCGGTCAGTGAGTGAGGCATCCAAGATGGAT
CAACTTGTCGAACAGGTCAACCAACTGAACGAC
>PhCRN36_334 PLHAL3343716
AGAATCCGCCGGCTCAAATACAAGATGGTAGAGGTTACCCTGATGTGTGCGGTAGTTCGGGAAGGGGGATTGGACGTCGTCCCTGTG
GATATCGACGAGCAGAAGACAGTAGGCCACCTGAAGGATGCGATTAAGGCGAAGAAGCCTGACACGATCAAGTGTGAAGCTGACCAG
CTGCGGCTGTTCCTGGCTAAGACAGCGGCGGGCGCGTGGCTCGAATCAAGCACAGATGATGTGAAGAAGCTCAAGAAGGGGGAAAAG
ACTGCGCTAATCGAAGCGCTGACACACGAAGACAAAGAGCTACAGGGAGAGGATTCGCTGGAGTACGTGCTGGAAGAAAACAATATG
CCCACCCCACAGTCGAGACAGAGTCACGTGCTGGTGGTGGTTCCAGAGGCGGTTGATGGGTCAGTGAGTGAGGCATCCAAGATGGAT
CAACTTGTCGAACAGGTCAA
>PhCRN36_703 PLHAL7033312
AGAATCCGCCGGCTCAAATACAAGATGGTAGAGGTTACCCTGATGTGTGCGGTAGTTCGGGAAGGGGGATTGGACGTCGTCCCTGTG
GATATCGACGAGCAGAAGACAGTAGGCCACCTGAAGGATGCGATTAAGGCGAAGAAGCCTGACACGATCAAGTGTGAAGCTGACCAG
CTGCGGCTGTTCCTGGCTAAGACAGCGGCGGGCGCGTGGCTCGAATCAAGCACAGATGATGTGAAGAAGCTCAAGAAGGGGGAAAAG
ACTGCGCTAATCGAAGCGCTGACACACGAAGACAAAGAGCTACAGGGAGAGGATTCGCTGGAGTACGTGCTGGAAGAAAACAATATG
CCCACCCCACAGTCGAGACAGAGTCACGTGCTGGTGGTGGTTCCAGAGGCGGTTGATCGGTCAGTGAGTGAGGCATCCAAGATGGAT
CAACTTGTCGAACAGGTCAACCAACTGAACGACCAACTGAAAAAA
>PhCRN36_710 PLHAL7103651
AGAATCCGCCGGCTCAAATACAAGATGGTAGAGGTTACCCTGATGTGTGCGGTAGTTCGGGAAGGGGGATTGGACGTCGTCCCTGTG
GATATCGACGAGCAGAAGACAGTAGGCCACCTGAAGGATGCGATTAAGGCGAAGAAGCCTGACACGATCAAGTGTGAAGCTGACCAG
CTGCGGCTGTTCCTGGCTAAGACAGCGGCGGGCGCGTGGCTCGAATCAAGCACAGATGATGTGAAGAAGCTCAAGAAGGGGGAAAAG
ACTGCGCTAATCGAAGCGCTGACACACGAAGACAAAGAGCTACAGGGAGAGGATTCGCTGGAGTACGTGCTGGAAGAAAACAATATG
CCCACCCCACAGTCGAGACAGAGTCACGTGCTGGTGGTGGTTCCAGAGGCGGTTGATCGGTCAGTGAGTGAGGCATCCAAGATGGAT
CAACTTGTCGAACAGGTCAACCAACTGAACGACCAACTGAAAAAA
>PhCRN36_304 PLHAL3043575
AGAATCCGCCGGCTCAAATACAAGATGGTAGAGGTTACCCTGATGTGTGCGGTAGTTCGGGAAGGGGGATTGGACGTCGTCCCTGTG
GATATCGACGAGCAGAAGACAGTAGGCCACCTGAAGGATGCGATTAAGGCGAAGAAGCCTGACACGATCAAGTGTGAAGCTGACCAG
CTGCGGCTGTTCCTGGCTAAGACAGCGGCGGGCGCGTGGCTCGAATCAAGCACAGATGATGTGAAGAAGCTCAAGAAGGGGGAAAAG
ACTGCGCTAATCGAAGCGCTGACACACGAAGACAAAGAGCTACAGGGAGAGGATTCGCTGGAGTACGTGCTGGAAGAAAACAATATG
CCCACCCCACAGTCGAGACAGAGTCACGTGCTGGTGGTGGTTCCAGAGGCGGTTGATCGGTCAGTGAGTGAGGCATCCAAGATGGAT
CAACTTGTCGAACAGGTCAACCAACTGAACGACCAAC
>PhCRN36_700 PLHAL7004416
AGAATCCGCCGGCTCAAATACAAGATGGTAGAGGTTACCCTGATGTGTGCGGTAGTTCGGGAAGGGGGATTGGACGTCGTCCCTGTG
GATATCGACGAGCAGAAGACAGTAGGCCACCTGAAGGATGCGATTAAGGCGAAGAAGCCTGACACGATCAAGTGTGAAGCTGACCAG
CTGCGGCTGTTCCTGGCTAAGACAGCGGCGGGCGCGTGGCTCGAATCAAGCACAGATGATGTGAAGAAGCTCAAGAAGGGGGAAAAG
ACTGCGCTAATCGAAGCGCTGACACACGAAGACAAAGAGCTACAGGGAGAGGATTCGCTGGAGTACGTGCTGGAAGAAAACAATATG
CCCACCCCACAGTCGAGACAGAGTCACGTGCTGGTGGTGGTTCCAGAGGCGGTTGATCGGTCAGTGAGTGAGGCATCCAAGATGGAT
CAACTTGTCGAACAGGTCAACCAACTGAACGAC

>PhCRN36_730 PLHAL7303670
AGAATCCGCCGGCTCAAATACAAGATGGTAGAGGTTACCCTGATGTGTGCGGTAGTTCGGGAAGGGGGATTGGACGTCGTCCCTGTG
GATATCGACGAGCAGAAGACAGTAGGCCACCTGAAGGATGCGATTAAGGCGAAGAAGCCTGACACGATCAAGTGTGAAGCTGACCAG
CTGCGGCTGTTCCTGGCTAAGACAGCGGCGGGCGCGTGGCTCGAATCAAGCACAGATGATGTGAAGAAGCTCAAGAAGGGGGAAAAG
ACTGCGCTAATCGAAGCGCTGACACACGAAGACAAAGAGCTACAGGGAGAGGATTCGCTGGAGTACGTGCTGGAAGAAAACAATATG
CCCACCCCACAGTCGAGACAGAGTCACGTGCTGGTGGTGGTTCCAGAGGCGGTTGATCGGTCAGTGAGTGAGGCATCCAAGATGGAT
CAACTTGTCGAACAGGTCAACCAACTGAACGAC

>PhRXLR01_100 PLHAL100A10123
ATTTCGAAAATCTTCTCTCATCGCTAACCGTTCCCAATGCGTTCGCTGGTTTGGGCTGTCATTGCTACGCTTATCGTTTTAACGCCA
TTCTCTGAAGCAACCTCATCCATTGCATCCAACAATGAAGAATTCAAGCAAAACGTACGAGTTGCATCATCCTCTTTGGAACAAAAG
GGAACAATTGAAGACAGCGTCATTACAAGGAAGTTGCAAAGTGACAGCGTCAAAAAAGGAGACAGCACTGGTCTCGAAGAGCGGGGC
GGGTTACATGTGCCTACAATTCATGACAACAAGATAGTACAAGGTTTTTATAAGGTGATGCGATACCTTCGACAGAAACTGGGTATA
GACTTCTTGCTCACTCGGCTTCGCTATGGGAAGAATGGTTCCCATCAACCCAATATGGGATATTCAAGAGTTGACCACTATCACTAG
CTTTTAATCAACGCGTAATGTCATAATGTATGAACTCTTTGCTACAATCCAAGTCTACTCTTGTAACATTGTCGCTGATAGCTTGCT
CCACAAA
>PhRXLR01_334 PLHAL3340107
ATTTCGAAAATCTTCTCTCATCGCTAACCGTTCCCAATGCGTTCGCTGGTTTGGGCTGTCATTGCTACGCTTATCGTTTTAACGCCA
TTCTCTGAAGCAACCTCATCCATTGCATCCAACAATGAAGAATTCAAGCAAAACGTACGAGTTGCATCATCCTCTTTGGAACAAAAG
GGAACAATTGAAGACAGCGTCATTACAAGGAAGTTGCAAAGTGACAGCGTCAAAAAAGGAGACAGCACTGGTCTCGAAGAGCGGGGC
GGGTTACATGTGCCTACAATTCATGACAACAAGATAGTACAAGGTTTTTATAAGGTGATGCGATACCTTCGACAGAAACTGGGTATA
GACTTCTTGCTCACTCGGCTTCGCTATGGGAAGAATGGTTCCCATCAACCCAATATGGGATATTCAAGAGTTGACCACTATCACTAG
CTTTTAATCAACGCGTAATGCCATAATGTATGAACTCTTTGCTACAATCCAAGTCTACTCTTGTAACATTGTCGCTGATAGCTTGCT
CCACAAA
>PhRXLR01_703 PLHAL7030067
ATTTCGAAAATCTTCTCTCATCGCTAACCGTTCCCAATGCGTTCGCTGGTTTGGGCTGTCATTGCTACGCTTATCGTTTTAACGCCA
TTCTCTGAAGCAACCTCATCCATTGCATCCAACAATGAAGAATTCAAGCAAAACGTACGAGTTGCATCATCCTCTTTGGAACAAAAG
GGAACAATTGAAGACAGCGTCATTACAAGGAAGTTGCAAAGTGACAGCGTCAAAAAAGGAGACAGCACTGGTCTCGAAGAGCGGGGC
GGGTTACATGTGCCTACAATTCATGACAACAAGATAGTACAAGGTTTTTATAAGGTGATGCGATACCTTCGACAGAAACTGGGTATA
GACTTCTTGCTCACTCGGCTTCGCTATGGGAAGAATGGTTCCCATCAACCCAATATGGGATATTCAAGAGTTGACCACTATCACTAG
CTTTTAATCAACGCGTAATGCCATAATGTATGAACTCTTTGCTACAATCCAAGTCTACTCTTGTAACATTGTCGCTGATAGCTTGCT
CCACAAA
>PhRXLR01_710 PLHAL7100126
ATTTCGAAAATCTTCTCTCATCGCTAACCGTTCCCAATGCGTTCGCTGGTTTGGGCTGTCATTGCTACGCTTATCGTTTTAACGCCA
TTCTCTGAAGCAACCTCATCCATTGCATCCAACAATGAAGAATTCAAGCAAAACGTACGAGTTGCATCATCCTCTTTGGAACAAAAG
GGAACAATTGAAGACAGCGTCATTACAAGGAAGTTGCAAAGTGACAGCGTCAAAAAAGGAGACAGCACTGGTCTCGAAGAGCGGGGC
GGGTTACATGTGCCTACAATTCATGACAACAAGATAGTACAAGGTTTTTATAAGGTGATGCGATACCTTCGACAGAAACTGGGTATA
GACTTCTTGCTCACTCGGCTTCGCTATGGGAAGAATGGTTCCCATCAACCCAATATGGGATATTCAAGAGTTGACCACTATCACTAG
CTTTTAATCAACGCGTAATGCCATAATGTATGAACTCTTTGCTACAATCCAAGTCTACTCTTGTAACATTGTCGCTGATAGCTTGCT
CCACAAA
>PhRXLR01_304 PLHAL3040117
ATTTCGAAAATCTTCTCTCATCGCTAACCGTTCCCAATGCGTTCGCTGGTTTGGGCTGTCATTGCTACGCTTATCGTTTTAACGCCA
TTCTCTGAAGCAACCTCATCCATTGCATCCAACAATGAAGAATTCAAGCAAAACGTACGAGTTGCATCATCCTCTTTGGAACAAAAG
GGAACAATTGAAGACAGCGTCATTACAAGGAAGTTGCAAAGTGACAGCGTCAAAAAAGGAGACAGCACTGGTCTCGAAGAGCGGGGC
GGGTTACATGTGCCTACAATTCATGACAACAAGATAGTACAAGGTTTTTATAAGGTGATGCGATACCTTCGACAGAAACTGGGTATA
GACTTCTTGCTCACTCGGCTTCGCTATGGGAAGAATGGTTCCCATCAACCCAATATGGGATATTCAAGAGTTGACCACTATCACTAG
CTTTTAATCAACGCGTAATGTCATAATGTATGAACTCTTTGCTACAATCCAAGTCTACTCTTGTAACATTGTCGCTGATAGCTTGCT
CCACAAA
>PhRXLR01_700 PLHAL7000082
ATTTCGAAAATCTTCTCTCATCGCTAACCGTTCCCAATGCGTTCGCTGGTTTGGGCTGTCATTGCTACGCTTATCGTTTTAACGCCA
TTCTCTGAAGCAACCTCATCCATTGCATCCAACAATGAAGAATTCAAGCAAAACGTACGAGTTGCATCATCCTCTTTGGAACAAAAG
GGAACAATTGAAGACAGCGTCATTACAAGGAAGTTGCAAAGTGACAGCGTCAAAAAAGGAGACAGCACTGGTCTCGAAGAGCGGGGC
GGGTTACATGTGCCTACAATTCATGACAACAAGATAGTACAAGGTTTTTATAAGGTGATGCGATACCTTCGACAGAAACTGGGTATA
GACTTCTTGCTCACTCGGCTTCGCTATGGGAAGAATGGTTCCCATCAACCCAATATGGGATATTCAAGAGTTGACCACTATCACTAG
CTTTTAATCAACGCGTAATGCCATAATGTATGAACTCTTTGCTACAATCCAAGTCTACTCTTGTAACATTGTCGCTGATAGCTTGCT
CCACAAA
>PhRXLR01_730 PLHAL7300131
ATTTCGAAAATCTTCTCTCATCGCTAACCGTTCCCAATGCGTTCGCTGGTTTGGGCTGTCATTGCTACGCTTATCGTTTTAACGCCA
TTCTCTGAAGCAACCTCATCCATTGCATCCAACAATGAAGAATTCAAGCAAAACGTACGAGTTGCATCATCCTCTTTGGAACAAAAG
GGAACAATTGAAGACAGCGTCATTACAAGGAAGTTGCAAAGTGACAGCGTCAAAAAAGGAGACAGCACTGGTCTCGAAGAGCGGGGC
GGGTTACATGTGCCTACAATTCATGACAACAAGATAGTACAAGGTTTTTATAAGGTGATGCGATACCTTCGACAGAAACTGGGTATA
GACTTCTTGCTCACTCGGCTTCGCTATGGGAAGAATGGTTCCCATCAACCCAATATGGGATATTCAAGAGTTGACCACTATCACTAG
CTTTTAATCAACGCGTAATGCCATAATGTATGAACTCTTTGCTACAATCCAAGTCTACTCTTGTAACATTGTCGCTGATAGCTTGCT
CCACAAA

>PhRXLR02.1_100 PLHAL100A10020

ATTCTTCAATTTGTCTCTTGTACCACAGCTCACTAGAAACCACATCCAGCTTGTTTTCGGCATGCGTATCTCCCCCGTCGTGCTCGC
GTTGGCAGCTTTCGTGATTCCAAGTGGTGCAGTCTCATCGTCTATCACGAATAACGATGGTACAAGATCTCTGCGTTCAAGTAACCC
TGAGCCAGCCGCTATAGCAGACCTGGTGCCCGCGTTGCAAGGCTCTGACAGTACAAAGAGGCTTCTCATCTCCAACGATGATTTTGA
TTACGATGATCATGAAGAAAGGAAATCGTGGAAGAAGCGACAAAAAAAGATGGCGAAGTATCACAAAAAGTATATGAAGCAATACAT
GAAGCAATATATGAAGCCCTGGAAAAAGAGTAAACATTCGCATCGGTATTGGTATTGAGTATTTAGGCAGTAAGTTGAAGCTTAGTT
GGTTAGCGTACTGGTCAGAACATTGTTGTTCTTAGCAGGTATTGCTTTCTCATTTATACACTCTAACACCCTCATAGCTTCTTCACT
GATAATTATTCAATTTCCTTCTTAATCCTC
>PhRXLR02.1_334 PLHAL3340020
ATTCTTCAATTTGTCTCTTGTACCACAGCTCACTAGAAACCACATCCAGCTTGTTTTCGGCATGCGTATCTCCCCCGTCGTGCTCGC
GTTGGCAGCTTTCGTGATTCCAAGTGGTGCAGTCTCATCGTCTATCACGAATAACGATGGTACAAGATCTCTGCGTTCAAGTAACCC
TGAGCCAGCCGCTATAGCAGACCTGGTGCCCGCGTTGCAAGGCTCTGACAGTACAAAGAGGCTTCTCATCTCCAACGATGATTTTGA
TTACGATGATCATGAAGAAAGGAAATCGTGGAAGAAGCGACATAAAAAGATGGCGAAGTATCACAAAAAGTATATGAAGCAATACAT
GAAGCAATATATGAAGCCCTGGAAAAAGAGTAAATATTCGCATCGGTATTGGTATTGAGTATTTAGGCAGTAAGTTGAAGCTTAGTT
GGTTAGCGTACTGGTCAGAACATTGTTGTTCTTAGCAGGTATTGCTTTCTCATTTATACACTCTAACACCCTCATAGCTTCTTCACT
GATAATTATTCAATTTCCTTCTTAATCCTC
>PhRXLR02.1_703 PLHAL7030020
ATTCTTCAATTTGTCTCTTGTACCACAGCTCACTAGAAACCACATCCAGCTTGTTTTCGGCATGCGTATCTCCCCCGTCGTGCTCGC
GTTGGCAGCTTTCGTGATTCCAAGTGGTGCAGTCTCATCGTCTATCACGAATAACGATGGTACAAGATCTCTGCGTTCAAGTAACCC
TGAGCCAGCCGCTATAGCAGACCTGGTGCCCGCGTTGCAAGGCTCTGACAGTACAAAGAGGCTTCTCATCTCCAACGATGATTTTGA
TTACGATGATCATGAAGAAAGGAAATCGTGGAAGAAGCGACATAAAAAGATGGCGAAGTATCACAAAAAGTATATGAAGCAATACAT
GAAGCAATATATGAAGCCCTGGAAAAAGAGTAAATATTCGCATCGGTATTGGTATTGAGTATTTAGGCAGTAAGTTGAAGCTTAGTT
GGTTAGCGTACTGGTCAGAACATTGTTGTTCTTAGCAGGTATTGCTTTCTCATTTATACACTCTAACACCCTCATAGCTTCTTCACT
GATAATTATTCAATTTCCTTCTTAATCCTC
>PhRXLR02.1_710 PLHAL7100062
ATTCTTCAATTTGTCTCTTGTACCACAGCTCACTAGAAACCACATCCAGCTTGTTTTCGGCATGCGTATCTCCCCCGTCGTGCTCGC
GTTGGCAGCTTTCGTGATTCCAAGTGGTGCAGTCTCATCGTCTATCACGAATAACGATGGTACAAGATCTCTGCGTTCAAGTAACCC
TGAGCCAGCCGCTATAGCAGACCTGGTGCCCGCGTTGCAAGGCTCTGACAGTACAAAGAGGCTTCTCATCTCCAACGATGATTTTGA
TTACGATGATCATGAAGAAAGGAAATCGTGGAAGAAGCGACATAAAAAGATGGCGAAGTATCACAAAAAGTATATGAAGCAATACAT
GAAGCAATATATGAAGCCCTGGAAAAAGAGTAAATATTCGCATCGGTATTGGTATTGAGTATTTAGGCAGTAAGTTGAAGCTTAGTT
GGTTAGCGTACTGGTCAGAACATTGTTGTTCTTAGCAGGTATTGCTTTCTCATTTATACACTCTAACACCCTCATAGCTTCTTCACT
GATAATTATTCAATTTCCTTCTTAATCCTC
>PhRXLR02.1_304 PLHAL3040022
ATTCTTCAATTTGTCTCTTGTACCACAGCTCACTAGAAACCACATCCAGCTTGTTTTCGGCATGCGTATCTCCCCCGTCGTGCTCGC
GTTGGCAGCTTTCGTGATTCCAAGTGGTGCAGTCTCATCGTCTATCACGAATAACGATGGTACAAGATCTCTGCGTTCAAGTAACCC
TGAGCCAGCCGCTATAGCAGACCTGGTGCCCGCGTTGCAAGGCTCTGACAGTACAAAGAGGCTTCTCATCTCCAACGATGATTTTGA
TTACGATGATCATGAAGAAAGGAAATCGTGGAAGAAGCGACAAAAAAAGATGGCGAAGTATCACAAAAAGTATATGAAGCAATACAT
GAAGCAATATATGAAGCCCTGGAAAAAGAGTAAACATTCGCATCGGTATTGGTATTGAGTATTTAGGCAGTAAGTTGAAGCTTAGTT
GGTTAGCGTACTGGTCAGAACATTGTTGTTCTTAGCAGGTATTGCTTTCTCATTTATACACTCTAACACCCTCATAGCTTCTTCACT
GATAATTATTCAATTTCCTTCTTAATCCTC
>PhRXLR02.1_700 PLHAL7000053
ATTCTTCAATTTGTCTCTTGTACCACAGCTCACTAGAAACCACATCCAGCTTGTTTTCGGCATGCGTATCTCCCCCGTCGTGCTCGC
GTTGGCAGCTTTCGTGATTCCAAGTGGTGCAGTCTCATCGTCTATCACGAATAACGATGGTACAAGATCTCTGCGTTCAAGTAACCC
TGAGCCAGCCGCTATAGCAGACCTGGTGCCCGCGTTGCAAGGCTCTGACAGTACAAAGAGGCTTCTCATCTCCAACGATGATTTTGA
TTACGATGATCATGAAGAAAGGAAATCGTGGAAGAAGCGACATAAAAAGATGGCGAAGTATCACAAAAAGTATATGAAGCAATACAT
GAAGCAATATATGAAGCCCTGGAAAAAGAGTAAATATTCGCATCGGTATTGGTATTGAGTATTTAGGCAGTAAGTTGAAGCTTAGTT
GGTTAGCGTACTGGTCAGAACATTGTTGTTCTTAGCAGGTATTGCTTTCTCATTTATACACTCTAACACCCTCATAGCTTCTTCACT
GATAATTATTCAATTTCCTTCTTAATCCTC
>PhRXLR02.1_730 PLHAL7300003
ATTCTTCAATTTGTCTCTTGTACCACAGCTCACTAGAAACCACATCCAGCTTGTTTTCGGCATGCGTATCTCCCCCGTCGTGCTCGC
GTTGGCAGCTTTCGTGATTCCAAGTGGTGCAGTCTCATCGTCTATCACGAATAACGATGGTACAAGATCTCTGCGTTCAAGTAACCC
TGAGCCAGCCGCTATAGCAGACCTGGTGCCCGCGTTGCAAGGCTCTGACAGTACAAAGAGGCTTCTCATCTCCAACGATGATTTTGA
TTACGATGATCATGAAGAAAGGAAATCGTGGAAGAAGCGACATAAAAAGATGGCGAAGTATCACAAAAAGTATATGAAGCAATACAT
GAAGCAATATATGAAGCCCTGGAAAAAGAGTAAATATTCGCATCGGTATTGGTATTGAGTATTTAGGCAGTAAGTTGAAGCTTAGTT
GGTTAGCGTACTGGTCAGAACATTGTTGTTCTTAGCAGGTATTGCTTTCTCATTTATACACTCTAACACCCTCATAGCTTCTTCACT
GATAATTATTCAATTTCCTTCTTAATCCTC

>PhRXLR02.2_100 PLHAL100A10020
TCGGATCAGATATATCTGGTCGATGCGATCAGAATTAGATCCCTGTCGAAGCTGAAACATTGGAAGCAAGCCTGGTTAAACAAGGCC
ACTATTCAATTTGTCTCCTCTTCCACAGCTAGCTACCTACCTACAACCAACGTGTTTCCGGCATGCGTATCTCCCCAATCGTGCTCG
CGTTGGCAGCCTTCGTGATTCCAAGTGGTGCAGTCTCATCGTCTATCACGAATAACGATGGTACAAGATCTCTGCGTACAAGTAACC
CTGAGCCAGCTGCTATAGCAGACCTGATGCCCGCATTGCAAGGCTCCGACAGTACTAAGAGGCTTCTCATCTCCAACGATGATTTTG
ATTACGATGGTCATGAAGAGAGGAAATCGAAGAAGAAGCAAAAAAAAAAGCTAAAAAGATGGCGAAGCGTTTTAAAAAGTCGTTAAA
GAAGTTTTGGAACTAGTAGATATT
>PhRXLR02.2_334 PLHAL3340020
TCGGATCAGATATATCTGGTCGATGCGATCAGAATTAGATCCCTGTCGAAGCTGAAACATTGGAAGCAAGCCTGGTTAAACAAGGCC
ACTATTCAATTTGTCTCCTCTTCCACAGCTAGCTACCTACCTACAACCAACGTGTTTCCGGCATGCGTATCTCCCCAATCGTGCTCG
CGTTGGCAGCCTTCGTGATTCCAAGTGGTGCAGTCTCATCGTCTATCACGAATAACGATGGTACAAGATCTCTGCGTACAAGTAACC

CTGAGCCAGCTGCTATAGCAGACCTGATGCCCGCATTGCAAGGCTCCGACAGTACTAAGAGGCTTCTCATCTCCAACGATGATTTTG
ATTACGATGGTCATGAAGAGAGGAAATCGAAGAAGAAGCAAAAAAAAAAAGCTAAAAAGATGGCGAAGCGTTTTAAAAAGTCGTTAA
AGAAGTTTTGGAACTAGTAGATATTCGTATTGGCATTGATTGATGAGTGTTTAGGCTATAAGTTGAAGCTTGGGTGGTAAGCGTCAC
TGGTAAAACCATTGTTGTTCTTGGCAGGTATTGCTTCAAGACCCTTGCAGCTTCTTCACTAATAATTAATTAATGTCCGTTTTCTTA
ATCCT
>PhRXLR02.2_703 PLHAL7030020
TCGGATCAGATATATCTGGTCGATGCGATCAGAATTAGATCCCTGTCGAAGCTGAAACATTGGAAGCAAGCCTGGTTAAACAAGGCC
ACTATTCAATTTGTCTCCTCTTCCACAGCTAGCTACCTACCTACAACCAACGTGTTTCCGGCATGCGTATCTCCCCAATCGTGCTCG
CGTTGGCAGCCTTCGTGATTCCAAGTGGTGCAGTCTCATCGTCTATCACGAATAACGATGGTACAAGATCTCTGCGTACAAGTAACC
CTGAGCCAGCTGCTATAGCAGACCTGATGCCCGCATTGCAAGGCTCCGACAGTACTAAGAGGCTTCTCATCTCCAACGATGATTTTG
ATTACGATGGTCATGAAGAGAGGAAATCGAAGAAGAAGCAAAAAAAAAAAGCTAAAAAGATGGCGAAGCGTTTTAAAAAGTCGTTAA
AGAAGTTTTGGAACTAGTAGATATTCGTATTGGCATTGATTGATGAGTGTTTAGGCTATAAGTTGAAGCTTGGGTGGTAAGCGTCAC
TGGTAAAACCATTGTTGTTCTTGGCAGGTATTGCTTCAAGACCCTTGCAGCTTCTTCACTAATAATTAATTAATGTCCGTTTTCTTA
ATCCT
>PhRXLR02.2_710 PLHAL7100062
TCGGATCAGATATATCTGGTCGATGCGATCAGAATTAGATCCCTGTCGAAGCTGAAACATTGGAAGCAAGCCTGGTTAAACAAGGCC
ACTATTCAATTTGTCTCCTCTTCCACAGCTAGCTACCTACCTACAACCAACGTGTTTCCGGCATGCGTATCTCCCCAATCGTGCTCG
CGTTGGCAGCCTTCGTGATTCCAAGTGGTGCAGTCTCATCGTCTATCACGAATAACGATGGTACAAGATCTCTGCGTACAAGTAACC
CTGAGCCAGCTGCTATAGCAGACCTGATGCCCGCATTGCAAGGCTCCGACAGTACTAAGAGGCTTCTCATCTCCAACGATGATTTTG
ATTACGATGGTCATGAAGAGAGGAAATCGAAGAAGAAGCAAAAAAAAAAAGCTAAAAAGATGGCGAAGCGTTTTAAAAAGTCGTTAA
AGAAGTTTTGGAACTAGTAGATATTCGTATTGGCATTGATTGATGAGTGTTTAGGCTATAAGTTGAAGCTTGGGTGGTAAGCGTCAC
TGGTAAAACCATTGTTGTTCTTGGCAGGTATTGCTTCAAGACCCTTGCAGCTTCTTCACTAATAATTAATTAATGTCCGTTTTCTTA
ATCCT
>PhRXLR02.2_304 PLHAL3040022
TCGGATCAGATATATCTGGTCGATGCGATCAGAATTAGATCCCTGTCGAAGCTGAAACATTGGAAGCAAGCCTGGTTAAACAAGGCC
ACTATTCAATTTGTCTCCTCTTCCACAGCTAGCTACCTACCTACAACCAACGTGTTTCCGGCATGCGTATCTCCCCAATCGTGCTCG
CGTTGGCAGCCTTCGTGATTCCAAGTGGTGCAGTCTCATCGTCTATCACGAATAACGATGGTACAAGATCTCTGCGTACAAGTAACC
CTGAGCCAGCTGCTATAGCAGACCTGATGCCCGCATTGCAAGGCTCCGACAGTACTAAGAGGCTTCTCATCTCCAACGATGATTTTG
ATTACGATGGTCATGAAGAGAGGAAATCGAAGAAGAAGCAAAAAAAAAAAGCTAAAAAGATGGCGAAGCGTTTTAAAAAGTCGTTAA
AGAAGTTTTGGAACTAGTAGATATTCGTATTGGCATTGATTGATGAGTGTTTAGGCTATAAGTTGAAGCTTGGGTGGTAAGCGTCAC
TGGTAAAACCATTGTTGTTCTTGGCAGGTATTGCTTCAAGACCCTTGCAGCTTCTTCACTAATAATTAATTAATGTCCGTTTTCTTA
ATCCT
>PhRXLR02.2_700 PLHAL7000053
TCGGATCAGATATATCTGGTCGATGCGATCAGAATTAGATCCCTGTCGAAGCTGAAACATTGGAAGCAAGCCTGGTTAAACAAGGCC
ACTATTCAATTTGTCTCCTCTTCCACAGCTAGCTACCTACCTACAACCAACGTGTTTCCGGCATGCGTATCTCCCCAATCGTGCTCG
CGTTGGCAGCCTTCGTGATTCCAAGTGGTGCAGTCTCATCGTCTATCACGAATAACGATGGTACAAGATCTCTGCGTACAAGTAACC
CTGAGCCAGCTGCTATAGCAGACCTGATGCCCGCATTGCAAGGCTCCGACAGTACTAAGAGGCTTCTCATCTCCAACGATGATTTTG
ATTACGATGGTCATGAAGAGAGGAAATCGAAGAAGAAGCAAAAAAAAAAAGCTAAAAAGATGGCGAAGCGTTTTAAAAAGTCGTTAA
AGAAGTTTTGGAACTAGTAGATATTCGTATTGGCATTGATTGATGAGTGTTTAGGCTATAAGTTGAAGCTTGGGTGGTAAGCGTCAC
TGGTAAAACCATTGTTGTTCTTGGCAGGTATTGCTTCAAGACCCTTGCAGCTTCTTCACTAATAATTAATTAATGTCCGTTTTCTTA
ATCCT
>PhRXLR02.2_730 PLHAL7300003
TCGGATCAGATATATCTGGTCGATGCGATCAGAATTAGATCCCTGTCGAAGCTGAAACATTGGAAGCAAGCCTGGTTAAACAAGGCC
ACTATTCAATTTGTCTCCTCTTCCACAGCTAGCTACCTACCTACAACCAACGTGTTTCCGGCATGCGTATCTCCCCAATCGTGCTCG
CGTTGGCAGCCTTCGTGATTCCAAGTGGTGCAGTCTCATCGTCTATCACGAATAACGATGGTACAAGATCTCTGCGTACAAGTAACC
CTGAGCCAGCTGCTATAGCAGACCTGATGCCCGCATTGCAAGGCTCCGACAGTACTAAGAGGCTTCTCATCTCCAACGATGATTTTG
ATTACGATGGTCATGAAGAGAGGAAATCGAAGAAGAAGCAAAAAAAAAAAGCTAAAAAGATGGCGAAGCGTTTTAAAAAGTCGTTAA
AGAAGTTTTGGAACTAGTAGATATTCGTATTGGCATTGATTGATGAGTGTTTAGGCTATAAGTTGAAGCTTGGGTGGTAAGCGTCAC
TGGTAAAACCATTGTTGTTCTTGGCAGGTATTGCTTCAAGACCCTTGCAGCTTCTTCACTAATAATTAATTAATGTCCGTTTTCTTA
ATCCT

>PhRXLR04_100 PLHAL100A13730
GTCAGCAGGCAACAAGCTTTTTATCAAATTGCATGCTGACAAGGCGAAGGACCTCTTCAGCAGTCGTGAGTTTGGAAAGTGGTCGAA
ATACTTTACGGATGCCTTCCCTGATAATCCAGAAAAGGCCGCTGGTTATATGGTGTCGATATTGGCAACAAAACACGAGGTCCAATT
GGTCAATTCCATTCCGAAAACCGAGGTTGTCAATGACAAATTAGCTTTTGGTGATGAATTCGAAGCTGCGCTGATAAATTATCTCCG
TGGTAATGAGAAGTCTGAGACGTTTAAAGATAGTCTTAAAACGTCACTGCTTGCTCTCAGTGGTAGACTGCAGAAGGACGAGAGTTC
AGAGCTCATTGGTGTGAAGATGCAAGAACAAATCATGTCTGTTTCCAGCGGCAACGAGGAGAAACTGGTTGATGCTGTTAATGTACA
ACCAGTGCACAAGTTGTCCTCACTCCAGGAGGTGGTCAGCGAGCCAGTTACGTTGGTGACTCCCAGACTAAGCACCCAAGAGGAAAA
AGCGGCATATTTATTGAATGAAGCCGGACTTGACAAAGTGGTGAAAGATATCTTCAACAGCAAACAGTTCAAAGTGTGGTCGGAACA
AATG
>PhRXLR04_334 PLHAL3343777
GTCAGCAGGCAACAAGCTTTTTATCAAATTGCATGCTGACAAGGCGAAGGACCTCTTCAGCAGTCGTGAGTTTGGAAAGTGGTCGAA
ATACTTTACGGATGCCTTCCCTGATAATCCAGAAAAGGCCGCTGGTTATATGGTGTCGATATTGGCAACAAAACACGAGGTCCAATT
GGTCAATTCCATTCCGAAAACCGAGGTTGTCAATGACAAATTAGCTTTTGGTGATGAATTCGAAGCTGCGCTGATAAATTATCTCCG
TGGTAATGAGAAGTCTGAGACGTTTAAAGATAGTCTTAAAACGTCACTGCTTGCTCTCAGTGGTAGACTGCAGAAGGACGAGAGTTC
AGAGCTCATTGGTGTGAAGATGCAAGAACAAATCATGTCTGTTTCCAGCGGCAACGAGGAGAAACTGGTTGATGCTGTTAATGTACA
ACCAGTGCACAAGTTGTCCTCACTCCAGGAGGTGGTCAGCGAGCCAGTTACGTTGGTGACTCCCAGACTAAGCACACAAGAGGAAAA

AGCGGCATATTTATTGAAAGAAGCCGGACTTGACAAAGTGGTGAAAGATATCTTCAACAGCAAACAGTTCAAAGTGTGGTCGGAACA
AATG
>PhRXLR04_703 PLHAL7034915
GTCAGCAGGCAACAAGCTTTTTATCAAATTGCATGCTGACAAGGCGAAGGACCTCTTCAGCAGTCGTGAGTTTGGAAAGTGGTCGAA
ATACTTTACGGATGCCTTCCCTGATAATCCAGAAAAGGCCGCTGGTTATATGGTGTCGATATTGGCAACAAAACACGAGGTCCAATT
GGTCAATTCCATTCCGAAAACCGAGGTTGTCAATGACAAATTAGCTTTTGGTGATGAATTCGAAGCTGCGCTGATAAATTATCTCCG
TGGTAATGAGAAGTCTGAGACGTTTAAAGATAGTCTTAAAACGTCACTGCTTGCTCTCAGTGGTAGACTGCAGAAGGACGAGAGTTC
AGAGCTCATTGGTGTGAAGATGCAAGAACAAATCATGTCTGTTTCCAGCGGCAACGAGGAGAAACTGGTTGATGCTGTTAATGTACA
ACCAGTGCACAAGTTGTCCTCACTCCAGGAGGTGGTCAGCGAGCCAGTTACGTTGGTGACTCCCAGACTAAGCAC
>PhRXLR04_710 PLHAL7103657
GTCAGCAGGCAACAAGCTTTTTATCAAATTGCATGCTGACAAGGCGAAGGACCTCTTCAGCAGTCGTGAGTTTGGAAAGTGGTCGAA
ATACTTTACGGATGCCTTCCCTGATAATCCAGAAAAGGCCGCTGGTTATATGGTGTCGATATTGGCAACAAAACACGAGGTCCAATT
GGTCAATTCCATTCCGAAAACCGAGGTTGTCAATGACAAATTAGCTTTTGGTGATGAATTCGAAGCTGCGCTGATAAATTATCTCCG
TGGTAATGAGAAGTCTGAGACGTTTAAAGATAGTCTTAAAACGTCACTGCTTGCTCTCAGTGGTAGACTGCAGAAGGACGAGAGTTC
AGAGCTCATTGGTGTGAAGATGCAAGAACAAATCATGTCTGTTTCCAGCGGCAACGAGGAGAAACTGGTTGATGCTGTTAATGTACA
ACCAGTGCACAAGTTGTCCTCACTCCAGGAGGTGGTCAGCGAGCCAGTTACGTTGGTGACTCCCAGACTAAGCACACAAGAGGAAAA
AGCGGCATATTTATTGAAAGAAGCCGGACTTGACAAAGTGGTGAAAGATATCTTCAACAGCAAACAGTTCAAAGTGTGGTCGGAACA
AATG
>PhRXLR04_304 PLHAL3043670
GTCAGCAGGCAACAAGCTTTTTATCAAATTGCATGCTGACAAGGCGAAGGACCTCTTCAGCAGTCGTGAGTTTGGAAAGTGGTCGAA
ATACTTTACGGATGCCTTCCCTGATAATCCAGAAAAGGCCGCTGGTTATATGGTGTCGATATTGGCAACAAAACACGAGGTCCAATT
GGTCAATTCCATTCCGAAAACCGAGGTTGTCAATGACAAATTAGCTTTTGGTGATGAATTCGAAGCTGCGCTGATAAATTATCTCCG
TGGTAATGAGAAGTCTGAGACGTTTAAAGATAGTCTTAAAACGTCACTGCTTGCTCTCAGTGGTAGACTGCAGAAGGACGAGAGTTC
AGAGCTCATTGGTGTGAAGATGCAAGAACAAATCATGTCTGTTTCCAGCGGCAACGAGGAGAAACTGGTTGATGCTGTTAATGTACA
ACCAGTGCACAAGTTGTCCTCACTCCAGGAGGTGGTCAGCGAGCCAGTTACGTTGGTGACTCCCAGACTAAGCACACAAGAGGAAAA
AGCGGCATATTTATTGAAAGAAGCCGGACTTGACAAAGTGGTGAAAGATATCTTCAACAGCAAACAGTTCAAAGTGTGGTCGGAACA
AATG
>PhRXLR04_700 PLHAL7004535
GTCAGCAGGCAACAAGCTTTTTATCAAATTGCATGCTGACAAGGCGAAGGACCTCTTCAGCAGTCGTGAGTTTGGAAAGTGGTCGAA
ATACTTTACGGATGCCTTCCCTGATAATCCAGAAAAGGCCGCTGGTTATATGGTGTCGATATTGGCAACAAAACACGAGGTCCAATT
GGTCAATTCCATTCCGAAAACCGAGGTTGTCAATGACAAATTAGCTTTTGGTGATGAATTCGAAGCTGCGCTGATAAATTATCTCCG
TGGTAATGAGAAGTCTGAGACGTTTAAAGATAGTCTTAAAACGTCACTGCTTGCTCTCAGTGGTAGACTGCAGAAGGACGAGAGTTC
AGAGCTCATTGGTGTGAAGATGCAAGAACAAATCATGTCTGTTTCCAGCGGCAACGAGGAGAAACTGGTTGATGCTGTTAATGTACA
ACCAGTGCACAAGTTGTCCTCACTCCAGGAGGTGGTCAGCGAGCCAGTTACGTTGGTGACTCCCAGACTAAGCACACAAGAGGAAAA
AGCGGCATATTTATTGAAAGAAGCCGGACTTGACAAAGTGGTGAAAGATATCTTCAACAGCAAACAGTTCAAAGTGTGGTCGGAACA
AATG
>PhRXLR04_730 PLHAL7303780
GTCAGCAGGCAACAAGCTTTTTATCAAATTGCATGCTGACAAGGCGAAGGACCTCTTCAGCAGTCGTGAGTTTGGAAAGTGGTCGAA
ATACTTTACGGATGCCTTCCCTGATAATCCAGAAAAGGCCGCTGGTTATATGGTGTCGATATTGGCAACAAAACACGAGGTCCAATT
GGTCAATTCCATTCCGAAAACCGAGGTTGTCAATGACAAATTAGCTTTTGGTGATGAATTCGAAGCTGCGCTGATAAATTATCTCCG
TGGTAATGAGAAGTCTGAGACGTTTAAAGATAGTCTTAAAACGTCACTGCTTGCTCTCAGTGGTAGACTGCAGAAGGACGAGAGTTC
AGAGCTCATTGGTGTGAAGATGCAAGAACAAATCATGTCTGTTTCCAGCGGCAACGAGGAGAAACTGGTTGATGCTGTTAATGTACA
ACCAGTGCACAAGTTGTCCTCACTCCAGGAGGTGGTCAGCGAGCCAGTTACGTTGGTGACTCCCAGACTAAGCACCCAAGAGGAAAA
AGCGGCATATTTATTGAATGAAGCCGGACTTGACAAAGTGGTGAAAGATATCTTCAACAGCAAACAGTTCAAAGTGTGGTCGGAACA
AATG

>PhRXLR05_100 PLHAL100A10020
TCCATAACTGTTAACTATTCGAAATGCGCTTCTGCCTTGTCTTTATTAGGCTCGCAGCGTTCGTGATCTTAAGCGGTGGTGCCACAT
CGACAACCACGGACAACGATGACACAAGATTGCTGCAGACGAGTAATATTGAGACAGCTGCTGTGGCAAATGTGCTACACGTAATGC
AGAGCTCCGAGAGTAGTAAGAGGCTCCTTAGGCTGAACGACCAAGCGGATATCAGTGGTCATGACGAAGAAAGAAGCTCGCTGATTG
AGAAGGGATGGAAAAAACTTCGAAAGCTCATCAAGAAGGTGTGGAAATACGTTAAGAAGCCTTTTAAGAAGACCGCGAAGATTATCA
AAAAGCCTTTTAAGAGTCGCACGAAGAACATTCATATAGTGTACTACAAAAGCCGTTTTTGATCTATAACAGTATACATGACTAG
>PhRXLR05_334 PLHAL3340020
TCCATAACTGTTAACTATTCGAAATGCGCTTCTGCCTTGTCTTTATTAGGCTCGCAGCGTTCGTGATCTTAAGCGGTGGTGCCACAT
CGACAACCACGGACAACGATGACACAAGATTGCTGCAGACGAGTAATATTGAGACAGCTGCTGTGGCAAATGTGCTACACGTAATGC
AGAGCTCCGAGAGTAGTAAGAGGCTCCTTAGGCTGAACGACCAAGCGGATATCAGTGGTCATGACGAAGAAAGAAGCTCGCTGATTG
AGAAGGGATGGAAAAAACTTCGAAAGCTCATCAAGAAGGTGTGGAAATACGTTAAGAAGCCTTTTAAGAAGACCGCGAAGATTATCA
AAAAGCCTTTTAAGAGTCGCACGAAGAACATTCATATAGTGTACTACAAAAGCCGTTTTTGATCTATAACAGTATACATGACTAG
>PhRXLR05_703 PLHAL7030020
TCCATAACTGTTAACTATTCGAAATGCGCTTCTGCCTTGTCTTTATTAGGCTCGCAGCGTTCGTGATCTTAAGCGGTGGTGCCACAT
CGACAACCACGGACAACGATGACACAAGATTGCTGCAGACGAGTAATATTGAGACAGCTGCTGTGGCAAATGTGCTACACGTAATGC
AGAGCTCCGAGAGTAGTAAGAGGCTCCTTAGGCTGAACGACCAAGCGGATATCAGTGGTCATGACGAAGAAAGAAGCTCGCTGATTG
AGAAGGGATGGAAAAAACTTCGAAAGCTCATCAAGAAGGTGTGGAAATACGTTAAGAAGCCTTTTAAGAAGACCGCGAAGATTATCA
AAAAGCCTTTTAAGAGTCGCACGAAGAACATTCATATAGTGTACTACAAAAGCCGTTTTTGATCTATAACAGTATACATGACTAG
>PhRXLR05_710 PLHAL7100062
TCCATAACTGTTAACTATTCGAAATGCGCTTCTGCCTTGTCTTTATTAGGCTCGCAGCGTTCGTGATCTTAAGCGGTGGTGCCACAT
CGACAACCACGGACAACGATGACACAAGATTGCTGCAGACGAGTAATATTGAGACAGCTGCTGTGGCAAATGTGCTACACGTAATGC

AGAGCTCCGAGAGTAGTAAGAGGCTCCTTAGGCTGAACGACCAAGCGGATATCAGTGGTCATGACGAAGAAAGAAGCTCGCTGATTG
AGAAGGGATGGAAAAAACTTCGAAAGCTCATCAAGAAGGTGTGGAAATACGTTAAGAAGCCTTTTAAGAAGACCGCGAAGATTATCA
AAAAGCCTTTTAAGAGTCGCACGAAGAACATTCATATAGTGTACTACAAAAGCCGTTTTTGATCTATAACAGTATACATGACTAG
>PhRXLR05_304 PLHAL3040022
TCCATAACTGTTAACTATTCGAAATGCGCTTCTGCCTTGTCTTTATTAGGCTCGCAGCGTTCGTGATCTTAAGCGGTGGTGCCACAT
CGACAACCACGGACAACGATGACACAAGATTGCTGCAGACGAGTAATATTGAGACAGCTGCTGTGGCAAATGTGCTACACGTAATGC
AGAGCTCCGAGAGTAGTAAGAGGCTCCTTAGGCTGAACGACCAAGCGGATATCAGTGGTCATGACGAAGAAAGAAGCTCGCTGATTG
AGAAGGGATGGAAAAAACTTCGAAAGCTCATCAAGAAGGTGTGGAAATACGTTAAGAAGCCTTTTAAGAAGACCGCGAAGATTATCA
AAAAGCCTTTTAAGAGTCGCACGAAGAACATTCATATAGTGTACTACAAAAGCCGTTTTTGATCTATAACAGTATACATGACTAG
>PhRXLR05_700 PLHAL7000053
TCCATAACTGTTAACTATTCGAAATGCGCTTCTGCCTTGTCTTTATTAGGCTCGCAGCGTTCGTGATCTTAAGCGGTGGTGCCACAT
CGACAACCACGGACAACGATGACACAAGATTGCTGCAGACGAGTAATATTGAGACAGCTGCTGTGGCAAATGTGCTACACGTAATGC
AGAGCTCCGAGAGTAGTAAGAGGCTCCTTAGGCTGAACGACCAAGCGGATATCAGTGGTCATGACGAAGAAAGAAGCTCGCTGATTG
AGAAGGGATGGAAAAAACTTCGAAAGCTCATCAAGAAGGTGTGGAAATACGTTAAGAAGCCTTTTAAGAAGACCGCGAAGATTATCA
AAAAGCCTTTTAAGAGTCGCACGAAGAACATTCATATAGTGTACTACAAAAGCCGTTTTTGATCTATAACAGTATACATGACTAG
>PhRXLR05_730 PLHAL7300003
TCCATAACTGTTAACTATTCGAAATGCGCTTCTGCCTTGTCTTTATTAGGCTCGCAGCGTTCGTGATCTTAAGCGGTGGTGCCACAT
CGACAACCACGGACAACGATGACACAAGATTGCTGCAGACGAGTAATATTGAGACAGCTGCTGTGGCAAATGTGCTACACGTAATGC
AGAGCTCCGAGAGTAGTAAGAGGCTCCTTAGGCTGAACGACCAAGCGGATATCAGTGGTCATGACGAAGAAAGAAGCTCGCTGATTG
AGAAGGGATGGAAAAAACTTCGAAAGCTCATCAAGAAGGTGTGGAAATACGTTAAGAAGCCTTTTAAGAAGACCGCGAAGATTATCA
AAAAGCCTTTTAAGAGTCGCACGAAGAACATTCATATAGTGTACTACAAAAGCCGTTTTTGATCTATAACAGTATACATGACTAG

>PhRXLR06_100 PLHAL100A10005
ACTTTAAAATCCCTCCAGCAGCGACGACACCCGTCATCGTTAGACCTGAAGGAAGAAAGGTGTTAGTATTTTGTACGTTTAAAATGC
GTATTCAGCTTCTGTGGCTTTCCTTTGCTGTGTTGAGCACCATTCTATCGACTTGTGATGCTACTTCTGACAAATTGGATCCGCAGA
GAGTGCAACCGAATCAAAATGGATCAGGACACAATCAATCCATTCGATCCGCTTTAAAGACCAGTCATGGCAAGACAATAGCAGATG
ATGAAGAGCGATTTATTTCGCTGTCAGGCATGTCGGAGAAGATTGCCAAGTATTACAAAGCTATTGTTGCCAAACTCTCTAAGTATT
TCAGAGACTATCACGAGAGGCGCGAAATAAGGAAGCAGCGAATTTTAAATAAAAGTTTCGCCGAGATGATGGCGGGACAAAAGTCAG
TGGAAGACATTGGAAGAAATCAAGACGCAAGTTTTATGTCAAGTTCATTCTTGTGGACACCTGAAGCATTCAAGAGCATACTGCATA
AGTACGCACTCTTTCTCTACAAGTATGGAAATGGACATCTTGCGACTGTACCAGTAAAAACTGGTTAAAAAGTTTCTTCGCCTGCAT
TTAGAACATTCAAGGTACATGACATTAACAGAGAAACACGTTTTCGCATTCGTT
>PhRXLR06_334 PLHAL3340002
ACTTTAAAATCCCTCCAGCAGCGACGACACCCGTCATCGTTAGACCTGAAGGAAGAAAGGTGTTAGTATTTTGTACGTTTAAAATGC
GTATTCAGCTTCTGTGGCTTTCCTTTGCTGTGTTGAGCACCATTCTATCGACTTGTGATGCTACTTCTGACAAATTGGATCCGCAGA
GAGTGCAACCGAATCAAAATGGATCAGGACACAATCAATCCATTCGATCCGCTTTAAAGACCAGTCATGGCAAGACAATAGCAGATG
ATGAAGAGCGATTTATTTCGCTGTCAGGCATGTCGGAGAAGATTGCCAAGTATTACAAAGCTATTGTTGCCAAACTCTCTAAGTATT
TCAGAGACTATCACGAGAGGCGCGAAATAAGGAAGCAGCGAATTTTAAATAAAAGTTTCGCCGAGATGATGGCGGGACAAAAGTCAG
TGGAAGACATTGGAAGAAATCAAGACGCAAGTTTTATGTCAAGTTCATTCTTGTGGACACCTGAAGCATTCAAGAGCATACTGCATA
AGTACGCACTCTTTCTCTACAAGTATGGAAATGGACATCTTGCGACTGTACCAGTAAAAACTGGTTAAAAAGTTTCTTCGCCTGCAT
TTAGAACATTCAAGGTACATGACATTAACAGAGAAACACGTTTTCGCATTCGTT
>PhRXLR06_703 PLHAL7030704
ACTTTAAAATCCCTCCAGCAGCGACGACACCCGTCATCGTTAGACCTGAAGGAAGAAAGGTGTTAGTATTTTGTACGTTTAAAATGC
GTATTCAGCTTCTGTGGCTTTCCTTTGCTGTGTTGAGCACCATTCTATCGACTTGTGATGCTACTTCTGACAAATTGGATCCGCAGA
GAGTGCAACCGAATCAAAATGGATCAGGACACAATCAATCCATTCGATCCGCTTTAAAGACCAGTCATGGCAAGACAATAGCAGATG
ATGAAGAGCGATTTATTTCGCTGTCAGGCATGTCGGAGAAGATTGCCAAGTATTACAAAGCTATTGTTGCCAAACTCTCTAAGTATT
TCAGAGACTATCACGAGAGGCGCGAAATAAGGAAGCAGCGAATTTTAAATAAAAGTTTCGCCGAGATGATGGCGGGACAAAAGTCAG
TGGAAGACATTGGAAGAAATCAAGACGCAAGTTTTATGTCAAGTTCATTCTTGTGGACACCTGAAGCATTCAAGAGCATACTGCATA
AGTACGCACTCTTTCTCTACAAGTATGGAAATGGACATCTTGCGACTGTACCAGTAAAAACTGGTTAAAAAGTTTCTTCGCCTGCAT
TTAGAACATTCAAGGTACATGACATTAACAGAGAAACACGTTTTCGCATTCGTT
>PhRXLR06_710 PLHAL7100176
ACTTTAAAATCCCTCCAGCAGCGACACCCGTCATCGTTAGACCTGAAGGAAGAAAGGTGTTAGTATTTTGTACGTTTAAAATGCGTA
TTCAGCTTCTGTGGCTTTCCTTTGCTGTGTTGAGCACCATTCTATCGACTTGTGATGCTACTTCTGACAAATTGGATCCGCAGAGAG
TGCAACCGAATCAAAATGGATCAGGACACAATCAATCCATTCGATCCGCTTTAAAGACCAGTCATGGCAAGACAATAGCAGATGATG
AAGAGCGATTTATTTCGCTGTCAGGCATGTCGGAGAAGATTGCCAAGTATTACAAAGCTATTGTTGCCAAACTCTCTAAGTATTTCA
GAGACTATCACGAGAGGCGCGAAATAAGGAAGCAGCGAATTTTAAATAAAAGTTTCGCCGAGATGATGGCGGGACAAAAGTCAGTGG
AAGACATTGGAAGAAATCAAGACGCAAGTTTTATGTCAAGTTCATTCTTGTGGACACCTGAAGCATTCAAGAGCATACTGCATAAGT
ACGCACTCTTTCTCTACAAGTATGGAAATGGACATCTTGCGACTGTACCAGTAAAAACTGGTTAAAAAGTTTCTTCGCCTGCATTTA
GAACATTCAAGGTACATGACATTAACAGAGAAACACGTTTTCGCATTCGTT
>PhRXLR06_304 PLHAL3040001
ACTTTAAAATCCCTCCAGCAGCGACGACACCCGTCATCGTTAGACCTGAAGGAAGAAAGGTGTTAGTATTTTGTACGTTTAAAATGC
GTATTCAGCTTCTGTGGCTTTCCTTTGCTGTGTTGAGCACCATTCTATCGACTTGTGATGCTACTTCTGACAAATTGGATCCGCAGA
GAGTGCAACCGAATCAAAATGGATCAGGACACAATCAATCCATTCGATCCGCTTTAAAGACCAGTCATGGCAAGACAATAGCAGATG
ATGAAGAGCGATTTATTTCGCTGTCAGGCATGTCGGAGAAGATTGCCAAGTATTACAAAGCTATTGTTGCCAAACTCTCTAAGTATT
TCAGAGACTATCACGAGAGGCGCGAAATAAGGAAGCAGCGAATTTTAAATAAAAGTTTCGCCGAGATGATGGCGGGACAAAAGTCAG
TGGAAGACATTGGAAGAAATCAAGACGCAAGTTTTATGTCAAGTTCATTCTTGTGGACACCTGAAGCATTCAAGAGCATACTGCATA
AGTACGCACTCTTTCTCTACAAGTATGGAAATGGACATCTTGCGACTGTACCAGTAAAAACTGGTTAAAAAGTTTCTTCGCCTGCAT
TTAGAACATTCAAGGTACATGACATTAACAGAGAAACACGTTTTCGCATTCGTT

>PhRXLR06_700 PLHAL7000116
ACTTTAAAATCCCTCCAGCAGCGACGACACCCGTCATCGTTAGACCTGAAGGAAGAAAGGTGTTAGTATTTTGTACGTTTAAAATGC
GTATTCAGCTTCTGTGGCTTTCCTTTGCTGTGTTGAGCACCATTCTATCGACTTGTGATGCTACTTCTGACAAATTGGATCCGCAGA
GAGTGCAACCGAATCAAAATGGATCAGGACACAATCAATCCATTCGATCCGCTTTAAAGACCAGTCATGGCAAGACAATAGCAGATG
ATGAAGAGCGATTTATTTCGCTGTCAGGCATGTCGGAGAAGATTGCCAAGTATTACAAAGCTATTGTTGCCAAACTCTCTAAGTATT
TCAGAGACTATCACGAGAGGCGCGAAATAAGGAAGCAGCGAATTTTAAATAAAAGTTTCGCCGAGATGATGGCGGGACAAAAGTCAG
TGGAAGACATTGGAAGAAATCAAGACGCAAGTTTTATGTCAAGTTCATTCTTGTGGACACCTGAAGCATTCAAGAGCATACTGCATA
AGTACGCACTCTTTCTCTACAAGTATGGAAATGGACATCTTGCGACTGTACCAGTAAAAACTGGTTAAAAAGTTTCTTCGCCTGCAT
TTAGAACATTCAAGGTACATGACATTAACAGAGAAACACGTTTTCGCATTCGTT
>PhRXLR06_730 PLHAL7300002:
ACTTTAAAATCCCTCCAGCAGCGACGACACCCGTCATCGTTAGACCTGAAGGAAGAAAGGTGTTAGTATTTTGTACGTTTAAAATGC
GTATTCAGCTTCTGTGGCTTTCCTTTGCTGTGTTGAGCACCATTCTATCGACTTGTGATGCTACTTCTGACAAATTGGATCCGCAGA
GAGTGCAACCGAATCAAAATGGATCAGGACACAATCAATCCATTCGATCCGCTTTAAAGACCAGTCATGGCAAGACAATAGCAGATG
ATGAAGAGCGATTTATTTCGCTGTCAGGCATGTCGGAGAAGATTGCCAAGTATTACAAAGCTATTGTTGCCAAACTCTCTAAGTATT
TCAGAGACTATCACGAGAGGCGCGAAATAAGGAAGCAGCGAATTTTAAATAAAAGTTTCGCCGAGATGATGGCGGGACAAAAGTCAG
TGGAAGACATTGGAAGAAATCAAGACGCAAGTTTTATGTCAAGTTCATTCTTGTGGACACCTGAAGCATTCAAGAGCATACTGCATA
AGTACGCACTCTTTCTCTACAAGTATGGAAATGGACATCTTGCGACTGTACCAGTAAAAACTGGTTAAAAAGTTTCTTCGCCTGCAT
TTAGAACATTCAAGGTACATGACATTAACAGAGAAACACGTTTTCGCATTCGTT

>PhRXLR07_100 PLHAL100A10475
AAACGAAAAGAGTTCATCCAGGTCAAAATCGCCAGTTTTATTTTGCGACTAAAAGGTTGTGACATTTAGCAAGACCGTCGATGCGTT
TGAGCTACATCTTCGTTGTGGTAGCCACCATTATTACCAACTGTGACATTGCGTCAGCTAGCCTTAGGGCAATAATGTCTGATACGG
CGTCAGGCAACGGCCTTGGTACCCGAATACTAAGACAAACAAACGACAGTGACGATTTGGAACCGATCAGACACGCGATGCTTGATA
TGGAGCTACTAGAGAAAATCGCCAAGGATCCAAAGTATGCTGAGGAAGTATTCGGTAACTGGCGGCATAATGGTCAGACAAAGGCAG
AGATGGAGAACCGACTTCAGTCCAATGGTCTTCTGGGAAAATATCGATTTATCATCGATCGATACGCTGAACATTTAGCGAACTCGG
AATAAATTGATTTCACTTTTCTTTTATAGCTACAGAGCATTGCTGTTGTTTCCTACGTGAATAT
>PhRXLR07_334 PLHAL3340459
AAACGAAAAGAGTTCATCCAGGTCAAAATCGCCAGTTTTATTTTGCGACTAAAAGGTTGTGACATTTAGCAAGACCGTCGATGCGTT
TGAGCTACATCTTCGTTGTGGTAGCCACCATTATTACCAACTGTGACATTGCGTCAGCTAGCCTTAGGGCAATAATGTCTGATACGG
CGTCAGGCAACGGCCTTGGTACCCGAATACTAAGACAAACAAACGACAGTGACGATTTGGAACCGATCAGACACGCGATGCTTGATA
TGGAGCTACTAGAGAAAATCGCCAAGGATCCAAAGTATGCTGAGGAAGTATTCGGTAACTGGCGGCATAATGGTCAGACAAAGGCAG
AGATGGAGAACCGACTTCAGTCCAATGGTCTTCTGGGAAAATATCGATTTATCATCGATCGATACGCTGAACATTTAGCGAACTCGG
AATAAATTGATTTCACTTTTCTTTTATAGCTACAGAGCATTGCTGTTGTTTCCTACGTGAATAT
>PhRXLR07_703 PLHAL7030171
AAACGAAAAGAGTTCATCCAGGTCAAAATCGCCAGTTTTATTTTGCGACTAAAAGGTTGTGACATTTAGCAAGACCGTCGATGCGTT
TGAGCTACATCTTCGTTGTGGTAGCCACCATTATTACCAACTGTGACATTGCGTCAGCTAGCCTTAGGGCAATAATGTCTGATACGG
CGTCAGGCAACGGCCTTGGTACCCGAATACTAAGACAAACAAACGACAGTGACGATTTGGAACCGATCAGACACGCGATGCTTGATA
TGGAGCTACTAGAGAAAATCGCCAAGGATCCAAAGTATGCTGAGGAAGTATTCGGTAACTGGCGGCATAATGGTCAGACAAAGGCAG
AGATGGAGAACCGACTTCAGTCCAATGGTCTTCTGGGAAAATATCGATTTATCATCGATCGATACGCTGAACATTTAGCGAACTCGG
AATAAATTGATTTCACTTTTCTTTTATAGCTACAGAGCATTGCTGTTGTTTCCTACGTGAATAT
>PhRXLR07_710 PLHAL7100526
AAACGAAAAGAGTTCATCCAGGTCAAAATCGCCAGTTTTATTTTGCGACTAAAAGGTTGTGACATTTAGCAAGACCGTCGATGCGTT
TGAGCTACATCTTCGTTGTGGTAGCCACCATTATTACCAACTGTGACATTGCGTCAGCTAGCCTTAGGGCAATAATGTCTGATACGG
CGTCAGGCAACGGCCTTGGTACCCGAATACTAAGACAAACAAACGACAGTGACGATTTGGAACCGATCAGACACGCGATGCTTGATA
TGGAGCTACTAGAGAAAATCGCCAAGGATCCAAAGTATGCTGAGGAAGTATTCGGTAACTGGCGGCATAATGGTCAGACAAAGGCAG
AGATGGAGAACCGACTTCAGTCCAATGGTCTTCTGGGAAAATATCGATTTATCATCGATCGATACGCTGAACATTTAGCGAACTCGG
AATAAATTGATTTCACTTTTCTTTTATAGCTACAGAGCATTGCTGTTGTTTCCTACGTGAATAT
>PhRXLR07_304 PLHAL3040494
AAACGAAAAGAGTTCATCCAGGTCAAAATCGCCAGTTTTATTTTGCGACTAAAAGGTTGTGACATTTAGCAAGACCGTCGATGCGTT
TGAGCTACATCTTCGTTGTGGTAGCCACCATTATTACCAACTGTGACATTGCGTCAGCTAGCCTTAGGGCAATAATGTCTGATACGG
CGTCAGGCAACGGCCTTGGTACCCGAATACTAAGACAAACAAACGACAGTGACGATTTGGAACCGATCAGACACGCGATGCTTGATA
TGGAGCTACTAGAGAAAATCGCCAAGGATCCAAAGTATGCTGAGGAAGTATTCGGTAACTGGCGGCATAATGGTCAGACAAAGGCAG
AGATGGAGAACCGACTTCAGTCCAATGGTCTTCTGGGAAAATATCGATTTATCATCGATCGATACGCTGAACATTTAGCGAACTCGG
AATAAATTGATTTCACTTTTCTTTTATAGCTACAGAGCATTGCTGTTGTTTCCTACGTGAATAT
>PhRXLR07_700 PLHAL7000084
AAACGAAAAGAGTTCATCCAGGTCAAAATCGCCAGTTTTATTTTGCGACTAAAAGGTTGTGACATTTAGCAAGACCGTCGATGCGTT
TGAGCTACATCTTCGTTGTGGTAGCCACCATTATTACCAACTGTGACATTGCGTCAGCTAGCCTTAGGGCAATAATGTCTGATACGG
CGTCAGGCAACGGCCTTGGTACCCGAATACTAAGACAAACAAACGACAGTGACGATTTGGAACCGATCAGACACGCGATGCTTGATA
TGGAGCTACTAGAGAAAATCGCCAAGGATCCAAAGTATGCTGAGGAAGTATTCGGTAACTGGCGGCATAATGGTCAGACAAAGGCAG
AGATGGAGAACCGACTTCAGTCCAATGGTCTTCTGGGAAAATATCGATTTATCATCGATCGATACGCTGAACATTTAGCGAACTCGG
AATAAATTGATTTCACTTTTCTTTTATAGCTACAGAGCATTGCTGTTGTTTCCTACGTGAATAT
>PhRXLR07_730 PLHAL7300503
AAACGAAAAGAGTTCATCCAGGTCAAAATCGCCAGTTTTATTTTGCGACTAAAAGGTTGTGACATTTAGCAAGACCGTCGATGCGTT
TGAGCTACATCTTCGTTGTGGTAGCCACCATTATTACCAACTGTGACATTGCGTCAGCTAGCCTTAGGGCAATAATGTCTGATACGG
CGTCAGGCAACGGCCTTGGTACCCGAATACTAAGACAAACAAACGACAGTGACGATTTGGAACCGATCAGACACGCGATGCTTGATA
TGGAGCTACTAGAGAAAATCGCCAAGGATCCAAAGTATGCTGAGGAAGTATTCGGTAACTGGCGGCATAATGGTCAGACAAAGGCAG

AGATGGAGAACCGACTTCAGTCCAATGGTCTTCTGGGAAAATATCGATTTATCATCGATCGATACGCTGAACATTTAGCGAACTCGG
AATAAATTGATTTCACTTTTCTTTTATAGCTACAGAGCATTGCTGTTGTTTCCTACGTGAATAT

>PhRXLR08_100 PLHAL100A10055
ACTTTGCAATATTTCTTGAAGCGATGCGTGTGGTTTTGCTTCTACTCCTGACTATTGCCGTCTCAGTCACCTATGTCTTGGCATCCT
CCGACGAGAACGATAAAGCTCTACACACATCCGATACAACGTATGGAAATGGGCAATTGCTGAGCAACGAGGCAGCTAACACTGAGA
CCCCAACTCATGATGATGAAGAGAGAAACCGTCCGATCGGGCAGCTGATGAGCGATAACTTGTTTAAATTCAAGATGATGTCGAAAA
CAGCTTTCTGGAATGCTAACAAAATTGCTGCATACTTAAAGAAGAAAAATATTACAGCGAAAAAGTGGCACCGAGTTTACCAGCTCC
AGAAACAAAAAAACCAAGAAGCAAAGTTACCTGATGGAAGTTATGTCAAATCGCTCGAGCAAGCAGTTTATGAATTACTTCCTGCCT
CTCAGAACATACAAAAAACGACTACTTCCAATCCTGTCGTCTAAGAACCTGACTTGACGTTTCTGTATCGGATCAGAGCGTCAGAAA
CTTATATAGTTTGGTTTGTAATGCGTATCATCACTTATATTCGCATTTGTTGCTATAGTTTGTTGATCATATGCAGTTAATATATTA
CGAATCGAGTC
>PhRXLR08_334 PLHAL3340010
ACTTTGCAATATTTCTTGAAGCGATGCGTGTGGTTTTGCTTCTACTCCTGACTATTGCCGTCTCAGTCACCTATGTCTTGGCATCCT
CCGACGAGAACGATAAAGCTCTACACACATCCGATACAACGTATGGAAATGGGCAATTGCTGAGCAACGAGGCAGCTAACACTGAGA
CCCCAACTCATGATGATGAAGAGAGAAACCGTCCGATCGGGCAGCTGATGAGCGATTACTTGTTTAAATTCAAGATGATGTCGAAAA
CAGCTCTCTGGAATGCTAACAAAATTGCTGCATACTTAAAGAAGAAAAATATTACAGCGAAAAAGTGGCACCGAGTTTACCAGCTCC
AGAAACAAAAAAACCAAGAAGCAAAGTTACCTGATGGAAGTTATGTCAAATCGCTCGAGCAAGAAGTTTATGAATTACTTACTGCCT
CTCAGAACATACAAAAAACGACTACTTCCAATCCTGTCGTCTAAGAACCTGACTTGACGTTTCTGTATCGGATCAGAGCGTCAGAAA
CTTATATAGTTTGGTTTGTAATGCGTATCATCACTTATATTCGCATTTGTTGCTATAGTTTGTTGATCATATGCAGTTAATATATTA
CGAATCGAGTC
>PhRXLR08_703 PLHAL7030013
ACTTTGCAATATTTCTTGAAGCGATGCGTGTGGTTTTGCTTCTACTCCTGACTATTGCCGTCTCAGTCACCTATGTCTTGGCATCCT
CCGACGAGAACGATAAAGCTCTACACACATCCGATACAACGTATGGAAATGGGCAATTGCTGAGCAACGAGGCAGCTAACACTGAGA
CCCCAACTCATGATGATGAAGAGAGAAACCGTCCGATCGGGCAGCTGATGAGCGATTACTTGTTTAAATTCAAGATGATGTCGAAAA
CAGCTCTCTGGAATGCTAACAAAATTGCTGCATACTTAAAGAAGAAAAATATTACAGCGAAAAAGTGGCACCGAGTTTACCAGCTCC
AGAAACAAAAAAACCAAGAAGCAAAGTTACCTGATGGAAGTTATGTCAAATCGCTCGAGCAAGAAGTTTATGAATTACTTACTGCCT
CTCAGAACATACAAAAAACGACTACTTCCAATCCTGTCGTCTAAGAACCTGACTTGACGTTTCTGTATCAGATCAGAGCGTCAGAAA
CTTATATAGTTTGGTTTGTAATGCGTATCATCACTTATATTCGCATTTGTTGCTATAGTTTGTTGATCATATGCAGTTAATATATTA
CGAATCGAGTC
>PhRXLR08_710 PLHAL7100028
ACTTTGCAATATTTCTTGAAGCGATGCGTGTGGTTTTGCTTCTACTCCTGACTATTGCCGTCTCAGTCACCTATGTCTTGGCATCCT
CCGACGAGAACGATAAAGCTCTACACACATCCGATACAACGTATGGAAATGGGCAATTGCTGAGCAACGAGGCAGCTAACACTGAGA
CCCCAACTCATGATGATGAAGAGAGAAACCGTCCGATCGGGCAGCTGATGAGCGATTACTTGTTTAAATTCAAGATGATGTCGAAAA
CAGCTCTCTGGAATGCTAACAAAATTGCTGCATACTTAAAGAAGAAAAATATTACAGCGAAAAAGTGGCACCGAGTTTACCAGCTCC
AGAAACAAAAAAACCAAGAAGCAAAGTTACCTGATGGAAGTTATGTCAAATCGCTCGAGCAAGAAGTTTATGAATTACTTACTGCCT
CTCAGAACATACAAAAAACGACTACTTCCAATCCTGTCGTCTAAGAACCTGACTTGACGTTTCTGTATCAGATCAGAGCGTCAGAAA
CTTATATAGTTTGGTTTGTAATGCGTATCATCACTTATATTCGCATTTGTTGCTATAGTTTGTTGATCATATGCAGTTAATATATTA
CGAATCGAGTC
>PhRXLR08_304 PLHAL3040024
ACTTTGCAATATTTCTTGAAGCGATGCGTGTGGTTTTGCTTCTACTCCTGACTATTGCCGTCTCAGTCACCTATGTCTTGGCATCCT
CCGACGAGAACGATAAAGCTCTACACACATCCGATACAACGTATGGAAATGGGCAATTGCTGAGCAACGAGGCAGCTAACACTGAGA
CCCCAACTCATGATGATGAAGAGAGAAACCGTCCGATCGGGCAGCTGATGAGCGATAACTTGTTTAAATTCAAGATGATGTCGAAAA
CAGCTTTCTGGAATGCTAACAAAATTGCTGCATACTTAAAGAAGAAAAATATTACAGCGAAAAAGTGGCACCGAGTTTACCAGCTCC
AGAAACAAAAAAACCAAGAAGCAAAGTTACCTGATGGAAGTTATGTCAAATCGCTCGAGCAAGCAGTTTATGAATTACTTCCTGCCT
CTCAGAACATACAAAAAACGACTACTTCCAATCCTGTCGTCTAAGAACCTGACTTGACGTTTCTGTATCGGATCAGAGCGTCAGAAA
CTTATATAGTTTGGTTTGTAATGCGTATCATCACTTATATTCGCATTTGTTGCTATAGTTTGTTGATCATATGCAGTTAATATATTA
CGAATCGAGTC
>PhRXLR08_700 PLHAL7000267
ACTTTGCAATATTTCTTGAAGCGATGCGTGTGGTTTTGCTTCTACTCCTGACTATTGCCGTCTCAGTCACCTATGTCTTGGCATCCT
CCGACGAGAACGATAAAGCTCTACACACATCCGATACAACGTATGGAAATGGGCAATTGCTGAGCAACGAGGCAGCTAACACTGAGA
CCCCAACTCATGATGATGAAGAGAGAAACCGTCCGATCGGGCAGCTGATGAGCGATTACTTGTTTAAATTCAAGATGATGTCGAAAA
CAGCTCTCTGGAATGCTAACAAAATTGCTGCATACTTAAAGAAGAAAAATATTACAGCGAAAAAGTGGCACCGAGTTTACCAGCTCC
AGAAACAAAAAAACCAAGAAGCAAAGTTACCTGATGGAAGTTATGTCAAATCGCTCGAGCAAGAAGTTTATGAATTACTTACTGCCT
CTCAGAACATACAAAAAACGACTACTTCCAATCCTGTCGTCTAAGAACCTGACTTGACGTTTCTGTATCAGATCAGAGCGTCAGAAA
CTTATATAGTTTGGTTTGTAATGCGTATCATCACTTATATTCGCATTTGTTGCTATAGTTTGTTGATCATATGCAGTTAATATATTA
CGAATCGAGTC
>PhRXLR08_730 PLHAL7300018
ACTTTGCAATATTTCTTGAAGCGATGCGTGTGGTTTTGCTTCTACTCCTGACTATTGCCGTCTCAGTCACCTATGTCTTGGCATCCT
CCGACGAGAACGATAAAGCTCTACACACATCCGATACAACGTATGGAAATGGGCAATTGCTGAGCAACGAGGCAGCTAACACTGAGA
CCCCAACTCATGATGATGAAGAGAGAAACCGTCCGATCGGGCAGCTGATGAGCGATTACTTGTTTAAATTCAAGATGATGTCGAAAA
CAGCTCTCTGGAATGCTAACAAAATTGCTGCATACTTAAAGAAGAAAAATATTACAGCGAAAAAGTGGCACCGAGTTTACCAGCTCC
AGAAACAAAAAAACCAAGAAGCAAAGTTACCTGATGGAAGTTATGTCAAATCGCTCGAGCAAGAAGTTTATGAATTACTTACTGCCT
CTCAGAACATACAAAAAACGACTACTTCCAATCCTGTCGTCTAAGAACCTGACTTGACGTTTCTGTATCAGATCAGAGCGTCAGAAA
CTTATATAGTTTGGTTTGTAATGCGTATCATCACTTATATTCGCATTTGTTGCTATAGTTTGTTGATCATATGCAGTTAATATATTA
CGAATCGAGTC

>PhRXLR13_100 PLHAL100A10114
ATCTGCAGACATGCGTCTTCACCTACTAGTACTAAGTGTGATTGTGGTATCGTTGTTAGTCTCCGACAATGCGCATGCGAACTCACA
CGACGACAGCAAGACCAGAGCGTTAAGAGAAACTCCAATCAATGGACTCGTCACCAACCAATTGGCCGTTAGTCGTAATTTGACACC
AGCCAAGTTTATCACTAACAGTGAAGAGCGCCATAGCAGCGAAAAAAAGTCTAGGCGACTTCAAATCTACTTCCACTCGCCCTATTA
CGGTATCCATCCTGTTGATTATCATTATGTGGGGTCATATGAGAGTGGAGTTACGACGATTTGTTCAATAGTATTGTTTGTGATGGT
TTTTGGATGCCTTTACAAGATTTTCTCTCAGTGAGGCCATGTTCGACATTCGTGGCGTAATTTCATAATTAAATTAATGTCACAATA
ATGCCGAGTTTGTCAGTATTTTTTTAAAAAGTTAGTGTCCATGGGTAACATCATCAGCAACATTTTTGCCAGATTGCCAGGAGTATA
GACAACGAGTTTGTATCGTTGTTGTGATCGTACCGACAAGACTGTAGTCAGTTTGCATGTCTTGTGGCATCTATACCCGACGCTTTT
TTTACGGCAACGAAGTCGGCAGTTTTAGCAATTATTTGGTCAGGAACTTTATTGCCCAGAATCTAACTTTTCTTTTGTTCATAGCAG
AGCCTAAGCTACACACCGAGAAGAAGAGTCT
>PhRXLR13_334 PLHAL3340032
ATCTGCAGACATGCGTCTTCACCTACTAGTACTAAGTGTGATTGTGGTATCGTTGTTAGTCTCCGACAATGCGCATGCGAACTCACA
CGACGACAGCAAGACCAGAGCGTTAAGAGAAACTCCAATCAATGGACTCGTCACCAACCAATTGGCCGTTAGTCGTAATTTGACACC
AGCCAAGTTTATCACTAACAGTGAAGAGCGCCATAGCAGCGAAAAAAAGTCTAGGCGACTTCAAATCTACTTCCACTCGCCCTATTA
CGGTATCCATCCTGTTGATTATCATTATGTGGGGTCATATGAGAGTGGAGTTACGACGATTTGTTCAATAGTATTGTTTGTGATGGT
TTTTGGATGCCTTTACAAGATTTTCTCTCAGTGAGGCCATGTTCGACATTCGTGGCGTAATTTCATAATTAAATTAATGTCACAATA
ATGCCGAGTTTGTCAGTATTTTTTTAAAAAGTTAGTGTCCATGGGTAACATCATCAGCAACATTTTTGCCAGATTGCCAGGAGTATA
GACAACGAGTTTGTATCGTTGTTGTGATCGTACCGACAAGACTGTAGTCAGTTTGCATGTCTTGTGGCATCTATACCCGACGCTTTT
TTTACGGCAACGAAGTCGGCAGTTTTAGCAATTATTTGGTCAGGAACTTTATTGCCCAGAATCTAACTTTTCTTTTGTTCATAGCAG
AGCCTAAGCTACACACCGAGAAGAAGAGTCT
>PhRXLR13_703 PLHAL7030156
ATCTGCAGACATGCGTCTTCACCTACTAGTACTAAGTGTGATTGTGGTATCGTTGTTAGTCTCCGACAATGCGCATGCGAACTCACA
CGACGACAGCAAGACCAGAGCGTTAAGAGAAACTCCAATCAATGGACTCGTCACCAACCAATTGGCCGTTAGTCGTAATTTGACACC
AGCCAAGTTTATCACTAACAGTGAAGAGCGCCATAGCAGCGAAAAAAAGTCTAGGCGACTTCAAATCTACTTCCACTCGCCCTATTA
CGGTATCCATCCTGTTGATTATCATTATGTGGGGTCATATGAGAGTGGAGTTACGACGATTTGTTCAATAGTATTGTTTGTGATGGT
TTTTGGATGCCTTTACAAGATTTTCTCTCAGTGAGGCCATGTTCGACATTCGTGGCGTAATTTCATAATTAAATTAATGTCACAATA
ATGCCGAGTTTGTCAGTATTTTTTTAAAAAGTTAGTGTCCATGGGTAACATCATCAGCAACATTTTTGCCAGATTGCCAGGAGTATA
GACAACGAGTTTGTATCGTTGTTGTGATCGTACCGACAAGACTGTAGTCAGTTTGCATGTCTTGTGGCATCTATACCCGACGCTTTT
TTTACGGCAACGAAGTCGGCAGTTTTAGCAATTATTTGGTCAGGAACTTTATTGCCCAGAATCTAACTTTTCTTTTGTTCATAGCAG
AGCCTAAGCTACACACCGAGAAGAAGAGTCT
>PhRXLR13_710 PLHAL7100204
ATCTGCAGACATGCGTCTTCACCTACTAGTACTAAGTGTGATTGTGGTATCGTTGTTAGTCTCCGACAATGCGCATGCGAACTCACA
CGACGACAGCAAGACCAGAGCGTTAAGAGAAACTCCAATCAATGGACTCGTCACCAACCAATTGGCCGTTAGTCGTAATTTGACACC
AGCCAAGTTTATCACTAACAGTGAAGAGCGCCATAGCAGCGAAAAAAAGTCTAGGCGACTTCAAATCTACTTCCACTCGCCCTATTA
CGGTATCCATCCTGTTGATTATCATTATGTGGGGTCATATGAGAGTGGAGTTACGACGATTTGTTCAATAGTATTGTTTGTGATGGT
TTTTGGATGCCTTTACAAGATTTTCTCTCAGTGAGGCCATGTTCGACATTCGTGGCGTAATTTCATAATTAAATTAATGTCACAATA
ATGCCGAGTTTGTCAGTATTTTTTTAAAAAGTTAGTGTCCATGGGTAACATCATCAGCAACATTTTTGCCAGATTGCCAGGAGTATA
GACAACGAGTTTGTATCGTTGTTGTGATCGTACCGACAAGACTGTAGTCAGTTTGCATGTCTTGTGGCATCTATACCCGACGCTTTT
TTTACGGCAACGAAGTCGGCAGTTTTAGCAATTATTTGGTCAGGAACTTTATTGCCCAGAATCTAACTTTTCTTTTGTTCATAGCAG
AGCCTAAGCTACACACCGAGAAGAAGAGTCT
>PhRXLR13_304 PLHAL3040095
ATCTGCAGACATGCGTCTTCACCTACTAGTACTAAGTGTGATTGTGGTATCGTTGTTAGTCTCCGACAATGCGCATGCGAACTCACA
CGACGACAGCAAGACCAGAGCGTTAAGAGAAACTCCAATCAATGGACTCGTCACCAACCAATTGGCCGTTAGTCGTAATTTGACACC
AGCCAAGTTTATCACTAACAGTGAAGAGCGCCATAGCAGCGAAAAAAAGTCTAGGCGACTTCAAATCTACTTCCACTCGCCCTATTA
CGGTATCCATCCTGTTGATTATCATTATGTGGGGTCATATGAGAGTGGAGTTACGACGATTTGTTCAATAGTATTGTTTGTGATGGT
TTTTGGATGCCTTTACAAGATTTTCTCTCAGTGAGGCCATGTTCGACATTCGTGGCGTAATTTCATAATTAAATTAATGTCACAATA
ATGCCGAGTTTGTCAGTATTTTTTTAAAAAGTTAGTGTCCATGGGTAACATCATCAGCAACATTTTTGCCAGATTGCCAGGAGTATA
GACAACGAGTTTGTATCGTTGTTGTGATCGTACCGACAAGACTGTAGTCAGTTTGCATGTCTTGTGGCATCTATACCCGACGCTTTT
TTTACGGCAACGAAGTCGGCAGTTTTAGCAATTATTTGGTCAGGAACTTTATTGCCCAGAATCTAACTTTTCTTTTGTTCATAGCAG
AGCCTAAGCTACACACCGAGAAGAAGAGTCT
>PhRXLR13_700 PLHAL7000085
ATCTGCAGACATGCGTCTTCACCTACTAGTACTAAGTGTGATTGTGGTATCGTTGTTAGTCTCCGACAATGCGCATGCGAACTCACA
CGACGACAGCAAGACCAGAGCGTTAAGAGAAACTCCAATCAATGGACTCGTCACCAACCAATTGGCCGTTAGTCGTAATTTGACACC
AGCCAAGTTTATCACTAACAGTGAAGAGCGCCATAGCAGCGAAAAAAAGTCTAGGCGACTTCAAATCTACTTCCACTCGCCCTATTA
CGGTATCCATCCTGTTGATTATCATTATGTGGGGTCATATGAGAGTGGAGTTACGACGATTTGTTCAATAGTATTGTTTGTGATGGT
TTTTGGATGCCTTTACAAGATTTTCTCTCAGTGAGGCCATGTTCGACATTCGTGGCGTAATTTCATAATTAAATTAATGTCACAATA
ATGCCGAGTTTGTCAGTATTTTTTTAAAAAGTTAGTGTCCATGGGTAACATCATCAGCAACATTTTTGCCAGATTGCCAGGAGTATA
GACAACGAGTTTGTATCGTTGTTGTGATCGTACCGACAAGACTGTAGTCAGTTTGCATGTCTTGTGGCATCTATACCCGACGCTTTT
TTTACGGCAACGAAGTCGGCAGTTTTAGCAATTATTTGGTCAGGAACTTTATTGCCCAGAATCTAACTTTTCTTTTGTTCATAGCAG
AGCCTAAGCTACACACCGAGAAGAAGAGTCT
>PhRXLR13_730 PLHAL7300142
ATCTGCAGACATGCGTCTTCACCTACTAGTACTAAGTGTGATTGTGGTATCGTTGTTAGTCTCCGACAATGCGCATGCGAACTCACA
CGACGACAGCAAGACCAGAGCGTTAAGAGAAACTCCAATCAATGGACTCGTCACCAACCAATTGGCCGTTAGTCGTAATTTGACACC
AGCCAAGTTTATCACTAACAGTGAAGAGCGCCATAGCAGCGAAAAAAAGTCTAGGCGACTTCAAATCTACTTCCACTCGCCCTATTA
CGGTATCCATCCTGTTGATTATCATTATGTGGGGTCATATGAGAGTGGAGTTACGACGATTTGTTCAATAGTATTGTTTGTGATGGT
TTTTGGATGCCTTTACAAGATTTTCTCTCAGTGAGGCCATGTTCGACATTCGTGGCGTAATTTCATAATTAAATTAATGTCACAATA
ATGCCGAGTTTGTCAGTATTTTTTTAAAAAGTTAGTGTCCATGGGTAACATCATCAGCAACATTTTTGCCAGATTGCCAGGAGTATA

GACAACGAGTTTGTATCGTTGTTGTGATCGTACCGACAAGACTGTAGTCAGTTTGCATGTCTTGTGGCATCTATACCCGACGCTTTT
TTTACGGCAACGAAGTCGGCAGTTTTAGCAATTATTTGGTCAGGAACTTTATTGCCCAGAATCTAACTTTTCTTTTGTTCATAGCAG
AGCCTAAGCTACACACCGAGAAGAAGAGTCT

>PhRXLR15_100 PLHAL100A10710
CTAAGTTCGGTCTCATGGGTGGCGCTTTCTCCAAGGTCGCTGTGCTCATCATTTCAGCCCCCACCCTCACAGCTGTTAAATCCTACG
TCCCCAATTCTCAGACGGAGCAAAATCATGACATCGTGCCGATTGGTGGTACAGACACCGATATGTTGCCCAAGAGATCTCTCCAAG
GGAGTTATGACCAAGTAGCGATCCCTGTGGTTGAAGAAGAGCGTGCATCTTTAGCTAATTTGATTGAAAATGCAGGAGAAGATGCGA
TTAGAATACTGGTTTCTTCACATATGGAGAGGACTGAAAATAACGTTCTGGAGTGTTTAGAAAAGAAGATTATGAATGGAGAATACC
TCGGTTCTCCCGCCGACCGAGATGCCATCATCAAGTTATCCTCTTACCACGCCAAAAAAGTTGAAGAGGGCAACGATATATCAACCG
CAACCACGAATATAATGAATCGCTATGACCCGATCTCTTTTATCCGTCGAGATGTCAAAGCTCTACATGATGGCTCCTCCGTCGCCG
CCAGTAAAGACATCCAATTTCTTGAGGAGGCCTTCGACATTTACATGTTTTGCGTCGGCGAAATTGAGAACTACGCTCAAGAATTAA
AAGACGCCATAGCTGAAGTCGCACATCATACCAAAGAAGCTACAGTAGCTCTCATTGTCGCCAACTTAGCCCGTGATATCAATTCTG
CCCACTCCGCTGAAATGGCCGAATCTTCCTATGCCCACCACACTATG
>PhRXLR15_334 PLHAL3340702
CTAAGTTCGGTCTCATGGGTGGCGCTTTCTCCAAGGTCGCTGTGCTCATCATTTCAGCCCCCACCCTCACAGCTGTTAAATCCTACG
TCCCCAATTCTCAGACGGAGCAAAATCATGACATCGTGCCGATTGGTGGTACAGACACCGATATGTTGCCCAAGAGATCTCTCCAAG
GGAGTTATGACCAAGTAGCGATCCCTGTGGTTGAAGAAGAGCGTGCATCTTTAGCTAATTTGATTGAAAATGCAGGAGAAGATGCGA
TTAGAATACTGGTTTCTTCACATATGGAGATCACTGAAAATAACGTTCTGGAGTGTTTAGAAAAGAAGATTATGAATGGAGAATACC
TCGGTTCTCCCGCCGACCGAGATGCCATCATCAAGTTATCCTCTTACCACGCCAAAAAAGTTGAAGAGGGCAACGATATATCAACCG
CAACCACGAATATAATGAATCGCTATGACCCGATCTCTTTTATCCGTCGAGATGTCAAAGCTCTACATGATGGCTCCTCCGTCGCCG
CCAGTAAAGACATCCAATTTCTTGAGGAGGCCTTCGACATTTACATGTTTTGCGTCGGCGAAATTGAGAACTACGCTCAAGAATTAA
AAGACGCCATAGCTGAAGTCGCACATCATACCAAAGAAGCTACAGTAGCTCTCATTGTCGCCAACTTAGCCCGTGATATCAATTCTG
CCCACTCCGCTGAAATGGCCGAATCTTCCTATGCCCACCACACTATG
>PhRXLR15_703 PLHAL7030737
CTAAGTTCGGTCTCATGGGTGGCGCTTTCTCCAAGGTCGCTGTGCTCATCATTTCAGCCCCCACCCTCACAGCTGTTAAATCCTACG
TCCCCAATTCTCAGACGGAGCAAAATCATGACATCGTGCCGATTGGTGGTACAGACACCGATATGTTGCCCAAGAGATCTCTCCAAG
GGAGTTATGACCAAGTAGCGATCCCTGTGGTTGAAGAAGAGCGTGCATCTTTAGCTAATTTGATTAAAAATGCAGGAGAAGATGCGA
TTAGAATACTGATTTCTTCACATGTGGAGAGGACTGAAAATAACGTTCCGGAGTGTTTAGAAAAGGAGATTATGAATGGAGAATACC
TCGGTTCTCCCGCCGACCGAGATGCCATCATCAAGTTATCCTCTTACCACGCCAAAAAAGTTGAAGAGGGCAACGATATATCAACCG
CAACCACGAATATAATGAATCGCTATGACCCGATCTCTTTTATCCGTCGAGATGTCAAAGCTCTACATGATGGCTCCTCCGTCGCCG
CCAGTAAAGACATCCAATTTCTTGAGGAGGCCTTCGACATTTACATGTTTTGCGTCGGCGAAATTGAGAACTACGCTCAAGAATTAA
AAGACGCCATAGCTGAAGTCGCACATCATACCAAAGAAGCTACAGTAGCTCTCATTGTCGCCAACTTAGCCCGTGATATCAATTCTG
CCCACTCCGCTGAAATGGCCGAATCTTCCTATGCCCACCACACTATG
>PhRXLR15_710 PLHAL7100720
CTAAGTTCGGTCTCATGGGTGGCGCTTTCTCCAAGGTCGCTGTGCTCATCATTTCAGCCCCCACCCTCACAGCTGTTAAATCCTACG
TCCCCAATTCTCAGACGGAGCAAAATCATGACATCGTGCCGATTGGTGGTACAGACACCGATATGTTGCCCAAGAGATCTCTCCAAG
GGAGTTATGACCAAGTAGCGATCCCTGTGGTTGAAGAAGAGCGTGCATCTTTAGCTAATTTGATTGAAAATGCAGGAGAAGATGCGA
TTAGAATACTGGTTTCTTCACATATGGAGAGGACTGAAAATAACGTTCTGGAGTGTTTAGAAAAGAAGATTATGAATGGAGAATACC
TCGGTTCTCCCGCCGACCGAGATGCCATCATCAAGTTATCCTCTTACCACGCCAAAAAAGTTGAAGAGGGCAACGATATATCAACCG
CAACCACGAATATAATGAATCGCTATGACCCGATCTCTTTTATCCGTCGAGATGTCAAAGCTCTACATGATGGCTCCTCCGTCGCCG
CCAGTAAAGACATCCAATTTCTTGAGGAGGCCTTCGACATTTACATGTTTTGCGTCGGCGAAATTGAGAACTACGCTCAAGAATTAA
AAGACGCCATAGCTGAAGTCGCACATCATACCAAAGAAGCTACAGTAGCTCTCATTGTCGCCAACTTAGCCCGTGATATCAATTCTG
CCCACTCCGCTGAAATGGCCGAATCTTCCTATGCCCACCACACTATG
>PhRXLR15_304 PLHAL3040711
CTAAGTTCGGTCTCATGGGTGGCGCTTTCTCCAAGGTCGCTGTGCTCATCATTTCAGCCCCCACCCTCACAGCTGTTAAATCCTACG
TCCCCAATTCTCAGACGGAGCAAAATCATGACATCGTGCCGATTGGTGGTACAGACACCGATATGTTGCCCAAGAGATCTCTCCAAG
GGAGTTATGACCAAGTAGCGATCCCTGTGGTTGAAGAAGAGCGTGCATCTTTAGCTAATTTGATTGAAAATGCAGGAGAAGATGCGA
TTAGAATACTGGTTTCTTCACATATGGAGAGGACTGAAAATAACGTTCTGGAGTGTTTAGAAAAGAAGATTATGAATGGAGAATACC
TCGGTTCTCCCGCCGACCGAGATGCCATCATCAAGTTATCCTCTTACCACGCCAAAAAAGTTGAAGAGGGCAACGATATATCAACCG
CAACCACGAATATAATGAATCGCTATGACCCGATCTCTTTTATCCGTCGAGATGTCAAAGCTCTACATGATGGCTCCTCCGTCGCCG
CCAGTAAAGACATCCAATTTCTTGAGGAGGCCTTCGACATTTACATGTTTTGCGTCGGCGAAATTGAGAACTACGCTCAAGAATTAA
AAGACGCCATAGCTGAAGTCGCACATCATACCAAAGAAGCTACAGTAGCTCTCATTGTCGCCAACTTAGCCCGTGATATCAATTCTG
CCCACTCCGCTGAAATGGCCGAATCTTCCTATGCCCACCACACTATG
>PhRXLR15_700 PLHAL7000699
CTAAGTTCGGTCTCATGGGTGGCGCTTTCTCCAAGGTCGCTGTGCTCATCATTTCAGCCCCCACCCTCACAGCTGTTAAATCCTACG
TCCCCAATTCTCAGACGGAGCAAAATCATGACATCGTGCCGATTGGTGGTACAGACACCGATATGTTGCCCAAGAGATCTCTCCAAG
GGAGTTATGACCAAGTAGCGATCCCTGTGGTTGAAGAAGAGCGTGCATCTTTAGCTAATTTGATTGAAAATGCAGGAGAAGATGCGA
TTAGAATACTGGTTTCTTCACATATGGAGAGGACTGAAAATAACGTTCTGGAGTGTTTAGAAAAGAAGATTATGAATGGAGAATACC
TCGGTTCTCCCGCCGACCGAGATGCCATCATCAAGTTATCCTCTTACCACGCCAAAAAAGTTGAAGAGGGCAACGATATATCAACCG
CAACCACGAATATAATGAATCGCTATGACCCGATCTCTTTTATCCGTCGAGATGTCAAAGCTCTACATGATGGCTCCTCCGTCGCCG
CCAGTAAAGACATCCAATTTCTTGAGGAGGCCTTCGACATTTACATGTTTTGCGTCGGCGAAATTGAGAACTACGCTCAAGAATTAA
AAGACGCCATAGCTGAAGTCGCACATCATACCAAAGAAGCTACAGTAGCTCTCATTGTCGCCAACTTAGCCCGTGATATCAATTCTG
CCCACTCCGCTGAAATGGCCGAATCTTCCTATGCCCACCACACTATG
>PhRXLR15_730 PLHAL7300744
CTAAGTTCGGTCTCATGGGTGGCGCTTTCTCCAAGGTCGCTGTGCTCATCATTTCAGCCCCCACCCTCACAGCTGTTAAATCCTACG
TCCCCAATTCTCAGACGGAGCAAAATCATGACATCGTGCCGATTGGTGGTACAGACACCGATATGTTGCCCAAGAGATCTCTCCAAG

GGAGTTATGACCAAGTAGCGATCCCTGTGGTTGAAGAAGAGCGTGCATCTTTAGCTAATTTGATTGAAAATGCAGGAGAAGATGCGA
TTAGAATACTGGTTTCTTCACATATGGAGAGGACTGAAAATAACGTTCTGGAGTGTTTAGAAAAGAAGATTATGAATGGAGAATACC
TCGGTTCTCCCGCCGACCGAGATGCCATCATCAAGTTATCCTCTTACCACGCCAAAAAAGTTGAAGAGGGCAACGATATATCAACCG
CAACCACGAATATAATGAATCGCTATGACCCGATCTCTTTTATCCGTCGAGATGTCAAAGCTCTACATGATGGCTCCTCCGTCGCCG
CCAGTAAAGACATCCAATTTCTTGAGGAGGCCTTCGACATTTACATGTTTTGCGTCGGCGAAATTGAGAACTACGCTCAAGAATTAA
AAGACGCCATAGCTGAAGTCGCACATCATACCAAAGAAGCTACAGTAGCTCTCATTGTCGCCAACTTAGCCCGTGATATCAATTCTG
CCCACTCCGCTGAAATGGCCGAATCTTCCTATGCCCACCACACTATG

>PhRXLR18_100 PLHAL100A10251
GCATAAGTTTTAGCGAGAAACATTGTTATTCATACACATAGCAAATTGCTTCATCGTCTCCAAGCCTCCAGTGGCATCCAAGATCTG
CGAAGATGCTCTTTGCTTTCATTTTCGTTATCATGTCTGTGGCTGTGTCTGGAAACATCAGTTATGCCATCACAGCCACGCAAGTTC
AACAAACGTTGGCCTTAAGGAGCGCTCGTTGGAAGCCTTCGACCTCGCTAAAACTTCTGAAGCGAGAAGGTTTCTGCGAAGTAGAGA
GCTTGACGCCGTGAAGGAAGAGCGTGTCTCTTCTAAAATGGATTTCATCCCAGAATCAGAATTCAGCTCCAAGGTCCTGAAGGCGAT
CAAGAAGTCAAATAAAGAGCATAACAACGCATTTATGCTAACACGTAAGCCCAAGACTCCTTTGAGCAAATCTAGATTTAAAAGAGA
GGTGCTGGTGGATGCCGTCTTCGATAAACTTAACAATCTAAAACTTGATAATCCTAATAGACCGTAATGAGACACAGCTCCATTTTT
TCATAT
>PhRXLR18_334 PLHAL3340167
GCATAAGTTTTAGCGAGAAACATTGTTATTCATACACATAGCAAATTGCTTCATCGTCTCCAAGCCTCCAGTGGCATCCAAGATCTG
CGAAGATGCTCTTTGCTTTCATTTTCGTTATCATGTCTGTGGCAGTGTCTGGAAACATCAGTTATGCCATCACAGCCACGCAAGTTC
AACAAACGTTGGCCTTGAGCGAGCGCTCGTTGGAAGCCTTCGACCTCGCTAAAACTTCTGAAGCGAGAAGGTTTCTGCGAAGTAGAG
AGCTTGACGCCGTGAAGGAAGAGCGTGTCTCTTCTAAAATGGATTTCATCCCAGAATCAGAATTCAGCTCCAAGGTCCTGAAGGCGA
TCAAGAAGTCAAATAAAGAGCATAACAACGCATTTATGCTATCACGTAAGCCCAAGACTCCTTTGAGCAAATCTAGATTTAAAAGAG
AGGTGCTGGTGGATGCCGTCTTCGATAAACTTAACAATTTAAAACTTGATTATCCTAATAGACCGTAATGAGACACAGCTCCATTTT
TTCATAT
>PhRXLR18_703 PLHAL70302937
GCATAAGTTTTAGCGAGAAACATTGTTATTCATACACATAGCAAATTGCTTCATCGTCTCCAAGCCTCCAGTGGCATCCAAGATCTG
CGAAGATGCTCTTTGCTTTCATTTTCGTTATCATGTCTGTGGCAGTGTCTGGAAACATCAGTTATGCCATCACAGCCACGCAAGTTC
AACAAACGTTGGCCTTGAGCGAGCGCTCGTTGGAAGCCTTCGACCTCGCTAAAACTTCTGAAGCGAGAAGGTTTCTGCGAAGTAGAG
AGCTTGACGCCGTGAAGGAAGAGCGTGTCTCTTCTAAAATGGATTTCATCCCAGAATCAGAATTCAGCTCCAAGGTCCTGAAGGCGA
TCAAGAAGTCAAATAAAGAGCATAACAACGCATTTATGCTATCACGTAAGCCCAAGACTCCTTTGAGCAAATCTAGATTTAAAAGAG
AGGTGCTGGTGGATGCCGTCTTCGATAAACTTAACAATTTAAAACTTGATTATCCTAATAGACCGTAATGAGACACAGCTCCATTTT
TTCATAT
>PhRXLR18_710 PLHAL7100338
GCATAAGTTTTAGCGAGAAACATTGTTATTCATACACATAGCAAATTGCTTCATCGTCTCCAAGCCTCCAGTGGCATCCAAGATCTG
CGAAGATGCTCTTTGCTTTCATTTTCGTTATCATGTCTGTGGCAGTGTCTGGAAACATCAGTTATGCCATCACAGCCACGCAAGTTC
AACAAACGTTGGCCTTGAGCGAGCGCTCGTTGGAAGCCTTCGACCTCGCTAAAACTTCTGAAGCGAGAAGGTTTCTGCGAAGTAGAG
AGCTTGACGCCGTGAAGGAAGAGCGTGTCTCTTCTAAAATGGATTTCATCCCAGAATCAGAATTCAGCTCCAAGGTCCTGAAGGCGA
TCAAGAAGTCAAATAAAGAGCATAACAACGCATTTATGCTATCACGTAAGCCCAAGACTCCTTTGAGCAAATCTAGATTTAAAAGAG
AGGTGCTGGTGGATGCCGTCTTCGATAAACTTAACAATTTAAAACTTGATTATCCTAATAGACCGTAATGAGACACAGCTCCATTTT
TTCATAT
>PhRXLR18_304 PLHAL3040327
GCATAAGTTTTAGCGAGAAACATTGTTATTCATACACATAGCAAATTGCTTCATCGTCTCCAAGCCTCCAGTGGCATCCAAGATCTG
CGAAGATGCTCTTTGCTTTCATTTTCGTTATCATGTCTGTGGCTGTGTCTGGAAACATCAGTTATGCCATCACAGCCACGCAAGTTC
AACAAACGTTGGCCTTAAGGAGCGCTCGTTGGAAGCCTTCGACCTCGCTAAAACTTCTGAAGCGAGAAGGTTTCTGCGAAGTAGAGA
GCTTGACGCCGTGAAGGAAGAGCGTGTCTCTTCTAAAATGGATTTCATCCCAGAATCAGAATTCAGCTCCAAGGTCCTGAAGGCGAT
CAAGAAGTCAAATAAAGAGCATAACAACGCATTTATGCTAACACGTAAGCCCAAGACTCCTTTGAGCAAATCTAGATTTAAAAGAGA
GGTGCTGGTGGATGCCGTCTTCGATAAACTTAACAATCTAAAACTTGATAATCCTAATAGACCGTAATGAGACACAGCTCCATTTTT
TCATAT
>PhRXLR18_700 PLHAL7000181
GCATAAGTTTTAGCGAGAAACATTGTTATTCATACACATAGCAAATTGCTTCATCGTCTCCAAGCCTCCAGTGGCATCCAAGATCTG
CGAAGATGCTCTTTGCTTTCATTTTCGTTATCATGTCTGTGGCAGTGTCTGGAAACATCAGTTATGCCATCACAGCCACGCAAGTTC
AACAAACGTTGGCCTTGAGCGAGCGCTCGTTGGAAGCCTTCGACCTCGCTAAAACTTCTGAAGCGAGAAGGTTTCTGCGAAGTAGAG
AGCTTGACGCCGTGAAGGAAGAGCGTGTCTCTTCTAAAATGGATTTCATCCCAGAATCAGAATTCAGCTCCAAGGTCCTGAAGGCGA
TCAAGAAGTCAAATAAAGAGCATAACAACGCATTTATGCTATCACGTAAGCCCAAGACTCCTTTGAGCAAATCTAGATTTAAAAGAG
AGGTGCTGGTGGATGCCGTCTTCGATAAACTTAACAATTTAAAACTTGATTATCCTAATAGACCGTAATGAGACACAGCTCCATTTT
TTCATAT
>PhRXLR18_730 PLHAL7300012
GCATAAGTTTTAGCGAGAAACATTGTTATTCATACACATAGCAAATTGCTTCATCGTCTCCAAGCCTCCAGTGGCATCCAAGATCTG
CGAAGATGCTCTTTGCTTTCATTTTCGTTATCATGTCTGTGGCAGTGTCTGGAAACATCAGTTATGCCATCACAGCCACGCAAGTTC
AACAAACGTTGGCCTTGAGCGAGCGCTCGTTGGAAGCCTTCGACCTCGCTAAAACTTCTGAAGCGAGAAGGTTTCTGCGAAGTAGAG
AGCTTGACGCCGTGAAGGAAGAGCGTGTCTCTTCTAAAATGGATTTCATCCCAGAATCAGAATTCAGCTCCAAGGTCCTGAAGGCGA
TCAAGAAGTCAAATAAAGAGCATAACAACGCATTTATGCTATCACGTAAGCCCAAGACTCCTTTGAGCAAATCTAGATTTAAAAGAG
AGGTGCTGGTGGATGCCGTCTTCGATAAACTTAACAATTTAAAACTTGATTATCCTAATAGACCGTAATGAGACACAGCTCCATTTT
TTCATAT

>PhRXLR22_100 PLHAL100A11736
TCATGTTGCAATTCGCGACTGCTTTCCTTGCAATCAGTGCAAACGTTGTCATGACGCAGCCAAATCAAGAATACATTGCTCCCGGGG
CTCGTGTCAGCTCTAATTACCGCGACATGACCATCCGCCGCTTACGTACTCACGAAATTGGGACAGTCCCAGAAGAGAGGATGCCTA
TACACGAATTGGAAATTGAAGATCTAATCACCATTATCGCCTCTAAGGTGGTCCCCGAATTATCGCACTCTGAGTCAATGTCTCTAC
TAAGCGCTGAGGCAGCACGGAGTCGGAGTTTTAAGGATATGGCAGCTGATGAACTCGATGTGGATGGTGCGCTGGATTTGCTAAAAG
CTGTGCAATCAAAGCCGGAAACCACAGACATGACTCC
>PhRXLR22_334 PLHAL3341813
TCATGTTGCAATTCGCGACTGCTTTCCTTGCAATCAGTGCAAACGTTGTCATGACGCAGCCAAATCAAGAATACATTGCTCCCGGGG
CTCGTGTCAGCTCTAATTACCGCGACATGACCATCCGCCGCTTACGTACTCACGAAATTGGGACAGTCCCAGAAGAGAGGATGCCTA
TACACGAATTGGAAATTGAAGATCTAATCACCATTATCGCCTCTAAGGTGGTCCCCGAATTATCGCACTCTGAGTCAATGTCTCTAC
TAAGCGCTGAGGCAGCACGGAGTCGGAGTTTTAAGGATATGGCAGCTGATGAACTCGATGTGGATGGTGCGCTGGATTTGCTAAAAG
CTGTGCAATCAAAGCCGGAAACCACAGACATGACTCC
>PhRXLR22_703 PLHAL7031639
TCATGTTGCAATTCGCGACTGCTTTCCTTGCAATCAGTGCAAACGTTGTCATGACGCAGCCAAATCAAGAATACATTGCTCCCGGGG
CTCGTGTCAGCTCTAATTACCGCGACATGACCATCCGCCGCTTACGTACTCACGAAATTGGGACAGTCCCAGAAGAGAGGATGCCTA
TACACGAATTGGAAATTGAAGATCTAATCACCATTATCGCCTCTAAGGTGGTCCCCGAATTATCGCACTCTGAGTCAATGTCTCTAC
TAAGCGCTGAGGCAGCACGGAGTCGGAGTTTTAAGGATATGGCAGCTGATGAACTCGATGTGGATGGTGCGCTGGATTTGCTAAAAG
CTGTGCAATCAAAGCCGGAAACCACAGACATGACTCC
>PhRXLR22_710 PLHAL7101461
TCATGTTGCAATTCGCGACTGCTTTCCTTGCAATCAGTGCAAACGTTGTCATGACGCAGCCAAATCAAGAATACATTGCTCCCGGGG
CTCGTGTCAGCTCTAATTACCGCGACATGACCATCCGCCGCTTACGTACTCACGAAATTGGGACAGTCCCAGAAGAGAGGATGCCTA
TACACGAATTGGAAATTGAAGATCTAATCACCATTATCGCCTCTAAGGTGGTCCCCGAATTATCGCACTCTGAGTCAATGTCTCTAC
TAAGCGCTGAGGCAGCACGGAGTCGGAGTTTTAAGGATATGGCAGCTGATGAACTCGATGTGGATGGTGCGCTGGATTTGCTAAAAG
CTGTGCAATCAAAGCCGGAAACCACAGACATGACTCC
>PhRXLR22_304 PLHAL3041724
TCATGTTGCAATTCGCGACTGCTTTCCTTGCAATCAGTGCAAACGTTGTCATGACGCAGCCAAATCAAGAATACATTGCTCCCGGGG
CTCGTGTCAGCTCTAATTACCGCGACATGACCATCCGCCGCTTACGTACTCACGAAATTGGGACAGTCCCAGAAGAGAGGATGCCTA
TACACGAATTGGAAATTGAAGATCTAATCACCATTATCGCCTCTAAGGTGGTCCCCGAATTATCGCACTCTGAGTCAATGTCTCTAC
TAAGCGCTGAGGCAGCACGGAGTCGGAGTTTTAAGGATATGGCAGCTGATGAACTCGATGTGGATGGTGCGCTGGATTTGCTAAAAG
CTGTGCAATCAAAGCCGGAAACCACAGACATGACTCC
>PhRXLR22_700 PLHAL7001969
TCATGTTGCAATTCGCGACTGCTTTCCTTGCAATCAGTGCAAACGTTGTCATGACGCAGCCAAATCAAGAATACATTGCTCCCGGGG
CTCGTGTCAGCTCTAATTACCGCGACATGACCATCCGCCGCTTACGTACTCACGAAATTGGGACAGTCCCAGAAGAGAGGATGCCTA
TACACGAATTGGAAATTGAAGATCTAATCACCATTATCGCCTCTAAGGTGGTCCCCGAATTATCGCACTCTGAGTCAATGTCTCTAC
TAAGCGCTGAGGCAGCACGGAGTCGGAGTTTTAAGGATATGGCAGCTGATGAACTCGATGTGGATGGTGCGCTGGATTTGCTAAAAG
CTGTGCAATCAAAGCCGGAAACCACAGACATGACTCC
>PhRXLR22_730 PLHAL7301675
TCATGTTGCAATTCGCGACTGCTTTCCTTGCAATCAGTGCAAACGTTGTCATGACGCAGCCAAATCAAGAATACATTGCTCCCGGGG
CTCGTGTCAGCTCTAATTACCGCGACATGACCATCCGCCGCTTACGTACTCACGAAATTGGGACAGTCCCAGAAGAGAGGATGCCTA
TACACGAATTGGAAATTGAAGATCTAATCACCATTATCGCCTCTAAGGTGGTCCCCGAATTATCGCACTCTGAGTCAATGTCTCTAC
TAAGCGCTGAGGCAGCACGGAGTCGGAGTTTTAAGGATATGGCAGCTGATGAACTCGATGTGGATGGTGCGCTGGATTTGCTAAAAG
CTGTGCAATCAAAGCCGGAAACCACAGACATGACTCC

>PhRXLR24_100 PLHAL100A10037
TTTGAACCCCAAAATACGAGTCCATAAAGCGACTTTTTCTCGAGAACTAAGTTTAGGTGGGTGTGAAATCTTTTTCATGGGTACGGT
CGACAAGCGGAGTTCAAGCAAATTTTCGCTGAGCGGAGACAGAGCTTGACGAAACGCTACCCTCTCTTCGTCACCAATCACTTCCTT
TTGTTCTTGTGAATCTTGCGCCCTTAGTTGGCGATCGCTTGCATCCCGTTCATTGCTCTGAAGAGTTTGATTTGAGGTGGAGAGTTC
TGCAAAGACGTCGACGAGTAGAACCACAGCGTAAAGAAGATGCAGCACGAAGGAAAAATATGGTCTGCGCATAGTGGTTTAGTGTTG
TAGGATGAGTTGGTAAGACTGACCGAGCAGAAT
>PhRXLR24_334 PLHAL3340018
TTTGAACCCCAAAATACGAGTCCATAAAGCGACTTTTTCTCGAGAACTAAGTTTAGGTGGGTGTGAAATCTTTTTCATGGGTACGGT
CGACAAGCGGAGTTCAAGCAAATTTTCGCTGAGCGGAGACAGAGCTTGACGAAACGCTACCCTCTCTTCGTCACCAATCACTTCCTT
TTGTTCTTGTGAATCTTGCGCCCTTAGTTGGCGATCGCTTGCATCCCGTTCATTGCTCTGAAGAGTTTGATTTGAGGTGGAGAGTTC
TGCAAAGACGTCGACGAGTAGAACCACAGCGTAAAGAAGATGCAGCACGAAGGAAAAATATGGTCTGCGCATAGTGGTTTAGTGTTG
TAGGATGAGTTGGTAAGACTGACCGAGCAGAAT
>PhRXLR24_703 PLHAL7030805
TTTGAACCCCAAAATACGAGTCCATAAAGCGACTTTTTCTCGAGAACTAAGTTCTTTAGGTGGGTGTGAAATCTTTTTCATGGGTAC
GGTCGACAAGCGGAGTTCAAGCAAATTTTCGCTGAGCGGAGACAGAGCTTGACGAAACGCTACCCTCTCTTCGTCACCAATCACTTC
CTTTTGTTCTTGTGAATCTTGCGCCCTTAGTTGGCGATCGCTTGCATCCCGTTCATTGCTCTGAAGAGTTTGATTTGAGGTGGAGAG
TTCTGCAAAGACGTCGACGAGTAGAACCACAGCGTAAAGAAGATGCAGCACGAAGGAAAAATATGGTCTGCGCATAGTGGTTTAGTG
TTGTAGGATGAGTTGGTAAGACTGACCGAGCAGAAT
>PhRXLR24_710 PLHAL7100765
TTTGAACCCCAAAATACGAGTCCATAAAGCGACTTTTTCTCGAGAACTAAGTTCTTTAGGTGGGTGTGAAATCTTTTTCATGGGTAC
GGTCGACAAGCGGAGTTCAAGCAAATTTTCGCTGAGCGGAGACAGAGCTTGACGAAACGCTACCCTCTCTTCGTCACCAATCACTTC
CTTTTGTTCTTGTGAATCTTGCGCCCTTAGTTGGCGATCGCTTGCATCCCGTTCATTGCTCTGAAGAGTTTGATTTGAGGTGGAGAG
TTCTGCAAAGACGTCGACGAGTAGAACCACAGCGTAAAGAAGATGCAGCACGAAGGAAAAATATGGTCTGCGCATAGTGGTTTAGTG
TTGTAGGATGAGTTGGTAAGACTGACCGAGCAGAAT

>PhRXLR24_304 PLHAL3040428
TTTGAACCCCAAAATACGAGTCCATAAAGCGACTTTTTCTCGAGAACTAAGTTTAGGTGGGTGTGAAATCTTTTTCATGGGTACGGT
CGACAAGCGGAGTTCAAGCAAATTTTCGCTGAGCGGAGACAGAGCTTGACGAAACGCTACCCTCTCTTCGTCACCAATCACTTCCTT
TTGTTCTTGTGAATCTTGCGCCCTTAGTTGGCGATCGCTTGCATCCCGTTCATTGCTCTGAAGAGTTTGATTTGAGGTGGAGAGTTC
TGCAAAGACGTCGACGAGTAGAACCACAGCGTAAAGAAGATGCAGCACGAAGGAAAAATATGGTCTGCGCATAGTGGTTTAGTGTTG
TAGGATGAGTTGGTAAGACTGACCGAGCAGAAT
>PhRXLR24_700 PLHAL7000772
TTTGAACCCCAAAATACGAGTCCATAAAGCGACTTTTTCTCGAGAACTAAGTTCTTTAGGTGGGTGTGAAATCTTTTTCATGGGTAC
GGTCGACAAGCGGAGTTCAAGCAAATTTTCGCTGAGCGGAGACAGAGCTTGACGAAACGCTACCCTCTCTTCGTCACCAATCACTTC
CTTTTGTTCTTGTGAATCTTGCGCCCTTAGTTGGCGATCGCTTGCATCCCGTTCATTGCTCTGAAGAGTTTGATTTGAGGTGGAGAG
TTCTGCAAAGACGTCGACGAGTAGAACCACAGCGTAAAGAAGATGCAGCACGAAGGAAAAATATGGTCTGCGCATAGTGGTTTAGTG
TTGTAGGATGAGTTGGTAAGACTGACCGAGCAGAAT
>PhRXLR24_730 PLHAL7300813
TTTGAACCCCAAAATACGAGTCCATAAAGCGACTTTTTCTCGAGAACTAAGTTCTTTAGGTGGGTGTGAAATCTTTTTCATGGGTAC
GGTCGACAAGCGGAGTTCAAGCAAATTTTCGCTGAGCGGAGACAGAGCTTGACGAAACGCTACCCTCTCTTCGTCACCAATCACTTC
CTTTTGTTCTTGTGAATCTTGCGCCCTTAGTTGGCGATCGCTTGCATCCCGTTCATTGCTCTGAAGAGTTTGATTTGAGGTGGAGAG
TTCTGCAAAGACGTCGACGAGTAGAACCACAGCGTAAAGAAGATGCAGCACGAAGGAAAAATATGGTCTGCGCATAGTGGTTTAGTG
TTGTAGGATGAGTTGGTAAGACTGACCGAGCAGAAT

>PhRXLR31_100 PLHAL100A10522
AATAAAGTAGAAATATATTACTTCTTGTATCTTTCTACGATGAAAGGTCGCAATCTTAATGAGATCCACGTTATACCATCGCTATGA
CTGAAGATGACAATGAGAGAAGATTTTTGCGTCGGCTTAAAATGAAACCAACAAAGATACACGCGACTGCTTAACGAGCAAAGATAT
GGACATCAGTCTTGATTCACTTTTCTATTTCCCAAGAGCGTCTCAACACTTAAAAATGAAGATCATTGCGACAGCAACAATTCTTGG
CATGGCATTACTCCATGTTGCAACCCCAACGATTGCTGCCGTTGCCTTTCGTCCCGCACTCGAAGCAGACGAAACGAATAAGCAGCA
ACAAGATGCACAAGACGAGGAACGAGCACTTGCTGCTGAGAATCATGACGCAACAGCAAGTGGAGACTGGAATGCTCTTCTTGCGGA
TCGAGAGACGCGTGCTCTAGCGTATATTCTTGAGGCAGCAGATATTATCGATGAAGAAGATAACTATGAGGAAGAGGAGGACGACTA
TGAAGATGAAGATTTGCTTGCATCTGGTGGAAGACGCCGTGCACTAGCTGATGGTGGACGACGTCGAGCGCTAGCGAATGGTGGAAG
ACGTCGAGCGCTTGCTAATGGTGGAAGACGTCGAGCGCTTGCTAATGGTGGAAGACGTCGAGCTCTGGCTGACGGTGGAAGACGTCG
TGCTCTTGCTGATGGTGGAAGACGTCGAGCTCTTGCTGATGGTGGAAGACGTCGAGCTCTTGCTGATGATCATTAGCGATACAATGA
TAAGCTTCTGGAACTCGAACGTGGATGACCAAAGGGTCGGACGCAGTGCATATTCAACATTCGTTATAAATGATTTTTGTTATTTTG
TGTTTTTTATAAAAAACACAAATATAGCAATATTCTTAGCAGTCTTGTCAAAAATGATATAAGTTACTCGATAAGATTCTAATGACA
GGATAAATACAAAGTTCAAAAAT
>PhRXLR31_334 PLHAL3340691
AATAAAGTAGAAATATATTACTTCTTGTATCTTTCTACGATGAAAGGTCGCAATCTTAATGAGATCCACGTTATACCATCGCTATGA
CTGAAGATGACAATGAGAGAAGATCTTTGCGTCGGCTTAAAATGAAACCAACAAAGATACACGCGACTGCTTAACGAGCAAAGATAT
GGACATCAGTCTTGATTCACTTTTCTATTTCCCAAGAGCGTCTCAACACTTAAAAATGAAGATCATTGCGACAGCAACAATTCTTGG
CATGGCATTACTCCATGTTGCAACCCCAACGATTGCTGCCGTTGCCTTTCGTCCCGCACTCGAAGCAGACGAAACGAATAAGCAGCA
ACAAGATGCACAAGACGAGGAACGAGCACTTGCTGCTGAGAATCAAGACGCAACAGCAAGTGGAGACTGGAATGCTCTTCTTGCGGA
TCGAGAGACGCGTGCTCTAGCGTATATTCTTGAGGCAGCAGATATTATCGATGAAGAAGATAACTATGAGGAAGAGGAGGACGACTA
TGAAGATGAAGATTTGCTTGCATCTGGTGGAAGACGCCGTGCACTAGCTGATGGTGGACGACGCCGAGCGCTAGCGAATGGTGGACG
ACGTCGAGCGCTTGCTAATGGTGGAAGACGTCGAGCGCTTGCTAATGGTGGAAGACGTCGAGCTCTGGCTGACGGTGGAAGACGTCG
TGCTCTTGCTGATGGTGGAAGACGTCGAGCTCTTGCTGATGGTGGAAGACGTCGAGCTCTTGCTGATGGTGGAAGACGTCGAGCTCT
TGCTGATGATCATTAGCGATACAATGATAAGCTTCTGGAACTCGAACGTGGATGACCAAAGGGTCGGACGCAGTGCATATTCAACAT
TCGTTATAAATGATTTTTGTTATTTTGTGTTTTTTATAAAAAACACAAATATAGCAATATTCTTAGCAGTCTTGTCAAAAATGATAT
AAGTTACTCGATAAGATTCTAATGACAGGATAAATACAAAGTTCAAAAAT
>PhRXLR31_703 PLHAL7030845
AATAAAGTAGAAATATATTACTTCTTGTATCTTTCTACGATGAAAGGTCGCAATCTTAATGAGATCCACGTTATACCATCGCTATGA
CTGAAGATGACAATGAGAGAAGATCTTTGCGTCGGCTTAAAATGAAACCAACAAAGATACACGCGACTGCTTAACGAGCAAAGATAT
GGACATCAGTCTTGATTCACTTTTCTATTTCCCAAGAGCGTCTCAACACTTAAAAATGAAGATCATTGCGACAGCAACAATTCTTGG
CATGGCATTACTCCATGTTGCAACCCCAACGATTGCTGCCGTTGCCTTTCGTCCCGCACTCGAAGCAGACGAAACGAATAAGCAGCA
ACAAGATGCACAAGACGAGGAACGAGCACTTGCTGCTGAGAATCATGACGCAACAGCAAGTGGAGACTGGAATGCTCTTCTTGCGGA
TCGAGAGACGCGTGCTCTAGCGTATATTCTTGAGGCAGCAGATATTATCGATGAAGAAGATAACTATGAGGAAGAGGAGGACGACTA
TGAAGATGAAGATTTGCTTGCATCTGGTGGAAGACGCCGTGCACTAGCTGATGGTGGACGACGTCGAGCGCTAGCGAATGGTGGAAG
ACGTCGAGCGCTTGCTAATGGTGGAAGACGTCGAGCGCTTGCTAATGGTGGAAGACGTCGAGCTCTGGCTGACGGTGGAAGACGTCG
TGCTCTTGCTGATGGTGGAAGACGTCGAGCTCTTGCTGATGGTGGAAGACGTCGAGCTCTTGCTGATGATCATTAGCGATACAATGA
TAAGCTTCTGGAACTCGAACGTGGATGACCAAAGGGTCGGACGCAGTGCATATTCAACATTCGTTATAAATGATTTTTGTTATTTTG
TGTTTTTTATAAAAAACACAAATATAGCAATATTCTTAGCAGTCTTGTCAAAAATGATATAAGTTACTCGATAAGATTCTAATGACA
GGATAAATACAAAGTTCAAAAAT
>PhRXLR31_710 PLHAL7100553
AATAAAGTAGAAATATATTACTTCTTGTATCTTTCTACGATGAAAGGTCGCAATCTTAATGAGATCCACGTTATACCATCGCTATGA
CTGAAGATGACAATGAGAGAAGATCTTTGCGTCGGCTTAAAATGAAACCAACAAAGATACACGCGACTGCTTAACGAGCAAAGATAT
GGACATCAGTCTTGATTCACTTTTCTATTTCCCAAGAGCGTCTCAACACTTAAAAATGAAGATCATTGCGACAGCAACAATTCTTGG
CATGGCATTACTCCATGTTGCAACCCCAACGATTGCTGCCGTTGCCTTTCGTCCCGCACTCGAAGCAGACGAAACGAATAAGCAGCA
ACAAGATGCACAAGACGAGGAACGAGCACTTGCTGCTGAGAATCATGACGCAACAGCAAGTGGAGACTGGAATGCTCTTCTTGCGGA
TCGAGAGACGCGTGCTCTAGCGTATATTCTTGAGGCAGCAGATATTATCGATGAAGAAGATAACTATGAGGAAGAGGAGGACGACTA
TGAAGATGAAGATTTGCTTGCATCTGGTGGAAGACGCCGTGCACTAGCTGATGGTGGACGACGTCGAGCGCTAGCGAATGGTGGAAG
ACGTCGAGCGCTTGCTAATGGTGGAAGACGTCGAGCGCTTGCTAATGGTGGAAGACGTCGAGCTCTGGCTGACGGTGGAAGACGTCG

TGCTCTTGCTGATGGTGGAAGACGTCGAGCTCTTGCTGATGGTGGAAGACGTCGAGCTCTTGCTGATGATCATTAGCGATACAATGA
TAAGCTTCTGGAACTCGAACGTGGATGACCAAAGGGTCGGACGCAGTGCATATTCAACATTCGTTATAAATGATTTTTGTTATTTTG
TGTTTTTTATAAAAAACACAAATATAGCAATATTCTTAGCAGTCTTGTCAAAAATGATATAAGTTACTCGATAAGATTCTAATGACA
GGATAAATACAAAGTTCAAAAAT
>PhRXLR31_304 PLHAL3040833
AATAAAGTAGAAATATATTACTTCTTGTATCTTTCTACGATGAAAGGTCGCAATCTTAATGAGATCCACGTTATACCATCGCTATGA
CTGAAGATGACAATGAGAGAAGATTTTTGCGTCGGCTTAAAATGAAACCAACAAAGATACACGCGACTGCTTAACGAGCAAAGATAT
GGACATCAGTCTTGATTCACTTTTCTATTTCCCAAGAGCGTCTCAACACTTAAAAATGAAGATCATTGCGACAGCAACAATTCTTGG
CATGGCATTACTCCATGTTGCAACCCCAACGATTGCTGCCGTTGCCTTTCGTCCCGCACTCGAAGCAGACGAAACGAATAAGCAGCA
ACAAGATGCACAAGACGAGGAACGAGCACTTGCTGCTGAGAATCATGACGCAACAGCAAGTGGAGACTGGAATGCTCTTCTTGCGGA
TCGAGAGACGCGTGCTCTAGCGTATATTCTTGAGGCAGCAGATATTATCGATGAAGAAGATAACTATGAGGAAGAGGAGGACGACTA
TGAAGATGAAGATTTGCTTGCATCTGGTGGAAGACGCCGTGCACTAGCTGATGGTGGACGACGTCGAGCGCTAGCGAATGGTGGAAG
ACGTCGAGCGCTTGCTAATGGTGGAAGACGTCGAGCGCTTGCTAATGGTGGAAGACGTCGAGCTCTGGCTGACGGTGGAAGACGTCG
TGCTCTTGCTGATGGTGGAAGACGTCGAGCTCTTGCTGATGGTGGAAGACGTCGAGCTCTTGCTGATGATCATTAGCGATACAATGA
TAAGCTTCTGGAACTCGAACGTGGATGACCAAAGGGTCGGACGCAGTGCATATTCAACATTCGTTATAAATGATTTTTGTTATTTTG
TGTTTTTTATAAAAAACACAAATATAGCAATATTCTTAGCAGTCTTGTCAAAAATGATATAAGTTACTCGATAAGATTCTAATGACA
GGATAAATACAAAGTTCAAAAAT
>PhRXLR31_700 PLHAL7000849
AATAAAGTAGAAATATATTACTTCTTGTATCTTTCTACGATGAAAGGTCGCAATCTTAATGAGATCCACGTTATACCATCGCTATGA
CTGAAGATGACAATGAGAGAAGATCTTTGCGTCGGCTTAAAATGAAACCAACAAAGATACACGCGACTGCTTAACGAGCAAAGATAT
GGACATCAGTCTTGATTCACTTTTCTATTTCCCAAGAGCGTCTCAACACTTAAAAATGAAGATCATTGCGACAGCAACAATTCTTGG
CATGGCATTACTCCATGTTGCAACCCCAACGATTGCTGCCGTTGCCTTTCGTCCCGCACTCGAAGCAGACGAAACGAATAAGCAGCA
ACAAGATGCACAAGACGAGGAACGAGCACTTGCTGCTGAGAATCATGACGCAACAGCAAGTGGAGACTGGAATGCTCTTCTTGCGGA
TCGAGAGACGCGTGCTCTAGCGTATATTCTTGAGGCAGCAGATATTATCGATGAAGAAGATAACTATGAGGAAGAGGAGGACGACTA
TGAAGATGAAGATTTGCTTGCATCTGGTGGAAGACGCCGTGCACTAGCTGATGGTGGACGACGTCGAGCGCTAGCGAATGGTGGAAG
ACGTCGAGCGCTTGCTAATGGTGGAAGACGTCGAGCGCTTGCTAATGGTGGAAGACGTCGAGCTCTGGCTGACGGTGGAAGACGTCG
TGCTCTTGCTGATGGTGGAAGACGTCGAGCTCTTGCTGATGGTGGAAGACGTCGAGCTCTTGCTGATGATCATTAGCGATACAATGA
TAAGCTTCTGGAACTCGAACGTGGATGACCAAAGGGTCGGACGCAGTGCATATTCAACATTCGTTATAAATGATTTTTGTTATTTTG
TGTTTTTTATAAAAAACACAAATATAGCAATATTCTTAGCAGTCTTGTCAAAAATGATATAAGTTACTCGATAAGATTCTAATGACA
GGATAAATACAAAGTTCAAAAAT
>PhRXLR31_730 PLHAL7300805
AATAAAGTAGAAATATATTACTTCTTGTATCTTTCTACGATGAAAGGTCGCAATCTTAATGAGATCCACGTTATACCATCGCTATGA
CTGAAGATGACAATGAGAGAAGATTTTTGCGTCGGCTTAAAATGAAACCAACAAAGATACACGCGACTGCTTAACGAGCAAAGATAT
GGACATCAGTCTTGATTCACTTTTCTATTTCCCAAGAGCGTCTCAACACTTAAAAATGAAGATCATTGCGACAGCAACAATTCTTGG
CATGGCATTACTCCATGTTGCAACCCCAACGATTGCTGCCGTTGCCTTTCGTCCCGCACTCGAAGCAGACGAAACGAATAAGCAGCA
ACAAGATGCACAAGACGAGGAACGAGCACTTGCTGCTGAGAATCATGACGCAACAGCAAGTGGAGACTGGAATGCTCTTCTTGCGGA
TCGAGAGACGCGTGCTCTAGCGTATATTCTTGAGGCAGCAGATATTATCGATGAAGAAGATAACTATGAGGAAGAGGAGGACGACTA
TGAAGATGAAGATTTGCTTGCATCTGGTGGAAGACGCCGTGCACTAGCTGATGGTGGACGACGTCGAGCGCTAGCGAATGGTGGAAG
ACGTCGAGCGCTTGCTAATGGTGGAAGACGTCGAGCGCTTGCTAATGGTGGAAGACGTCGAGCTCTGGCTGACGGTGGAAGACGTCG
TGCTCTTGCTGATGGTGGAAGACGTCGAGCTCTTGCTGATGGTGGAAGACGTCGAGCTCTTGCTGATGATCATTAGCGATACAATGA
TAAGCTTCTGGAACTCGAACGTGGATGACCAAAGGGTCGGACGCAGTGCATATTCAACATTCGTTATAAATGATTTTTGTTATTTTG
TGTTTTTTATAAAAAACACAAATATAGCAATATTCTTAGCAGTCTTGTCAAAAATGATATAAGTTACTCGATAAGATTCTAATGACA
GGATAAATACAAAGTTCAAAAAT

>PhRXLR32_100 PLHAL100A10020
AAGAAAACATTTGTCCATCCAGACAACTCTCCCTCATCAAATCCTGTAAGATAAGCATATGGTTTCTAGCATCAAGGTAAAGTCTGG
GCGAAGTATGATCCCGCTGCTGTCAGTCAGGTTCACTTCGCTTCTTGTCGTTTGCAAACCACACACCCTCAAGTTGTTCGACATGCG
TTACATTCTTCCACTGTTGATCGTTGCAGTCTTTGCGGTCTTAAGTGGCACAGCCTCGTCAACAGCTTTGAAGGAAGCCAAAAATGA
TATCAATGACAACAATGACAAGTCCACTGCGCTGACCGCGCCTCAAAGCGCAATCGTTACCACGAGGCAATTGCAATCTCCGACAGC
CGGCGAAGTTGAGGAAGAACGAGCCTGGCCAATCATTCGTCAAGCTCAGAGCTATCTGAAATCATTCTATGAATGGATTAAACATTT
CTTTAAAGCAATGTTTTTAGTCGAAACGCCAGCGTCGAGAAAATGGTACCATTTCCGCCAACATCTTAGGAGCATGAGGAGAGGCAG
TCGAAGAATGTACTATGACACAATGGGGAGGATTAAATCATGGTTCACGAGAGCGGGTCATTCCATATCAGAGGATTCCCAGAAAGC
CAAGCACTCAGTAAGCGAAGGCCTCCAAAAGGGATCGCATCGGGTTGGTGAAAAGAGTGAGTTATTGAGACATTCAGTGACAGAGCA
AGCTCAAAAGGCAGGACACATGGCCGATAGGGGAGGTAAGCGATTTTGGAATTTCATCAAGACAGGTCATGCTTAACCGATAGGGGA
GGTAAGCGATTTTGGAATTTCATTAAGACAGGTCATGCTTAAAGCCATGGCACTGGTGTATTGATTCCCATTAGCAACCAACGTTGT
TTCGATCATTTTAGTACATTCATCTTTCTATTCCATACAATTTTAGACTTGAAAATGCATGGTGTAGCTATTGTT
>PhRXLR32_334 PLHAL3340020
AAGAAAACATTTGTCCATCCAGACAACTCTCCCTCATCAAATCCTGTAAGATAAGCATATGGTTTCTAGCATCAAGGTAAAGTCTGG
GCGAAGTATGATCCCGCTGCTGTCAGTCAGGTTCACTTCGCTTCTTGTCGTTTGCAAACCACACACCCTCAAGTTGTTCGACATGCG
TTACATTCTTCCACTGTTGATCGTTGCAGTCTTTGCGGTCTTAAGTGGCACAGCCTCGTCAACAGCTTTGAAGGAAGCCAAAAATGA
TATCAATGACAACAATGACAAGTCCACTGCGCTGACCGCGCCTCAAAGCGCAATCGTTACCACGAGGCAATTGCAATCTCCGACAGC
CGGCGAAGTTGAGGAAGAACGAGCCTGGCCAATCATTCGTCAAGCTCAGAGCTATCTGAAATCATTCTATGAATGGATTAAACATTT
CTTTAAAGCAATGTTTTTAGTCGAAACGCCAGCGTCGAGAAAATGGTACCATTTCCGCCAACATCTTAGGAGCATGAGGAGAGGCAG
TCGAAGAATGTACTATGACACAATGGGGAGGATTAAATCATGGTTCACGAGAGCGGGTCATTCCATATCAGAGGATTCCCAGAAAGC
CAAGCACTCAGTAAGCGAAGGCCTCCAAAAGGGATCGCATCGGGTTGGTGAAAAGTTTGAGTCATTGAGACATTCAGTGACAGAGCA
AGCTCAAAAGGCAGGACACATGGCCGATAGGGGATCTAAGCGAACTTGGAATTTCATGAAGACAGGTCATGCTTAAAGCCATGGCAC

TGGTGTATTGATTCCCATTAGCAACCAACGTTGTTTCGATCATTTTAGTACATTCATCTTTCTATTCCATACAATTTTAAACTTGAA
AATGCATGGTGTAGCTATTGTT
>PhRXLR32_703 PLHAL7030020
AAGAAAACATTTGTCCATCCAGACAACTCTCCCTCATCAAATCCTGTAAGATAAGCATATGGTTTCTAGCATCAAGGTAAAGTCTGG
GCGAAGTATGATCCCGCTGCTGTCAGTCAGGTTCACTTCGCTTCTTGTCGTTTGCAAACCACACACCCTCAAGTTGTTCGACATGCG
TTACATTCTTCCACTGTTGATCGTTGCAGTCTTTGCGGTCTTAAGTGGCACAGCCTCGTCAACAGCTTTGAAGGAAGCCAAAAATGA
TATCAATGACAACAATGACAAGTCCACTGCGCTGACCGCGCCTCAAAGCGCAATCGTTACCACGAGGCAATTGCAATCTCCGACAGC
CGGCGAAGTTGAGGAAGAACGAGCCTGGCCAATCATTCGTCAAGCTCAGAGCTATCTGAAATCATTCTATGAATGGATTAAACATTT
CTTTAAAGCAATGTTTTTAGTCGAAACGCCAGCGTCGAGAAAATGGTACCATTTCCGCCAACATCTTAGGAGCATGAGGAGAGGCAG
TCGAAGAATGTACTATGACACAATGGGGAGGATTAAATCATGGTTCACGAGAGCGGGTCATTCCATATCAGAGGATTCCCAGAAAGC
CAAGCACTCAGTAAGCGAAGGCCTCCAAAAGGGATCGCATCGGGTTGGTGAAAAGTTTGAGTCATTGAGACATTCAGTGACAGAGCA
AGCTCAAAAGGCAGGACACATGGCCGATAGGGGATCTAAGCGAACTTGGAATTTCATGAAGACAGGTCATGCTTAAAGCCATGGCAC
TGGTGTATTGATTCCCATTAGCAACCAACGTTGTTTCGATCATTTTAGTACATTCATCTTTCTATTCCATACAATTTTAAACTTGAA
AATGCATGGTGTAGCTATTGTT
>PhRXLR32_710 PLHAL7100062
AAGAAAACATTTGTCCATCCAGACAACTCTCCCTCATCAAATCCTGTAAGATAAGCATATGGTTTCTAGCATCAAGGTAAAGTCTGG
GCGAAGTATGATCCCGCTGCTGTCAGTCAGGTTCACTTCGCTTCTTGTCGTTTGCAAACCACACACCCTCAAGTTGTTCGACATGCG
TTACATTCTTCCACTGTTGATCGTTGCAGTCTTTGCGGTCTTAAGTGGCACAGCCTCGTCAACAGCTTTGAAGGAAGCCAAAAATGA
TATCAATGACAACAATGACAAGTCCACTGCGCTGACCGCGCCTCAAAGCGCAATCGTTACCACGAGGCAATTGCAATCTCCGACAGC
CGGCGAAGTTGAGGAAGAACGAGCCTGGCCAATCATTCGTCAAGCTCAGAGCTATCTGAAATCATTCTATGAATGGATTAAACATTT
CTTTAAAGCAATGTTTTTAGTCGAAACGCCAGCGTCGAGAAAATGGTACCATTTCCGCCAACATCTTAGGAGCATGAGGAGAGGCAG
TCGAAGAATGTACTATGACACAATGGGGAGGATTAAATCATGGTTCACGAGAGCGGGTCATTCCATATCAGAGGATTCCCAGAAAGC
CAAGCACTCAGTAAGCGAAGGCCTCCAAAAGGGATCGCATCGGGTTGGTGAAAAGTTTGAGTCATTGAGACATTCAGTGACAGAGCA
AGCTCAAAAGGCAGGACACATGGCCGATAGGGGATCTAAGCGAACTTGGAATTTCATGAAGACAGGTCATGCTTAAAGCCATGGCAC
TGGTGTATTGATTCCCATTAGCAACCAACGTTGTTTCGATCATTTTAGTACATTCATCTTTCTATTCCATACAATTTTAAACTTGAA
AATGCATGGTGTAGCTATTGTT
>PhRXLR32_304 PLHAL3040022
AAGAAAACATTTGTCCATCCAGACAACTCTCCCTCATCAAATCCTGTAAGATAAGCATATGGTTTCTAGCATCAAGGTAAAGTCTGG
GCGAAGTATGATCCCGCTGCTGTCAGTCAGGTTCACTTCGCTTCTTGTCGTTTGCAAACCACACACCCTCAAGTTGTTCGACATGCG
TTACATTCTTCCACTGTTGATCGTTGCAGTCTTTGCGGTCTTAAGTGGCACAGCCTCGTCAACAGCTTTGAAGGAAGCCAAAAATGA
TATCAATGACAACAATGACAAGTCCACTGCGCTGACCGCGCCTCAAAGCGCAATCGTTACCACGAGGCAATTGCAATCTCCGACAGC
CGGCGAAGTTGAGGAAGAACGAGCCTGGCCAATCATTCGTCAAGCTCAGAGCTATCTGAAATCATTCTATGAATGGATTAAACATTT
CTTTAAAGCAATGTTTTTAGTCGAAACGCCAGCGTCGAGAAAATGGTACCATTTCCGCCAACATCTTAGGAGCATGAGGAGAGGCAG
TCGAAGAATGTACTATGACACAATGGGGAGGATTAAATCATGGTTCACGAGAGCGGGTCATTCCATATCAGAGGATTCCCAGAAAGC
CAAGCACTCAGTAAGCGAAGGCCTCCAAAAGGGATCGCATCGGGTTGGTGAAAAGAGTGAGTTATTGAGACATTCAGTGACAGAGCA
AGCTCAAAAGGCAGGACACATGGCCGATAGGGGAGGTAAGCGATTTTGGAATTTCATCAAGACAGGTCATGCTTAACCGATAGGGGA
GGTAAGCGATTTTGGAATTTCATTAAGACAGGTCATGCTTAAAGCCATGGCACTGGTGTATTGATTCCCATTAGCAACCAACGTTGT
TTCGATCATTTTAGTACATTCATCTTTCTATTCCATACAATTTTAGACTTGAAAATGCATGGTGTAGCTATTGTT
>PhRXLR32_700 PLHAL7000053
AAGAAAACATTTGTCCATCCAGACAACTCTCCCTCATCAAATCCTGTAAGATAAGCATATGGTTTCTAGCATCAAGGTAAAGTCTGG
GCGAAGTATGATCCCGCTGCTGTCAGTCAGGTTCACTTCGCTTCTTGTCGTTTGCAAACCACACACCCTCAAGTTGTTCGACATGCG
TTACATTCTTCCACTGTTGATCGTTGCAGTCTTTGCGGTCTTAAGTGGCACAGCCTCGTCAACAGCTTTGAAGGAAGCCAAAAATGA
TATCAATGACAACAATGACAAGTCCACTGCGCTGACCGCGCCTCAAAGCGCAATCGTTACCACGAGGCAATTGCAATCTCCGACAGC
CGGCGAAGTTGAGGAAGAACGAGCCTGGCCAATCATTCGTCAAGCTCAGAGCTATCTGAAATCATTCTATGAATGGATTAAACATTT
CTTTAAAGCAATGTTTTTAGTCGAAACGCCAGCGTCGAGAAAATGGTACCATTTCCGCCAACATCTTAGGAGCATGAGGAGAGGCAG
TCGAAGAATGTACTATGACACAATGGGGAGGATTAAATCATGGTTCACGAGAGCGGGTCATTCCATATCAGAGGATTCCCAGAAAGC
CAAGCACTCAGTAAGCGAAGGCCTCCAAAAGGGATCGCATCGGGTTGGTGAAAAGTTTGAGTCATTGAGACATTCAGTGACAGAGCA
AGCTCAAAAGGCAGGACACATGGCCGATAGGGGATCTAAGCGAACTTGGAATTTCATGAAGACAGGTCATGCTTAAAGCCATGGCAC
TGGTGTATTGATTCCCATTAGCAACCAACGTTGTTTCGATCATTTTAGTACATTCATCTTTCTATTCCATACAATTTTAAACTTGAA
AATGCATGGTGTAGCTATTGTT
>PhRXLR32_730 PLHAL7300003
AAGAAAACATTTGTCCATCCAGACAACTCTCCCTCATCAAATCCTGTAAGATAAGCATATGGTTTCTAGCATCAAGGTAAAGTCTGG
GCGAAGTATGATCCCGCTGCTGTCAGTCAGGTTCACTTCGCTTCTTGTCGTTTGCAAACCACACACCCTCAAGTTGTTCGACATGCG
TTACATTCTTCCACTGTTGATCGTTGCAGTCTTTGCGGTCTTAAGTGGCACAGCCTCGTCAACAGCTTTGAAGGAAGCCAAAAATGA
TATCAATGACAACAATGACAAGTCCACTGCGCTGACCGCGCCTCAAAGCGCAATCGTTACCACGAGGCAATTGCAATCTCCGACAGC
CGGCGAAGTTGAGGAAGAACGAGCCTGGCCAATCATTCGTCAAGCTCAGAGCTATCTGAAATCATTCTATGAATGGATTAAACATTT
CTTTAAAGCAATGTTTTTAGTCGAAACGCCAGCGTCGAGAAAATGGTACCATTTCCGCCAACATCTTAGGAGCATGAGGAGAGGCAG
TCGAAGAATGTACTATGACACAATGGGGAGGATTAAATCATGGTTCACGAGAGCGGGTCATTCCATATCAGAGGATTCCCAGAAAGC
CAAGCACTCAGTAAGCGAAGGCCTCCAAAAGGGATCGCATCGGGTTGGTGAAAAGTTTGAGTCATTGAGACATTCAGTGACAGAGCA
AGCTCAAAAGGCAGGACACATGGCCGATAGGGGATCTAAGCGAACTTGGAATTTCATGAAGACAGGTCATGCTTAAAGCCATGGCAC
TGGTGTATTGATTCCCATTAGCAACCAACGTTGTTTCGATCATTTTAGTACATTCATCTTTCTATTCCATACAATTTTAAACTTGAA
AATGCATGGTGTAGCTATTGTT

>PhRXLR33_100 PLHAL100A10225
CTTTCAATTAAGAGATGATCATGCGTCATCTCTCTCTTCTTCTCTTGCTTTTCTTCTCTTATGCAGCATGTCTTCATGGTCTTCACA
ACAATGACATTGCTACTCAAAGTATCGATTCAGAATCTCACATGAGAACTAGTGACCAAGATAAAAAAGAAAACAGTTTACGATCAA
GTATCAAGACAAAGGACGAAGAACGACTCAGTTTTCCATTTTTCTGGAAAGGGCCTCGTTCGACAGCTACGACTGAAATTAGTGCAG

AAGCCGCTAGAGCAGCTACGACTGAATTTAGTGCAGTAGCCACTAGAGAAGCTACAAACGCTGAGAAAGAAATCATTGACGGTGCCA
AAGTTGTCGATGTGAAGAAAGTTGGTCGACTCCGACATATATTGAATCAGATTAAGAAGATTCCGGTGAAAGGAGATGTTCTGTATA
TGCTGATAATTTATGGAATTCTCTTCGTCAGTACGATTCTTATTATCTTAGCGGGAGTAGCGATCAATCATCACGTCCAGAGTCAGT
ATATTCACGATCAATAGGTGAAATTAGGCTGAATTTCAAACTCATTTTCCATTGTAAAAATTCGGATAATATCAGACTATTAGATTT
GTTTT
>PhRXLR33_334 PLHAL3340055
CTTTCAATTAAGAGATGATCATGCGTCATCTCTCTCTTCTTCTCTTGCTTTTCTTCTCTTATGCAGCATGTCTTCATGGTCTTCACA
ACAATGACATTGCTACTCAAAGTATCGATTCAGAATCTCACATGAGAACTAGTGACCAAGATAAAAAAGAAAACAGTTTACGATCAA
GTATCAAGACAAAGGACGAAGAACGACTCAGTTTTCCATTTTTCTGGAAAGGGCCTCGTTCGACAGCTACGACTGAAATTAGTGCAG
AAGCCGCTAGAGCAGCTACGACTGAATTTAGTGCAGTAGCCACTAGAGAAGCTACAAACGCTGAGAAAGAAATCATTGACGGTGCCA
AAGTTGTCGATGTGAAGAAAGTTGGTCGACTCCGACATATATTGAATCAGATTAAGAAGATTCCGGTGAAAGGAGATGTTCTGTATA
TGCTGATAATTTATGGAATTCTCTTCGTCAGTACGATTCTTATTATCTTAGCGGGAGTAGCGATCAATCATCACGTCCAGAGTCAGT
ATATTCACGATCAATAGGTGAAATTAGGCTGAATTTCAAACTCATTTTCCATTGTAAAAATTCGGATAATATCAGACTATTAGATTT
GTTTT
>PhRXLR33_703 PLHAL7030057
CTTTCAATTAAGAGATGATCATGCGTCATCTCTCTCTTCTTCTCTTGCTTTTCTTCTCTTATGCAGCATGTCTTCATGGTCTTCACA
ACAATGACATTGCTACTCAAAGTATCGATTCAGAATCTCACATGAGAACTAGTGACCAAGATAAAAAAGAAAACAGTTTACGATCAA
GTATCAAGACAAAGGACGAAGAACGACTCAGTTTTCCATTTTTCTGGAAAGGGCCTCGTTCGACAGCTACGACTGAAATTAGTGCAG
AAGCCGCTAGAGCAGCTACGACTGAATTTAGTGCAGTAGCCACTAGAGAAGCTACAAACGCTGAGAAAGAAATCATTGACGGTGCCA
AAGTTGTCGATGTGAAGAAAGTTGGTCGACTCCGACATATATTGAATCAGATTAAGAAGATTCCGGTGAAAGGAGATGTTCTGTATA
TGCTGATTATTTATGGAATTCTCTTCGTCAGTACGATTCTTATTATCTTAGCGGGAGTAGCGATCAATCATCACGTCCAGAGTCAGT
ATATTCACGATCAATAGGTGAAATTAGGCTGAATTTCAAACTCATTTTCCATTGTAAAAATTCGGATAATATCAGACTATTAGATTT
GTTTT
>PhRXLR33_710 PLHAL7100087
CTTTCAATTAAGAGATGATCATGCGTCATCTCTCTCTTCTTCTCTTGCTTTTCTTCTCTTATGCAGCATGTCTTCATGGTCTTCACA
ACAATGACATTGCTACTCAAAGTATCGATTCAGAATCTCACATGAGAACTAGTGACCAAGATAAAAAAGAAAACAGTTTACGATCAA
GTATCAAGACAAAGGACGAAGAACGACTCAGTTTTCCATTTTTCTGGAAAGGGCCTCGTTCGACAGCTACGACTGAAATTAGTGCAG
AAGCCGCTAGAGCAGCTACGACTGAATTTAGTGCAGTAGCCACTAGAGAAGCTACAAACGCTGAGAAAGAAATCATTGACGGTGCCA
AAGTTGTCGATGTGAAGAAAGTTGGTCGACTCCGACATATATTGAATCAGATTAAGAAGATTCCGGTGAAAGGAGATGTTCTGTATA
TGCTGATTATTTATGGAATTCTCTTCGTCAGTACGATTCTTATTATCTTAGCGGGAGTAGCGATCAATCATCACGTCCAGAGTCAGT
ATATTCACGATCAATAGGTGAAATTAGGCTGAATTTCAAACTCATTTTCCATTGTAAAAATTCGGATAATATCAGACTATTAGATTT
GTTTT
>PhRXLR33_304 PLHAL3040047
CTTTCAATTAAGAGATGATCATGCGTCATCTCTCTCTTCTTCTCTTGCTTTTCTTCTCTTATGCAGCATGTCTTCATGGTCTTCACA
ACAATGACATTGCTACTCAAAGTATCGATTCAGAATCTCACATGAGAACTAGTGACCAAGATAAAAAAGAAAACAGTTTACGATCAA
GTATCAAGACAAAGGACGAAGAACGACTCAGTTTTCCATTTTTCTGGAAAGGGCCTCGTTCGACAGCTACGACTGAAATTAGTGCAG
AAGCCGCTAGAGCAGCTACGACTGAATTTAGTGCAGTAGCCACTAGAGAAGCTACAAACGCTGAGAAAGAAATCATTGACGGTGCCA
AAGTTGTCGATGTGAAGAAAGTTGGTCGACTCCGACATATATTGAATCAGATTAAGAAGATTCCGGTGAAAGGAGATGTTCTGTATA
TGCTGATAATTTATGGAATTCTCTTCGTCAGTACGATTCTTATTATCTTAGCGGGAGTAGCGATCAATCATCACGTCCAGAGTCAGT
ATATTCACGATCAATAGGTGAAATTAGGCTGAATTTCAAACTCATTTTCCATTGTAAAAATTCGGATAATATCAGACTATTAGATTT
GTTTT
>PhRXLR33_700 PLHAL7000245
CTTTCAATTAAGAGATGATCATGCGTCATCTCTCTCTTCTTCTCTTGCTTTTCTTCTCTTATGCAGCATGTCTTCATGGTCTTCACA
ACAATGACATTGCTACTCAAAGTATCGATTCAGAATCTCACATGAGAACTAGTGACCAAGATAAAAAAGAAAACAGTTTACGATCAA
GTATCAAGACAAAGGACGAAGAACGACTCAGTTTTCCATTTTTCTGGAAAGGGCCTCGTTCGACAGCTACGACTGAAATTAGTGCAG
AAGCCGCTAGAGCAGCTACGACTGAATTTAGTGCAGTAGCCACTAGAGAAGCTACAAACGCTGAGAAAGAAATCATTGACGGTGCCA
AAGTTGTCGATGTGAAGAAAGTTGGTCGACTCCGACATATATTGAATCAGATTAAGAAGATTCCGGTGAAAGGAGATGTTCTGTATA
TGCTGATTATTTATGGAATTCTCTTCGTCAGTACGATTCTTATTATCTTAGCGGGAGTAGCGATCAATCATCACGTCCAGAGTCAGT
ATATTCACGATCAATAGGTGAAATTAGGCTGAATTTCAAACTCATTTTCCATTGTAAAAATTCGGATAATATCAGACTATTAGATTT
GTTTT
>PhRXLR33_730 PLHAL7300066
CTTTCAATTAAGAGATGATCATGCGTCATCTCTCTCTTCTTCTCTTGCTTTTCTTCTCTTATGCAGCATGTCTTCATGGTCTTCACA
ACAATGACATTGCTACTCAAAGTATCGATTCAGAATCTCACATGAGAACTAGTGACCAAGATAAAAAAGAAAACAGTTTACGATCAA
GTATCAAGACAAAGGACGAAGAACGACTCAGTTTTCCATTTTTCTGGAAAGGGCCTCGTTCGACAGCTACGACTGAAATTAGTGCAG
AAGCCGCTAGAGCAGCTACGACTGAATTTAGTGCAGTAGCCACTAGAGAAGCTACAAACGCTGAGAAAGAAATCATTGACGGTGCCA
AAGTTGTCGATGTGAAGAAAGTTGGTCGACTCCGACATATATTGAATCAGATTAAGAAGATTCCGGTGAAAGGAGATGTTCTGTATA
TGCTGATAATTTATGGAATTCTCTTCGTCAGTACGATTCTTATTATCTTAGCGGGAGTAGCGATCAATCATCACGTCCAGAGTCAGT
ATATTCACGATCAATAGGTGAAATTAGGCTGAATTTCAAACTCATTTTCCATTGTAAAAATTCGGATAATATCAGACTATTAGATTT
GTTTT

>PhRXLR34_100 PLHAL100A10027
AAGGTCAAACTTACGGCAGACATTGAGAGCCGCTTGCAAAAATTACTTCTTTCTCCTCCTCTCGTGGGTGGGTTGGAGGTTATACTG
TCTGTATATATGTCAGACTGTCACGAAAAAAACAATTGCTTCTCATATCTGAAATATTCGAGCAACACAAAGAACTAAAGAGTAAAT
GAGAGTTCAATACGCAGTACAACTAGCTATTGCCGTCGTTGTAATGTGCGTCGACGCTAAGACCTCAATAACAAGCGCCAAGATAAT
CAGACAGTCGAATTCTTCGTCATCAGCTCTTCCAACTGCTGACGATAACGGCAGCACCTTTGAACTTGTCCAAAGTGACGAAGAGAG
GTTTTGGCTAATTCATTATCCGTCGGATCTACTGATGTCAAGCAATTGGTTCATCAATTCGGTATCTAAGTTTTTCAAGATGATTCA
TTACCCACCGAATAGCCTGTTGAAATCCTGCTTACCTCAATTCACAAGTTGGGAAAATTGGATTTCATTAACTTCCGTCAATTCACA

AGTTGGGATAGAATGACCGTTTACAAGTTCAATTTTGTATATGAGTATTACAAAACGACGCGTGGAAATGCGGCATCAAGAGAAATG
TACGAAGCACTTTCTCAACAATCCGATGGTCCGTCCTTGCTTGCAATATTGCTCCATAATGGACAGGACGAGCCACTCATCAAGGAC
AGTTGCAAATCTGTCGAACTAGCGCAATTTTCGCAATGGGCTGAAGATGATAGTAAAAAGCTCGAAAACATTTTCAATTTCTTGGCT
AACGATTATAATTTGATTCGGGAAAGAAAACATGAAATGATGATTTTGGCGGAGAGAGTGGATGTAATCAAAAACATCAAGCAGTCT
TATAGCGACTTTCTCA
>PhRXLR34_334 PLHAL3340045
AAGGTCAAACTTACGGCAGACATTGAGAGCCGCTTGCAAAAATTACTTCTTTCTCCTCCTCTCGTGGGTGGGTTGGAGGTTATACTG
TCTGTATATATGTCAGACTGTCACGAAAAAAACAATTGCTTCTCATATCTGAAATATTCGAGCAACACAAAGAACTAAAGAGTAAAT
GAGAGTTCAATACGCAGTACAACTAGCTATTGCCGTCGTTGTAATGTGCGTCGACGCTAAGACCTCAATAACAAGCGCCAAGATAAT
CAGACAGTCGAATTCTTCGTCATCAGCTCTTCCAACTGCTGACGATAACGGCAGCACCTTTGAACTTGTCCAAAGTGACGAAGAGAG
GTTTTGGCTAATTCATTATCCGTCGGATCTACTGATGTCAAGCAATTGGTTCATCAATTCGGTATCTAAGTTTTTCAAGATGATTCA
TTACCCACCGAATAGCCTGTTGAAATCCTGCTTACCTCAATTCACAAGTTGGGAAAATTGGATTTCATTAACTTCCGTCAATTCACA
AGTTGGGATAGAATGACCGTTTACAAGTTCAATTTTGTATATGAGTATTACAAAACGACGCGTGGAAATGCGGCATCAAGAGAAATG
TACGAAGCACTTTCTCAACAATCCGATGGTCCGTCCTTGCTTGCAATATTGCTCCATAATGGACAGGACGAGCCACTCATCAAGGAC
AGTTGCAAATCTGTCGAACTAGCGCAATTTTCGCAATGGGCTGAAGATGATAGTAAAAAGCTCGAAAACATTTTCAATTTCTTGGCT
AACGATTATAATTTGATTCGGGAAAGAAAACATGAAATGATGATTTTGGCGGAGAGAGTGGATGTAATCAAAAACATCAAGCAGTCT
TATAGCGACTTTCTCA
>PhRXLR34_703 PLHAL7030033
AAGGTCAAACTTACGGCAGACATTGAGAGCCGCTTGCAAAAATTACTTCTTTCTCCTCCTCTCGTGGGTGGGTTGGAGGTTATACTG
TCTGTATATATGTCAGACTGTCACGAAAAAAACAATTGCTTCTCATATCTGAAATATTCGAGCAACACAAAGAACTAAAGAGTAAAT
GAGAGTTCAATACGCAGTACAACTAGCTATTGCCGTCGTTGTAATGTGCGTCGACGCTAAGACCTCAATAACAAGCGCCAAGATAAT
CAGACAGTCGAATTCTTCGTCATCAGCTCTTCCAACTGCTGACGATAACGGCAGCACCTTTGAACTTGTCCAAAGTGACGAAGAGAG
GTTTTGGCTAATTCATTATCCGTCGGATCTACTGATGTCAAGCAATTGGTTCATCAATTCGGTATCTAAGTTTTTCAAGATGATTCA
TTACCCACCGAATAGCCTGTTGAAATCCTGCTTACCTCAATTCACAAGTTGGGAAAATTGGATTTCATTAACTTCCGTCAATTCACA
AGTTGGGATAGAATGACCGTTTACAAGTTCAATTTTGTATATGAGTATTACAAAACGACGCGTGGAAATGCGGCATCAAGAGAAATG
TACGAAGCACTTTCTCAACAATCCGATGGTCCGTCCTTGCTTGCAATATTGCTCCATAATGGACAGGACGAGCCACTCATCAAGGAC
AGTTGCAAATCTGTCGAACTAGCGCAATTTTCGCAATGGGCTGAAGATGATAGTAAAAAGCTCGAAAACATTTTCAATTTCTTGGCT
AACGATTATAATTTGATTCGGGAAAGAAAACATGAAATGATGATTTTGGCGGAGAGAGTGGATGTAATCAAAAACATCAAGCAGTCT
TATAGCGACTTTCTCA
>PhRXLR34_710 PLHAL7100064
AAGGTCAAACTTACGGCAGACATTGAGAGCCGCTTGCAAAAATTACTTCTTTCTCCTCCTCTCGTGGGTGGGTTGGAGGTTATACTG
TCTGTATATATGTCAGACTGTCACGAAAAAAACAATTGCTTCTCATATCTGAAATATTCGAGCAACACAAAGAACTAAAGAGTAAAT
GAGAGTTCAATACGCAGTACAACTAGCTATTGCCGTCGTTGTAATGTGCGTCGACGCTAAGACCTCAATAACAAGCGCCAAGATAAT
CAGACAGTCGAATTCTTCGTCATCAGCTCTTCCAACTGCTGACGATAACGGCAGCACCTTTGAACTTGTCCAAAGTGACGAAGAGAG
GTTTTGGCTAATTCATTATCCGTCGGATCTACTGATGTCAAGCAATTGGTTCATCAATTCGGTATCTAAGTTTTTCAAGATGATTCA
TTACCCACCGAATAGCCTGTTGAAATCCTGCTTACCTCAATTCACAAGTTGGGAAAATTGGATTTCATTAACTTCCGTCAATTCACA
AGTTGGGATAGAATGACCGTTTACAAGTTCAATTTTGTATATGAGTATTACAAAACGACGCGTGGAAATGCGGCATCAAGAGAAATG
TACGAAGCACTTTCTCAACAATCCGATGGTCCGTCCTTGCTTGCAATATTGCTCCATAATGGACAGGACGAGCCACTCATCAAGGAC
AGTTGCAAATCTGTCGAACTAGCGCAATTTTCGCAATGGGCTGAAGATGATAGTAAAAAGCTCGAAAACATTTTCAATTTCTTGGCT
AACGATTATAATTTGATTCGGGAAAGAAAACATGAAATGATGATTTTGGCGGAGAGAGTGGATGTAATCAAAAACATCAAGCAGTCT
TATAGCGACTTTCTCA
>PhRXLR34_304 PLHAL3040020
AAGGTCAAACTTACGGCAGACATTGAGAGCCGCTTGCAAAAATTACTTCTTTCTCCTCCTCTCGTGGGTGGGTTGGAGGTTATACTG
TCTGTATATATGTCAGACTGTCACGAAAAAAACAATTGCTTCTCATATCTGAAATATTCGAGCAACACAAAGAACTAAAGAGTAAAT
GAGAGTTCAATACGCAGTACAACTAGCTATTGCCGTCGTTGTAATGTGCGTCGACGCTAAGACCTCAATAACAAGCGCCAAGATAAT
CAGACAGTCGAATTCTTCGTCATCAGCTCTTCCAACTGCTGACGATAACGGCAGCACCTTTGAACTTGTCCAAAGTGACGAAGAGAG
GTTTTGGCTAATTCATTATCCGTCGGATCTACTGATGTCAAGCAATTGGTTCATCAATTCGGTATCTAAGTTTTTCAAGATGATTCA
TTACCCACCGAATAGCCTGTTGAAATCCTGCTTACCTCAATTCACAAGTTGGGAAAATTGGATTTCATTAACTTCCGTCAATTCACA
AGTTGGGATAGAATGACCGTTTACAAGTTCAATTTTGTATATGAGTATTACAAAACGACGCGTGGAAATGCGGCATCAAGAGAAATG
TACGAAGCACTTTCTCAACAATCCGATGGTCCGTCCTTGCTTGCAATATTGCTCCATAATGGACAGGACGAGCCACTCATCAAGGAC
AGTTGCAAATCTGTCGAACTAGCGCAATTTTCGCAATGGGCTGAAGATGATAGTAAAAAGCTCGAAAACATTTTCAATTTCTTGGCT
AACGATTATAATTTGATTCGGGAAAGAAAACATGAAATGATGATTTTGGCGGAGAGAGTGGATGTAATCAAAAACATCAAGCAGTCT
TATAGCGACTTTCTCA
>PhRXLR34_700 PLHAL7000363
AAGGTCAAACTTACGGCAGACATTGAGAGCCGCTTGCAAAAATTACTTCTTTCTCCTCCTCTCGTGGGTGGGTTGGAGGTTATACTG
TCTGTATATATGTCAGACTGTCACGAAAAAAACAATTGCTTCTCATATCTGAAATATTCGAGCAACACAAAGAACTAAAGAGTAAAT
GAGAGTTCAATACGCAGTACAACTAGCTATTGCCGTCGTTGTAATGTGCGTCGACGCTAAGACCTCAATAACAAGCGCCAAGATAAT
CAGACAGTCGAATTCTTCGTCATCAGCTCTTCCAACTGCTGACGATAACGGCAGCACCTTTGAACTTGTCCAAAGTGACGAAGAGAG
GTTTTGGCTAATTCATTATCCGTCGGATCTACTGATGTCAAGCAATTGGTTCATCAATTCGGTATCTAAGTTTTTCAAGATGATTCA
TTACCCACCGAATAGCCTGTTGAAATCCTGCTTACCTCAATTCACAAGTTGGGAAAATTGGATTTCATTAACTTCCGTCAATTCACA
AGTTGGGATAGAATGACCGTTTACAAGTTCAATTTTGTATATGAGTATTACAAAACGACGCGTGGAAATGCGGCATCAAGAGAAATG
TACGAAGCACTTTCTCAACAATCCGATGGTCCGTCCTTGCTTGCAATATTGCTCCATAATGGACAGGACGAGCCACTCATCAAGGAC
AGTTGCAAATCTGTCGAACTAGCGCAATTTTCGCAATGGGCTGAAGATGATAGTAAAAAGCTCGAAAACATTTTCAATTTCTTGGCT
AACGATTATAATTTGATTCGGGAAAGAAAACATGAAATGATGATTTTGGCGGAGAGAGTGGATGTAATCAAAAACATCAAGCAGTCT
TATAGCGACTTTCTCA
>PhRXLR34_730 PLHAL7300036

AAGGTCAAACTTACGGCAGACATTGAGAGCCGCTTGCAAAAATTACTTCTTTCTCCTCCTCTCGTGGGTGGGTTGGAGGTTATACTG
TCTGTATATATGTCAGACTGTCACGAAAAAAACAATTGCTTCTCATATCTGAAATATTCGAGCAACACAAAGAACTAAAGAGTAAAT
GAGAGTTCAATACGCAGTACAACTAGCTATTGCCGTCGTTGTAATGTGCGTCGACGCTAAGACCTCAATAACAAGCGCCAAGATAAT
CAGACAGTCGAATTCTTCGTCATCAGCTCTTCCAACTGCTGACGATAACGGCAGCACCTTTGAACTTGTCCAAAGTGACGAAGAGAG
GTTTTGGCTAATTCATTATCCGTCGGATCTACTGATGTCAAGCAATTGGTTCATCAATTCGGTATCTAAGTTTTTCAAGATGATTCA
TTACCCACCGAATAGCCTGTTGAAATCCTGCTTACCTCAATTCACAAGTTGGGAAAATTGGATTTCATTAACTTCCGTCAATTCACA
AGTTGGGATAGAATGACCGTTTACAAGTTCAATTTTGTATATGAGTATTACAAAACGACGCGTGGAAATGCGGCATCAAGAGAAATG
TACGAAGCACTTTCTCAACAATCCGATGGTCCGTCCTTGCTTGCAATATTGCTCCATAATGGACAGGACGAGCCACTCATCAAGGAC
AGTTGCAAATCTGTCGAACTAGCGCAATTTTCGCAATGGGCTGAAGATGATAGTAAAAAGCTCGAAAACATTTTCAATTTCTTGGCT
AACGATTATAATTTGATTCGGGAAAGAAAACATGAAATGATGATTTTGGCGGAGAGAGTGGATGTAATCAAAAACATCAAGCAGTCT
TATAGCGACTTTCTCA

>PhRXLR35_100 PLHAL100A10499
CTTGATAATTTGCTTTGAAGAGCTCAAGTACATCCACAGACGTCATTCCGTTGCAGGTTTACAGACTCAGTAAAATGCGCCTTGCCT
TCTTTTTCATTATGGCTTCAACTACATCTCTTCTCGTGAATAGCGTTGCAATTTCTACTGCCGGTGCAGGTAAAGTCATGACCAAAA
ATAAGGACTCGTCGACCAGAAAAGGTCAAATCGAATATACTGAAAAGCTTCTCCGTGGAGCTGGAAGTCCTCAGCGCGAGGAAGAAC
GTTCTACTAATAATGTCAAATTCCCGTCTTCTCTCACGAATGAAATCGCGAAACTTCAAGTTTCTTCATCAAAAGCAACGAATCCTG
TGGTGCAACCAGGTATAGTGGCACCTTCAAAAGCGGTTCCAAACAAAGCGAATCGTCTCGTATTCAATATACTGTTAGAAGGTATCG
ATTTCAAGCGT
>PhRXLR35_334 PLHAL3340491
CTTGATAATTTGCTTTGAAGAGCTCAAGTACATCCACAGACGTCATTCCGTTGCAGGTTTACAGACTCAGTAAAATGCGCCTTGCCT
TCTTTTTCATTATGGCTTCAACTACATCTCTTCTCGTGAATAGCGTTGCAATTTCTACTGCCGGTGCAGGTAAAGTCATGACCAAAA
ATAAGGACTCGTCGACCAGAAAAGGTCAAATCGAATATACTAAAAAGCTTCTCCGTGGAGCTGGAAGTCCTCAGCGCGAGGAAGAAC
GTTCTACTAATAATGTCAAATTCCCGTCTTCTCTCACGAATGAAATCGCGAAACTTCAAGTTTCTTCATCAAAAGCAACGAATCCTG
TGGTGCAACCAGGTATAGTGGCACCTTCAAAAGCGGTTCCAAACAAAGCGAATCATCTCGTATTCAATATACTGTTAGAAGGTATCG
ATTTCAAGCGT
>PhRXLR35_703 PLHAL7030571
CTTGATAATTTGCTTTGAAGAGCTCAAGTACATCCACAGACGTCATTCCGTTGCAGGTTTACAGACTCAGTAAAATGCGCCTTGCCT
TCTTTTTCATTATGGCTTCAACTACATCTCTTCTCGTGAATAGCGTTGCAATTTCTACTGCCGGTGCAGGTAAAGTCATGACCAAAA
ATAAGGACTCGTCGACCAGAAAAGGTCAAATCGAATATACTGAAAAGCTTCTCCGTGGAGCTGGAAGTCCTCAGCGCGAGGAAGAAC
GTTCTACTAATAATGTCAAATTCCCGTCTTCTCTCACGAATGAAATCGCGAAACTTCAAGTTTCTTCATCAAAAGCAACGAATCCTG
TGGTGCAACCAGGTATAGTGGCACCTTCAAAAGCGGTTCCAAACAAAGCGAATCGTCACGTATTCAATATACTGTTAGAAGGTATCG
ATTTCAAGCGT
>PhRXLR35_710 PLHAL7100194
CTTGATAATTTGCTTTGAAGAGCTCAAGTACATCCACAGACGTCATTCCGTTGCAGGTTTACAGACTCAGTAAAATGCGCCTTGCCT
TCTTTTTCATTATGGCTTCAACTACATCTCTTCTCGTGAATAGCGTTGCAATTTCTACTGCCGGTGCAGGTAAAGTCATGACCAAAA
ATAAGGACTCGTCGACCAGAAAAGGTCAAATCGAATATACTAAAAAGCTTCTCCGTGGAGCTGGAAGTCCTCAGCGCGAGGAAGAAC
GTTCTACTAATAATGTCAAATTCCCGTCTTCTCTCACGAATGAAATCGCGAAACTTCAAGTTTCTTCATCAAAAGCAACGAATCCTG
TGGTGCAACCAGGTATAGTGGCACCTTCAAAAGCGGTTCCAAACAAAGCGAATCATCTCGTATTCAATATACTGTTAGAAGGTATCG
ATTTCAAGCGT
>PhRXLR35_304 PLHAL3040530
CTTGATAATTTGCTTTGAAGAGCTCAAGTACATCCACAGACGTCATTCCGTTGCAGGTTTACAGACTCAGTAAAATGCGCCTTGCCT
TCTTTTTCATTATGGCTTCAACTACATCTCTTCTCGTGAATAGCGTTGCAATTTCTACTGCCGGTGCAGGTAAAGTCATGACCAAAA
ATAAGGACTCGTCGACCAGAAAAGGTCAAATCGAATATACTGAAAAGCTTCTCCGTGGAGCTGGAAGTCCTCAGCGCGAGGAAGAAC
GTTCTACTAATAATGTCAAATTCCCGTCTTCTCTCACGAATGAAATCGCGAAACTTCAAGTTTCTTCATCAAAAGCAACGAATCCTG
TGGTGCAACCAGGTATAGTGGCACCTTCAAAAGCGGTTCCAAACAAAGCGAATCGTCTCGTATTCAATATACTGTTAGAAGGTATCG
ATTTCAAGCGT
>PhRXLR35_700 PLHAL7000693
CTTGATAATTTGCTTTGAAGAGCTCAAGTACATCCACAGACGTCATTCCGTTGCAGGTTTACAGACTCAGTAAAATGCGCCTTGCCT
TCTTTTTCATTATGGCTTCAACTACATCTCTTCTCGTGAATAGCGTTGCAATTTCTACTGCCGGTGCAGGTAAAGTCATGACCAAAA
ATAAGGACTCGTCGACCAGAAAAGGTCAAATCGAATATACTGAAAAGCTTCTCCGTGGAGCTGGAAGTCCTCAGCGCGAGGAAGAAC
GTTCTACTAATAATGTCAAATTCCCGTCTTCTCTCACGAATGAAATCGCGAAACTTCAAGTTTCTTCATCAAAAGCAACGAATCCTG
TGGTGCAACCAGGTATAGTGGCACCTTCAAAAGCGGTTCCAAACAAAGCGAATCGTCACGTATTCAATATACTGTTAGAAGGTATCG
ATTTCAAGCGT
>PhRXLR35_730 PLHAL7300137
CTTGATAATTTGCTTTGAAGAGCTCAAGTACATCCACAGACGTCATTCCGTTGCAGGTTTACAGACTCAGTAAAATGCGCCTTGCCT
TCTTTTTCATTATGGCTTCAACTACATCTCTTCTCGTGAATAGCGTTGCAATTTCTACTGCCGGTGCAGGTAAAGTCATGACCAAAA
ATAAGGACTCGTCGACCAGAAAAGGTCAAATCGAATATACTGAAAAGCTTCTCCGTGGAGCTGGAAGTCCTCAGCGCGAGGAAGAAC
GTTCTACTAATAATGTCAAATTCCCGTCTTCTCTCACGAATGAAATCGCGAAACTTCAAGTTTCTTCATCAAAAGCAACGAATCCTG
TGGTGCAACCAGGTATAGTGGCACCTTCAAAAGCGGTTCCAAACAAAGCGAATCGTCACGTATTCAATATACTGTTAGAAGGTATCG
ATTTCAAGCGT

>PhRXLR36_100 PLHAL100A11143
GTTCTAGTTTTCATCAATGTGCCGTGCATTCGACTTAGCCACTACCCTCTTCATCGTAATCAGTCCCTGTGCAGCTGCTGATTTCGA
GCAGATCAAACCTTTTGATGCGCAAGCCCATGACATCTTACCAAACGGTGTTACTGACACGAAGCCGTCACCAAGAAGGTATCTTAA
ACGGAGCGATGATGACGTGTCTCCCGCAACGGCTGAGGAAGAGCGCGTTGTCATTCATGATCTCCTTGATGAGGCAGGTCAAAAAGC
CCTTTTGCCGTACGAACATTTCAACACAAACAGCATTGATGACTCAACCTAGGTGATCATGAAAAAGGAAGACGTGGCCTCTTTCGC

CGTTTTGAGAGACAAAAAAATTGAACCTCTCCAAAAGAAGTACAACGACGCTATAGATAAATACCAGCAAGCTGTCGGCAGGCTTCA
AATGGCTATAACCGATTCCATAAAAGAACACCAGGCTGCCTATGAAGCTAAGGAAGATTTTCAAGCTGCTTCGGCTTTTATCGTGCC
CACAACGATCGCAACACTTACACAGTCCTCTCCGTTAAAGCCAGAAATGCTGCCCAGGATTACATAAACGCTCTAGTCCTCTACAAT
TTCTACTCCGCTCAAGTCACCAAATTCAACAAAGCCGCCGACACTGTCAAAACCAATGAACAACGCCTATTCCTCAAAGACGCTAAG
GAGAAAGCAGCCGTACGTGCGTCCGTGC
>PhRXLR36_334 PLHAL3341171
GTTCTAGTTTTCATCAATGTGCCGTGCATTCGACTTAGCCACTACCCTCTTCATCGTAATCAGTCCCTGTGCAGCTGCTGATTTCGA
GCAGATCAAACCTTTTGATGCGCAAGCCCATGACATCTTACCAAACGGTGTTACTGACACGAAGCCGTCACCAAGAAGGTATCTTAA
ACGGAGCGATGATGACGTGTCTCCCGCAACGGCTGAGGAAGAGCGCGTTGTCATTCATGATCTCCTTGATGAGGCAGGTCAAAAAGC
CCTTTTGCCGTACGAACATTTCAACACAAACAGCATTGATGACTCAACCTAGGTGATCATGAAAAAGGAAGACGTGGCCTCTTTCGC
CGTTTTGAGAGACAAAAAAATTGAACCTCTCCAAAAGAAGTACAACGACGCTATAGATAAATACCAGCAAGCTGTCGGCAGGCTTCA
AATGGCTATAACCGATTCCATAAAAGAACACCAGGCTGCCTATGAAGCTAAGGAAGATTTTCAAGCTGCTTCGGCTTTTATCGTGCC
CACAACGATCGCAACACTTACACAGTCCTCTCCGTTAAAGCCAGAAATGCTGCCCAGGATTACATAAACGCTCTAGTCCTCTACAAT
TTCTACTCCGCTCAAGTCACCAAATTCAACAAAGCCGCCGACACTGTCAAAACCAATGAACAACGCCTATTCCTCAAAGACGCTAAG
GAGAAAGCAGCCGTACGTGCGTCCGTGC
>PhRXLR36_703 PLHAL7031076
GTTCTAGTTTTCATCAATGTGCCGTGCATTCGACTTAGCCACTACCCTCTTCATCGTAATCAGTCCCTGTGCAGCTGCTGATTTCGA
GCAGATCAAACCTTTTGATGCGCAAGCCCATGACATCTTACCAAACGGTGTTACTGACACGAAGCCGTCACCAAGAAGGTATCTTAA
ACGGAGCGATGATGACGTGTCTCCCGCAACGGCTGAGGAAGAGCGCGTTGTCATTCATGATCTCCTTGATGAGGCAGGTCAAAAAGC
CCTTTTGCCGTACGAACATTTCAACACAAACAGCATTGATGACTCAACCTAGGTGATCATGAAAAAGGAAGACGTGGCCTCTTTCGC
CGTTTTGAGAGACAAAAAAATTGAACCTCTCCAAAAGAAGTACAACGACGCTATAGATAAATACCAGCAAGCTGTCGGCAGGCTTCA
AATGGCTATAACCGATTCCATAAAAGAACACCAGGCTGCCTATGAAGCTAAGGAAGATTTTCAAGCTGCTTCGGCTTTTATCGTGCC
CACAACGATCGCAACACTTACACAGTCCTCTCCGTTAAAGCCAGAAATGCTGCCCAGGATTACATAAACGCTCTAGTCCTCTACAAT
TTCTACTCCGCTCAAGTCACCAAATTCAACAAAGCCGCCGACACTGTCAAAACCAATGAACAACGCCTATTCCTCAAAGACGCTAAG
GAGAAAGCAGCCGTACGTGCGTCCGTGC
>PhRXLR36_710 PLHAL7101030
GTTCTAGTTTTCATCAATGTGCCGTGCATTCGACTTAGCCACTACCCTCTTCATCGTAATCAGTCCCTGTGCAGCTGCTGATTTCGA
GCAGATCAAACCTTTTGATGCGCAAGCCCATGACATCTTACCAAACGGTGTTACTGACACGAAGCCGTCACCAAGAAGGTATCTTAA
ACGGAGCGATGATGACGTGTCTCCCGCAACGGCTGAGGAAGAGCGCGTTGTCATTCATGATCTCCTTGATGAGGCAGGTCAAAAAGC
CCTTTTGCCGTACGAACATTTCAACACAAACAGCATTGATGACTCAACCTAGGTGATCATGAAAAAGGAAGACGTGGCCTCTTTCGC
CGTTTTGAGAGACAAAAAAATTGAACCTCTCCAAAAGAAGTACAACGACGCTATAGATAAATACCAGCAAGCTGTCGGCAGGCTTCA
AATGGCTATAACCGATTCCATAAAAGAACACCAGGCTGCCTATGAAGCTAAGGAAGATTTTCAAGCTGCTTCGGCTTTTATCGTGCC
CACAACGATCGCAACACTTACACAGTCCTCTCCGTTAAAGCCAGAAATGCTGCCCAGGATTACATAAACGCTCTAGTCCTCTACAAT
TTCTACTCCGCTCAAGTCACCAAATTCAACAAAGCCGCCGACACTGTCAAAACCAATGAACAACGCCTATTCCTCAAAGACGCTAAG
GAGAAAGCAGCCGTACGTGCGTCCGTGC
>PhRXLR36_304 PLHAL3041145
GTTCTAGTTTTCATCAATGTGCCGTGCATTCGACTTAGCCACTACCCTCTTCATCGTAATCAGTCCCTGTGCAGCTGCTGATTTCGA
GCAGATCAAACCTTTTGATGCGCAAGCCCATGACATCTTACCAAACGGTGTTACTGACACGAAGCCGTCACCAAGAAGGTATCTTAA
ACGGAGCGATGATGACGTGTCTCCCGCAACGGCTGAGGAAGAGCGCGTTGTCATTCATGATCTCCTTGATGAGGCAGGTCAAAAAGC
CCTTTTGCCGTACGAACATTTCAACACAAACAGCATTGATGACTCAACCTAGGTGATCATGAAAAAGGAAGACGTGGCCTCTTTCGC
CGTTTTGAGAGACAAAAAAATTGAACCTCTCCAAAAGAAGTACAACGACGCTATAGATAAATACCAGCAAGCTGTCGGCAGGCTTCA
AATGGCTATAACCGATTCCATAAAAGAACACCAGGCTGCCTATGAAGCTAAGGAAGATTTTCAAGCTGCTTCGGCTTTTATCGTGCC
CACAACGATCGCAACACTTACACAGTCCTCTCCGTTAAAGCCAGAAATGCTGCCCAGGATTACATAAACGCTCTAGTCCTCTACAAT
TTCTACTCCGCTCAAGTCACCAAATTCAACAAAGCCGCCGACACTGTCAAAACCAATGAACAACGCCTATTCCTCAAAGACGCTAAG
GAGAAAGCAGCCGTACGTGCGTCCGTGC
>PhRXLR36_700 PLHAL7001243
GTTCTAGTTTTCATCAATGTGCCGTGCATTCGACTTAGCCACTACCCTCTTCATCGTAATCAGTCCCTGTGCAGCTGCTGATTTCGA
GCAGATCAAACCTTTTGATGCGCAAGCCCATGACATCTTACCAAACGGTGTTACTGACACGAAGCCGTCACCAAGAAGGTATCTTAA
ACGGAGCGATGATGACGTGTCTCCCGCAACGGCTGAGGAAGAGCGCGTTGTCATTCATGATCTCCTTGATGAGGCAGGTCAAAAAGC
CCTTTTGCCGTACGAACATTTCAACACAAACAGCATTGATGACTCAACCTAGGTGATCATGAAAAAGGAAGACGTGGCCTCTTTCGC
CGTTTTGAGAGACAAAAAAATTGAACCTCTCCAAAAGAAGTACAACGACGCTATAGATAAATACCAGCAAGCTGTCGGCAGGCTTCA
AATGGCTATAACCGATTCCATAAAAGAACACCAGGCTGCCTATGAAGCTAAGGAAGATTTTCAAGCTGCTTCGGCTTTTATCGTGCC
CACAACGATCGCAACACTTACACAGTCCTCTCCGTTAAAGCCAGAAATGCTGCCCAGGATTACATAAACGCTCTAGTCCTCTACAAT
TTCTACTCCGCTCAAGTCACCAAATTCAACAAAGCCGCCGACACTGTCAAAACCAATGAACAACGCCTATTCCTCAAAGACGCTAAG
GAGAAAGCAGCCGTACGTGCGTCCGTGC
>PhRXLR36_730 PLHAL7301182
GTTCTAGTTTTCATCAATGTGCCGTGCATTCGACTTAGCCACTACCCTCTTCATCGTAATCAGTCCCTGTGCAGCTGCTGATTTCGA
GCAGATCAAACCTTTTGATGCGCAAGCCCATGACATCTTACCAAACGGTGTTACTGACACGAAGCCGTCACCAAGAAGGTATCTTAA
ACGGAGCGATGATGACGTGTCTCCCGCAACGGCTGAGGAAGAGCGCGTTGTCATTCATGATCTCCTTGATGAGGCAGGTCAAAAAGC
CCTTTTGCCGTACGAACATTTCAACACAAACAGCATTGATGACTCAACCTAGGTGATCATGAAAAAGGAAGACGTGGCCTCTTTCGC
CGTTTTGAGAGACAAAAAAATTGAACCTCTCCAAAAGAAGTACAACGACGCTATAGATAAATACCAGCAAGCTGTCGGCAGGCTTCA
AATGGCTATAACCGATTCCATAAAAGAACACCAGGCTGCCTATGAAGCTAAGGAAGATTTTCAAGCTGCTTCGGCTTTTATCGTGCC
CACAACGATCGCAACACTTACACAGTCCTCTCCGTTAAAGCCAGAAATGCTGCCCAGGATTACATAAACGCTCTAGTCCTCTACAAT
TTCTACTCCGCTCAAGTCACCAAATTCAACAAAGCCGCCGACACTGTCAAAACCAATGAACAACGCCTATTCCTCAAAGACGCTAAG
GAGAAAGCAGCCGTACGTGCGTCCGTGC

>PhRXLR41_100 PLHAL100A12976
AAAAAGTCAATCAAGTTCGAAATCTTTTCAACTCATGCGAGGTGCATTTTACGTGGCCAATGCTATCCTCATCGCGAGTAGCATTCG
CACAGCTGCTGAATCTGTCCAGATCAAGTCTGATATAACGCAATACCATGACAAGTTGCCAGTCAGTGATTCTGACACAAAGACGTT
GCCCAGAAGATCTCTTAGAGGGAGCGGAGATCAACTGGAATCTCCAGTGGCTGGAGAAGAGCGCGTCACTTCACCTGGAGTGCTCAA
GGGTGCAGGGAACGATGTGTCTGAGGCAATCTTGTGGTTAAATAAATTCAAGATGAAA
>PhRXLR41_334 PLHAL3343099
AAAAAGTCAATCAAGTTCGAAATCTTTTCAACTCATGCGAGGTGCATTTTACGTGGCCAATGCTATCCTCATCGCGAGTAGCATTCG
CACAGCTGCTGAATCTGTCCAGATCAAGTCTGATATAACGCAATACCATGACAAGTTGCCAGTCAGTGATTCTGACACAAAGACGTT
GCCCAGAAGATCTCTTAGAGGGAGCGGAGATCAACTGGAATCTCCAGTGGCTGGAGAAGAGCGCGTCACTTCACCTGGAGTGCTCAA
GGGTGCAGGGAACGATGTGTCTGAGGCAATCTTGTGGTTAAATAAATTCAAGATGAAA
>PhRXLR41_304 PLHAL3042961
AAAAAGTCAATCAAGTTCGAAATCTTTTCAACTCATGCGAGGTGCATTTTACGTGGCCAATGCTATCCTCATCGCGAGTAGCATTCG
CACAGCTGCTGAATCTGTCCAGATCAAGTCTGATATAACGCAATACCATGACAAGTTGCCAGTCAGTGATTCTGACACAAAGACGTT
GCCCAGAAGATCTCTTAGAGGGAGCGGAGATCAACTGGAATCTCCAGTGGCTGGAGAAGAGCGCGTCACTTCACCTGGAGTGCTCAA
GGGTGCAGGGAACGATGTGTCTGAGGCAATCTTGTGGTTAAATAAATTCAAGATGAAA

>PhRXLR42_100 PLHAL100A10212
TCGTCAGGATACATATCTTAAAATTCCAAGTCAGTTCTTTGTCTCACTCTCTTCCTCATGCGCGGTGCAATCTACGTGACTACTTCT
CTCCTCATCGCAACAAGCGTCCACACAGCTGCTGAATCCCTTCAGATCAAGTCACGAAATGTACGAAAGCATGACATCTTGCCAATT
GGTGGTATGGACACCAAGACGTTACCCAAAATGTCGTTGAAAGAAAGCAATGACCGAGTGGTGCACACCACAGCTGATGAAGAGCGC
AACAGTTTAGCTAGATTGATTGCCAAGACACGTGGTGAAGCATTAAAATTAATGTTGCCGGAAACATTGGCAAACGAGAAGGATAAA
GCGATCGTCAAAGTCAGTGCGGATGACTTAAGCAAGATGATTATGAAAAACTTGGGCTCTCCTGCCGCCTTCAAGGAAAAAAAAGAA
ATCCAAGATCTTGAAGAAGAAGATGGCCAACTCATCGAAGATTTGGCCTATCTCGATACCGTGTATACTGCGCGCCGTCAAATAGCC
GATGCAACTCGCCAATTTTCCCTGAACAAAAGTAATGAAGCCAAAAAACTGATCAAG
>PhRXLR42_334 PLHAL3340207
TCGTCAGGATACATATCTTAAAATTCCAAGTCAGTTCTTTGTCTCACTCTCTTCCTCATGCGCGGTGCAATCTACGTGACTACTTCT
CTCCTCATCGCAACAAGCGTCCACACAGCTGCTGAATCCCTTCAGATCAAGTCACGAAATGTACGAAAGCATGACATCTTGCCAATT
GGTGGTATGGACACCAAGACGTTACCCAAAATGTCGTTGAAAGAAAGCAATGACCGAGTGGTGCACACCACAGCTGATGAAGAGCGC
AACAGTTTAGCTAGATTGATTGCCAAGACACGTGGTGAAGCATTAAAATTAATGTTGCCGGAAACATTGGCAAACGAGAAGGATAAA
GCGATCGTCAAAGTCAGTGCGGATGACTTAAGCAAGATGATTATGAAAAACTTGGGCTCTCCTGCCGCCTTCAAGGAAAAAAAAGAA
ATCCAAGATCTTGAAGAAGAAGATGGCCAACTCATCGAAGATTTGGCCTATCTCGATACCGTGTATACTGCGCGCCGTCAAATAGCC
GATGCAACTCGCCAATTTTCCCTGAACAAAAGTAATGAAGCCAAAAAACTGATCAAG
>PhRXLR42_703 PLHAL7030246
TCGTCAGGATACATATCTTAAAATTCCAAGTCAGTTCTTTGTCTCACTCTCTTCCTCATGCGCGGTGCAATCTACGTGACTACTTCT
CTCCTCATCGCAACAAGCGTCCACACAGCTGCTGAATCCCTTCAGATCAAGTCACGAAATGTACGAAAGCATGACATCTTGCCAATT
GGTGGTATGGACACCAAGACGTTACCCAAAATGTCGTTGAAAGAAAGCAATGACCGAGTGGTGCACACCACAGCTGATGAAGAGCGC
AACAGTTTAGCTAGATTGATTGCCAAGACACGTGGTGAAGCATTAAAATTAATGTTGCCGGAAACATTGGCAAACGAGAAGGATAAA
GCGATCGTCAAAGTCAGTGCGGATGACTTAAGCAAGATGATTATGAAAAACTTGGGCTCTCCTGCCGCCTTCAAGGAAAAAAAAGAA
ATCCAAGATCTTGAAGAAGAAGATGGCCAACTCATCGAAGATTTGGCCTATCTCGATACCGTGTATACTGCGCGCCGTCAAATAGCC
GATGCAACTCGCCAATTTTCCCTGAACAAAAGTAATGAAGCCAAAAAACTGATCAAG
>PhRXLR42_710 PLHAL7100279
TCGTCAGGATACATATCTTAAAATTCCAAGTCAGTTCTTTGTCTCACTCTCTTCCTCATGCGCGGTGCAATCTACGTGACTACTTCT
CTCCTCATCGCAACAAGCGTCCACACAGCTGCTGAATCCCTTCAGATCAAGTCACGAAATGTACGAAAGCATGACATCTTGCCAATT
GGTGGTATGGACACCAAGACGTTACCCAAAATGTCGTTGAAAGAAAGCAATGACCGAGTGGTGCACACCACAGCTGATGAAGAGCGC
AACAGTTTAGCTAGATTGATTGCCAAGACACGTGGTGAAGCATTAAAATTAATGTTGCCGGAAACATTGGCAAACGAGAAGGATAAA
GCGATCGTCAAAGTCAGTGCGGATGACTTAAGCAAGATGATTATGAAAAACTTGGGCTCTCCTGCCGCCTTCAAGGAAAAAAAAGAA
ATCCAAGATCTTGAAGAAGAAGATGGCCAACTCATCGAAGATTTGGCCTATCTCGATACCGTGTATACTGCGCGCCGTCAAATAGCC
GATGCAACTCGCCAATTTTCCCTGAACAAAAGTAATGAAGCCAAAAAACTGATCAAG
>PhRXLR42_304 PLHAL3040563
TCGTCAGGATACATATCTTAAAATTCCAAGTCAGTTCTTTGTCTCACTCTCTTCCTCATGCGCGGTGCAATCTACGTGACTACTTCT
CTCCTCATCGCAACAAGCGTCCACACAGCTGCTGAATCCCTTCAGATCAAGTCACGAAATGTACGAAAGCATGACATCTTGCCAATT
GGTGGTATGGACACCAAGACGTTACCCAAAATGTCGTTGAAAGAAAGCAATGACCGAGTGGTGCACACCACAGCTGATGAAGAGCGC
AACAGTTTAGCTAGATTGATTGCCAAGACACGTGGTGAAGCATTAAAATTAATGTTGCCGGAAACATTGGCAAACGAGAAGGATAAA
GCGATCGTCAAAGTCAGTGCGGATGACTTAAGCAAGATGATTATGAAAAACTTGGGCTCTCCTGCCGCCTTCAAGGAAAAAAAAGAA
ATCCAAGATCTTGAAGAAGAAGATGGCCAACTCATCGAAGATTTGGCCTATCTCGATACCGTGTATACTGCGCGCCGTCAAATAGCC
GATGCAACTCGCCAATTTTCCCTGAACAAAAGTAATGAAGCCAAAAAACTGATCAAG
>PhRXLR42_700 PLHAL7000188
TCGTCAGGATACATATCTTAAAATTCCAAGTCAGTTCTTTGTCTCACTCTCTTCCTCATGCGCGGTGCAATCTACGTGACTACTTCT
CTCCTCATCGCAACAAGCGTCCACACAGCTGCTGAATCCCTTCAGATCAAGTCACGAAATGTACGAAAGCATGACATCTTGCCAATT
GGTGGTATGGACACCAAGACGTTACCCAAAATGTCGTTGAAAGAAAGCAATGACCGAGTGGTGCACACCACAGCTGATGAAGAGCGC
AACAGTTTAGCTAGATTGATTGCCAAGACACGTGGTGAAGCATTAAAATTAATGTTGCCGGAAACATTGGCAAACGAGAAGGATAAA
GCGATCGTCAAAGTCAGTGCGGATGACTTAAGCAAGATGATTATGAAAAACTTGGGCTCTCCTGCCGCCTTCAAGGAAAAAAAAGAA
ATCCAAGATCTTGAAGAAGAAGATGGCCAACTCATCGAAGATTTGGCCTATCTCGATACCGTGTATACTGCGCGCCGTCAAATAGCC
GATGCAACTCGCCAATTTTCCCTGAACAAAAGTAATGAAGCCAAAAAACTGATCAAG
>PhRXLR42_730 PLHAL7300227
TCGTCAGGATACATATCTTAAAATTCCAAGTCAGTTCTTTGTCTCACTCTCTTCCTCATGCGCGGTGCAATCTACGTGACTACTTCT
CTCCTCATCGCAACAAGCGTCCACACAGCTGCTGAATCCCTTCAGATCAAGTCACGAAATGTACGAAAGCATGACATCTTGCCAATT

GGTGGTATGGACACCAAGACGTTACCCAAAATGTCGTTGAAAGAAAGCAATGACCGAGTGGTGCACACCACAGCTGATGAAGAGCGC
AACAGTTTAGCTAGATTGATTGCCAAGACACGTGGTGAAGCATTAAAATTAATGTTGCCGGAAACATTGGCAAACGAGAAGGATAAA
GCGATCGTCAAAGTCAGTGCGGATGACTTAAGCAAGATGATTATGAAAAACTTGGGCTCTCCTGCCGCCTTCAAGGAAAAAAAAGAA
ATCCAAGATCTTGAAGAAGAAGATGGCCAACTCATCGAAGATTTGGCCTATCTCGATACCGTGTATACTGCGCGCCGTCAAATAGCC
GATGCAACTCGCCAATTTTCCCTGAACAAAAGTAATGAAGCCAAAAAACTGATCAAG

>PhRXLR43_100 PLHAL100A11758
GGGATGTACGACACTAGCTCTACGCTGCATTATAATACATGTAAACTATGATTATGCACACAAGGGAACGATAGAAACAGCAACGTG
AAGGTAAAGTATCTCAGTAATTAATCAGTTCATTTCTCATTTTCCTCGTAACTACACTAGCGCCTCTTCAAGACCCCCGATGCTGCT
CCGCTCATTTCTACTCATTTGTGCGGCTACGCTGTTTGTTGGTCTCAGCGAAGTTTCAAGTTCAAGTCATACCAATTCATCGGATTC
AAAGGGTACTGCTTCATTGAACAGCCAATCTACTCCAACTGACAACATCATCTACTCTGATGAGGAAGAGAGGCATGTAGTCGTACC
AACAGCTGTCGCCTCGAAACTGAGAGACGATTTAAATAAGGCTCTAAACAAACTTCCCAGCATCTTGAAGCGCGATGCCACCACCCT
ACCCGAGAATGTTGGGCCAAAGACAAATAATCCCCTTACAAATGCGTTTACTCCACAAAAATCGACACCAAATCACCAAGTGGATCC
AAGTGTGGTACCAACGATAAGTTTAGCTCAGAAGATGAGGATCAA
>PhRXLR43_334 PLHAL3341852
GGGATGTACGACACTAGCTCTACGCTGCATTATAATACATGTAAACTATGATTATGCACACAAGGGAACGATAGAAACAGCAACGTG
AAGGTAAAGTATCTCAGTAATTAATCAGTTCATTTCTCATTTTCCTCGTAACTACACTAGCGCCTCTTCAAGACCCCCGATGCTGCT
CCGCTCATTTCTACTCATTTGTGCGGCTACGCTGTTTGTTGGTCTCAGCGAAGTTTCAAGTTCAAGTCATACCAATTCATCGGATTC
AAAGGGTACTGCTTCATTGAACAGCCAATCTACTCCAACTGACAACATCATCTACTCTGATGAGGAAGAGAGGCATGTAGTCGTACC
AACAGCTGTCGCCTCGAAACTGAGAGACGATTTAAATAAGGCTCTAAACAAACTTCCCAGCATCTTGAAGCGCGATGCCACCACCCT
ACCCGAGAATGTTGGGCCAAAGACAAATAATCCCCTTACAAATGCGTTTACTCCACAAAAATCGACACCAAATCACCAAGTGGATCC
AAGTGTGGTACCAACGATAAGTTTAGCTCAGAAGATGAGGATCAA
>PhRXLR43_703 PLHAL7031622
GGGATGTACGACACTAGCTCTACGCTGCATTATAATACATGTAAACTATGATTATGCACACAAGGGAACGATAGAAACAGCAACGTG
AAGGTAAAGTATCTCAGTAATTAATCAGTTCATTTCTCATTTTCCTCGTAACTACACTAGCGCCTCTTCAAGACCCCCGATGCTGCT
CCGCTCATTTCTACTCATTTGTGCGGCTACGCTGTTTGTTGGTCTCAGCGAAGTTTCAAGTTCAAGTCATACCAATTCATCGGATTC
AAAGGGTACTGCTTCATTGAACAGCCAATCTACTCCAACTGACAACATCATCTACTCTGATGAGGAAGAGAGGCATGTAGTCGTACC
AACAGCTGTCGCCTCGAAACTGAGAGACGATTTAAATAAGGCTCTAAACAAACTTCCCAGCATCTTGAAGCGCGATGCCACCACCCT
ACCCGAGAATGTTGGGCCAAAGACAAATAATCCCCTTACAAATGCGTTTACTCCACAAAAATCGACACCAAATCACCAAGTGGATCC
AAGTGTGGTACCAACGATAAGTTTAGCTCAGAAGATGAGGATCAA
>PhRXLR43_710 PLHAL7101446
GGGATGTACGACACTAGCTCTACGCTGCATTATAATACATGTAAACTATGATTATGCACACAAGGGAACGATAGAAACAGCAACGTG
AAGGTAAAGTATCTCAGTAATTAATCAGTTCATTTCTCATTTTCCTCGTAACTACACTAGCGCCTCTTCAAGACCCCCGATGCTGCT
CCGCTCATTTCTACTCATTTGTGCGGCTACGCTGTTTGTTGGTCTCAGCGAAGTTTCAAGTTCAAGTCATACCAATTCATCGGATTC
AAAGGGTACTGCTTCATTGAACAGCCAATCTACTCCAACTGACAACATCATCTACTCTGATGAGGAAGAGAGGCATGTAGTCGTACC
AACAGCTGTCGCCTCGAAACTGAGAGACGATTTAAATAAGGCTCTAAACAAACTTCCCAGCATCTTGAAGCGCGATGCCACCACCCT
ACCCGAGAATGTTGGGCCAAAGACAAATAATCCCCTTACAAATGCGTTTACTCCACAAAAATCGACACCAAATCACCAAGTGGATCC
AAGTGTGGTACCAACGATAAGTTTAGCTCAGAAGATGAGGATCAA
>PhRXLR43_304 PLHAL3041750
GGGATGTACGACACTAGCTCTACGCTGCATTATAATACATGTAAACTATGATTATGCACACAAGGGAACGATAGAAACAGCAACGTG
AAGGTAAAGTATCTCAGTAATTAATCAGTTCATTTCTCATTTTCCTCGTAACTACACTAGCGCCTCTTCAAGACCCCCGATGCTGCT
CCGCTCATTTCTACTCATTTGTGCGGCTACGCTGTTTGTTGGTCTCAGCGAAGTTTCAAGTTCAAGTCATACCAATTCATCGGATTC
AAAGGGTACTGCTTCATTGAACAGCCAATCTACTCCAACTGACAACATCATCTACTCTGATGAGGAAGAGAGGCATGTAGTCGTACC
AACAGCTGTCGCCTCGAAACTGAGAGACGATTTAAATAAGGCTCTAAACAAACTTCCCAGCATCTTGAAGCGCGATGCCACCACCCT
ACCCGAGAATGTTGGGCCAAAGACAAATAATCCCCTTACAAATGCGTTTACTCCACAAAAATCGACACCAAATCACCAAGTGGATCC
AAGTGTGGTACCAACGATAAGTTTAGCTCAGAAGATGAGGATCAA
>PhRXLR43_700 PLHAL7002047
GGGATGTACGACACTAGCTCTACGCTGCATTATAATACATGTAAACTATGATTATGCACACAAGGGAACGATAGAAACAGCAACGTG
AAGGTAAAGTATCTCAGTAATTAATCAGTTCATTTCTCATTTTCCTCGTAACTACACTAGCGCCTCTTCAAGACCCCCGATGCTGCT
CCGCTCATTTCTACTCATTTGTGCGGCTACGCTGTTTGTTGGTCTCAGCGAAGTTTCAAGTTCAAGTCATACCAATTCATCGGATTC
AAAGGGTACTGCTTCATTGAACAGCCAATCTACTCCAACTGACAACATCATCTACTCTGATGAGGAAGAGAGGCATGTAGTCGTACC
AACAGCTGTCGCCTCGAAACTGAGAGACGATTTAAATAAGGCTCTAAACAAACTTCCCAGCATCTTGAAGCGCGATGCCACCACCCT
ACCCGAGAATGTTGGGCCAAAGACAAATAATCCCCTTACAAATGCGTTTACTCCACAAAAATCGACACCAAATCACCAAGTGGATCC
AAGTGTGGTACCAACGATAAGTTTAGCTCAGAAGATGAGGATCAA
>PhRXLR43_730 PLHAL7301706
GGGATGTACGACACTAGCTCTACGCTGCATTATAATACATGTAAACTATGATTATGCACACAAGGGAACGATAGAAACAGCAACGTG
AAGGTAAAGTATCTCAGTAATTAATCAGTTCATTTCTCATTTTCCTCGTAACTACACTAGCGCCTCTTCAAGACCCCCGATGCTGCT
CCGCTCATTTCTACTCATTTGTGCGGCTACGCTGTTTGTTGGTCTCAGCGAAGTTTCAAGTTCAAGTCATACCAATTCATCGGATTC
AAAGGGTACTGCTTCATTGAACAGCCAATCTACTCCAACTGACAACATCATCTACTCTGATGAGGAAGAGAGGCATGTAGTCGTACC
AACAGCTGTCGCCTCGAAACTGAGAGACGATTTAAATAAGGCTCTAAACAAACTTCCCAGCATCTTGAAGCGCGATGCCACCACCCT
ACCCGAGAATGTTGGGCCAAAGACAAATAATCCCCTTACAAATGCGTTTACTCCACAAAAATCGACACCAAATCACCAAGTGGATCC
AAGTGTGGTACCAACGATAAGTTTAGCTCAGAAGATGAGGATCAA

>PhRXLR44_100 PLHAL100A10434
ACAATCGAACATTCGTCAAACTTAGCAAACACCAATTGAGACTTTTGACAGAATCAATCAATTCGTCATGCGACACCAACCGTTTCT
GGCCACGCTGCTTGTTACTATCACTCTACACGCAAGCGGAATTCCATCCCTTGCTGCTAATGTGGCGTCTTCGGGCGTTCCATTGGA
GCGTCCTCTGTCTAAACTGGTTGGTACAGTTGATAAGAGGCATTTAAAAGCTGGGGACGCTGCTGAATTAATGGAGACTGAAGAAAG

AACGAGGACTTTTGGAACATTCTTTGCTAATGCAAAACAGGCCAAAAATGTCAAAATTACAGCTGGCGGTGCGACATCAACAGGCGT
GAATATCGAAAAAGTGCAAGCAGCCGTGAAAAACTTCGAAGCTCCAAAAG
>PhRXLR44_334 PLHAL3341719
ACAATCGAACATTCGTCAAACTTAGCAAACACCAATTGAGACTTTTGACAGAATCAATCAATTCGTCATGCGACACCAACCGTTTCT
GGCCACGCTGCTTGTTACTATCACTCTACACGCAAGCGGAATTCCATCCCTTGCTGCTAATGTGGCGTCTTCGGGCGTTCCATTGGA
GCGTCCTCTGTCTAAACTGGTTGGTACAGTTGATAAGAGGCATTTAAAAGCTGGGGACGCTGCTGAATTAATGGAGACTGAAGAAAG
AACGAGGACTTTTGGAACATTCTTTGCTAATGCAAAACAGGCCAAAAATGTCAAAATTACAGCTGGCGGTGCGACATCAACAGGCGT
GAATATCGAAAAAGTGCAAGCAGCCGTGAAAAACTTCGAAGCTCCAAAAG
>PhRXLR44_703 PLHAL7031329
ACAATCGAACATTCGTCAAACTTAGCAAACACCAATTGAGACTTTTGACAGAATCAATCAATTCGTCATGCGACACCAACCGTTTCT
GGCCACGCTGCTTGTTACTATCACTCTACACGCAAGCGGAATTCCATCCCTTGCTGCTAATGTGGCGTCTTCGGGCGTTCCATTGGA
GCGTCCTCTGTCTAAACTGGTTGGTACAGTTGATAAGAGGCATTTAAAAGCTGGGGACGCTGCTGAATTAATGGAGACTGAAGAAAG
AACGAGGACTTTTGGAACATTCTTTGCTAATGCAAAACAGGCCAAAAATGTCAAAATTACAGCTGGCGGTGCGACATCAACAGGCGT
GAATATCGAAAAAGTGCAAGCAGCCGTGAAAAACTTCGAAGCTCCAAAAG
>PhRXLR44_710 PLHAL7100495
ACAATCGAACATTCGTCAAACTTAGCAAACACCAATTGAGACTTTTGACAGAATCAATCAATTCGTCATGCGACACCAACCGTTTCT
GGCCACGCTGCTTGTTACTATCACTCTACACGCAAGCGGAATTCCATCCCTTGCTGCTAATGTGGCGTCTTCGGGCGTTCCATTGGA
GCGTCCTCTGTCTAAACTGGTTGGTACAGTTGATAAGAGGCATTTAAAAGCTGGGGACGCTGCTGAATTAATGGAGACTGAAGAAAG
AACGAGGACTTTTGGAACATTCTTTGCTAATGCAAAACAGGCCAAAAATGTCAAAATTACAGCTGGCGGTGCGACATCAACAGGCGT
GAATATCGAAAAAGTGCAAGCAGCCGTGAAAAACTTCGAAGCTCCAAAAG
>PhRXLR44_304 PLHAL3040445
ACAATCGAACATTCGTCAAACTTAGCAAACACCAATTGAGACTTTTGACAGAATCAATCAATTCGTCATGCGACACCAACCGTTTCT
GGCCACGCTGCTTGTTACTATCACTCTACACGCAAGCGGAATTCCATCCCTTGCTGCTAATGTGGCGTCTTCGGGCGTTCCATTGGA
GCGTCCTCTGTCTAAACTGGTTGGTACAGTTGATAAGAGGCATTTAAAAGCTGGGGACGCTGCTGAATTAATGGAGACTGAAGAAAG
AACGAGGACTTTTGGAACATTCTTTGCTAATGCAAAACAGGCCAAAAATGTCAAAATTACAGCTGGCGGTGCGACATCAACAGGCGT
GAATATCGAAAAAGTGCAAGCAGCCGTGAAAAACTTCGAAGCTCCAAAAG
>PhRXLR44_700 PLHAL7001666
ACAATCGAACATTCGTCAAACTTAGCAAACACCAATTGAGACTTTTGACAGAATCAATCAATTCGTCATGCGACACCAACCGTTTCT
GGCCACGCTGCTTGTTACTATCACTCTACACGCAAGCGGAATTCCATCCCTTGCTGCTAATGTGGCGTCTTCGGGCGTTCCATTGGA
GCGTCCTCTGTCTAAACTGGTTGGTACAGTTGATAAGAGGCATTTAAAAGCTGGGGACGCTGCTGAATTAATGGAGACTGAAGAAAG
AACGAGGACTTTTGGAACATTCTTTGCTAATGCAAAACAGGCCAAAAATGTCAAAATTACAGCTGGCGGTGCGACATCAACAGGCGT
GAATATCGAAAAAGTGCAAGCAGCCGTGAAAAACTTCGAAGCTCCAAAAG
>PhRXLR44_730 PLHAL7300700
ACAATCGAACATTCGTCAAACTTAGCAAACACCAATTGAGACTTTTGACAGAATCAATCAATTCGTCATGCGACACCAACCGTTTCT
GGCCACGCTGCTTGTTACTATCACTCTACACGCAAGCGGAATTCCATCCCTTGCTGCTAATGTGGCGTCTTCGGGCGTTCCATTGGA
GCGTCCTCTGTCTAAACTGGTTGGTACAGTTGATAAGAGGCATTTAAAAGCTGGGGACGCTGCTGAATTAATGGAGACTGAAGAAAG
AACGAGGACTTTTGGAACATTCTTTGCTAATGCAAAACAGGCCAAAAATGTCAAAATTACAGCTGGCGGTGCGACATCAACAGGCGT
GAATATCGAAAAAGTGCAAGCAGCCGTGAAAAACTTCGAAGCTCCAAAAG

>PhRXLR48_100 PLHAL100A10739
CACTCTCACGAATGTATAGACAAACCCTCAAATCATTCACTCTTCAAATTACCAAGCGGCTGTCCATTTCTTGAGCAGAAAACAAAA
TAGGAGTGATCATGCTCAGACGTAGCGCTCTCATATTGGCAGTGGCGACTATGGCAACTATAGGCAGCGGTTCAGCTCGAGTCTCTG
ATACCACATCGACATCTTCGGCCTATTTGATGACGACAAATCCTACAGCCACTGAAGGCAATCGACTACTTCGTGGTGCAAGTGCGA
ATGACGCCAGTAAAGTTCCAGTCTATCATTATCCACCTGAACAAGATGAAAGGAGCAAGAAAGCACCTTTTATCGAGGATCGACTGA
ATGAAGAGTTGACGAACCCTGAGTACGTTAATCGGCTTTACGAACGATGGTATTCGGAGGGCTTATCGTCAAATGAAGTCGCTTTAG
GTCTGGATCAGATCGATAACCAAGAGGTCCAAGAGACTTATCAAAACCTCGGATTGGGCTATAGAGAGTATATTGATTGGAGGAACA
GTATGTAGTTAGGT
>PhRXLR48_334 PLHAL3340729
CACTCTCACGAATGTATAGACAAACCCTCAAATCATTCACTCTTCAAATTACCAAGCGGCTGTCCATTTCTTGAGCAGAAAACAAAA
TAGGAGTGATCATGCTCAGACGTAGCGCTCTCATATTGGCAGTGGCGACTATGGCAACTATAGGCAGCGGTTCAGCTCGAGTCTCTG
ATACCACATCGACATCTTCGGCCTATTTGATGACGACAAATCCTACAGCCACTGAAGGCAATCGACTACTTCGTGGTGCAAGTGCGA
ATGACGCCAGTAAAGTTCCAGTCTATCATTATCCACCTGAACAAGATGAAAGGAGCAAGAAAGCACCTTTTATCGAGGATCGACTGA
ATGAAGAGTTGACGAACCCTGAGTACGTTAATCGGCTTTACGAACGATGGTATTCGGAGGGCTTATCGTCAAATGAAGTCGCTTTAG
GTCTGGATCAGATCGATAACCAAGAGGTCCAAGAGACTTATCAAAACCTCGGATTGGGCTATAGAGAGTATATTGATTGGAGGAACA
GTATGTAGTTAGGT
>PhRXLR48_703 PLHAL7030741 20409
CACTCTCACGAATGTATAGACAAACCCTCAAATCATTCACTCTTCAAATTACCAAGCGGCTGTCCATTTCTTGAGCAGAAAACAAAA
TAGGAGTGATCATGCTCAGACGTAGCGCTCTCATATTGGCAGTGGCGACTATGGCAACTATAGGCAGCGGTTCAGCTCGAGTCTCTG
ATACCACATCGACATCTTCGGCCTATTTGATGACGACAAATCCTACAGCCACTGAAGGCAATCGACTACTTCGTGGTGCAAGTGCGA
ATGACGCCAGTAAAGTTCCAGTCTATCATTATCCACCTGAACAAGATGAAAGGAGCAAGAAAGCACCTTTTATCGAGGATCGACTGA
ATGAAGAGTTGACGAACCCTGAGTACGTTAATCGGCTTTACGAACGATGGTATTCGGAGGGCTTATCGTCAAATGAAGTCGCTTTAG
GTCTGGATCAGATCGATAACCAAGAGGTCCAAGAGACTTATCAAAACCTCGGATTGGGCTATAGAGAGTATATTGATTGGAGGAACA
GTATGTAGTTAGGT
>PhRXLR48_710 PLHAL7100049
CACTCTCACGAATGTATAGACAAACCCTCAAATCATTCACTCTTCAAATTACCAAGCGGCTGTCCATTTCTTGAGCAGAAAACAAAA
TAGGAGTGATCATGCTCAGACGTAGCGCTCTCATATTGGCAGTGGCGACTATGGCAACTATAGGCAGCGGTTCAGCTCGAGTCTCTG
ATACCACATCGACATCTTCGGCCTATTTGATGACGACAAATCCTACAGCCACTGAAGGCAATCGACTACTTCGTGGTGCAAGTGCGA

ATGACGCCAGTAAAGTTCCAGTCTATCATTATCCACCTGAACAAGATGAAAGGAGCAAGAAAGCACCTTTTATCGAGGATCGACTGA
ATGAAGAGTTGACGAACCCTGAGTACGTTAATCGGCTTTACGAACGATGGTATTCGGAGGGCTTATCGTCAAATGAAGTCGCTTTAG
GTCTGGATCAGATCGATAACCAAGAGGTCCAAGAGACTTATCAAAACCTCGGATTGGGCTATAGAGAGTATATTGATTGGAGGAACA
GTATGTAGTTAGGT
>PhRXLR48_304 PLHAL3040734
CACTCTCACGAATGTATAGACAAACCCTCAAATCATTCACTCTTCAAATTACCAAGCGGCTGTCCATTTCTTGAGCAGAAAACAAAA
TAGGAGTGATCATGCTCAGACGTAGCGCTCTCATATTGGCAGTGGCGACTATGGCAACTATAGGCAGCGGTTCAGCTCGAGTCTCTG
ATACCACATCGACATCTTCGGCCTATTTGATGACGACAAATCCTACAGCCACTGAAGGCAATCGACTACTTCGTGGTGCAAGTGCGA
ATGACGCCAGTAAAGTTCCAGTCTATCATTATCCACCTGAACAAGATGAAAGGAGCAAGAAAGCACCTTTTATCGAGGATCGACTGA
ATGAAGAGTTGACGAACCCTGAGTACGTTAATCGGCTTTACGAACGATGGTATTCGGAGGGCTTATCGTCAAATGAAGTCGCTTTAG
GTCTGGATCAGATCGATAACCAAGAGGTCCAAGAGACTTATCAAAACCTCGGATTGGGCTATAGAGAGTATATTGATTGGAGGAACA
GTATGTAGTTAGGT
>PhRXLR48_700 PLHAL7001611
CACTCTCACGAATGTATAGACAAACCCTCAAATCATTCACTCTTCAAATTACCAAGCGGCTGTCCATTTCTTGAGCAGAAAACAAAA
TAGGAGTGATCATGCTCAGACGTAGCGCTCTCATATTGGCAGTGGCGACTATGGCAACTATAGGCAGCGGTTCAGCTCGAGTCTCTG
ATACCACATCGACATCTTCGGCCTATTTGATGACGACAAATCCTACAGCCACTGAAGGCAATCGACTACTTCGTGGTGCAAGTGCGA
ATGACGCCAGTAAAGTTCCAGTCTATCATTATCCACCTGAACAAGATGAAAGGAGCAAGAAAGCACCTTTTATCGAGGATCGACTGA
ATGAAGAGTTGACGAACCCTGAGTACGTTAATCGGCTTTACGAACGATGGTATTCGGAGGGCTTATCGTCAAATGAAGTCGCTTTAG
GTCTGGATCAGATCGATAACCAAGAGGTCCAAGAGACTTATCAAAACCTCGGATTGGGCTATAGAGAGTATATTGATTGGAGGAACA
GTATGTAGTTAGGT
>PhRXLR48_730 PLHAL7300750
CACTCTCACGAATGTATAGACAAACCCTCAAATCATTCACTCTTCAAATTACCAAGCGGCTGTCCATTTCTTGAGCAGAAAACAAAA
TAGGAGTGATCATGCTCAGACGTAGCGCTCTCATATTGGCAGTGGCGACTATGGCAACTATAGGCAGCGGTTCAGCTCGAGTCTCTG
ATACCACATCGACATCTTCGGCCTATTTGATGACGACAAATCCTACAGCCACTGAAGGCAATCGACTACTTCGTGGTGCAAGTGCGA
ATGACGCCAGTAAAGTTCCAGTCTATCATTATCCACCTGAACAAGATGAAAGGAGCAAGAAAGCACCTTTTATCGAGGATCGACTGA
ATGAAGAGTTGACGAACCCTGAGTACGTTAATCGGCTTTACGAACGATGGTATTCGGAGGGCTTATCGTCAAATGAAGTCGCTTTAG
GTCTGGATCAGATCGATAACCAAGAGGTCCAAGAGACTTATCAAAACCTCGGATTGGGCTATAGAGAGTATATTGATTGGAGGAACA
GTATGTAGTTAGGT

>PhRXLR49_100 PLHAL100A10475
GAAAAGGATAGGCTGCAGGATTGCAGAAAACAATGATTTGCGAATATTTAACTATTGTAAAGCCGGCTCCAGAATTTAAGCAAGGCC
CTAGAGGTCTCACTTATCAACTTTCTCTTCTTATCTCACTACCATCTCCTCCGCCCTACCTTCTGCAACGATGCGCCTTCTCGTACT
ACTACCGGCTGTGATGGCATCAACACTTCTGGCGACTAGTAGTGCCGTTGTCAATCCTGACCCCATTCATCATGCCAACTCTTTACA
AGTAGCTTCGAAAGTTAATATTGCTGCTTTAAAGAATGGTCCTCGTGCTCTGAGAACCGCTGGAACAGTCCCAAAGGAGAGGCGCTT
AGGAGATACATACTATCCCAGCGCTTACTCCTATACTGGCGGGAATAATTATGGATTCAACAACTATTCTCCCAACAGCAACGGCTA
TCAGAACACTTACCCTACAAGATACGGCAACCAGAAGCGAGATGACAGTGATGATTCCAGCAGCGAAGATAGCAAAGACGATTCTAG
CCACGATTAGCCGCATACCTCACCCGCAGTCGATCCTGATATTAATGACTGTATGAAGATGAAGGCTAGAATGACATTCGTGAGCTC
ATCTAAGTTATCGAAGTTCCTCTTGACCGAGGTACGTTTTTATAGTGTTGGGATACAGACCATGTTTGAAAATATACTTAATTT
>PhRXLR49_334 PLHAL3340459
GAAAAGGATAGGCTGCAGGATTGCAGAAAACAATGATTTGCGAATATTTAACTATTGTAAAGCCGGCTCCAGAATTTAAGCAAGGCC
CTAGAGGTCTCACTTATCAACTTTCTCTTCTTATCTCACTACCATCTCCTCCGCCCTACCTTCTGCAACGATGCGCCTTCTCGTACT
ACTACCGGCTGTGATGGCATCAACACTTCTGGCGACTAGTAGTGCCGTTGTCAATCCTGACCCCATTCATCATGCCAACTCTTTACA
AGTAGCTTCGAAAGTTAATATTGCTGCTTTAAAGAATGGTCCTCGTGCTCTGAGAACCGCTGGAACAGTCCCAAAGGAGAGGCGCTT
AGGAGATACATACTATCCCAGCGCTTACTCCTATACTGGCGGGAATAATTATGGATTCAACAACTATTCTCCCAACAGCAACGGCTA
TCAGAACACTTACCCTACAAGATACGGCAACCAGAAGCGAGATGACAGTGATGATTCCAGCAGCGAAGATAGCAAAGACGATTCTAG
CCACGATTAGCCGCATACCTCACCCGCAGTCGATCCTGATATTAATGACTGTATGAAGATGAAGGCTAGAATGACATTCGTGAGCTC
ATCTAAGTTATCGAAGTTCCTCTTGACCGAGGTACGTTTTTATAGTGTTGGGATACAGACCATGTTTGAAAATATACTTAATTT
>PhRXLR49_703 PLHAL7030171
GAAAAGGATAGGCTGCAGGATTGCAGAAAACAATGATTTGCGAATATTTAACTATTGTAAAGCCGGCTCCAGAATTTAAGCAAGGCC
CTAGAGGTCTCACTTATCAACTTTCTCTTCTTATCTCACTACCATCTCCTCCGCCCTACCTTCTGCAACGATGCGCCTTCTCGTACT
ACTACCGGCTGTGATGGCATCAACACTTCTGGCGACTAGTAGTGCCGTTGTCAATCCTGACCCCATTCATCATGCCAACTCTTTACA
AGTAGCTTCGAAAGTTAATATTGCTGCTTTAAAGAATGGTCCTCGTGCTCTGAGAACCGCTGGAACAGTCCCAAAGGAGAGGCGCTT
AGGAGATACATACTATCCCAGCGCTTACTCCTATACTGGCGGGAATAATTATGGATTCAACAACTATTCTCCCAACAGCAACGGCTA
TCAGAACACTTACCCTACAAGATACGGCAACCAGAAGCGAGATGACAGTGATGATTCCAGCAGCGAAGATAGCAAAGACGATTCTAG
CCACGATTAGCCGCATACCTCACCCGCAGTCGATCCTGATATTAATGACTGTATGAAGATGAAGGCTAGAATGACATTCGTGAGCTC
ATCTAAGTTATCGAAGTTCCTCTTGACCGAGGTACGTTTTTATAGTGTTGGGATACAGACCATGTTTGAAAATATACTTAATTT
>PhRXLR49_710 PLHAL7100526
GAAAAGGATAGGCTGCAGGATTGCAGAAAACAATGATTTGCGAATATTTAACTATTGTAAAGCCGGCTCCAGAATTTAAGCAAGGCC
CTAGAGGTCTCACTTATCAACTTTCTCTTCTTATCTCACTACCATCTCCTCCGCCCTACCTTCTGCAACGATGCGCCTTCTCGTACT
ACTACCGGCTGTGATGGCATCAACACTTCTGGCGACTAGTAGTGCCGTTGTCAATCCTGACCCCATTCATCATGCCAACTCTTTACA
AGTAGCTTCGAAAGTTAATATTGCTGCTTTAAAGAATGGTCCTCGTGCTCTGAGAACCGCTGGAACAGTCCCAAAGGAGAGGCGCTT
AGGAGATACATACTATCCCAGCGCTTACTCCTATACTGGCGGGAATAATTATGGATTCAACAACTATTCTCCCAACAGCAACGGCTA
TCAGAACACTTACCCTACAAGATACGGCAACCAGAAGCGAGATGACAGTGATGATTCCAGCAGCGAAGATAGCAAAGACGATTCTAG
CCACGATTAGCCGCATACCTCACCCGCAGTCGATCCTGATATTAATGACTGTATGAAGATGAAGGCTAGAATGACATTCGTGAGCTC
ATCTAAGTTATCGAAGTTCCTCTTGACCGAGGTACGTTTTTATAGTGTTGGGATACAGACCATGTTTGAAAATATACTTAATTT
>PhRXLR49_304 PLHAL3040494

GAAAAGGATAGGCTGCAGGATTGCAGAAAACAATGATTTGCGAATATTTAACTATTGTAAAGCCGGCTCCAGAATTTAAGCAAGGCC
CTAGAGGTCTCACTTATCAACTTTCTCTTCTTATCTCACTACCATCTCCTCCGCCCTACCTTCTGCAACGATGCGCCTTCTCGTACT
ACTACCGGCTGTGATGGCATCAACACTTCTGGCGACTAGTAGTGCCGTTGTCAATCCTGACCCCATTCATCATGCCAACTCTTTACA
AGTAGCTTCGAAAGTTAATATTGCTGCTTTAAAGAATGGTCCTCGTGCTCTGAGAACCGCTGGAACAGTCCCAAAGGAGAGGCGCTT
AGGAGATACATACTATCCCAGCGCTTACTCCTATACTGGCGGGAATAATTATGGATTCAACAACTATTCTCCCAACAGCAACGGCTA
TCAGAACACTTACCCTACAAGATACGGCAACCAGAAGCGAGATGACAGTGATGATTCCAGCAGCGAAGATAGCAAAGACGATTCTAG
CCACGATTAGCCGCATACCTCACCCGCAGTCGATCCTGATATTAATGACTGTATGAAGATGAAGGCTAGAATGACATTCGTGAGCTC
ATCTAAGTTATCGAAGTTCCTCTTGACCGAGGTACGTTTTTATAGTGTTGGGATACAGACCATGTTTGAAAATATACTTAATTT
>PhRXLR49_700 PLHAL7000084
GAAAAGGATAGGCTGCAGGATTGCAGAAAACAATGATTTGCGAATATTTAACTATTGTAAAGCCGGCTCCAGAATTTAAGCAAGGCC
CTAGAGGTCTCACTTATCAACTTTCTCTTCTTATCTCACTACCATCTCCTCCGCCCTACCTTCTGCAACGATGCGCCTTCTCGTACT
ACTACCGGCTGTGATGGCATCAACACTTCTGGCGACTAGTAGTGCCGTTGTCAATCCTGACCCCATTCATCATGCCAACTCTTTACA
AGTAGCTTCGAAAGTTAATATTGCTGCTTTAAAGAATGGTCCTCGTGCTCTGAGAACCGCTGGAACAGTCCCAAAGGAGAGGCGCTT
AGGAGATACATACTATCCCAGCGCTTACTCCTATACTGGCGGGAATAATTATGGATTCAACAACTATTCTCCCAACAGCAACGGCTA
TCAGAACACTTACCCTACAAGATACGGCAACCAGAAGCGAGATGACAGTGATGATTCCAGCAGCGAAGATAGCAAAGACGATTCTAG
CCACGATTAGCCGCATACCTCACCCGCAGTCGATCCTGATATTAATGACTGTATGAAGATGAAGGCTAGAATGACATTCGTGAGCTC
ATCTAAGTTATCGAAGTTCCTCTTGACCGAGGTACGTTTTTATAGTGTTGGGATACAGACCATGTTTGAAAATATACTTAATTT
>PhRXLR49_730 PLHAL7300503
GAAAAGGATAGGCTGCAGGATTGCAGAAAACAATGATTTGCGAATATTTAACTATTGTAAAGCCGGCTCCAGAATTTAAGCAAGGCC
CTAGAGGTCTCACTTATCAACTTTCTCTTCTTATCTCACTACCATCTCCTCCGCCCTACCTTCTGCAACGATGCGCCTTCTCGTACT
ACTACCGGCTGTGATGGCATCAACACTTCTGGCGACTAGTAGTGCCGTTGTCAATCCTGACCCCATTCATCATGCCAACTCTTTACA
AGTAGCTTCGAAAGTTAATATTGCTGCTTTAAAGAATGGTCCTCGTGCTCTGAGAACCGCTGGAACAGTCCCAAAGGAGAGGCGCTT
AGGAGATACATACTATCCCAGCGCTTACTCCTATACTGGCGGGAATAATTATGGATTCAACAACTATTCTCCCAACAGCAACGGCTA
TCAGAACACTTACCCTACAAGATACGGCAACCAGAAGCGAGATGACAGTGATGATTCCAGCAGCGAAGATAGCAAAGACGATTCTAG
CCACGATTAGCCGCATACCTCACCCGCAGTCGATCCTGATATTAATGACTGTATGAAGATGAAGGCTAGAATGACATTCGTGAGCTC
ATCTAAGTTATCGAAGTTCCTCTTGACCGAGGTACGTTTTTATAGTGTTGGGATACAGACCATGTTTGAAAATATACTTAATTT

>PhRXLR52_100 PLHAL100A10211
ACTTTCCAATTTCCTCTCCTGAGCTCACCGCCTTCGCTTTATCTCAACTCCTCCACAATGCAATTCCACCTACTCGTCATGACAACA
ATTGCAGCTTCGTTTGCAGCGACTGGTAGTGCTCTCCCGCACACCAACGTATTACCCAAAATCGGGACTCTAAGAGGTGCAATTAAC
AATGACGCTGCCACCTTCAATGGTCGGGCACTGAGAAACACCGAAAATCGCGGCTTGATTGGCGACGATTCCGACAGCAGCATCAGC
GATTCTGATTCTGAAGCAAAGGAATACCGCGCTTACAAATCTCATAAGGAACACTTCGGCTACCAAATGCCATAGAAAACTGGCTAT
GACGATAATCGCAAGTAACAAGACAAGACCTTGTACAGTTAAACAATGTAGACTTTCTAGTGTTTGTAACCTTGAAAATACAATTGT
GAGAC
>PhRXLR52_334 PLHAL3340014
ACTTTCCAATTTCCTCTCCTGAGCTCACCGCCTTCGCTTTATCTCAACTCCTCCACAATGCAATTCCACCTACTCGTCATGACAACA
ATTGCAGCTTCGTTTGCAGCGACTGGTAGTGCTCTCCCGCACACCAACGTATTACCCAAAATCGGGACTCTAAGAGGTGCAATTAAC
AATGACGCTGCCACCTTCAATGGTCGGGCACTGAGAAACACCGAAAATCGCGGCTTGATTGGCGACGATTCCGACAGCAGCATCAGC
GATTCTGATTCTGAAGCAAAGGAATACCGCGCTTACAAATCTCATAAGGAACACTTCGGCTACCAAATGCCATAGAAAACTGGCTAT
GACGATAATCGCAAGTAACAAGACAAGACCTTGTACAGTTAAACAATGTAGACTTTCTAGTGTTTGTAACCTTGAAAATACAATTGT
GAGAC
>PhRXLR52_703 PLHAL7030021
ACTTTCCAATTTCCTCTCCTGAGCTCACCGCCTTCGCTTTATCTCAACTCCTCCACAATGCAATTCCACCTACTCGTCATGACAACA
ATTGCAGCTTCGTTTGCAGCGACTGGTAGTGCTCTCCCGCACACCAACGTATTACCCAAAATCGGGACTCTAAGAGGTGCAATTAAC
AATGACGCTGCCACCTTCAATGGTCGGGCACTGAGAAACACCGAAAATCGCGGCTTGATTGGCGACGATTCCGACAGCAGCATCAGC
GATTCTGATTCTGAAGCAAAGGAATACCGCGCTTACAAATCTCATAAGGAACACTTCGGCTACCAAATGCCATAGAAAACTGGCTAT
GACGATAATCGCAAGTAACAAGACAAGACCTTGTACAGTTAAACAATGTAGACTTTCTAGTGTTTGTAACCTTGAAAATACAATTGT
GAGAC
>PhRXLR52_710 PLHAL7100035
ACTTTCCAATTTCCTCTCCTGAGCTCACCGCCTTCGCTTTATCTCAACTCCTCCACAATGCAATTCCACCTACTCGTCATGACAACA
ATTGCAGCTTCGTTTGCAGCGACTGGTAGTGCTCTCCCGCACACCAACGTATTACCCAAAATCGGGACTCTAAGAGGTGCAATTAAC
AATGACGCTGCCACCTTCAATGGTCGGGCACTGAGAAACACCGAAAATCGCGGCTTGATTGGCGACGATTCCGACAGCAGCATCAGC
GATTCTGATTCTGAAGCAAAGGAATACCGCGCTTACAAATCTCATAAGGAACACTTCGGCTACCAAATGCCATAGAAAACTGGCTAT
GACGATAATCGCAAGTAACAAGACAAGACCTTGTACAGTTAAACAATGTAGACTTTCTAGTGTTTGTAACCTTGAAAATACAATTGT
GAGAC
>PhRXLR52_304 PLHAL3040017
ACTTTCCAATTTCCTCTCCTGAGCTCACCGCCTTCGCTTTATCTCAACTCCTCCACAATGCAATTCCACCTACTCGTCATGACAACA
ATTGCAGCTTCGTTTGCAGCGACTGGTAGTGCTCTCCCGCACACCAACGTATTACCCAAAATCGGGACTCTAAGAGGTGCAATTAAC
AATGACGCTGCCACCTTCAATGGTCGGGCACTGAGAAACACCGAAAATCGCGGCTTGATTGGCGACGATTCCGACAGCAGCATCAGC
GATTCTGATTCTGAAGCAAAGGAATACCGCGCTTACAAATCTCATAAGGAACACTTCGGCTACCAAATGCCATAGAAAACTGGCTAT
GACGATAATCGCAAGTAACAAGACAAGACCTTGTACAGTTAAACAATGTAGACTTTCTAGTGTTTGTAACCTTGAAAATACAATTGT
GAGAC
>PhRXLR52_700 PLHAL7000005
ACTTTCCAATTTCCTCTCCTGAGCTCACCGCCTTCGCTTTATCTCAACTCCTCCACAATGCAATTCCACCTACTCGTCATGACAACA
ATTGCAGCTTCGTTTGCAGCGACTGGTAGTGCTCTCCCGCACACCAACGTATTACCCAAAATCGGGACTCTAAGAGGTGCAATTAAC
AATGACGCTGCCACCTTCAATGGTCGGGCACTGAGAAACACCGAAAATCGCGGCTTGATTGGCGACGATTCCGACAGCAGCATCAGC
GATTCTGATTCTGAAGCAAAGGAATACCGCGCTTACAAATCTCATAAGGAACACTTCGGCTACCAAATGCCATAGAAAACTGGCTAT

GACGATAATCGCAAGTAACAAGACAAGACCTTGTACAGTTAAACAATGTAGACTTTCTAGTGTTTGTAACCTTGAAAATACAATTGT
GAGAC
>PhRXLR52_730 PLHAL7300025
ACTTTCCAATTTCCTCTCCTGAGCTCACCGCCTTCGCTTTATCTCAACTCCTCCACAATGCAATTCCACCTACTCGTCATGACAACA
ATTGCAGCTTCGTTTGCAGCGACTGGTAGTGCTCTCCCGCACACCAACGTATTACCCAAAATCGGGACTCTAAGAGGTGCAATTAAC
AATGACGCTGCCACCTTCAATGGTCGGGCACTGAGAAACACCGAAAATCGCGGCTTGATTGGCGACGATTCCGACAGCAGCATCAGC
GATTCTGATTCTGAAGCAAAGGAATACCGCGCTTACAAATCTCATAAGGAACACTTCGGCTACCAAATGCCATAGAAAACTGGCTAT
GACGATAATCGCAAGTAACAAGACAAGACCTTGTACAGTTAAACAATGTAGACTTTCTAGTGTTTGTAACCTTGAAAATACAATTGT
GAGAC

>PhRXLR58_100 PLHAL100A10168
AATTTTCTTCGTCGTTCGTTCCAGGTGTGAAGTTTACTCAGTTGCCATGCGAATGAGCTCTTTCCTCCTTATACCCACGACAGCGAT
AGTTGCTGGTTGCGGTGCCGTCTCAGCATATAGACGACCGAGACTATTGGGTTCGAAGCTGCCTGATGAAGTCATGTCAGCGAAGGA
TGCTGTTTCAGCGAATAGTGCACGATTTCTAAGTAGCAAACCTGACAGAGTCAATGTCATTTATGCGCCAGAGAAAGAAGAAAGAAT
GATGAAAGGCATGGACGACATTCTCAATCTTGTGCGAGACAGCAGCAGCCTAGAAAAGATTGTTCCTCACATACCACCTTCTGAATT
AAAGAAAGACTTGGAAACTTTCGGGACATTTTCTATTGGTCCGACCCAATTATCGCGATCGCATGATCAGTACCCTGAACTTGTGAC
ACGAATGAAAGAGAATGTTGCGAAAGCTACAAACCAGCAAGCACCAGGCCAATTTTTTGGACTATTGCCACAAAATTCAGCTCCACA
TCTCAATCAATATCCAGTAAGCCATGCTGTAGACCACACGCCGCAAGTCCCAAGTGGATATTCTCCTCAAAATCAGCACGTTGGCAA
TCCTAATGGACAAATCTCTGCACAACATCAGCAAATTGGCTCTCCGTATGCACAAGCACAACAGCA
>PhRXLR58_334 PLHAL3340150
AATTTTCTTCGTCGTTCGTTCCAGGTGTGAAGTTTACTCAGTTGCCATGCGAATGAGCTCTTTCCTCCTTATACCCACGACAGCGAT
AGTTGCTGGTTGCGGTGCCGTCTCAGCATATAGACGACCGAGACTATTGGGTTCGAAGCTGCCTGATGAAGTCATGTCAGCGAAGGA
TGCTGTTTCAGCGAATAGTGCACGATTTCTAAGTAGCAAACCTGACAGAGTCAATGTCATTTATGCGCCAGAGAAAGAAGAAAGAAT
GATGAAAGGCATGACGACATTCTCAATCTTGTGCGAGACAGCAGCAGCCTAGAAAAGATTGTTCCTCACATACCACCTTCTGAATTA
AAGAAAGACTTGGAAACTTTCGGGACATTTTCTATTGGTCCGACCCAATTATCGCGATCGCATGATCAGTACCCTGAACTTGTGACA
CGAATGAAAGAGAATGTTGCGAAAGCTACAAACCAGCAAGCACCAGGCCAATTTTTTGGACTATTGCCACAAAATTCAGCTCCACAT
CTCAATCAATATCCAGTAAGCCATGCTGTAGACCACACGCCGCAAGTCCCAAGTGGATATTCTCCTCAAAATCAGCACGTTGGCAAT
CCTAATGGACAAATCTCTGCACAACATCAGCAAATTGGCTCTCCGTATGGACAAGCACAACAGCA
>PhRXLR58_703 PLHAL7030202
AATTTTCTTCGTCGTTCGTTCCAGGTGTGAAGTTTACTCAGTTGCCATGCGAATGAGCTCTTTCCTCCTTATACCCACGACAGCGAT
AGTTGCTGGTTGCGGTGCCGTCTCAGCATATAGACGACCGAGACTATTGGGTTCGAAGCTGCCTGATGAAGTCATGTCAGCGAAGGA
TGCTGTTTCAGCGAATAGTGCACGATTTCTAAGTAGCAAACCTGACAGAGTCAATGTCATTTATGCGCCAGAGAAAGAAGAAAGAAT
GATGAAAGGCATGGACGACATTCTCAATCTTGTGCGAGACAGCAGCAGCCTAGAAAAGATTGTTCCTCACATACCACCTTCTGAATT
AAAGAAAGACTTGGAAACTTTCGGGACATTTTCTATTGGTCCGACCCAATTATCGCGATCGCATGATCAGTACCCTGAACTTGTGAC
ACGAATGAAAGAGAATGTTGCGAAAGCTACAAACCAGCAAGCACCAGGCCAATTTTTTGGACTATTGCCACAAAATTCAGCTCCACA
TCTCAATCAATATCCAGTAAGCCATGCTGTAGACCACACGCCGCAAGTCCCAAGTGGATATTCTCCTCAAAATCAGCACGTTGGCAA
TCCTAATGGACAAATCTCTGCACAACATCAGCAAATTGGCTCTCCGTATGGACAAGCACAACAGCA
>PhRXLR58_710 PLHAL7100249
AATTTTCTTCGTCGTTCGTTCCAGGTGTGAAGTTTACTCAGTTGCCATGCGAATGAGCTCTTTCCTCCTTATACCCACGACAGCGAT
AGTTGCTGGTTGCGGTGCCGTCTCAGCATATAGACGACCGAGACTATTGGGTTCGAAGCTGCCTGATGAAGTCATGTCAGCGAAGGA
TGCTGTTTCAGCGAATAGTGCACGATTTCTAAGTAGCAAACCTGACAGAGTCAATGTCATTTATGCGCCAGAGAAAGAAGAAAGAAT
GATGAAAGGCATGGACGACATTCTCAATCTTGTGCGAGACAGCAGCAGCCTAGAAAAGATTGTTCCTCACATACCACCTTCTGAATT
AAAGAAAGACTTGGAAACTTTCGGGACATTTTCTATTGGTCCGACCCAATTATCGCGATCGCATGATCAGTACCCTGAACTTGTGAC
ACGAATGAAAGAGAATGTTGCGAAAGCTACAAACCAGCAAGCACCAGGCCAATTTTTGGACTATTGCCACAAAATTCAGCTCCACAT
CTCAATCAATATCCAGTAAGCCATGCTGTAGACCACACGCCGCAAGTCCCAAGTGGATATTCTCCTCAAAATCAGCACGTTGGCAAT
CCTAATGGACAAATCTCTGCACAACATCAGCAAATTGGCTCTCCGTATGGACAAGCACAACAGCA
>PhRXLR58_304 PLHAL3040169
AATTTTCTTCGTCGTTCGTTCCAGGTGTGAAGTTTACTCAGTTGCCATGCGAATGAGCTCTTTCCTCCTTATACCCACGACAGCGAT
AGTTGCTGGTTGCGGTGCCGTCTCAGCATATAGACGACCGAGACTATTGGGTTCGAAGCTGCCTGATGAAGTCATGTCAGCGAAGGA
TGCTGTTTCAGCGAATAGTGCACGATTTCTAAGTAGCAAACCTGACAGAGTCAATGTCATTTATGCGCCAGAGAAAGAAGAAAGAAT
GATGAAAGGCATGGACGACATTCTCAATCTTGTGCGAGACAGCAGCAGCCTAGAAAAGATTGTTCCTCACATACCACCTTCTGAATT
AAAGAAAGACTTGGAAACTTTCGGGACATTTTCTATTGGTCCGACCCAATTATCGCGATCGCATGATCAGTACCCTGAACTTGTGAC
ACGAATGAAAGAGAATGTTGCGAAAGCTACAAACCAGCAAGCACCAGGCCAATTTTTTGGACTATTGCCACAAAATTCAGCTCCACA
TCTCAATCAATATCCAGTAAGCCATGCTGTAGACCACACGCCGCAAGTCCCAAGTGGATATTCTCCTCAAAATCAGCACGTTGGCAA
TCCTAATGGACAAATCTCTGCACAACATCAGCAAATTGGCTCTCCGTATGCACAAGCACAACAGCA
>PhRXLR58_700 PLHAL7000103
AATTTTCTTCGTCGTTCGTTCCAGGTGTGAAGTTTACTCAGTTGCCATGCGAATGAGCTCTTTCCTCCTTATACCCACGACAGCGAT
AGTTGCTGGTTGCGGTGCCGTCTCAGCATATAGACGACCGAGACTATTGGGTTCGAAGCTGCCTGATGAAGTCATGTCAGCGAAGGA
TGCTGTTTCAGCGAATAGTGCACGATTTCTAAGTAGCAAACCTGACAGAGTCAATGTCATTTATGCGCCAGAGAAAGAAGAAAGAAT
GATGAAAGGCATGGACGACATTCTCAATCTTGTGCGAGACAGCAGCAGCCTAGAAAAGATTGTTCCTCACATACCACCTTCTGAATT
AAAGAAAGACTTGGAAACTTTCGGGACATTTTCTATTGGTCCGACCCAATTATCGCGATCGCATGATCAGTACCCTGAACTTGTGAC
ACGAATGAAAGAGAATGTTGCGAAAGCTACAAACCAGCAAGCACCAGGCCAATTTTTTGGACTATTGCCACAAAATTCAGCTCCACA
TCTCAATCAATATCCAGTAAGCCATGCTGTAGACCACACGCCGCAAGTCCCAAGTGGATATTCTCCTCAAAATCAGCACGTTGGCAA
TCCTAATGGACAAATCTCTGCACAACATCAGCAAATTGGCTCTCCGTATGGACAAGCACAACAGCA
>PhRXLR58_730 PLHAL7300320
AATTTTCTTCGTCGTTCGTTCCAGGTGTGAAGTTTACTCAGTTGCCATGCGAATGAGCTCTTTCCTCCTTATACCCACGACAGCGAT
AGTTGCTGGTTGCGGTGCCGTCTCAGCATATAGACGACCGAGACTATTGGGTTCGAAGCTGCCTGATGAAGTCATGTCAGCGAAGGA

TGCTGTTTCAGCGAATAGTGCACGATTTCTAAGTAGCAAACCTGACAGAGTCAATGTCATTTATGCGCCAGAGAAAGAAGAAAGAAT
GATGAAAGGCATGGACGACATTCTCAATCTTGTGCGAGACAGCAGCAGCCTAGAAAAGATTGTTCCTCACATACCACCTTCTGAATT
AAAGAAAGACTTGGAAACTTTCGGGACATTTTCTATTGGTCCGACCCAATTATCGCGATCGCATGATCAGTACCCTGAACTTGTGAC
ACGAATGAAAGAGAATGTTGCGAAAGCTACAAACCAGCAAGCACCAGGCCAATTTTTTGGACTATTGCCACAAAATTCAGCTCCACA
TCTCAATCAATATCCAGTAAGCCATGCTGTAGACCACACGCCGCAAGTCCCAAGTGGATATTCTCCTCAAAATCAGCACGTTGGCAA
TCCTAATGGACAAATCTCTGCACAACATCAGCAAATTGGCTCTCCGTATGGACAAGCACAACAGCA
